# A systems approach to understanding injury care in LMICs using causal loop diagrams

**DOI:** 10.1136/bmjgh-2025-023069

**Published:** 2026-06-21

**Authors:** Justine I Davies, Kathryn Chu, Lucia D’Ambruoso, Laura Bojke, Rene English, Heike Geduld, Sa’Ad Lahri, Hassan Mahomed, Richard Matzopoulos, Antuela A Tako

**Affiliations:** 1 Department of Applied Health Research, University of Birmingham, Birmingham, UK; 2 Centre for Global Surgery, Stellenbosch University, Stellenbosch, South Africa; 3 Centre for Global Development, Institute of Applied Health Sciences, School of Medicine, Medical Sciences and Nutrition, University of Aberdeen Institute of Applied Health Sciences, Aberdeen, UK; 4 Centre for Health Economics, York University, York, UK; 5 Department of Health Systems and Public Health, Stellenbosch University, Stellenbosch, Western Cape, South Africa; 6 Emergency and Clinical Services Support, Western Cape Government Department of Health and Wellness, Cape Town, South Africa; 7 Department of Emergency Medicine, Faculty of Medicine and Health Sciences, Stellenbosch University, Cape Town, South Africa; 8 Metro Health Services, Western Cape Department of Health and Wellness, Western Cape Government, Cape Town, South Africa; 9 Division of Health Systems and Public Health, Department of Global Health, Faculty of Medicine and Health Sciences, Stellenbosch University, Stellenbosch, South Africa; 10 School of Public Health, Faculty of Health Sciences, University of Cape Town, Rondebosch, South Africa; 11 Burden of Disease Research Unit, South African Medical Research Council, Cape Town, South Africa; 12 Nottingham Business School, Nottingham Trent University, Nottingham, UK

**Keywords:** Health policy, Health services research, Health systems, Injury

## Abstract

**Background:**

Around 40% of mortality after injuries is avoidable with access to quality care. Investments in interventions which maximise the number of injured persons who access and receive quality care are paramount, especially in low-resourced settings. Selecting such interventions within complex adaptive health systems is challenging due to the multiple interactions within these systems.

**Methods:**

We created a conceptual map, or causal loop diagram (CLD), representing the health system for injury care, focused on challenges to improving patient outcomes in low- or middle-income countries (LMICs). We used the stages of seeking, reaching, receiving and remaining in care as the care pathway framework to conceptualise the diagram. Based on expert opinion and literature searches, we elicited micro (patient and their family), meso (health service) and macro (country context) factors which affect access to quality care at each stage of the care pathway and mapped interactions between them. Factors and their interactions were adjusted during a workshop with external experts. A CLD was created using Vensim to show relationships between all factors at all stages of the care pathway. Feedback loops within the CLD which are either reinforcing (perpetuating positive or negative change) or balancing (limiting change) were elicited. Leverage points were identified as factors which are present in multiple causal loops and are potential intervention points for maximal change.

**Results:**

We initially identified 41 factors affecting stage 1, 13 affecting stage 2, 51 affecting stage 3 and 49 affecting stage 4. Consolidating factors of a similar theme gave 45 unique factors for the overarching model; 13 micro, 20 meso and 12 macro. We constructed two CLDs—one for the overarching health system and the other focused on healthcare services at facilities. The health service-specific map contained 22 meso factors. We identified 928 reinforcing or balancing causal loops acting within the health system for injury care affecting health outcomes. Factors acting as leverage points involved in the largest number of feedback loops relate to trust in or perceptions of the health system (748 loops and 694 loops, respectively) and willingness to seek care (636 loops). The health service-focused CLD identified funding and the provision of quality care as the main leverage points (112 loops each).

**Discussion:**

Our work, using injury care as an exemplar, moves beyond understanding the impact of factors on patient outcomes in silos. Rather, in considering comprehensive connections between all factors on outcomes, we show interactions which could lead to unintended consequences and rapidly worsen or improve health outcomes. Our identification of trust and willingness to seek care as key leverage points suggests these should be focal points for research and investment.

**WHAT IS ALREADY KNOWN ON THIS TOPIC**

- Health systems are complex dynamic systems hence are difficult to assess.
- Health systems contain many interacting factors with results of interventions potentially having unpredictable downstream effects on quality of care delivered.
- Few, if any, studies have attempted to understand a whole health system’s ability to deliver quality care.

**WHAT THIS STUDY ADDS**

- This study aims to describe interactions between factors in a whole health system, using injury care as an exemplar, from the point of injury to rehabilitation to optimal function.
- We have found multiple interacting factors affecting patient outcomes.
- Some factors, such as trust in and perceptions of the health system, are likely to act as leverage points for change, having multiple effects on the health systems’ ability to deliver quality care and optimal patient outcomes throughout the patient journey.

**HOW THIS STUDY MIGHT AFFECT RESEARCH, PRACTICE OR POLICY**

- Knowledge of the factors which have most influence on the ability of the health system to improve patient outcomes should assist policymakers in planning health system improvement initiatives.
- In providing an understanding of the interactions between factors acting within the health system, researchers may be able to better select interventions and outcomes for studies.

## Introduction

 Low- or middle-income countries (LMICs) suffer 85% of global injury deaths and disease burden.^1 2^ Access to quality health services after injuries can reduce this burden of death and disability, with estimates suggesting that 30 to 40% of mortality could be reduced if patients in LMICs had timely access to quality care after injuries.^3^,^5^ This would require that health systems facilitate patients who have been injured to recognise the need to seek healthcare, that there are means to reach that care in a timely manner and that the healthcare provided is appropriate to manage the patient’s injury until optimal rehabilitation is achieved. In high-income countries, where health systems are better resourced, public health messaging and investments in health literacy maximise injured patient’s care-seeking behaviour, and integrated health services link patients to prehospital emergency care and thence to healthcare facilities and rehabilitation.^6^,^8^ Whereas in many LMICs, investment in public health messaging and integrated health systems for injury care has not been prioritised.^9^ This contributes to a large gap in outcomes between high- and low- or middle-income countries; differentially structured injury and governance systems in LMICs also result in divergent outcomes across many of these settings.^10^,^12^

While investment in health systems is undoubtedly needed to reduce avoidable death and disability after injury, knowing the key leverage points at which to intervene and overcome the multiple barriers which limit injured persons’ access to quality care is challenging.^13^ Indeed, a critical knowledge gap is understanding how, within a system, an intervention works. This is particularly important for injury care, which is impacted on by multiple factors within the complex healthcare systems in which it is provided. Use of ambulance services provides a prime example of the need to understand complexity within contexts; while these services are known to reduce delays to care and are associated with reduced mortality and morbidity in some contexts, in others, as we have shown, they may be deleterious.^11^ The barriers to the effectiveness of interventions must be understood in the contexts in which they might be applied if they are to be deployed efficiently.

Unfortunately, most policy or planning decisions are informed by research exploring effects of a small number of variables acting on single patient or process outcomes using simple multivariable models.^14 15^ The field of implementation research expands knowledge by shedding some light on why evidence-based interventions may or may not work within the contexts they are implemented. However, even broad tools to understand contexts, such as the Consolidated Framework for Implementation Research, suffer criticism of inappropriate and superficial application.^16^ Additionally, while implementation research broadens understanding beyond effectiveness, its outputs generally focus only on the specific intervention in question and do not address the effects of barriers on the whole health system. Compounding these issues, most research on interventions to improve health after injury has focused only on care provided at the healthcare facility and does not consider the whole patient care pathway. Use of a systems-based approach can provide a framework to holistically understand the complexity of a system and interactions among the multiple factors within it.^17^

With increasing recognition of the importance of providing healthcare for persons who have been injured comes an opportunity to develop a conceptual map, or ‘causal loop diagram’ (CLD), of services with a focus on access to quality care. The use of whole systems approaches to address health system problems in LMIC settings is relatively unexplored.^18^ We aimed to produce an expert-informed CLD for injury care in LMICs which will allow health policymakers, planners and researchers to conceptualise the key drivers and barriers to good health outcomes from the point of injury to optimal rehabilitation. It will also drive efforts on data collection for integration and synthesis to understand the quantitative nature of these relationships.

## Methods

Guided by systems thinking methodologies, we developed a CLD that represents a conceptual model of the injury care system.^1719^,^22^ CLDs are visual tools used to synthesise existing knowledge and are especially suitable for representing the interconnected nature of complex systems and identifying key influencing factors in implementation of interventions. The CLD development was informed by expert knowledge, literature searches and external-expert consultation.

### Part 1: problem conceptualisation

To help consider and identify the boundaries for examining the injury healthcare system, we conceptualised the problem and aims, including defining the care context and setting. The core team (JID, KC, LB, LD’A and AAT (online supplemental material 1)) agreed the broad frameworks to be used during our in-person discussions. We agreed on the underlying framework for patients’ care pathways based on Thaddeus and Maine’s Three Delays framework: (1) seeking care (from injury occurrence to making the decision to seek care), (2) reaching care (from the decision to seek care to reaching definitive care (as appropriate for the person’s injury)), (3) receiving care (from reaching definitive care until discharge from the acute admission) and (4) remaining in care—added to recognise the chronicity of injury care (receiving care after discharge until rehabilitation to optimal function for the patient and their injury).^13 23^ We excluded injury prevention, as interventions are likely to be distant from health services or require involvement of policy for wider country infrastructure, and hence considered not feasible to address within this work.

We conceptualised injury as per the WHO definition of a ‘bodily lesion resulting from acute exposure to energy (mechanical, thermal, electrical, chemical or radiant) in amounts that exceed the threshold of physiological tolerance’.^24^ We focused on moderate to severe injuries, given their likely need for health services. We considered quality care according to the Institute of Medicine/WHO domains of healthcare quality.^25^ In brief, ‘effective’ refers to receiving treatment known to improve patient outcomes; ‘safe’ refers to care delivered with minimal harm; ‘timely’ refers to minimising harmful delays; ‘patient-centred’ refers to being respectful to patients’ needs and values; ‘equitable’ means that quality does not vary with individual’s characteristics; and ‘integrated’ refers to coordinated across specialties and facilities. We considered ‘efficiency’ (avoiding waste in the health system) as related to the efficient use of resources, which is the ultimate aim of our map.

### Part 2: Model conceptualisation and development

**Figure 1:**
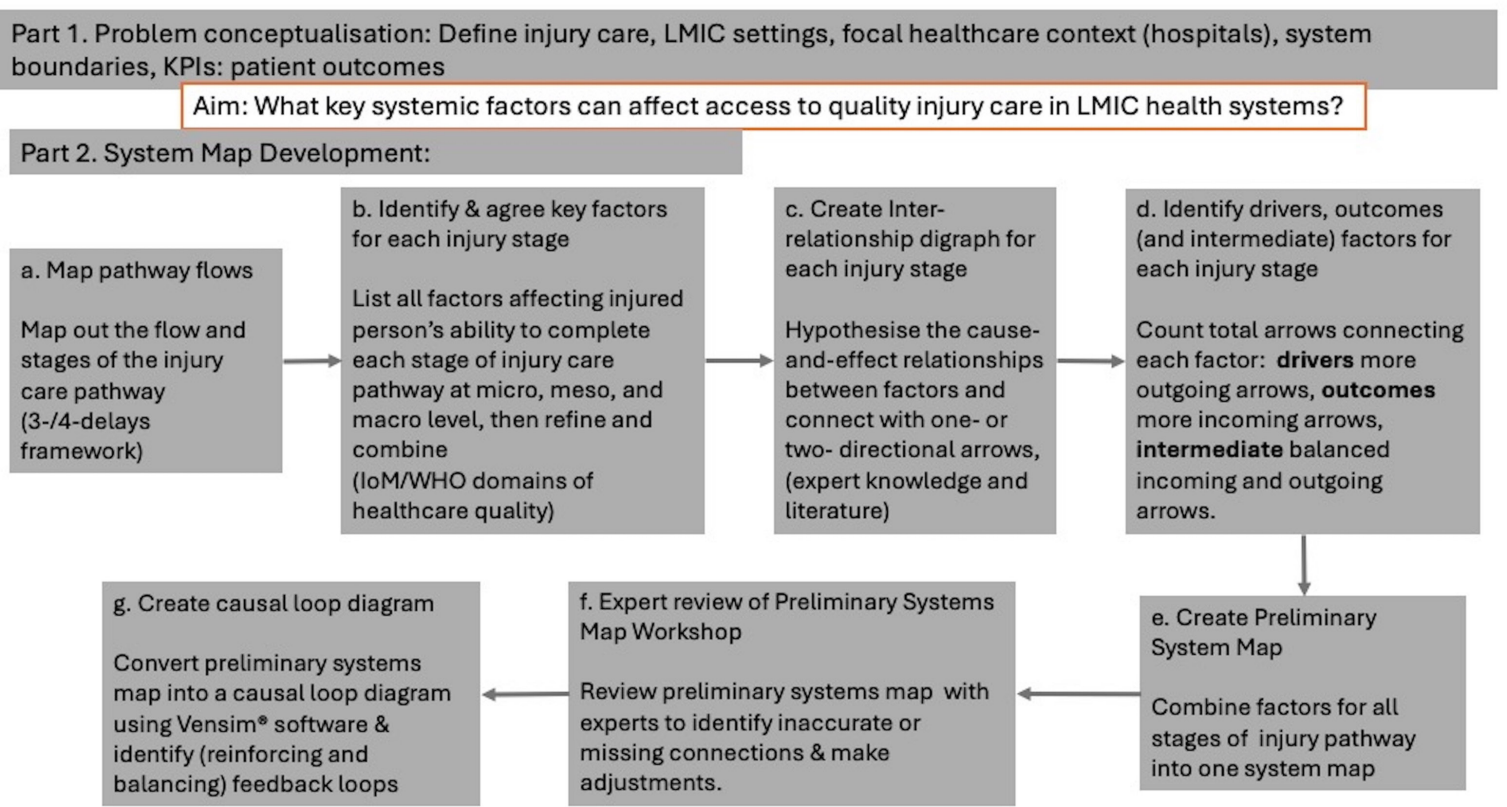
Methods schema. Overview of the steps followed to develop the system dynamics maps. IoM, Institute of Medicine; KPI, Key Performance Indicator; LMIC, low- or middle-income country.

Based on international guidance for developing systems maps and CLDs,^1719^,^22^ we followed the below steps (figure 1).

### Mapping injury care pathway flows

Based on our expert knowledge of injury care, health system dynamics, health systems and policy and social science, the core team (JID, KC, LD’A, LB and AAT—see online supplemental material 1) created a flow map of the injury care pathway (from the point of injury to optimal rehabilitation).

### Identify and agree key factors for each stage of the care pathway

Our ultimate aim was to capture drivers and facilitators which affect patient health outcomes (morbidity or mortality) via access to quality injury care in LMICs and which could be amenable to interventions. To achieve this, we captured factors affecting quality of care at each stage of the care pathway (seeking, reaching, receiving and remaining in care). Agreed frameworks to inform identification of factors at the micro level—affecting the patient, their family and immediate community—were informed by the socioecological model^26^; meso-level factors—related to health services—were informed by the WHO health system building blocks (leadership and governance; financing, service delivery; health workforce; medical products, equipment and technologies; and data and research)^27^; and macro-level factors—related to the wider context and environment in which injuries occur and health services are based—were informed using a political economy analysis lens.^28^ We took a broad approach to identifying factors to ensure completeness. Although we considered macro factors to be less amenable to interventions and likely out of scope, we included these in initial discussions to capture factors which are so integral to the provision of care for injured patients as to warrant their inclusion.

We identified factors based on our expertise in access to quality care after injuries, informed also by substantial evidence from our previous work empirically assessing delays to access of quality care in four LMICs.^10 11 29 30^ To fill gaps in knowledge, we conducted literature searches, when required.

### Create inter-relationship digraph for each injury stage

For each stage of the care pathway, we discussed and agreed links between factors based on hypothesised causal relationships and directionality. Most factors and connections were based on our expertise; where we were uncertain, our knowledge was complemented by searches of the literature (online supplemental material 2).

### Identify drivers, outcomes and intermediate factors

For each stage of the care pathway, we classified factors as drivers, outcomes or intermediate factors, defined based on the net sum of connections between factors and their directionality. Drivers had more outgoing connections, outcomes had more incoming connections and intermediate factors were balanced.^31^

### Create a preliminary conceptual map

We mapped connections between all factors, combining all stages of the care pathway. We started with the most numerically prominent drivers, outcomes or intermediate factors, then added in the less prominent ones until we were satisfied that we had a comprehensive preliminary map. Factors were renamed as required to ensure comprehension.

### External expert review of the preliminary conceptual map

The preliminary map was reviewed and refined in an in-person workshop with external experts in emergency and injury care, public health and health systems and policy from South Africa (RE, HG, S’AL, HM and RM—online supplemental material 1) in June 2025. After explaining the purpose of the work, external experts were shown the preliminary map and given time to individually consider it and list factors or connections which were inaccurate or missing; the core team were available to address any questions arising. In turn, each expert then presented their list of missing factors or inaccurate linkages, with whole-group discussion after each to agree required changes. Discussions continued until saturation was reached (defined as experts stating that they had no additional considerations to those previously presented). The map was adjusted in real time, then shared online for further comment or verification from the external experts.

### Create CLD

The map was converted into a CLD using Vensim software. Thereafter, we identified feedback loops, distinguishing reinforcing and balancing loops based on the net sum of connections and their directionality.^10^ Reinforcing loops consist of entirely positive or an even number of negative connections, thus resulting in growth or decline at an ever-increasing rate. Balancing loops have an odd number of negative connections, meaning that the factors generate resistance and eventually diminish the impact of change.

We then described the number of loops impacting the outcome ‘health outcomes’ to illustrate the complexity of the system. To understand how loops cluster and the system embeddedness of each loop, on discussion between authors, we summarised the loops qualitatively by theme and present the range of the loop length (as a proxy for system embeddedness) for each theme. Alongside, we discussed and agreed overarching interventions that might be relevant to each theme. Finally, we explored potential leverage points (those likely to have the greatest impact on the health system and thence patient outcomes, if changed) by considering the number of loops in which individual factors were involved in.^32^

## Results

**Figure 2:**
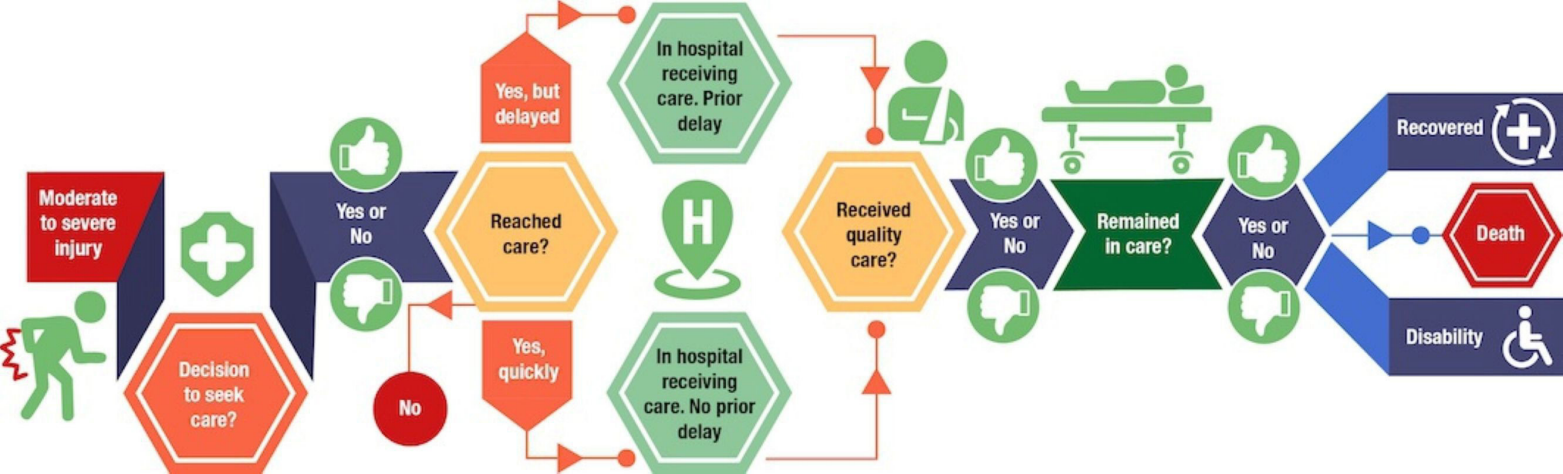
Injury care pathway concept. High-level flow map of the injury pathway showing the potential health outcomes of recovery, death or disability. The likelihood of outcomes is contingent on flow through the pathway; if there were delays in reaching care, whether care received was quality, or whether the patient remained in care, and the combinations thereof. See online supplemental file 1 for detail.

The basic flow map of the injury care pathway from being injured to an outcome of death, recovery or (long term) disability is shown in figure 2 (detailed in online supplemental material 3, figure 1a). We initially identified 41 micro-level, meso-level and macro-level factors affecting pathway stage 1; 13 affecting stage 2; 51 affecting stage 3; and 49 affecting stage 4 (online supplemental material 4, table 1). There is some similarity in factors seen across pathway stages and levels; however, this depends on perspective. For example, many factors at stage 1 were conceived of as relating to beliefs or perceptions held by patients, communities and families, whereas similar factors identified at later pathway stages were thought to be influenced by knowledge or experience of interactions with health services. This is especially seen in pathway stage 4, which occurs after the patient has experienced care. Consolidating factors of a similar theme gave 45 unique factors for the overarching model; 13 micro, 20 meso and 12 macro (online supplemental material 4, table 2) in our preliminary conceptual map. The preliminary map reviewed by the expert panel is presented in online supplemental material 5, figure 2.

**Table 1:** Final factors included in the map and related number of feedback loops

Factors included in the main injury care map	Feedback loops (n)
Health outcomes	928
GDP (macro)	874
Total available workforce (macro)	873
Patient’s trust in healthcare system (micro)	748
Patient’s perception of health system (micro)	694
Injured seeking care, n (meso)	650
Willingness to seek formal care (micro)	636
Patients in definitive care, n (meso)	636
Patients receiving higher quality injury care, n (meso)	506
Patients receiving lower quality injury care, n (meso)	506
Poverty (macro)	469
Patients remaining in care/rehab, n (meso)	458
Available funding for injury care (meso)	405
System-level funding (macro)	405
Patients taking an ambulance, n (meso)	399
Patients in definitive care via other transport, n (meso)	360
Patients taking other transport, n (meso)	360
Discrimination (meso)	360
Availability of quality injury care facility (meso)	352
Patients in definitive care via ambulance, n (meso)	348
Community conflict (macro)	331
Injured seeking non-formal care, n (micro)	321
Willingness to remain in care (micro)	304
Patients receiving higher quality rehab, n (meso)	235
Patients receiving lower quality rehab, n (meso)	235
Availability of quality injury rehab or follow-up (meso)	184
Ability to pay (micro)	148
Willingness to receive care (micro)	146
Individual wealth (micro)	137
Availability of other transport (micro)	132
Geographical accessibility (macro)	128
Availability of ambulance service (meso)	122
Patients in definitive care delayed, n (meso)	106
Patients in definitive care on time, n (meso)	102
Universal health insurance (macro)	74
Injuries, n (macro)	0
Patient needing to remain in care, n (micro)	0
Gini coefficient (macro)	0
Good Samaritan laws (macro)	0
Health education (meso)	0
Injury health policy (macro)	0
Injury severity (micro)	0
Non-formal healthcare beliefs (micro)	0
Patient or community knowledge (micro)	0
Referral pathways (meso)	0
Right of way of ambulance (macro)	0
**Health facility level factors**	**Feedback loops (n)**
Funding at the facility for injury care	112
Health outcomes	112
Provision of quality injury care	95
Patients receiving higher quality care, n	85
Patients receiving lower quality care, n	85
Continuous improvement initiatives	55
Data collection	54
Quality of leadership and governance	50
Actual capacity of injury care facility to provide injury care	40
Training for injury staff	40
Number of credentialed injury staff	39
Patient-centred care initiatives	32
Staff attrition	31
Staff morale	31
Discrimination (by staff towards patients)	20
Difference between injury care capacity vs need	17
Overload facility level	15
Staff workload	15
Dedicated facility space to provide injury care	12
Essential MET	12
Injury protocols/guidelines	12
Need for adjustment in capacity	2
Patients receiving care, n	0

Factors involved in the main and health-facility level health system dynamic maps and the number of feedback loops they are involved in.

GDP, gross domestic product; MET, Medicines, equipment, and technology.

**Table 2:** Summary of loops in the CLD and example interventions

Feedback loop theme	Loops (n)	Loop length range (min–max)	Frequent variables involved	Description of system behaviour observed	Example interventions
Patient behaviour loops	11	3–6 variables	Trust, willingness to seek/receive care, injured seeking care or non-formal care, remaining in care	These loops capture behavioural decisions such as whether patients trust the system and whether they choose to enter or stay in formal care pathways. They form the foundational behavioural drivers in the model.	Trust-building and risk communication Community engagement and outreach Health literacy and education campaigns
Transport and access loops	64	7–9 variables	Ambulance use, other transport, accessibility, on-time versus delayed definitive care	These loops model accessibility constraints, showing how delays, transport mode and geographical access shape entry into care and quality of outcomes.	Ambulance fleet expansion Emergency response time standards Transport subsidies Rural and remote infrastructure investment
Care quality loops	155	10–12 variables	Higher-quality/lower-quality injury care and rehab, remaining in rehab, availability of quality care	These loops track quality differentiation, showing how different quality levels of injury care or rehabilitation influence patient outcomes and long-term system flow.	Clinical guidelines and protocols Clinician training Workforce training and retention Equipment and supply chain reliability Facility accreditation and quality assurance
Funding and system loops	154	9–14 variables	Workforce availability, GDP, system-level funding, funding for injury care, insurance	These loops describe how resource constraints (financial, workforce and structural) impact system performance, care quality and patient flow.	Budget reallocation or expansion Workforce retainment pipeline policies Insurance expansion and risk pooling Capital investment in facilities and systems
Socioeconomic loops	358	14–17 variables	Poverty, wealth, ability to pay, conflict/violence, discrimination	These loops integrate structural inequity and societal conditions and show how they reinforce or weaken other parts of the system, affecting trust, access and quality outcomes.	Universal health coverage and financial protection Removal of user fees Antidiscrimination policy and enforcement Social protection and income support Cross-sector investment (education, housing, security)
Full-system multicausal loops	186	18–20 variables (largest)	A combination of all above categories: trust → behaviour → transport → care quality → funding → socioeconomic	These loops represent the full complexity of the injury care system, where behavioural, system, resource, equity and access factors interact in a large reinforcing structure.	Interlinked multisectoral (health, transport, finance, social protection, etc) long-term investment

CLD, causal loop diagram; GDP, gross domestic product.

Although we aimed to exclude macro factors, given that interventions to change them often lie outside the health system, we ultimately included 12 macro factors that we felt were essential to understand care pathways for injured patients. These included five variables related to financing and wealth (gross domestic product (GDP), system-level funding, Gini coefficient, poverty and universal health insurance). Our rationale being GDP contributes to the determination of the fiscal window for health which is reflected in—but not perfectly associated with—system-level funding; Gini coefficient is associated with inequitable access to care at a country level and poverty acts across more limited geographical areas; universal health insurance affects ability of individuals to access care, however, individual’s wealth (included as a micro factor) impacts individual’s access to healthcare regardless of the other financial macro factors.^33^,^36^ Non-financial macro factors included geographical accessibility—which can be alleviated by health service interventions such as improved ambulance services and ambulance right of way. Community conflict and violence, which limit the ability of ambulances to reach patients, were considered an important specific factor in the context of South Africa.^37^ This is also seen in other countries.^38^ Macro factors are further explored in our other fellowship outputs.^39^

**Figure 3:**
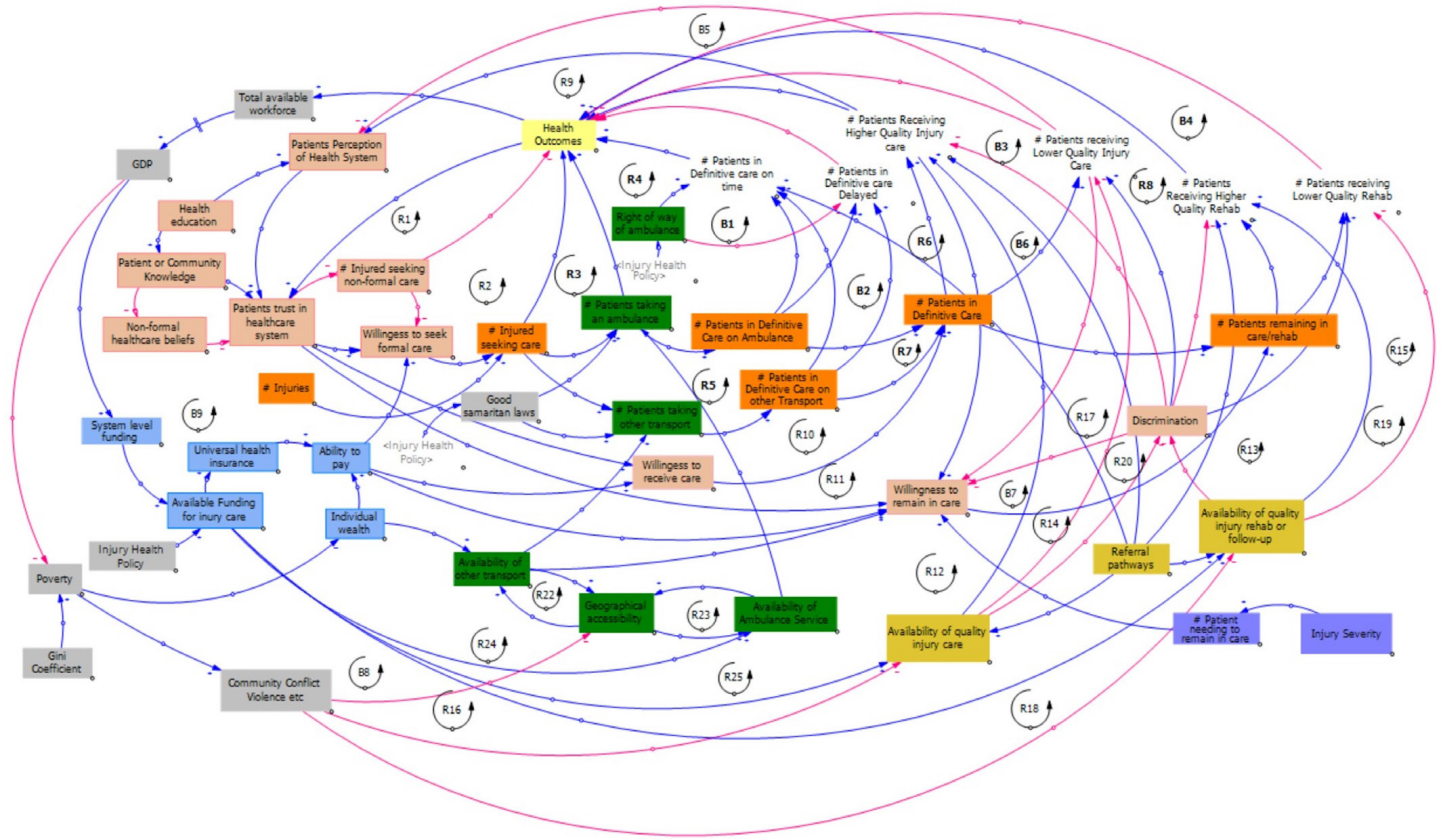
Main causal loop diagrams. Grey factors are related to policy and funding; blue, related to ability to pay; light pink, related to beliefs and knowledge; dark green, related to geographical accessibility; purple, injury-related; light orange, health service related; dark orange, the number (#) of patients transitioning through the injury care pathway; and yellow, outcomes. Positive relationships between pairs of variables are shown using blue arrows and negative relationships in red arrows. Bold arrows show the feedback loops, central to the four stages of access to healthcare which directly impact patient health outcomes. Reinforcing feedback loops are represented by the R symbol and balancing loops by a B symbol. Positive relationships between pairs of variables are shown in blue arrows and negative relationships in red font arrows. Individual and health service loops are shown in online supplemental material 5.

After the workshop, substantial changes from the initial map included assimilating acute and chronic mental health conditions into comorbidities and including human capital as a health services factor. The outputs from the workshop, including factors and their definitions are shown in online supplemental material 6, table 2, figure 3a. The complexities of the health services at facilities (representing stages 3 or 4 of the care pathway) necessitated the creation of a separate map (online supplemental material 6, figure 3b).

In the full CLD (figure 3), we identified 928 reinforcing and balancing feedback loops involving health outcomes. The final list of factors and the number of feedback loops in which each factor is involved are shown in table 1; the factors involved in each loop are shown in online supplemental material 7. The six reinforcing (R3, R4, R5, R6, R7 and R8) and four balancing (B1, B2, B3 and B4) feedback loops that form the central four stages of the injury care pathway and most directly impact on patient health outcomes are isolated in online supplemental material 8, figure 4. Online supplemental material 9, figure 5 shows the isolated health service CLD. A summary of all the feedback loops by overarching theme and the interventions which might be appropriate to consider within each theme is shown in table 2. Shorter, less structurally embedded loops were dominated by patient behaviour and perception variables, intermediate loops incorporated access and care quality constraints, longer loops included system-level funding and workforce constraints and the longest loops incorporated upstream socioeconomic determinants.

Excluding macro-level factors (considered less amenable to health systems interventions) and those capturing the number of patients moving through the system, factors involved in the largest number of feedback loops, considered leverage points, relate to patients’ trust in the system (748 loops), patients perceptions of (694 loops) and willingness to seek formal care (636 loops) (see table 1). Considering the micro-level leverage factor, trust in the system, (online supplemental material 10) illustrates the effect of increasing trust on increasing patient numbers using health services. In the short term this leads to improved health outcomes which could be maintained over the long term if investments or efficiencies in services are commensurate with or exceed demand. However, if investment does not track demand, eventually health outcomes worsen, reducing trust in the system. The mechanisms by which these effects manifest within the health services can be seen in the health service CLD in online supplemental material 9, where the number of patients receiving care impacts on ‘care capacity and need’, thence producing a ‘need to adjust capacity’ to maintain the ‘actual capacity of the injury care facility to provide injury care’. Trust also impacts on long-term health service and individual outcomes via wider effects of improved health of individuals and populations via macro factors, including ‘total workforce available’ and ‘GDP’ (loop B10).^40^ Considering the meso-level factors, discrimination was a key leverage point involved in 360 feedback loops, and examples of feedback loops involving this factor are shown in online supplemental material 11. Example causal loops involving macro factors show the impact of injury care on society and the economy and vice versa and are illustrated in online supplemental material 12.

When isolating provision of quality care and/or rehabilitation at the facility (online supplemental material 9), it can be seen to consist primarily of reinforcing feedback loops, with many factors leading to an increase in provision of quality injury care or rehabilitation, more patients receiving quality injury care and ultimately improved health outcomes. The latter can eventually result in more funding being available for injury care as part of the longer chain of influences including effects of a healthier workforce on GDP, described in the main CLD.^40^ Increased availability of funding for injury care, in turn, leads to improvements in the aforementioned factors that determine quality injury care and rehabilitation. This positive reinforcing loop is only, however, fulfilled if an improved GDP does indeed result in increased availability of funding for injury care. When excluding factors describing the number of patients moving through the system, the factors involved in the largest number of feedback loops were facility funding for injury care (112 loops), provision of quality injury care (95 loops), continuous improvement initiatives (55 loops), data collection (54) and quality of leadership and governance (50); table 1.

## Discussion

We show the complexity of the health system for injury care via identification of nearly 1000 reinforcing and balancing loops related to health outcomes in our CLD, despite aiming for parsimony in the number of factors included. We have not quantified the CLD, however, understanding this complexity—even in a qualitative or conceptual way—is necessary to understand where unintended consequences of delivering change may emerge including where small changes in some factors could result in large changes in related factors or patient outcomes.^41^ The CLD, therefore, has utility for guiding policymakers and planners towards intervention points where benefits can be maximised throughout the system. It also has utility for researchers’ understanding of which outcomes are relevant to assess when studying effects of interventions.

Our work moves beyond the usual approach seen in the scientific literature of understanding the impact of factors on patient (or process) outcomes using single or multiple variable models. It also moves beyond the system-thinking approaches employed to date which generally consider limited components of the health system.^42^,^46^ Rather, in considering comprehensive connections between factors at micro level, meso level and macro level and thence on outcomes, we show a richness of interactions that has not previously been shown. The need for such an understanding is well illustrated in a study assessing the implementation of a coaching-based intervention to improve adherence to a WHO checklist for safe childbirth. While the intervention was associated with greater adherence to the checklist, the lack of effect on mother or child morbidity or mortality outcomes seen was likely due to a failure to understand the whole health system prior to implementation, where a dearth of referral pathways once unsafe births were recognised should have been identified.^47 48^

Our CLD also allowed identification of multiple leverage points where interventions are likely to have maximal opportunity to improve—or worsen—patient outcomes. Patient trust in the health system was the most prominent leverage point and forms a useful illustration of the utility of the CLD.^32^ Our finding that trust is key to health outcomes is well supported by the literature, with this relationship coming to the fore during the recent COVID-19 pandemic, where trust was central to ensuring vaccine uptake.^49^,^52^ Our CLD shows complex relationships between trust and health education, healthcare beliefs, cultural determinants and previous health service use, each of which have been demonstrated in previous work.^53^,^55^ Given that we show the multifactorial and inter-related nature of determinants of trust, it is likely that complex interventions, acting at multiple determinants, are needed to improve it. These might include, for example, public health messaging, community outreach, shared decision making and designing services which meet patients’ wider, non-healthcare needs.^56^,^59^

A cautionary tale is also shown in our CLD; via the positive effect of trust on willingness to receive care, trust increases the number of patients in definitive care. This, ideally, increases the number of patients receiving higher quality injury care and leads to improved health outcomes, forming a reinforcing loop. However, increasing the number of patients attending care can also—both acutely and over time—reduce the quality of services provided, which in turn can negatively impact trust, given finite resources.

Discrimination is also a key leverage point. Reducing discrimination is necessary for providing patient-centred care. Reducing discrimination also improves trust, with important consequences as discussed above.^60 61^ However, like trust, discrimination is not often considered in health service planning or in quality improvement initiatives, with these focusing mainly on effectiveness and safety. Nevertheless, staff training which reduces discrimination is relatively simple to implement and our finding that discrimination is a potential leverage point suggests that more needs to be invested to understand this important area of quality care.^62 63^ Linking factors in one CLD shows all consequences which should be considered to ensure that interventions to improve trust, discrimination or other factors have continued beneficial effects.

There were multiple other reinforcing loops in our CLD which might result in virtuous or vicious circles. Indeed, in low-resourced systems, a vicious cycle could very easily emerge when system outputs are not well captured and data are sparse.^41^ For example, we show that a reduction in availability of ambulances or quality care might have a rapid deleterious effect on patient health outcomes and a more insidious effect on the economy, with knock-on negative effects on funding available for injury care and further future deleterious patient outcomes. This is an important consideration, especially given that ambulance services are being considered in LMIC settings, and there is evidence which supports that, unless these expensive interventions are implemented in a well-organised system, ambulances might worsen health outcomes.^11^

Our CLD shows the importance of macro factors including GDP, poverty and geographic isolation in improving patient outcomes.^39^ For example, quality of care is in a virtuous loop with GDP, hence improving it should be important for policymakers for driving economic growth.^3 40 64 65^ Another macro factor, geographical accessibility, was involved in 128 feedback loops in our CLD. Poor geographic accessibility can be mitigated by improving road infrastructure which results in reductions in the negative effects of isolation on other outcomes, including poverty^66^; however, the benefits accrued depend on methods of improving access. If a simple health system lens is used to address geographic accessibility of health services, the solution may be focused on developing more localised health services or improving emergency transport to centralised services; neither of which capitalise on the more generalised beneficial effects on poverty reduction from improving general infrastructure.^67^ This point shows a limitation of focusing on factors which are most proximate to the health services when considering interventions. Given that to ensure feasibility, macro factors are not widely captured in our CLD, it cannot be used for considering potential interventions at a policy level. Rather, the complexities that our CLD highlight should offer caution to planners and policymakers when considering change focused at a meso level and micro level.^68^ Indeed, when contemplating implementing interventions, consideration should be given to the wider CLD or individual feedback loops in which these factors are involved, such that potential unintended negative consequences are anticipated and mitigated when planning health services.

In addition to illustrating downstream consequences of interventions, our CLD also illustrates gaps in the medical literature which need to be filled to fully understand the impacts of interventions. Individual studies often show the effects of interventions on limited clinical or process outcomes which are proximate to the intervention. However, there is very little literature to show the consequences of interventions on more downstream outcomes. For example, although our CLD leads to the hypothesis that increased trust, over the long term could lead to health services overload and worsen patient outcomes if not accompanied by investment in services to accommodate increased demand, there is no literature that we are aware of which empirically tests this hypothesis.

## Limitations

Our study has several limitations. The author team has broad and relevant experience; however, it is likely that we omitted factors that others would consider important or included some which others would omit. External expert input, extensive discussions between authors and in-depth consideration of all factors in the final CLD aimed to mitigate against this; but only with development of the eventual full quantitative map will we understand which factors predominate. Where we were uncertain of relationships between factors, we referred to the literature. However, many of the connections between factors were not always well delineated, especially in LMICs, and it was necessary to combine knowledge of the literature with team members’ experience. On a similar theme, health system dynamics approaches often consider quantities of patients (or other ‘goods’) flowing through a system. Whereas most of the scientific literature considers the likelihood of experiencing patient or process outcomes while accounting for multiple confounding variables. Thus, connections between variables and their directionality were not numerically verifiable. The levels of uncertainty about the directionality of some variables which are presented in the map vary. For example, while we have presented transport by ambulances as having positive effects on most linked factors, this isn’t always seen in the literature.^11^ Given the multiple connections between factors and the lack of evidence in this field, we have not presented the certainty of the evidence behind the connections. A next phase of our work is to interrogate the literature to quantify connections to produce a functional quantitative health system dynamics map. However, given the scarcity of data on many connections and especially in LMICs, we were not able to achieve this at this stage. Finally, we did not consider injury prevention in this work, and its exclusion detracts from our desire to consider the system as a whole. Prevention was excluded for reasons of feasibility and because many of the interventions for injury prevention fall outside of health services, despite them ultimately reducing burden on that system. These interventions, however, are more closely linked with the macro factors, which are explored more fully in our other work.^39^

In summary, our work has illustrated that issues encountered in healthcare systems are complex problems that are difficult to solve. However, the first step towards understanding the complexity is to describe it, as we have in our CLD. By identifying the interconnections between factors in the CLD, we can illuminate much of the complexity and facilitate the understanding and action towards improving health systems for injury. Our work also acts as an exemplar for the wider health system. We have identified potential leverage points where intervention in the health system could be made, including improving patients’ trust in the healthcare system and reducing discrimination. Our map should have utility for policymakers for understanding the complexity of the health system and the high-level consequences of intervening at any of the factors. It also has utility for academics who work at the policy–academic interface to shed light on areas where evidence can be sought to more holistically determine impact of interventions implemented in a complex health system.

## Supplementary material

10.1136/bmjgh-2025-023069 online supplemental file 1

## Data Availability

Data sharing not applicable as no datasets generated and/or analysed for this study.
