# Supplementary material for: A systems approach to understanding injury care in LMICs using causal loop diagrams
Source: BMJ Glob Health. 2026 Jun 21;11(6):e023069. doi: 10.1136/bmjgh-2025-023069 (PMC13288975; doi:10.1136/bmjgh-2025-023069)
Supplement: online supplemental file 1 [file bmjgh-11-6-s001.pdf]

Understanding health system dynamics for injury care appendices

Supplementary Material 1: Team expertise

Experts involved in the development of the preliminary model

Justine Davies is a health system expert, with particular interest in access to quality care after injuries. She leads the NIHR funded Equi-Injury project (NIHR133135) to identify barriers to access to quality care in low- or middle-income countries and the NIHR funded project, Rwanda 912, to develop and implement ambulance dispatch software suitable for low resourced contexts in Rwanda (NIHR203062). She led the NIHR funded Equi-Trauma project to identify barriers to access to quality care in countries in sub-Saharan Africa. She directs a WHO Collaborating Centre on Emergency, Critical, and Operative care.

Kathryn Chu is a surgeon and health system expert with interest in access to quality care after injuries. She co-leads the NIHR funded Equi-Injury project to identify barriers to access to quality care in low- or middle-income countries. She co-led the NIHR funded Equi-Trauma project to identify barriers to access to quality care in countries in sub-Saharan Africa.

Lucia D'Ambruoso is a social scientist with an interest in community access to quality care. She is a co-investigator on Equi-Injury and Rwanda 912 projects.

Laura Bojke is a Health Economist with an interest in understanding macroeconomic consequences of improving access to interventions. She is a co-investigator on Equi-Injury and Rwanda 912 projects.

Antuela Tako is a systems expert with an interest in health system dynamics modelling. She developed the PartiSim and SIMTEGR8 approaches that guide the process of co-developing health system models with experts and other stakeholders to evaluate and improve system performance.

Additional experts involved in the workshop and subsequent iteration of the map

Rene English is a health systems expert and a public health medicine specialist. She has worked closely within the South African health system and currently works as the Head of Public Health and Health Systems at Stellenbosch University (South Africa). She is the former head of the Health Systems Trust, a non-profit organisation dedicated to improving health systems in Southern Africa.

Heike Geduld is an emergency medicine physician and with interest in access to quality care after injuries. She is responsible for Emergency Medicine co-ordination, policy and governance for the Western Cape Department (South Africa) of Health and Wellness. Heike has consulted for the WHO on Emergency Care Systems and was a co-author of the WHO/ICRC Basic Emergency Care Course (BEC).

Sa'ad Lahri is an emergency medicine physician and Head of the Clinical Unit for Emergency Medicine in the Metro East health district of the Western Cape, South Africa. He is involved in the coordination and quality improvement of emergency medicine services across the Metro East platform, with interests in emergency care systems strengthening and improving access to timely care for injured and critically ill patients.

Hassan Mahomed is a health systems expert and a public health medicine specialist who works within the Western Cape Department of Health (South Africa). He is analyses health system challenges to find solutions including fundamental health systems reform initiatives. He has been involved in the review of emergency care including injury care within the provincial health system.

Richard Matzopoulos is an injury epidemiologist. He leads research on measuring the health and social burden of violence and injury and evaluating interventions and policies that target upstream determinants. He coordinates the Violence and Injury Research Programme at the University of Cape Town.

Supplementary Material 2: Bibliography of literature accessed to define factors and causal relationships

1.Ae-Ngibise KA, Masanja H, Kellerman R, Owusu-Agyei S. Risk factors for injury mortality in rural Tanzania: a secondary data analysis. Bmj Open. 2012;2(6).

2. Aluisio AR, Barry MA, Martin KD, Mbanjumucyo G, Mutabazi ZA, Karim N, et al. Impact of emergency medicine training implementation on mortality outcomes in Kigali, Rwanda: An interrupted time-series study. African Journal of Emergency Medicine. 2019;9(1):14-20.
3. Amato S, Bonnell L, Mohan M, Roy N, Malhotra A. Comparing trauma mortality of injured patients in India and the USA: a risk-adjusted analysis. Trauma Surgery & Acute Care Open. 2021;6(1).
4. Arabi YM, Haddad S, Tamim HM, Al-Dawood A, Al-Qahtani S, Ferayan A, et al. Mortality reduction after implementing a clinical practice guidelines-based management protocol for severe traumatic brain injury. Journal of Critical Care. 2010;25(2):190-5.
5. Areas FZ, Schwarzbold ML, Diaz AP, Rodrigues IK, Sousa DS, Ferreira CL, et al. Predictors of Hospital Mortality and the Related Burden of Disease in Severe Traumatic Brain Injury: A Prospective Multicentric Study in Brazil. Frontiers in Neurology. 2019;10.
6. Bedard AF, Mata LV, Dymond C, Moreira F, Dixon J, Schauer SG, et al. A scoping review of worldwide studies evaluating the effects of prehospital time on trauma outcomes. International Journal of Emergency Medicine. 2020;13(1).
7. Bhandarkar P, Patil P, Soni KD, O'Reilly GM, Dharap S, Mathew J, et al. An Analysis of 30-Day in-Hospital Trauma Mortality in Four Urban University Hospitals Using the Australia India Trauma Registry. World Journal of Surgery. 2021;45(2):380-9.
8. Boschini LP, Lu-Myers Y, Msiska N, Cairns B, Charles AG. Effect of direct and indirect transfer status on trauma mortality in sub Saharan Africa. Injury. 2016;47(5):1118-22.
9. Brown E, Tohira H, Bailey P, Fatovich D, Pereira G, Finn J. Longer Prehospital Time was not Associated with Mortality in Major Trauma: A Retrospective Cohort Study. Prehospital Emergency Care. 2019;23(4):527-37.
10. Chao TE, Chu K, Hardcastle TC, Steyn E, Gaarder C, Hsee L, et al. Trauma care and its financing around the world. Journal of Trauma and Acute Care Surgery. 2024;97(5):e60-e4.
11. Chipendo PI, Shawar YR, Shiffman J, Razzak JA. Understanding factors impacting global priority of emergency care: a qualitative policy analysis. BMJ Glob Health. 2021;6(12).
12. Davies J, S; Chu, K; ignatowitcz, A; Odland, ML. EquiTrauma: Providing access to equitable trauma care in lower and middle income countries - an NIHR funded study 2019 [Available from: https://www.birmingham.ac.uk/research/applied-health/research/EquiTrauma.aspx.
13. Dharap SB, Kamath S, Kumar V. Does prehospital time affect survival of major trauma patients where there is no prehospital care. Journal of Postgraduate Medicine. 2017;63(3):169-75.
14. Equi-Trauma C, Odland ML, Abdul-Latif AM, Ignatowicz A, Alayande B, Appia Ofori B, et al. Equitable access to quality trauma systems in low-income and middle-income countries: assessing gaps and developing priorities in Ghana, Rwanda and South Africa. BMJ Glob Health. 2022;7(4).
15. Finklestein AD, J. A comedy of errors: the London ambulance service case study: School of Informatics, City University, UK; 1993 [Available from: http://www.inf.ed.ac.uk/teaching/courses/pi/2013_2014/notes/1/comedy_of_errors.pdf16. Ghalichi LD, J; The Equi-Injury study. Delays in seeking and reaching care for injured patients in four low- and middle-income countries. BMJ GLobal Health. 2025;Under review.
17. Haagsma JA, James SL, Castle CD, Dingels ZV, Fox JT, Hamilton EB, et al. Burden of injury along the development spectrum: associations between the Socio-demographic Index and disability-adjusted life year estimates from the Global Burden of Disease Study 2017. Inj Prev. 2020;26(Supp 1):i12-i26.
18. Hashmi ZG, Haider AH, Zafar SN, Kisat M, Moosa A, Siddiqui F, et al. Hospital-based trauma quality improvement initiatives: first step toward improving trauma outcomes in the developing world. J Trauma Acute Care Surg. 2013;75(1):60-8; discussion 8.
19. Henry JA, Reingold AL. Prehospital trauma systems reduce mortality in developing countries: a systematic review and meta-analysis. J Trauma Acute Care Surg. 2012;73(1):261-8.
20. Hondo K, Shiraishi A, Fujie S, Saitoh D, Otomo Y. In-Hospital Trauma Mortality Has Decreased in Japan Possibly Due to Trauma Education. Journal of the American College of Surgeons. 2013;217(5):850-+.
21. Kamau C, Chikophe I, Abdallah A, Mogere E. Impact of advanced trauma life support training on 30-day mortality in severely injured patients at a Kenyan tertiary center: a retrospective matched case-control study. International Journal of Emergency Medicine. 2024;17(1).
22. Krebs E, Gerardo CJ, Park LP, Nickenig Vissoci JR, Byiringiro JC, Byiringiro F, et al. Mortality-Associated Characteristics of Patients with Traumatic Brain Injury at the University Teaching Hospital of Kigali, Rwanda. World neurosurgery. 2017;102:571-82.
23. Lee J, Abdel-Aty M, Cai Q, Wang L. Effects of emergency medical services times on traffic injury severity: A random effects ordered probit approach. Traffic Injury Prevention. 2018;19(6):577-81.

24. Liu MW, Ma ZQ, Liao RL, Chen WM, Zhang BR, Zhang QJ, et al. Incidence and mortality related risk factors in patients with severe traumatic brain injury: A meta-analysis. Experimental and Therapeutic Medicine. 2025;29(4).
25. Moran CG, Lecky F, Bouamra O, Lawrence T, Edwards A, Woodford M, et al. Changing the System - Major Trauma Patients and Their Outcomes in the NHS (England) 2008–17. EClinicalMedicine. 2018;2-3:13-21.
26. Noble HE, Scott JW, Nyinawankusi JD, Uwitonze JM, Kabagema I, Maine RG, et al. The impact of data feedback on continuous quality improvement projects in Rwanda: A mixed methods analysis. Afr J Emerg Med. 2020;10(Suppl 1):S78-S84.
27. Pereira DHG, Neto JND, Melo TCD, dos Santos C, Silva ERC, Oliveira AMP, et al. Traumatic Brain Injury: in-hospital Survival Rates and the Main Predictors of in-hospital Mortality in Northeastern Brazil. Brazilian Neurosurgery-Arquivos Brasileiros De Neurocirurgia. 2024;43(03):e164-e71.
28. Pouramin P, Li CS, Busse JW, Sprague S, Devereaux PJ, Jagnoor J, et al. Delays in hospital admissions in patients with fractures across 18 low-income and middle-income countries (INORMUS): a prospective observational study. The Lancet Global health. 2020;8(5):e711-e20.
29. Razzak JA, Bhatti J, Wright K, Nyirenda M, Tahir MR, Hyder AA. Improvement in trauma care for road traffic injuries: an assessment of the effect on mortality in low-income and middle-income countries. Lancet. 2022;400(10348):329-36.
30. Rogers FB, Rittenhouse KJ, Gross BW. The golden hour in trauma: Dogma or medical folklore? Injury-International Journal of the Care of the Injured. 2015;46(4):525-7.
31. Shah SJ, Gondwe J, An S, Hester TE, Charles A. The effect of interhospital transfer on pediatric burn injury mortality in a resource-limited setting. World Journal of Surgery. 2024;48(12):2811-7.
32. Sreeharsha P, Kanna RM, Milton R, Shetty AP, Rajasekaran S. Risk factors for thirty-day morbidity and mortality after spinal trauma. European Spine Journal. 2023;32(1):110-7.
33. Uwamahoro C, Aluisio AR, Chu E, Reibling E, Mutabazi Z, Karim N, et al. Evaluation of a modified South African Triage Score as a predictor of patient disposition at a tertiary hospital in Rwanda. African Journal of Emergency Medicine. 2020;10(1):17-22.
34. Whitaker J, Edem I, Togun E, Amoah AS, Dube A, Chirwa L, et al. Health system assessment for access to care after injury in low- or middle-income countries: A mixed methods study from Northern Malawi. PLoS Med. 2024;21(1):e1004344.
35. Whitaker J, O'Donohoe N, Denning M, Poenaru D, Guadagno E, Leather AJM, et al. Assessing trauma care systems in low-income and middle-income countries: a systematic review and evidence synthesis mapping the Three Delays framework to injury health system assessments. BMJ Global Health. 2021;6(5):e004324.
36. Wintemute GJ. INJURY MORTALITY AND SOCIOECONOMIC-DEVELOPMENT - AN EXPLORATORY ANALYSIS. International Journal of Epidemiology. 1986;15(4):540-3.
37. WHO. Basic emergency care: approach to the acutely ill and injured. Geneva: WHO World Health Organization (WHO) [Available from: https://www.who.int/publications/i/item/9789241513081.

Supplementary Material 3: Conceptualisation of delays stages and factor levels

Supplementary material Figure 1a shows the detail behind Figure 1 from the main text. The flow map of the injury care pathway showing potential outcomes of recovery, death, or disability. Odds of outcomes are contingent on flow through the pathway; whether or not there were delays in reaching care, whether care received was quality, or whether the patient remained in care, and the combinations thereof. Colours represent the achievement of each delay stage as a result of achievement of previous stages. Arrows to outcomes are colours commensurate with the achievement of each delay stage and represent the probability of outcomes of recovered, death, or disability, based on combined progress through the delay stages.

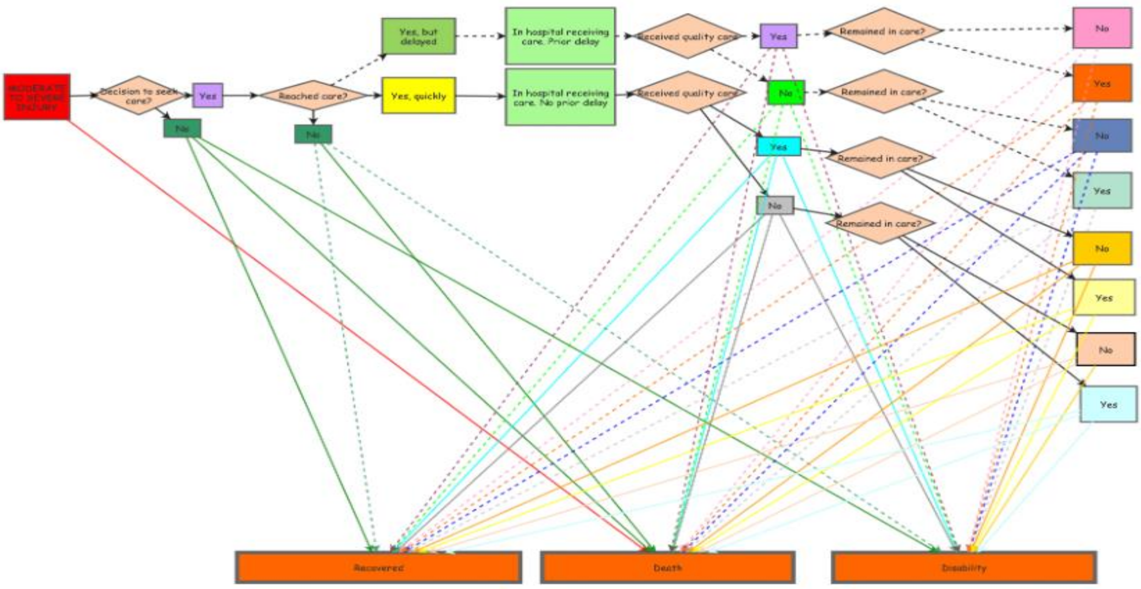
MODERATE TO SEVERE INJURY
Decision to seek care?
Yes
No
Reached care?
No
Yes, but delayed
Yes, quickly
In hospital receiving care. Prior delay
In hospital receiving care. No prior delay
Received quality care
Received quality care
Yes
No
Yes
No
Remained in care?
Remained in care?
Remained in care?
Remained in care?
No
Yes
No
Yes
No
Yes
No
Yes
Recovered
Death
Disability

We consider a minority of patients die whilst making the decision to seek care and, given that this information is difficult to capture, we have captured these people along with those who die at the scene (the arrow from moderate to severe injury to death). We also consider that a minority of people die after deciding to seek care, and before attempting to reach care, we capture these people along with those who do not reach care. We acknowledge that for some injured people, the decision to seek care or the process of attempting to reach care may take so long that the person recovers or gives up during this time; however, we believe that the numbers of these people are minimal, especially for those who have moderate to severe injuries, hence the flow from not having reached care to recovered or disabled is shown as a dotted line. At the stage of reaching care, patients can either reach care with or without delay, and this likely impacts on their subsequent outcomes at the stages of receiving or remaining in care, with increased chance of adverse outcomes for those who are delayed vs those who are not. Hence the flow diverges at this stage. Likewise, risk of adverse outcomes at the stage of remaining in care is affected by whether the patient was delayed in reaching care or received effective care or not after reaching care; hence the flow further diverges at these stages.

Supplementary Material 4: List of factors

Supplementary Material 4, Table 1 Initial list of factors considered to impact on outcomes across micro, meso, and macro levels, by care-pathway stage

**Micro factors (held by/originating in the patient, family, or community)**	**Meso factors (pertaining to/originating in the healthcare services)**	**Macro factors (pertaining to/originating in the environment in which the healthcare services and patients, families, communities are situated)**
**Stage 1 (seeking care)**		
**Perception of injury severity**<br>**Injury mechanism or type**<br><br>**Individual's co-morbidities**<br>**Perception of individual health status**<br>**Race/ethnicity/religion**<br>**Sex/gender-based decision making**<br>**Age/age-based decision making**<br>**Health education**<br>**General education**<br>**Perception of the health system**<br>**Beliefs about health and healthcare**<br>**Disease origin beliefs**<br>**Perception of traditional healers**<br>**Perception of available transport options**	**Health education (knowledge of severity)**<br>**Health education (care availability, transport)**	**Gross domestic product (GDP)**<br>**GINI coefficient**<br><br>**Poverty**<br>**Injury prevalence**<br>**Drug/substance use attributable fraction of injuries (%)**<br>**Assault-related fraction of injuries (%)**<br>**Alcohol-attributable fraction (AAF) of injuries (%)**<br>**Road traffic injury rate per 100,000 population**<br>**Labour informality rate (%)**<br>**% of urban population in informal settlements**<br>**% of population covered by UHC**<br>**Policy recognition and support for injury**<br>**% of health budget allocated to injury care**<br>**Pre-hospital EMS coverage per 100,000 population (and response time)**

Willingness to go to care<br><br>Perception or knowledge of transport cost<br>Perception of direct medical costs at facility<br>Perception of indirect medical costs<br>Individual wealth<br><br>Presence of health insurance<br>(Individual's) ability to pay<br>Community safety		Out-of-pocket health expenditure due to injury (% total health spending)<br>Catastrophic health expenditure (% of households)<br>Good Samaritan laws (presence/absence)<br><br>Geographical proximity to definitive injury care<br>Violence, climate, conflict, other disruptions at societal level
Stage 2 (reaching care)		
Injury severity<br><br>Injury mechanism or type<br>Individual wealth<br><br>Presence of health insurance<br>Availability of non-ambulance transport<br>Availability of ambulance transport<br>Direct costs of transport<br>(Individual's) ability to pay	Availability of ambulance service and dispatch centre<br>Health education on time sensitivity<br>Health education on appropriate facilities for injuries	As for Delay 1<br><br>Road conditions<br>Right of way for ambulances (legal obligation)
Stage 3 (receiving care)		
Injury severity<br>Injury type<br><br>Individual's comorbidities<br>Age-related effects on outcomes<br>Race/ethnicity/religion<br>Disease origin beliefs<br>Health education (including consent related)<br>Perceptions of need to receive care<br><br>Sex-related decision making<br>Sex-related ability to pay	Funding at the facility for injury care<br>Availability of a trauma centre<br><br>Availability of Emergency Unit (EU)<br>Availability of Operating Room (OR)<br>Availability of ICU/HDU<br>Availability of beds for injured patients<br>Appropriate numbers of staff in wards with injured patients<br>Appropriate training for staff, dependent on cadre<br>Presence of a designated trauma team<br>Credentialing for staff to deliver care to injured patients	Gross domestic product (GDP)<br>GINI coefficient (paired with injury burden)<br>Poverty<br>Injury prevalence<br>Drug/substance use attributable fraction of injuries (%)<br>Assault-related fraction of injuries (%)<br>Alcohol-attributable fraction (AAF) of injuries (%)<br>Road traffic injury rate per 100,000 population<br><br>Labour informality rate (%)<br><br><br>% of population covered by UHC

Sex-related discrimination<br>Age-related discrimination<br>Individual wealth<br>Presence of health insurance<br>Direct medical costs<br>Indirect medical costs<br>(Individual's) ability to pay	Availability of medicines/equipment/services for injury care<br>Clear inter/intra facility referral pathways<br>Conduct of regular QI cycles and data to inform them<br>Provision of patient centred care<br>Delivery of effective care<br>Delivery of safe care<br>Delivery of timely care<br>Clinical SOPs<br>Presence of age-related discrimination<br>Presence of sex related discrimination	Policy recognition and support for injury<br>% of health budget allocated to injury care<br>Out-of-pocket health expenditure due to injury (% of total health spending)<br>Catastrophic health expenditure due to injury (% of households)<br>Geographical proximity to definitive injury care<br>Violence, climate, conflict, other disruptions at societal level
Stage 4 (remaining in care)		
Perception of injury severity<br>Injury mechanism or type<br>Individual's co-morbidities<br>Perception of individual health status<br>Perception of need to remain in care)<br>General education<br>Disease origin beliefs<br>Perception of traditional healers<br>Perception of health system<br>Sex/gender-based decision making<br>Age/Age-based decision making<br>Willingness to remain in care<br>Race/ethnicity/religion<br>Direct medical costs	Need for post discharge care<br>Funding at the facility for injury rehab/ongoing care<br>Availability of rehab/ongoing care facilities<br>Appropriate numbers of staff in rehab and ongoing care<br>Appropriate training for staff, dependent on cadre<br>Credentialing for all staff required to deliver care to injured patients<br>Clinical SOPs<br>Clear inter/intra facility referral pathways<br>Availability of necessary equipment and services for rehab/ongoing care<br>Conduct of regular QI cycles and data to inform them<br>Provision of patient centred care<br>Delivery of effective care<br>Delivery of safe care<br>Delivery of timely care	As for Stage 3

**Indirect medical costs**<br>**Individual wealth**<br>**Individual's ability to pay**<br>**Presence of health insurance**<br>**Transport availability**<br>**Transport cost**<br>**Community safety**		

Table 1. Factors considered by authors to impact on each stage of the care-pathway for patients after moderate to severe injury presented by care-pathway stage and "level" of factors (micro, meso, or macro). Ambulance refers to a staffed and equipped EMS (Emergency Medical Services). EU is Emergency Unit (or department), OR is operating room, ICU is intensive care unit, HDU is high dependency unit, QI is quality improvement, GDP is Gross Domestic Product, UHC is universal health coverage including injury care. Willingness to go to/remain in care is a product of perceptions, beliefs, and knowledge. Appropriate staff include doctors, nurses, surgeons, occupational therapists, and physiotherapists. Necessary equipment and services for injury care include radiology, including XR, USS, CT, and MRI (in order of importance); laboratory services include FBC, U & E, blood type and crossmatch; medications and equipment include iv fluids, pain relief, anaesthetics, heparin, tranexamic acid; and availability of blood for transfusion, oxygen, anaesthetic equipment, rehabilitation equipment/gym, prostheses, and walking aides.

Supplementary Material 4, Table 2: consolidated factors and definitions

Factors presented in manuscript Table 1 were consolidated where there was overlap; the factors were further categorised into themes for clarity of presentation in the maps, as shown below and in the main manuscript.

Factor name	Definition	Overarching category
Injury policy	National or subnational injury policy	Policy and Funding
Good Samaritan laws	Laws to protect first responders from prosecution	Policy and Funding
Injury related funding	Funding earmarked for injury care services	Policy and Funding
(Perceptions of) Ability to pay	Ability of the patient to pay the direct and indirect costs of health care for injuries (including prehospital, hospital, and rehab)	Ability to pay
Age/age-based decision making	Any direct effects of age on outcomes and age-based decision making	Person factors
Sex/gender-based decision making	Any direct effects of sex on outcomes and sex-based decision making	Person factors
Co-morbidities	The patient's comorbid disease	Person factors
Altered mental state due to alcohol & drugs	The patient's acute mental health status	Person factors
Culture/religion/race/ethnicity	The patient's culture, religion, race, and ethnicity	Beliefs and knowledge
Alternative beliefs	Patient, family, community beliefs about alternative	Beliefs and knowledge
Health education	Availability of health education	Beliefs and knowledge
Knowledge	Patient, family, or community health/healthcare knowledge	Beliefs and knowledge
Willingness to go to care	The patient's willingness to go to care	Beliefs and knowledge
Willingness to receive care	The patient's willingness to receive care once facility is reached	Beliefs and knowledge
Willingness to remain in care	The patient's willingness to remain in care after discharge from acute care	Beliefs and knowledge
Patients choose to seek other care	The patient chooses to seek "non formal" care	Beliefs and knowledge
Violence, climate, conflict, other disruptions at societal level	Environmental factors which impact upon infrastructure	Isolation
Patient location	Where the patient lives	Isolation

Road conditions	Roads that are passable by transport to get patients to facilities	Isolation
(Perceptions of) Availability of ambulance services	Availability of staffed and equipped ambulance services	Transport related
(Perceptions of) Available transport options	Availability of other non-ambulance transport	Transport related
Awareness of the right facility	Awareness (patient, family, community, transport, or ambulance driver) of the most appropriate facility to take the patient to	Transport related
Right of way ambulance law	Laws to give ambulances the right of way over other traffic	Transport related
Injury prevalence	National or subnational prevalence of injury	Background factors
(Perceptions of) Injury severity	Severity of injury	Injury related
Injury type	Type of injury	Injury related
Need to remain in care	Clinical need to remain in care after the acute care episode	Injury related
Patient centredness/Health service experiences	Interactions with patients are respectful	Health service related
Discrimination	Discrimination against the patient due to their culture, race, religion, ethnicity, or other	Health service related
Injury ready facilities (space)	Acute care facilities have the spaces to provide care to injured patients	Health service related
Rehab-ready facilities (space)	Rehab and ongoing care facilities have the spaces to provide care to injured patients	Health service related
QI/Data	Regular quality improvement cycles informed by data	Health service related
Timeliness of care given	Care is given in a timely manner (without undue delays)	Health service related
Appropriate injury protocols and guidelines	Protocols and guidelines exist for management and rehab of the injured patient	Health service related
Essential MET (diagnostics medicines and equipment)	MET needed to treat injured patients are available	Health service related
Injury team (meaning the right number, cadres, training, and credentialed)	Team members needed to acutely manage injured patients are available, trained, and credentialed)	Health service related
Injury rehab team (meaning the right number, cadres, training, and credentialed)	Team members needed to provide ongoing care to injured patients are available, trained, and credentialed)	Health service related
Patient seeks care	The patient makes the decision to seek care	Outcomes
Patient reaches care	The patient reaches care	Outcomes

Patient receives care	The patient receives acute care	Outcomes
Patient remains in care	The patient remains in rehab or ongoing care until their functionality has been optimised	Outcomes

Supplementary Material Table 1 shows the factors included in the draft map with their definitions, and overarching categories. Factors which are similar in theme between delay-stages have been condensed into a single description.

Supplementary Material 5: Draft map (and definitions of factors) presented to workshop attendees

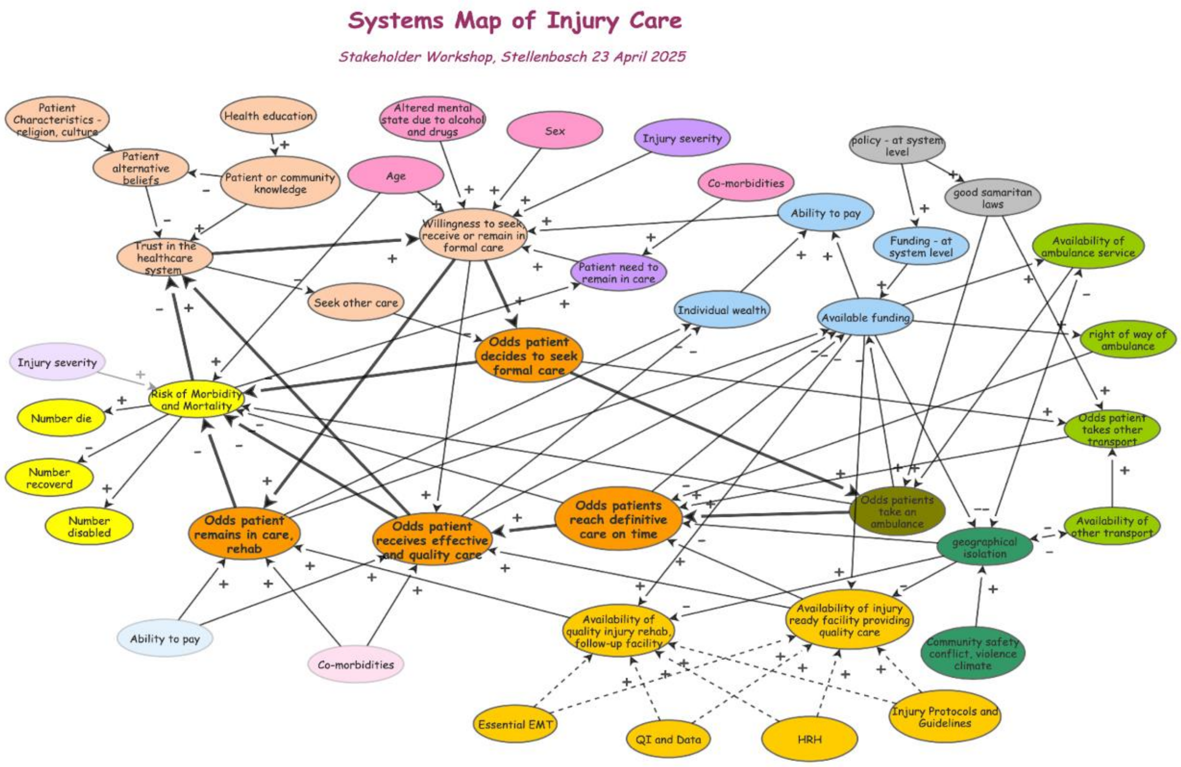

Supplementary Material Figure 2: Map shown to expert panel. Ghost variables are used to avoid connections crossing multiple variables and are show in the same colour as the parent variable, but paler. Bright pink are person-related factors; grey, related to policy and funding; blue, related to ability to pay; light pink, related to beliefs and knowledge; dark green, related to geographical isolation; light green, related to transport; purple, injury-related; light orange, health service related; dark orange, chance or odds of transitioning through the injury care pathway; and yellow, outcomes. HRH is human resources for health, QI is quality improvement, EMT is essential medicines and technologies.

Supplementary Material 6: Expert workshop outputs

Supplementary Material 6 Table 2: Final consensus factors included in the conceptual model

Factors on map presented to workshop	Definitions of factors
Intrinsic characteristics	Individual's, family, or community language, religious or cultural beliefs
Non-formal healthcare beliefs	Individual, family, or community beliefs about non-formal healthcare
Health education	Availability/receipt of health education
Patient or community knowledge	Individual, family, or community health/healthcare knowledge
Perception of health system	Individual, family, or community perception of the health system, including based on past experience
Trust in healthcare system	Patient, family, or community trust in the healthcare system
Willingness to seek, receive or remain in formal care	Willingness of the patient to seek or remain in formal healthcare
Seek non-formal care	Patients choose to seek non-formal health care, ie indigenous medicine
Sex	Patient's sex/gender-based decision making
Age	Patient's age/age-based decision making
Comorbidities	Patient's comorbidities, including acute and chronic mental health conditions (including altered mental state/alcohol/drugs)
Injury severity/type/mechanism	Severity, type, or mechanism of the initial injury
Need to remain in care	Clinical need to remain in care
Injury policy	National or subnational policy affecting injury care
Good Samaritan laws	Availability of laws protecting first responders
Injury related funding	Funding earmarked for injury care services
Ability to pay	Ability of the injured person to pay the direct and indirect costs of health care for injuries (including prehospital, hospital, and rehab)
Available funding	A "pool" of funding available at a national or subnational level for injury care
Individual wealth	Wealth of the patient (or their family)
Availability of ambulance service	Availability of staffed and equipped ambulance services
Odds patients take an ambulance	The odds that the patient will take an ambulance
Right of way of ambulance	Laws to give ambulances the right of way over other traffic
Availability of other transport	Availability of other non-ambulance transport
Odds patient takes other transport	The odds that the patient takes non-ambulance transport to get to care (including walking)
Geographical isolation	The temporal proximity of the patient to healthcare
Community safety conflict, violence climate	Environmental factors which impact upon injury care pathway
Availability of facility providing quality injury care	Availability of a facility which can provide quality definitive care appro to the patient
Availability of quality injury rehab or follow-up facility	Availability of a rehab or ongoing care facility which can provide quality rehab or ongoing care to the patient
Referral pathways	Referral pathways for interfacility transfers and to identifying which facilities patients should be taken to
Odds patient decides to seek formal care	The odds that the patient will seek formal healthcare
Odds patients reach definitive care on time	The odds that the patient will reach definitive care on time
Odds patient receives effective and quality care	The odds that the patient will receive effective (quality) care
Odds patient remains in care, rehab	The odds that the patient will remain in rehabilitation/ongoing care until optimal recovery
Risk of morbidity and mortality	The risk of morbidity or mortality to the patient
Number die	The number of persons who die
Number disabled	The number of patients who are disabled
Number survived	The number of patients who survive
**Health service map factors**	
**Factors indicating a quality injury facility**	

Timely	Care is provided without delay
Equity	All injured persons receive quality care appropriate to their medical needs
Safe	The care/treatment/environment are safe
Patient centred	The patient is treated with respect and dignity
Effective	Care provided is known to be effective to address the patient's condition
Integrated	Interfacility referral pathways are present and followed
Injury Protocols and Guidelines	Availability of injury protocols and guidelines at the facility level
QI and data	Availability of data and QI (quality improvement) at the facility level
Injury team	Team members needed to acutely manage injured patients are available, trained, and credentialed)
Essential MET	Availability of essential medicines, equipment, and technologies at the facility level
Injury protocols and guidelines	Availability of injury protocols and guidelines to guide management of the patient at that facility
Human capital	Positive work ethic
Leadership and governance	Hospital leadership and governance to implement injury care decisions
Discrimination	Discrimination of the injured person because of their intrinsic or extrinsic features (e.g: religion or colour)

Supplementary Material table 2. Factors included in the map agreed after the workshop, and their definitions. Implicit are the communication and relational aspects required for relevant factors.

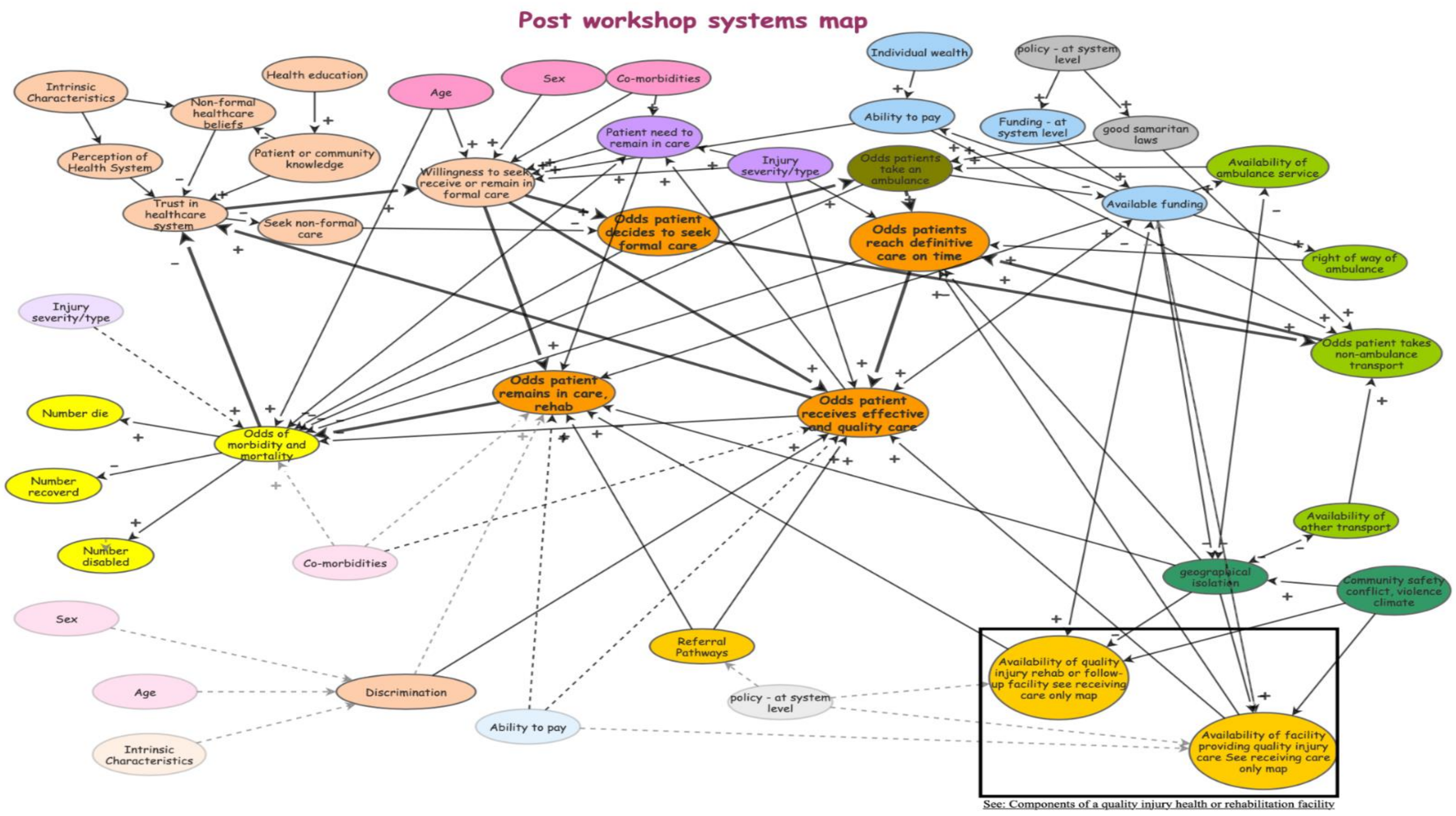

Supplementary Material Figure 3a. Ghost variables are used to avoid connections crossing multiple variables and are shown in paler shade of the same colour as the parent variable. Variables in bright pink are patient related factors, in light pink are related to the patient beliefs or knowledge, in purple are related to injury, in blue are related to wealth and costs, in grey are related to policy and funding, in green are related to transport and access, in orange are related to injury health facilities, in dark orange are related to odds of transitioning through the health services, and in yellow are related to patient outcomes.

Supplementary Material figure 3b: Post workshop health services map of the connections between factors necessary for quality acute and rehab care.

(see insert in Supplementary Material Figure 3a).

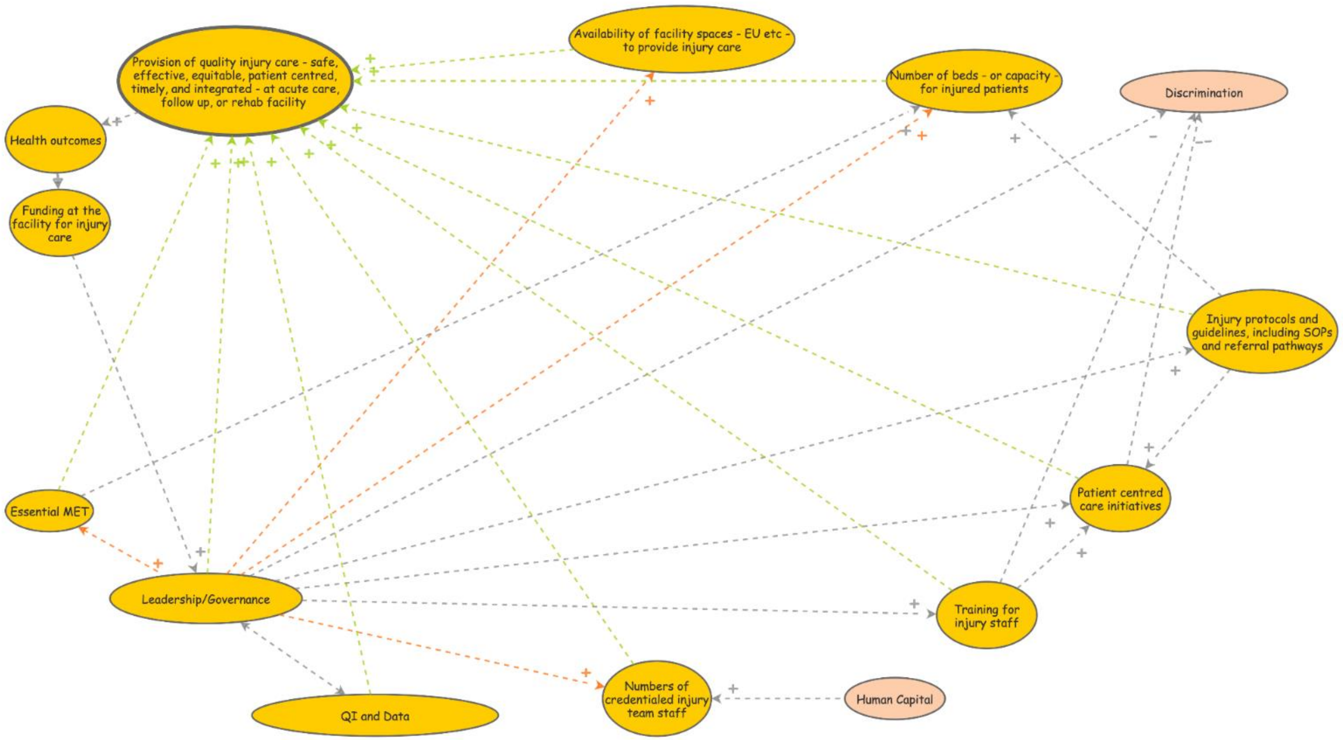

Supplementary Material 3b. Provision of quality injury care is considered according to IoM domains. Factors in orange are those which result in the care provision being quality. Factors in pink do not form causal loops as they have only arrows entering or leaving them. Green arrows indicate factors which directly affect provision of quality injury care. Grey arrows connect between factors. Orange arrows emerge from leadership and governance to indicate that the combination of funding and leadership and governance is required for these connections.

Supplementary Material 7

Supplementary Material Table 3 shows individual factors involved in each causal loop

Loop Number	Loop Length	Loop Theme	List of variables included in each feedback loop					
1	3	Patient Behaviour Loop	Patients trust in healthcare system	# Injured seeking non-formal care				
2	4	Patient Behaviour Loop	Patients trust in healthcare system	Willingness to seek formal care	# Injured seeking care			
3	5	Patient Behaviour Loop	Patients trust in healthcare system	Willingness to remain in care	# Patients remaining in care/rehab	# Patients Receiving Higher Quality Rehab		
4	5	Patient Behaviour Loop	Patients trust in healthcare system	Willingness to remain in care	# Patients remaining in care/rehab	# Patients receiving Lower Quality Rehab		
5	5	Patient Behaviour Loop	Patients trust in healthcare system	Willingness to receive care	# Patients in Definitive Care	# Patients Receiving Higher Quality Injury care		
6	5	Patient Behaviour Loop	Patients trust in healthcare system	Willingness to receive care	# Patients in Definitive Care	# Patients receiving Lower Quality Injury Care		
7	5	Patient Behaviour Loop	Patients trust in healthcare system	# Injured seeking non-formal care	Willingness to seek formal care	# Injured seeking care		
8	5	Patient Behaviour Loop	Patients trust in healthcare system	Willingness to seek formal care	# Injured seeking care	# Patients taking an ambulance		
9	6	Patient Behaviour Loop	Patients trust in healthcare system	Willingness to receive care	# Patients in Definitive Care	# Patients remaining in care/rehab	# Patients Receiving Higher Quality Rehab	
10	6	Patient Behaviour Loop	Patients trust in healthcare system	Willingness to receive care	# Patients in Definitive Care	# Patients remaining in care/rehab	# Patients receiving Lower Quality Rehab	
11	6	Patient Behaviour Loop	Patients trust in healthcare system	# Injured seeking non-formal care	Willingness to seek formal care	# Injured seeking care	# Patients taking an ambulance	
12	7	Transport & Access Loop	Total available workforce	GDP	System level funding	Available Funding for injury care	Availability of quality injury care	# Patients receiving Lower Quality Injury Care
13	7	Transport & Access Loop	Patients trust in healthcare system	Willingness to seek formal care	# Injured seeking care	# Patients taking an ambulance	# Patients in Definitive Care on Ambulance	# Patients in Definitive care Delayed

14	7	Transport & Access Loop	Total available workforce	GDP	Poverty	Community Conflict Violence etc	Availability of quality injury rehab or follow-up	# Patients Receiving Higher Quality Rehab													
15	7	Transport & Access Loop	Patients trust in healthcare system	Willingness to seek formal care	# Injured seeking care	# Patients taking an ambulance	# Patients in Definitive Care on Ambulance	# Patients in Definitive care on time													
16	7	Transport & Access Loop	Patients trust in healthcare system	Willingness to seek formal care	# Injured seeking care	# Patients taking other transport	# Patients in Definitive Care on other Transport	# Patients in Definitive care Delayed													
17	7	Transport & Access Loop	Total available workforce	GDP	System level funding	Available Funding for injury care	Availability of Ambulance Service	# Patients taking an ambulance													
18	7	Transport & Access Loop	Patients trust in healthcare system	Willingness to seek formal care	# Injured seeking care	# Patients taking other transport	# Patients in Definitive Care on other Transport	# Patients in Definitive care on time													
19	7	Transport & Access Loop	Total available workforce	GDP	Poverty	Community Conflict Violence etc	Availability of quality injury care	# Patients receiving Lower Quality Injury Care													
20	7	Transport & Access Loop	Total available workforce	GDP	System level funding	Available Funding for injury care	Availability of quality injury rehab or follow-up	# Patients receiving Lower Quality Rehab													
21	7	Transport & Access Loop	Total available workforce	GDP	Poverty	Community Conflict Violence etc	Availability of quality injury care	# Patients Receiving Higher Quality Injury care													
22	7	Transport & Access Loop	Total available workforce	GDP	Poverty	Community Conflict Violence etc	Availability of quality injury rehab or follow-up	# Patients receiving Lower Quality Rehab													
23	7	Transport & Access Loop	Total available workforce	GDP	System level funding	Available Funding for injury care	Availability of quality injury care	# Patients Receiving Higher Quality Injury care													
24	7	Transport & Access Loop	Total available workforce	GDP	System level funding	Available Funding for injury care	Availability of quality injury rehab or follow-up	# Patients Receiving Higher Quality Rehab													
25	8	Transport & Access Loop	Total available workforce	GDP	System level funding	Available Funding for injury care	Availability of quality injury care	Discrimination	# Patients Receiving Higher Quality Rehab												
26	8	Transport & Access Loop	Total available workforce	GDP	System level funding	Available Funding for injury care	Availability of quality injury rehab or follow-up	Discrimination	# Patients receiving Lower Quality Rehab												
27	8	Transport & Access Loop	Total available workforce	GDP	Poverty	Community Conflict Violence etc	Availability of quality injury rehab or follow-up	Discrimination	# Patients receiving Lower Quality Injury Care												
28	8	Transport & Access Loop	Patients trust in	Willingness to seek	# Injured seeking care	# Patients taking an	# Patients in Definitive	# Patients in Definitive Care	# Patients receiving Lower												

			healthcare system	formal care		ambulance	Care on Ambulance		Quality Injury Care												
29	8	Transport & Access Loop	Total available workforce	GDP	Poverty	Individual wealth	Ability to pay	Willingness to seek formal care	# Injured seeking care												
30	8	Transport & Access Loop	Patients trust in healthcare system	Willingness to receive care	# Patients in Definitive Care	# Patients receiving Lower Quality Injury Care	Willingness to remain in care	# Patients remaining in care/rehab	# Patients Receiving Higher Quality Rehab												
31	8	Transport & Access Loop	Total available workforce	GDP	System level funding	Available Funding for injury care	Availability of quality injury care	Discrimination	# Patients Receiving Higher Quality Injury care												
32	8	Transport & Access Loop	Patients trust in healthcare system	Willingness to seek formal care	# Injured seeking care	# Patients taking other transport	# Patients in Definitive Care on other Transport	# Patients in Definitive Care	# Patients receiving Lower Quality Injury Care												
33	8	Transport & Access Loop	Total available workforce	GDP	Poverty	Community Conflict Violence etc	Availability of quality injury care	Discrimination	# Patients Receiving Higher Quality Injury care												
34	8	Transport & Access Loop	Patients trust in healthcare system	Willingness to receive care	# Patients in Definitive Care	# Patients Receiving Higher Quality Injury care	Willingness to remain in care	# Patients remaining in care/rehab	# Patients Receiving Higher Quality Rehab												
35	8	Transport & Access Loop	Total available workforce	GDP	Poverty	Community Conflict Violence etc	Availability of quality injury rehab or follow-up	Discrimination	# Patients Receiving Higher Quality Rehab												
36	8	Transport & Access Loop	Total available workforce	GDP	System level funding	Available Funding for injury care	Availability of quality injury rehab or follow-up	Discrimination	# Patients receiving Lower Quality Injury Care												
37	8	Transport & Access Loop	Patients trust in healthcare system	Willingness to receive care	# Patients in Definitive Care	# Patients receiving Lower Quality Injury Care	Willingness to remain in care	# Patients remaining in care/rehab	# Patients receiving Lower Quality Rehab												
38	8	Transport & Access Loop	Total available workforce	GDP	System level funding	Available Funding for injury care	Availability of quality injury care	Discrimination	# Patients receiving Lower Quality Injury Care												
39	8	Transport & Access Loop	Patients trust in healthcare system	Willingness to seek formal care	# Injured seeking care	# Patients taking an ambulance	# Patients in Definitive Care on Ambulance	# Patients in Definitive Care	# Patients Receiving Higher Quality Injury care												
40	8	Transport & Access Loop	Patients trust in healthcare system	Willingness to seek formal care	# Injured seeking care	# Patients taking other transport	# Patients in Definitive Care on other Transport	# Patients in Definitive Care	# Patients Receiving Higher Quality Injury care												
41	8	Transport & Access Loop	Total available workforce	GDP	Poverty	Community Conflict Violence etc	Geographical accessibility	Availability of Ambulance Service	# Patients taking an ambulance												
42	8	Transport & Access Loop	Patients trust in	# Injured seeking non-	Willingness to seek	# Injured seeking care	# Patients taking an ambulance	# Patients in Definitive	# Patients in Definitive												

			healthcare system	formal care	formal care			Care on Ambulance	care on time												
43	8	Transport & Access Loop	Total available workforce	GDP	System level funding	Available Funding for injury care	Availability of quality injury care	Discrimination	# Patients receiving Lower Quality Rehab												
44	8	Transport & Access Loop	Total available workforce	GDP	Poverty	Community Conflict Violence etc	Availability of quality injury rehab or follow-up	Discrimination	# Patients receiving Lower Quality Rehab												
45	8	Transport & Access Loop	Patients trust in healthcare system	# Injured seeking non-formal care	Willingness to seek formal care	# Injured seeking care	# Patients taking other transport	# Patients in Definitive Care on other Transport	# Patients in Definitive care on time												
46	8	Transport & Access Loop	Total available workforce	GDP	System level funding	Available Funding for injury care	Availability of quality injury rehab or follow-up	Discrimination	# Patients Receiving Higher Quality Rehab												
47	8	Transport & Access Loop	Total available workforce	GDP	Poverty	Community Conflict Violence etc	Availability of quality injury rehab or follow-up	Discrimination	# Patients Receiving Higher Quality Injury care												
48	8	Transport & Access Loop	Total available workforce	GDP	Poverty	Community Conflict Violence etc	Availability of quality injury care	Discrimination	# Patients Receiving Higher Quality Rehab												
49	8	Transport & Access Loop	Patients trust in healthcare system	# Injured seeking non-formal care	Willingness to seek formal care	# Injured seeking care	# Patients taking an ambulance	# Patients in Definitive Care on Ambulance	# Patients in Definitive care Delayed												
50	8	Transport & Access Loop	Patients trust in healthcare system	# Injured seeking non-formal care	Willingness to seek formal care	# Injured seeking care	# Patients taking other transport	# Patients in Definitive Care on other Transport	# Patients in Definitive care Delayed												
51	8	Transport & Access Loop	Patients trust in healthcare system	Willingness to receive care	# Patients in Definitive Care	# Patients Receiving Higher Quality Injury care	Willingness to remain in care	# Patients remaining in care/rehab	# Patients receiving Lower Quality Rehab												
52	8	Transport & Access Loop	Total available workforce	GDP	Poverty	Community Conflict Violence etc	Availability of quality injury care	Discrimination	# Patients receiving Lower Quality Rehab												
53	8	Transport & Access Loop	Total available workforce	GDP	System level funding	Available Funding for injury care	Availability of quality injury rehab or follow-up	Discrimination	# Patients Receiving Higher Quality Injury care												
54	8	Transport & Access Loop	Total available workforce	GDP	Poverty	Community Conflict Violence etc	Availability of quality injury care	Discrimination	# Patients receiving Lower Quality Injury Care												
55	9	Transport & Access Loop	Patients trust in healthcare system	Willingness to seek formal care	# Injured seeking care	# Patients taking other transport	# Patients in Definitive Care on other Transport	# Patients in Definitive Care	# Patients remaining in care/rehab	# Patients receiving Lower Quality Rehab											
56	9	Transport & Access Loop	Patients trust in healthcare system	# Injured seeking non-	Willingness to seek formal care	# Injured seeking care	# Patients taking other transport	# Patients in Definitive Care on	# Patients in Definitive Care	# Patients receiving Lower Quality											

				formal care				other Transport		Injury Care											
57	9	Transport & Access Loop	Total available workforce	GDP	System level funding	Available Funding for injury care	Universal health insurance	Ability to pay	Willingness to seek formal care	# Injured seeking care											
58	9	Transport & Access Loop	Total available workforce	GDP	Poverty	Individual wealth	Availability of other transport	# Patients taking other transport	# Patients in Definitive Care on other Transport	# Patients in Definitive care Delayed											
59	9	Transport & Access Loop	Total available workforce	GDP	Poverty	Individual wealth	Ability to pay	Willingness to seek formal care	# Injured seeking care	# Patients taking an ambulance											
60	9	Transport & Access Loop	Patients trust in healthcare system	Willingness to seek formal care	# Injured seeking care	# Patients taking other transport	# Patients in Definitive Care on other Transport	# Patients in Definitive Care	# Patients remaining in care/rehab	# Patients Receiving Higher Quality Rehab											
61	9	Transport & Access Loop	Total available workforce	GDP	Poverty	Individual wealth	Availability of other transport	Willingness to remain in care	# Patients remaining in care/rehab	# Patients receiving Lower Quality Rehab											
62	9	Transport & Access Loop	Patients trust in healthcare system	# Injured seeking non-formal care	Willingness to seek formal care	# Injured seeking care	# Patients taking an ambulance	# Patients in Definitive Care on Ambulance	# Patients in Definitive Care	# Patients receiving Lower Quality Injury Care											
63	9	Transport & Access Loop	Total available workforce	GDP	Poverty	Individual wealth	Availability of other transport	Geographical accessibility	Availability of Ambulance Service	# Patients taking an ambulance											
64	9	Transport & Access Loop	Total available workforce	GDP	Poverty	Individual wealth	Ability to pay	Willingness to remain in care	# Patients remaining in care/rehab	# Patients Receiving Higher Quality Rehab											
65	9	Transport & Access Loop	Patients trust in healthcare system	# Injured seeking non-formal care	Willingness to seek formal care	# Injured seeking care	# Patients taking other transport	# Patients in Definitive Care on other Transport	# Patients in Definitive Care	# Patients Receiving Higher Quality Injury care											
66	9	Transport & Access Loop	Total available workforce	GDP	Poverty	Individual wealth	Ability to pay	Willingness to receive care	# Patients in Definitive Care	# Patients receiving Lower Quality Injury Care											
67	9	Transport & Access Loop	Total available workforce	GDP	System level funding	Available Funding for injury care	Availability of Ambulance Service	# Patients taking an ambulance	# Patients in Definitive Care on Ambulance	# Patients in Definitive care Delayed											
68	9	Transport & Access Loop	Total available workforce	GDP	System level funding	Available Funding for injury care	Availability of Ambulance Service	# Patients taking an ambulance	# Patients in Definitive Care on Ambulance	# Patients in Definitive care on time											
69	9	Transport & Access Loop	Total available workforce	GDP	Poverty	Individual wealth	Availability of other transport	# Patients taking other transport	# Patients in Definitive Care on other Transport	# Patients in Definitive care on time											
70	9	Transport & Access Loop	Patients trust in healthcare system	# Injured seeking non-formal care	Willingness to seek formal care	# Injured seeking care	# Patients taking an ambulance	# Patients in Definitive Care on Ambulance	# Patients in Definitive Care	# Patients Receiving Higher Quality Injury care											

71	9	Transport & Access Loop	Total available workforce	GDP	Poverty	Individual wealth	Ability to pay	Willingness to receive care	# Patients in Definitive Care	# Patients Receiving Higher Quality Injury care											
72	9	Transport & Access Loop	Total available workforce	GDP	Poverty	Individual wealth	Ability to pay	Willingness to remain in care	# Patients remaining in care/reha b	# Patients receiving Lower Quality Rehab											
73	9	Transport & Access Loop	Total available workforce	GDP	Poverty	Individual wealth	Availability of other transport	Willingness to remain in care	# Patients remaining in care/reha b	# Patients Receiving Higher Quality Rehab											
74	9	Transport & Access Loop	Patients trust in healthcar e system	Willingnes s to seek formal care	# Injured seeking care	# Patients taking an ambulanc e	# Patients in Definitive Care on Ambulance	# Patients in Definitive Care	# Patients remaining in care/reha b	# Patients receiving Lower Quality Rehab											
75	9	Transport & Access Loop	Patients trust in healthcar e system	Willingnes s to seek formal care	# Injured seeking care	# Patients taking an ambulanc e	# Patients in Definitive Care on Ambulance	# Patients in Definitive Care	# Patients remaining in care/reha b	# Patients Receiving Higher Quality Rehab											
76	10	Care Quality Loop	Total available workforce	GDP	System level funding	Available Funding for injury care	Availability of quality injury care	# Patients Receiving Higher Quality Injury care	Willingnes s to remain in care	# Patients remaining in care/reha b	# Patients Receiving Higher Quality Rehab										
77	10	Care Quality Loop	Total available workforce	GDP	System level funding	Available Funding for injury care	Availability of quality injury care	# Patients Receiving Higher Quality Injury care	Willingnes s to remain in care	# Patients remaining in care/reha b	# Patients receiving Lower Quality Rehab										
78	10	Care Quality Loop	Patients trust in healthcar e system	# Injured seeking non-formal care	Willingnes s to seek formal care	# Injured seeking care	# Patients taking other transport	# Patients in Definitive Care on other Transport	# Patients in Definitive Care	# Patients remaining in care/reha b	# Patients receiving Lower Quality Rehab										
79	10	Care Quality Loop	Total available workforce	GDP	Poverty	Individual wealth	Ability to pay	Willingness to receive care	# Patients in Definitive Care	# Patients remaining in care/reha b	# Patients receiving Lower Quality Rehab										
80	10	Care Quality Loop	Total available workforce	GDP	System level funding	Available Funding for injury care	Availability of quality injury care	# Patients receiving Lower Quality Injury Care	Patients Perceptio n of Health System	Patients trust in healthcare system	# Injured seeking non-formal care										
81	10	Care Quality Loop	Total available workforce	GDP	Poverty	Communit y Conflict Violence etc	Geographic al accessibility	Availability of other transport	# Patients taking other transport	# Patients in Definitive Care on other Transport	# Patients in Definitive care Delayed										
82	10	Care Quality Loop	Total available workforce	GDP	System level funding	Available Funding for injury care	Availability of quality injury care	Discriminatio n	Willingnes s to remain in care	# Patients remaining in care/reha b	# Patients receiving Lower Quality Rehab										
83	10	Care Quality Loop	Total available workforce	GDP	System level funding	Available Funding for injury care	Availability of quality injury care	# Patients receiving Lower Quality Injury Care	Willingnes s to remain in care	# Patients remaining in care/reha b	# Patients receiving Lower Quality Rehab										
84	10	Care Quality Loop	Patients trust in healthcar e system	# Injured seeking non-formal care	Willingnes s to seek formal care	# Injured seeking care	# Patients taking other transport	# Patients in Definitive Care on other Transport	# Patients in Definitive Care	# Patients remaining in care/reha b	# Patients Receiving Higher Quality Rehab										
85	10	Care Quality Loop	Total available workforce	GDP	Poverty	Communit y Conflict	Availability	# Patients receiving	Patients Perceptio	Patients trust in	# Injured seeking										

						Violence etc	of quality injury care	Lower Quality Injury Care	n of Health System	healthcare system	non- formal care										
86	10	Care Quality Loop	Total available workforce	GDP	System level funding	Available Funding for injury care	Availability of Ambulance Service	# Patients taking an ambulance	# Patients in Definitive Care on Ambulanc e	# Patients in Definitive Care	# Patients receiving Lower Quality Injury Care										
87	10	Care Quality Loop	Total available workforce	GDP	Poverty	Communit y Conflict Violence etc	Geographic al accessibility	Availability of other transport	Willingnes s to remain in care	# Patients remaining in care/reha b	# Patients Receiving Higher Quality Rehab										
88	10	Care Quality Loop	Total available workforce	GDP	System level funding	Available Funding for injury care	Availability of quality injury care	Discriminatio n	Willingnes s to remain in care	# Patients remaining in care/reha b	# Patients Receiving Higher Quality Rehab										
89	10	Care Quality Loop	Total available workforce	GDP	System level funding	Available Funding for injury care	Universal health insurance	Ability to pay	Willingnes s to remain in care	# Patients remaining in care/reha b	# Patients Receiving Higher Quality Rehab										
90	10	Care Quality Loop	Total available workforce	GDP	Poverty	Communit y Conflict Violence etc	Geographic al accessibility	Availability of other transport	# Patients taking other transport	# Patients in Definitive Care on other Transport	# Patients in Definitive care on time										
91	10	Care Quality Loop	Total available workforce	GDP	Poverty	Individual wealth	Ability to pay	Willingness to receive care	# Patients in Definitive Care	# Patients remaining in care/reha b	# Patients Receiving Higher Quality Rehab										
92	10	Care Quality Loop	Total available workforce	GDP	System level funding	Available Funding for injury care	Availability of quality injury care	# Patients receiving Lower Quality Injury Care	Willingnes s to remain in care	# Patients remaining in care/reha b	# Patients Receiving Higher Quality Rehab										
93	10	Care Quality Loop	Total available workforce	GDP	Poverty	Communit y Conflict Violence etc	Availability of quality injury care	# Patients receiving Lower Quality Injury Care	Willingnes s to remain in care	# Patients remaining in care/reha b	# Patients Receiving Higher Quality Rehab										
94	10	Care Quality Loop	Total available workforce	GDP	Poverty	Communit y Conflict Violence etc	Availability of quality injury care	# Patients Receiving Higher Quality Injury care	Willingnes s to remain in care	# Patients remaining in care/reha b	# Patients Receiving Higher Quality Rehab										
95	10	Care Quality Loop	Total available workforce	GDP	System level funding	Available Funding for injury care	Availability of quality injury care	# Patients Receiving Higher Quality Injury care	Patients Perceptio n of Health System	Patients trust in healthcare system	# Injured seeking non- formal care										
96	10	Care Quality Loop	Total available workforce	GDP	Poverty	Communit y Conflict Violence etc	Geographic al accessibility	Availability of other transport	Willingnes s to remain in care	# Patients remaining in care/reha b	# Patients receiving Lower Quality Rehab										
97	10	Care Quality Loop	Total available workforce	GDP	Poverty	Individual wealth	Availability of other transport	# Patients taking other transport	# Patients in Definitive Care on other Transport	# Patients in Definitive Care	# Patients receiving Lower Quality Injury Care										
98	10	Care Quality Loop	Total available workforce	GDP	Poverty	Communit y Conflict Violence etc	Geographic al accessibility	Availability of Ambulance Service	# Patients taking an ambulanc e	# Patients in Definitive Care on Ambulanc e	# Patients in Definitive care Delayed										
99	10	Care Quality Loop	Total available workforce	GDP	System level funding	Available Funding	Universal health insurance	Ability to pay	Willingnes s to	# Patients in Definitive Care	# Patients Receiving Higher										

						for injury care			receive care		Quality Injury care										
100	10	Care Quality Loop	Total available workforce	GDP	Poverty	Community Conflict Violence etc	Availability of quality injury rehab or follow-up	Discrimination	Willingness to remain in care	# Patients remaining in care/rehab	# Patients Receiving Higher Quality Rehab										
101	10	Care Quality Loop	Total available workforce	GDP	System level funding	Available Funding for injury care	Universal health insurance	Ability to pay	Willingness to receive care	# Patients in Definitive Care	# Patients receiving Lower Quality Injury Care										
102	10	Care Quality Loop	Total available workforce	GDP	Poverty	Community Conflict Violence etc	Geographical accessibility	Availability of Ambulance Service	# Patients taking an ambulance	# Patients in Definitive Care on Ambulance	# Patients in Definitive care on time										
103	10	Care Quality Loop	Total available workforce	GDP	System level funding	Available Funding for injury care	Universal health insurance	Ability to pay	Willingness to seek formal care	# Injured seeking care	# Patients taking an ambulance										
104	10	Care Quality Loop	Total available workforce	GDP	Poverty	Community Conflict Violence etc	Availability of quality injury care	Discrimination	Willingness to remain in care	# Patients remaining in care/rehab	# Patients receiving Lower Quality Rehab										
105	10	Care Quality Loop	Patients trust in healthcare system	# Injured seeking non-formal care	Willingness to seek formal care	# Injured seeking care	# Patients taking an ambulance	# Patients in Definitive Care on Ambulance	# Patients in Definitive Care	# Patients remaining in care/rehab	# Patients receiving Lower Quality Rehab										
106	10	Care Quality Loop	Total available workforce	GDP	System level funding	Available Funding for injury care	Universal health insurance	Ability to pay	Willingness to remain in care	# Patients remaining in care/rehab	# Patients receiving Lower Quality Rehab										
107	10	Care Quality Loop	Patients trust in healthcare system	# Injured seeking non-formal care	Willingness to seek formal care	# Injured seeking care	# Patients taking an ambulance	# Patients in Definitive Care on Ambulance	# Patients in Definitive Care	# Patients remaining in care/rehab	# Patients Receiving Higher Quality Rehab										
108	10	Care Quality Loop	Total available workforce	GDP	Poverty	Community Conflict Violence etc	Availability of quality injury rehab or follow-up	Discrimination	Willingness to remain in care	# Patients remaining in care/rehab	# Patients receiving Lower Quality Rehab										
109	10	Care Quality Loop	Total available workforce	GDP	Poverty	Community Conflict Violence etc	Availability of quality injury care	Discrimination	Willingness to remain in care	# Patients remaining in care/rehab	# Patients Receiving Higher Quality Rehab										
110	10	Care Quality Loop	Total available workforce	GDP	System level funding	Available Funding for injury care	Availability of quality injury rehab or follow-up	Discrimination	Willingness to remain in care	# Patients remaining in care/rehab	# Patients Receiving Higher Quality Rehab										
111	10	Care Quality Loop	Total available workforce	GDP	Poverty	Community Conflict Violence etc	Availability of quality injury care	# Patients Receiving Higher Quality Injury care	Patients Perception of Health System	Patients trust in healthcare system	# Injured seeking non-formal care										
112	10	Care Quality Loop	Total available workforce	GDP	System level funding	Available Funding for injury care	Availability of Ambulance Service	# Patients taking an ambulance	# Patients in Definitive Care on Ambulance	# Patients in Definitive Care	# Patients Receiving Higher Quality Injury care										
113	10	Care Quality Loop	Total available workforce	GDP	System level funding	Available Funding for injury care	Availability of quality injury rehab or follow-up	Discrimination	Willingness to remain in care	# Patients remaining in care/rehab	# Patients receiving Lower Quality Rehab										

114	10	Care Quality Loop	Total available workforce	GDP	Poverty	Individual wealth	Availability of other transport	# Patients taking other transport	# Patients in Definitive Care on other Transport	# Patients in Definitive Care	# Patients Receiving Higher Quality Injury care										
115	10	Care Quality Loop	Total available workforce	GDP	Poverty	Communit y Conflict Violence etc	Availability of quality injury care	# Patients receiving Lower Quality Injury Care	Willingnes s to remain in care	# Patients remaining in care/reha b	# Patients receiving Lower Quality Rehab										
116	10	Care Quality Loop	Total available workforce	GDP	Poverty	Communit y Conflict Violence etc	Availability of quality injury care	# Patients Receiving Higher Quality Injury care	Willingnes s to remain in care	# Patients remaining in care/reha b	# Patients receiving Lower Quality Rehab										
117	11	Care Quality Loop	Patients trust in healthcar e system	Willingnes s to seek formal care	# Injured seeking care	# Patients taking other transport	# Patients in Definitive Care on other Transport	# Patients in Definitive Care	# Patients receiving Lower Quality Injury Care	Willingnes s to remain in care	# Patients remaining in care/reha b	# Patients Receiving Higher Quality Rehab									
118	11	Care Quality Loop	Patients trust in healthcar e system	Willingnes s to seek formal care	# Injured seeking care	# Patients taking other transport	# Patients in Definitive Care on other Transport	# Patients in Definitive Care	# Patients receiving Lower Quality Injury Care	Willingnes s to remain in care	# Patients remaining in care/reha b	# Patients receiving Lower Quality Rehab									
119	11	Care Quality Loop	Total available workforce	GDP	Poverty	Individual wealth	Ability to pay	Willingness to seek formal care	# Injured seeking care	# Patients taking other transport	# Patients in Definitive Care on other Transport	# Patients in Definitive care Delayed									
120	11	Care Quality Loop	Total available workforce	GDP	Poverty	Individual wealth	Ability to pay	Willingness to seek formal care	# Injured seeking care	# Patients taking an ambulanc e	# Patients in Definitive Care on Ambulanc e	# Patients in Definitive care Delayed									
121	11	Care Quality Loop	Total available workforce	GDP	Poverty	Individual wealth	Ability to pay	Willingness to seek formal care	# Injured seeking care	# Patients taking an ambulanc e	# Patients in Definitive Care on Ambulanc e	# Patients in Definitive care on time									
122	11	Care Quality Loop	Total available workforce	GDP	System level funding	Available Funding for injury care	Universal health insurance	Ability to pay	Willingnes s to receive care	# Patients in Definitive Care	# Patients remaining in care/reha b	# Patients receiving Lower Quality Rehab									
123	11	Care Quality Loop	Total available workforce	GDP	Poverty	Communit y Conflict Violence etc	Availability of quality injury rehab or follow-up	Discriminatio n	# Patients Receiving Higher Quality Injury care	Willingnes s to remain in care	# Patients remaining in care/reha b	# Patients Receiving Higher Quality Rehab									
124	11	Care Quality Loop	Total available workforce	GDP	System level funding	Available Funding for injury care	Availability of quality injury care	Discriminatio n	# Patients receiving Lower Quality Injury Care	Patients Perceptio n of Health System	Patients trust in healthcare system	# Injured seeking non-formal care									
125	11	Care Quality Loop	Total available workforce	GDP	System level funding	Available Funding for injury care	Availability of quality injury rehab or follow-up	Discriminatio n	# Patients Receiving Higher Quality Injury care	Willingnes s to remain in care	# Patients remaining in care/reha b	# Patients Receiving Higher Quality Rehab									
126	11	Care Quality Loop	Total available workforce	GDP	System level funding	Available Funding for injury care	Availability of quality injury rehab or follow-up	Discriminatio n	# Patients receiving Lower Quality Injury Care	Willingnes s to remain in care	# Patients remaining in care/reha b	# Patients Receiving Higher Quality Rehab									
127	11	Care Quality Loop	Patients trust in healthcar e system	Willingnes s to seek formal care	# Injured seeking care	# Patients taking other transport	# Patients in Definitive Care on	# Patients in Definitive Care	# Patients Receiving Higher	Willingnes s to remain in care	# Patients remaining in	# Patients Receiving Higher									

							other Transport		Quality Injury care		care/reha b	Quality Rehab									
128	11	Care Quality Loop	Total available workforce	GDP	System level funding	Available Funding for injury care	Universal health insurance	Ability to pay	Willingnes s to receive care	# Patients in Definitive Care	# Patients remaining in care/reha b	# Patients Receiving Higher Quality Rehab									
129	11	Care Quality Loop	Total available workforce	GDP	Poverty	Communit y Conflict Violence etc	Availability of quality injury rehab or follow-up	Discriminatio n	# Patients Receiving Higher Quality Injury care	Willingnes s to remain in care	# Patients remaining in care/reha b	# Patients receiving Lower Quality Rehab									
130	11	Care Quality Loop	Total available workforce	GDP	System level funding	Available Funding for injury care	Availability of Ambulance Service	Geographica l accessibility	Availability of other transport	# Patients taking other transport	# Patients in Definitive Care on other Transport	# Patients in Definitive care on time									
131	11	Care Quality Loop	Total available workforce	GDP	Poverty	Communit y Conflict Violence etc	Availability of quality injury rehab or follow-up	Discriminatio n	# Patients receiving Lower Quality Injury Care	Willingnes s to remain in care	# Patients remaining in care/reha b	# Patients Receiving Higher Quality Rehab									
132	11	Care Quality Loop	Total available workforce	GDP	System level funding	Available Funding for injury care	Availability of Ambulance Service	# Patients taking an ambulance	# Patients in Definitive Care on Ambulanc e	# Patients in Definitive Care	# Patients remaining in care/reha b	# Patients receiving Lower Quality Rehab									
133	11	Care Quality Loop	Total available workforce	GDP	Poverty	Communit y Conflict Violence etc	Availability of quality injury rehab or follow-up	Discriminatio n	# Patients receiving Lower Quality Injury Care	Patients Perceptio n of Health System	Patients trust in healthcare system	# Injured seeking non-formal care									
134	11	Care Quality Loop	Patients trust in healthcar e system	Willingnes s to seek formal care	# Injured seeking care	# Patients taking other transport	# Patients in Definitive Care on other Transport	# Patients in Definitive Care	# Patients Receiving Higher Quality Injury care	Willingnes s to remain in care	# Patients remaining in care/reha b	# Patients receiving Lower Quality Rehab									
135	11	Care Quality Loop	Total available workforce	GDP	System level funding	Available Funding for injury care	Availability of quality injury care	Discriminatio n	# Patients Receiving Higher Quality Injury care	Willingnes s to remain in care	# Patients remaining in care/reha b	# Patients Receiving Higher Quality Rehab									
136	11	Care Quality Loop	Total available workforce	GDP	Poverty	Communit y Conflict Violence etc	Availability of quality injury care	Discriminatio n	# Patients Receiving Higher Quality Injury care	Willingnes s to remain in care	# Patients remaining in care/reha b	# Patients Receiving Higher Quality Rehab									
137	11	Care Quality Loop	Total available workforce	GDP	Poverty	Communit y Conflict Violence etc	Availability of quality injury care	Discriminatio n	# Patients receiving Lower Quality Injury Care	Willingnes s to remain in care	# Patients remaining in care/reha b	# Patients Receiving Higher Quality Rehab									
138	11	Care Quality Loop	Total available workforce	GDP	Poverty	Individual wealth	Ability to pay	Willingness to seek formal care	# Injured seeking care	# Patients taking other transport	# Patients in Definitive Care on other Transport	# Patients in Definitive care on time									
139	11	Care Quality Loop	Patients trust in healthcar e system	Willingnes s to seek formal care	# Injured seeking care	# Patients taking an ambulanc e	# Patients in Definitive Care on Ambulance	# Patients in Definitive Care	# Patients Receiving Higher Quality Injury care	Willingnes s to remain in care	# Patients remaining in care/reha b	# Patients receiving Lower Quality Rehab									
140	11	Care Quality Loop	Total available workforce	GDP	System level funding	Available Funding for injury care	Availability of quality injury care	Discriminatio n	# Patients Receiving Higher Quality Injury care	Willingnes s to remain in care	# Patients remaining in care/reha b	# Patients receiving Lower Quality Rehab									
141	11	Care Quality Loop	Total available workforce	GDP	Poverty	Communit y Conflict	Geographic	Availability of	# Patients taking an	# Patients in Definitive	# Patients in	# Patients receiving									

						Violence etc	al accessibility	Ambulance Service	ambulanc e	Care on Ambulanc e	Definitive Care	Lower Quality Injury Care									
142	11	Care Quality Loop	Total available workforce	GDP	System level funding	Available Funding for injury care	Availability of Ambulance Service	Geographica l accessibility	Availability of other transport	Willingnes s to remain in care	# Patients remaining in care/reha b	# Patients Receiving Higher Quality Rehab									
143	11	Care Quality Loop	Total available workforce	GDP	System level funding	Available Funding for injury care	Availability of quality injury care	# Patients Receiving Higher Quality Injury care	Patients Perceptio n of Health System	Patients trust in healthcare system	Willingnes s to seek formal care	# Injured seeking care									
144	11	Care Quality Loop	Total available workforce	GDP	System level funding	Available Funding for injury care	Availability of quality injury care	Discriminatio n	# Patients receiving Lower Quality Injury Care	Willingnes s to remain in care	# Patients remaining in care/reha b	# Patients Receiving Higher Quality Rehab									
145	11	Care Quality Loop	Total available workforce	GDP	System level funding	Available Funding for injury care	Availability of Ambulance Service	Geographica l accessibility	Availability of other transport	# Patients taking other transport	# Patients in Definitive Care on other Transport	# Patients in Definitive care Delayed									
146	11	Care Quality Loop	Total available workforce	GDP	System level funding	Available Funding for injury care	Availability of quality injury care	# Patients receiving Lower Quality Injury Care	Patients Perceptio n of Health System	Patients trust in healthcare system	Willingnes s to seek formal care	# Injured seeking care									
147	11	Care Quality Loop	Total available workforce	GDP	Poverty	Communit y Conflict Violence etc	Geographic al accessibility	Availability of other transport	# Patients taking other transport	# Patients in Definitive Care on other Transport	# Patients in Definitive Care	# Patients receiving Lower Quality Injury Care									
148	11	Care Quality Loop	Total available workforce	GDP	System level funding	Available Funding for injury care	Availability of quality injury care	Discriminatio n	# Patients receiving Lower Quality Injury Care	Willingnes s to remain in care	# Patients remaining in care/reha b	# Patients receiving Lower Quality Rehab									
149	11	Care Quality Loop	Patients trust in healthcar e system	Willingnes s to seek formal care	# Injured seeking care	# Patients taking an ambulanc e	# Patients in Definitive Care on Ambulance	# Patients in Definitive Care	# Patients receiving Lower Quality Injury Care	Willingnes s to remain in care	# Patients remaining in care/reha b	# Patients Receiving Higher Quality Rehab									
150	11	Care Quality Loop	Total available workforce	GDP	System level funding	Available Funding for injury care	Availability of quality injury rehab or follow-up	Discriminatio n	# Patients Receiving Higher Quality Injury care	Willingnes s to remain in care	# Patients remaining in care/reha b	# Patients receiving Lower Quality Rehab									
151	11	Care Quality Loop	Total available workforce	GDP	Poverty	Communit y Conflict Violence etc	Availability of quality injury care	Discriminatio n	# Patients Receiving Higher Quality Injury care	Willingnes s to remain in care	# Patients remaining in care/reha b	# Patients receiving Lower Quality Rehab									
152	11	Care Quality Loop	Patients trust in healthcar e system	Willingnes s to seek formal care	# Injured seeking care	# Patients taking an ambulanc e	# Patients in Definitive Care on Ambulance	# Patients in Definitive Care	# Patients receiving Lower Quality Injury Care	Willingnes s to remain in care	# Patients remaining in care/reha b	# Patients receiving Lower Quality Rehab									
153	11	Care Quality Loop	Total available workforce	GDP	System level funding	Available Funding for injury care	Availability of quality injury rehab or follow-up	Discriminatio n	# Patients receiving Lower Quality Injury Care	Willingnes s to remain in care	# Patients remaining in care/reha b	# Patients receiving Lower Quality Rehab									
154	11	Care Quality Loop	Total available workforce	GDP	Poverty	Communit y Conflict Violence etc	Geographic al accessibility	Availability of Ambulance Service	# Patients taking an ambulanc e	# Patients in Definitive Care on	# Patients in Definitive Care	# Patients Receiving Higher									

										Ambulance		Quality Injury care									
155	11	Care Quality Loop	Total available workforce	GDP	System level funding	Available Funding for injury care	Availability of Ambulance Service	Geographical accessibility	Availability of other transport	Willingness to remain in care	# Patients remaining in care/rehab	# Patients receiving Lower Quality Rehab									
156	11	Care Quality Loop	Patients trust in healthcare system	Willingness to seek formal care	# Injured seeking care	# Patients taking an ambulance	# Patients in Definitive Care on Ambulance	# Patients in Definitive Care	# Patients Receiving Higher Quality Injury care	Willingness to remain in care	# Patients remaining in care/rehab	# Patients Receiving Higher Quality Rehab									
157	11	Care Quality Loop	Total available workforce	GDP	Poverty	Community Conflict Violence etc	Availability of quality injury care	# Patients Receiving Higher Quality Injury care	Patients Perception of Health System	Patients trust in healthcare system	Willingness to seek formal care	# Injured seeking care									
158	11	Care Quality Loop	Total available workforce	GDP	Poverty	Individual wealth	Availability of other transport	Geographical accessibility	Availability of Ambulance Service	# Patients taking an ambulance	# Patients in Definitive Care on Ambulance	# Patients in Definitive care Delayed									
159	11	Care Quality Loop	Total available workforce	GDP	Poverty	Community Conflict Violence etc	Availability of quality injury rehab or follow-up	Discrimination	# Patients Receiving Higher Quality Injury care	Patients Perception of Health System	Patients trust in healthcare system	# Injured seeking non-formal care									
160	11	Care Quality Loop	Total available workforce	GDP	System level funding	Available Funding for injury care	Availability of quality injury rehab or follow-up	Discrimination	# Patients Receiving Higher Quality Injury care	Patients Perception of Health System	Patients trust in healthcare system	# Injured seeking non-formal care									
161	11	Care Quality Loop	Total available workforce	GDP	Poverty	Community Conflict Violence etc	Availability of quality injury care	Discrimination	# Patients receiving Lower Quality Injury Care	Willingness to remain in care	# Patients remaining in care/rehab	# Patients receiving Lower Quality Rehab									
162	11	Care Quality Loop	Total available workforce	GDP	Poverty	Community Conflict Violence etc	Availability of quality injury care	Discrimination	# Patients Receiving Higher Quality Injury care	Patients Perception of Health System	Patients trust in healthcare system	# Injured seeking non-formal care									
163	11	Care Quality Loop	Total available workforce	GDP	Poverty	Community Conflict Violence etc	Availability of quality injury care	# Patients receiving Lower Quality Injury Care	Patients Perception of Health System	Patients trust in healthcare system	Willingness to seek formal care	# Injured seeking care									
164	11	Care Quality Loop	Total available workforce	GDP	Poverty	Community Conflict Violence etc	Geographical accessibility	Availability of other transport	# Patients taking other transport	# Patients in Definitive Care on other Transport	# Patients in Definitive Care	# Patients Receiving Higher Quality Injury care									
165	11	Care Quality Loop	Total available workforce	GDP	Poverty	Individual wealth	Availability of other transport	Geographical accessibility	Availability of Ambulance Service	# Patients taking an ambulance	# Patients in Definitive Care on Ambulance	# Patients in Definitive care on time									
166	11	Care Quality Loop	Total available workforce	GDP	Poverty	Individual wealth	Availability of other transport	# Patients taking other transport	# Patients in Definitive Care on other Transport	# Patients in Definitive Care	# Patients remaining in care/rehab	# Patients receiving Lower Quality Rehab									
167	11	Care Quality Loop	Total available workforce	GDP	Poverty	Community Conflict Violence etc	Availability of quality injury care	Discrimination	# Patients receiving Lower Quality Injury Care	Patients Perception of Health System	Patients trust in healthcare system	# Injured seeking non-formal care									
168	11	Care Quality Loop	Total available workforce	GDP	System level funding	Available Funding	Availability of quality	Discrimination	# Patients receiving Lower	Patients Perception of	Patients trust in	# Injured seeking non-									

						for injury care	injury rehab or follow-up		Quality Injury Care	Health System	healthcare system	formal care									
169	11	Care Quality Loop	Total available workforce	GDP	System level funding	Available Funding for injury care	Availability of Ambulance Service	# Patients taking an ambulance	# Patients in Definitive Care on Ambulance	# Patients in Definitive Care	# Patients remaining in care/rehab	# Patients Receiving Higher Quality Rehab									
170	11	Care Quality Loop	Total available workforce	GDP	Poverty	Individual wealth	Availability of other transport	# Patients taking other transport	# Patients in Definitive Care on other Transport	# Patients in Definitive Care	# Patients remaining in care/rehab	# Patients Receiving Higher Quality Rehab									
171	11	Care Quality Loop	Total available workforce	GDP	Poverty	Community Conflict Violence etc	Availability of quality injury rehab or follow-up	Discrimination	# Patients receiving Lower Quality Injury Care	Willingness to remain in care	# Patients remaining in care/rehab	# Patients receiving Lower Quality Rehab									
172	11	Care Quality Loop	Total available workforce	GDP	System level funding	Available Funding for injury care	Availability of quality injury care	Discrimination	# Patients Receiving Higher Quality Injury care	Patients Perception of Health System	Patients trust in healthcare system	# Injured seeking non-formal care									
173	12	Care Quality Loop	Total available workforce	GDP	Poverty	Individual wealth	Ability to pay	Willingness to receive care	# Patients in Definitive Care	# Patients receiving Lower Quality Injury Care	Willingness to remain in care	# Patients remaining in care/rehab	# Patients Receiving Higher Quality Rehab								
174	12	Care Quality Loop	Total available workforce	GDP	System level funding	Available Funding for injury care	Availability of quality injury care	# Patients receiving Lower Quality Injury Care	Patients Perception of Health System	Patients trust in healthcare system	Willingness to seek formal care	# Injured seeking care	# Patients taking an ambulance								
175	12	Care Quality Loop	Total available workforce	GDP	Poverty	Individual wealth	Availability of other transport	Geographical accessibility	Availability of Ambulance Service	# Patients taking an ambulance	# Patients in Definitive Care on Ambulance	# Patients in Definitive Care	# Patients receiving Lower Quality Injury Care								
176	12	Care Quality Loop	Total available workforce	GDP	Poverty	Individual wealth	Ability to pay	Willingness to seek formal care	# Injured seeking care	# Patients taking other transport	# Patients in Definitive Care on other Transport	# Patients in Definitive Care	# Patients receiving Lower Quality Injury Care								
177	12	Care Quality Loop	Total available workforce	GDP	System level funding	Available Funding for injury care	Universal health insurance	Ability to pay	Willingness to seek formal care	# Injured seeking care	# Patients taking an ambulance	# Patients in Definitive Care on Ambulance	# Patients in Definitive care Delayed								
178	12	Care Quality Loop	Total available workforce	GDP	Poverty	Community Conflict Violence etc	Availability of quality injury care	# Patients receiving Lower Quality Injury Care	Patients Perception of Health System	Patients trust in healthcare system	Willingness to remain in care	# Patients remaining in care/rehab	# Patients receiving Lower Quality Rehab								
179	12	Care Quality Loop	Total available workforce	GDP	System level funding	Available Funding for injury care	Availability of quality injury care	# Patients Receiving Higher Quality Injury care	Patients Perception of Health System	Patients trust in healthcare system	Willingness to receive care	# Patients in Definitive Care	# Patients receiving Lower Quality Injury Care								
180	12	Care Quality Loop	Total available workforce	GDP	Poverty	Community Conflict Violence etc	Availability of quality injury care	# Patients Receiving Higher Quality Injury care	Patients Perception of Health System	Patients trust in healthcare system	# Injured seeking non-formal care	Willingness to seek formal care	# Injured seeking care								
181	12	Care Quality Loop	Patients trust in healthcare system	# Injured seeking non-formal care	Willingness to seek formal care	# Injured seeking care	# Patients taking other transport	# Patients in Definitive Care on other Transport	# Patients in Definitive Care	# Patients Receiving Higher Quality Injury care	Willingness to remain in care	# Patients remaining in care/rehab	# Patients Receiving Higher Quality Rehab								

182	12	Care Quality Loop	Total available workforce	GDP	Poverty	Community Conflict Violence etc	Availability of quality injury care	# Patients receiving Lower Quality Injury Care	Patients Perception of Health System	Patients trust in healthcare system	Willingness to remain in care	# Patients remaining in care/rehab	# Patients Receiving Higher Quality Rehab								
183	12	Care Quality Loop	Patients trust in healthcare system	# Injured seeking non-formal care	Willingness to seek formal care	# Injured seeking care	# Patients taking an ambulance	# Patients in Definitive Care on Ambulance	# Patients in Definitive Care	# Patients receiving Lower Quality Injury Care	Willingness to remain in care	# Patients remaining in care/rehab	# Patients receiving Lower Quality Rehab								
184	12	Care Quality Loop	Total available workforce	GDP	System level funding	Available Funding for injury care	Availability of quality injury rehab or follow-up	Discrimination	# Patients Receiving Higher Quality Injury care	Patients Perception of Health System	Patients trust in healthcare system	Willingness to seek formal care	# Injured seeking care								
185	12	Care Quality Loop	Total available workforce	GDP	System level funding	Available Funding for injury care	Availability of quality injury care	# Patients Receiving Higher Quality Injury care	Patients Perception of Health System	Patients trust in healthcare system	Willingness to remain in care	# Patients remaining in care/rehab	# Patients Receiving Higher Quality Rehab								
186	12	Care Quality Loop	Total available workforce	GDP	System level funding	Available Funding for injury care	Availability of quality injury care	# Patients Receiving Higher Quality Injury care	Patients Perception of Health System	Patients trust in healthcare system	Willingness to remain in care	# Patients remaining in care/rehab	# Patients receiving Lower Quality Rehab								
187	12	Care Quality Loop	Patients trust in healthcare system	# Injured seeking non-formal care	Willingness to seek formal care	# Injured seeking care	# Patients taking an ambulance	# Patients in Definitive Care on Ambulance	# Patients in Definitive Care	# Patients receiving Lower Quality Injury Care	Willingness to remain in care	# Patients remaining in care/rehab	# Patients Receiving Higher Quality Rehab								
188	12	Care Quality Loop	Total available workforce	GDP	Poverty	Community Conflict Violence etc	Availability of quality injury care	# Patients Receiving Higher Quality Injury care	Patients Perception of Health System	Patients trust in healthcare system	Willingness to receive care	# Patients in Definitive Care	# Patients receiving Lower Quality Injury Care								
189	12	Care Quality Loop	Total available workforce	GDP	System level funding	Available Funding for injury care	Availability of quality injury care	Discrimination	# Patients Receiving Higher Quality Injury care	Patients Perception of Health System	Patients trust in healthcare system	Willingness to seek formal care	# Injured seeking care								
190	12	Care Quality Loop	Total available workforce	GDP	Poverty	Individual wealth	Ability to pay	Willingness to receive care	# Patients in Definitive Care	# Patients receiving Lower Quality Injury Care	Willingness to remain in care	# Patients remaining in care/rehab	# Patients receiving Lower Quality Rehab								
191	12	Care Quality Loop	Patients trust in healthcare system	# Injured seeking non-formal care	Willingness to seek formal care	# Injured seeking care	# Patients taking other transport	# Patients in Definitive Care on other Transport	# Patients in Definitive Care	# Patients Receiving Higher Quality Injury care	Willingness to remain in care	# Patients remaining in care/rehab	# Patients receiving Lower Quality Rehab								
192	12	Care Quality Loop	Total available workforce	GDP	System level funding	Available Funding for injury care	Availability of Ambulance Service	Geographical accessibility	Availability of other transport	# Patients taking other transport	# Patients in Definitive Care on other Transport	# Patients in Definitive Care	# Patients receiving Lower Quality Injury Care								
193	12	Care Quality Loop	Total available workforce	GDP	System level funding	Available Funding for injury care	Universal health insurance	Ability to pay	Willingness to seek formal care	# Injured seeking care	# Patients taking other transport	# Patients in Definitive Care on other Transport	# Patients in Definitive care Delayed								
194	12	Care Quality Loop	Total available workforce	GDP	System level funding	Available Funding for injury care	Universal health insurance	Ability to pay	Willingness to seek formal care	# Injured seeking care	# Patients taking an ambulance	# Patients in Definitive Care on Ambulance	# Patients in Definitive care on time								
195	12	Care Quality Loop	Total available workforce	GDP	Poverty	Individual wealth	Ability to pay	Willingness to seek formal care	# Injured seeking care	# Patients taking	# Patients in Definitive Care on	# Patients in Definitive Care	# Patients Receiving Higher								

										other transport	other Transport		Quality Injury care								
196	12	Care Quality Loop	Total available workforce	GDP	Poverty	Communit y Conflict Violence etc	Availability of quality injury care	# Patients Receiving Higher Quality Injury care	Patients Perceptio n of Health System	Patients trust in healthcare system	Willingnes s to remain in care	# Patients remaining in care/reha b	# Patients Receiving Higher Quality Rehab								
197	12	Care Quality Loop	Patients trust in healthcar e system	# Injured seeking non- formal care	Willingnes s to seek formal care	# Injured seeking care	# Patients taking other transport	# Patients in Definitive Care on other Transport	# Patients in Definitive Care	# Patients receiving Lower Quality Injury Care	Willingnes s to remain in care	# Patients remaining in care/reha b	# Patients receiving Lower Quality Rehab								
198	12	Care Quality Loop	Patients trust in healthcar e system	# Injured seeking non- formal care	Willingnes s to seek formal care	# Injured seeking care	# Patients taking other transport	# Patients in Definitive Care on other Transport	# Patients in Definitive Care	# Patients receiving Lower Quality Injury Care	Willingnes s to remain in care	# Patients remaining in care/reha b	# Patients Receiving Higher Quality Rehab								
199	12	Care Quality Loop	Patients trust in healthcar e system	# Injured seeking non- formal care	Willingnes s to seek formal care	# Injured seeking care	# Patients taking an ambulance	# Patients in Definitive Care on Ambulance	# Patients in Definitive Care	# Patients Receiving Higher Quality Injury care	Willingnes s to remain in care	# Patients remaining in care/reha b	# Patients Receiving Higher Quality Rehab								
200	12	Care Quality Loop	Total available workforce	GDP	System level funding	Available Funding for injury care	Availability of quality injury rehab or follow-up	Discriminatio n	# Patients receiving Lower Quality Injury Care	Patients Perceptio n of Health System	Patients trust in healthcare system	Willingnes s to seek formal care	# Injured seeking care								
201	12	Care Quality Loop	Total available workforce	GDP	System level funding	Available Funding for injury care	Availability of Ambulance Service	Geographica l accessibility	Availability of other transport	# Patients taking other transport	# Patients in Definitive Care on other Transport	# Patients in Definitive Care	# Patients Receiving Higher Quality Injury care								
202	12	Care Quality Loop	Total available workforce	GDP	Poverty	Individual wealth	Availability of other transport	Geographica l accessibility	Availability of Ambulanc e Service	# Patients taking an ambulanc e	# Patients in Definitive Care on Ambulanc e	# Patients in Definitive Care	# Patients Receiving Higher Quality Injury care								
203	12	Care Quality Loop	Total available workforce	GDP	Poverty	Communit y Conflict Violence etc	Geographic al accessibility	Availability of Ambulance Service	# Patients taking an ambulanc e	# Patients in Definitive Care on Ambulanc e	# Patients in Definitive Care	# Patients remaining in care/reha b	# Patients Receiving Higher Quality Rehab								
204	12	Care Quality Loop	Total available workforce	GDP	Poverty	Individual wealth	Ability to pay	Willingness to receive care	# Patients in Definitive Care	# Patients receiving Lower Quality Injury Care	Patients Perceptio n of Health System	Patients trust in healthcare system	# Injured seeking non- formal care								
205	12	Care Quality Loop	Total available workforce	GDP	Poverty	Communit y Conflict Violence etc	Availability of quality injury care	# Patients receiving Lower Quality Injury Care	Patients Perceptio n of Health System	Patients trust in healthcare system	Willingnes s to seek formal care	# Injured seeking care	# Patients taking an ambulanc e								
206	12	Care Quality Loop	Total available workforce	GDP	Poverty	Communit y Conflict Violence etc	Availability of quality injury rehab or follow-up	Discriminatio n	# Patients receiving Lower Quality Injury Care	Patients Perceptio n of Health System	Patients trust in healthcare system	Willingnes s to seek formal care	# Injured seeking care								
207	12	Care Quality Loop	Total available workforce	GDP	Poverty	Individual wealth	Ability to pay	Willingness to receive care	# Patients in Definitive Care	# Patients Receiving Higher Quality Injury care	Willingnes s to remain in care	# Patients remaining in care/reha b	# Patients Receiving Higher Quality Rehab								
208	12	Care Quality Loop	Total available workforce	GDP	Poverty	Individual wealth	Ability to pay	Willingness to receive care	# Patients in Definitive Care	# Patients Receiving Higher Quality Injury care	Patients Perceptio n of Health System	Patients trust in healthcare system	# Injured seeking non- formal care								

209	12	Care Quality Loop	Total available workforce	GDP	System level funding	Available Funding for injury care	Availability of quality injury care	# Patients Receiving Higher Quality Injury care	Patients Perception of Health System	Patients trust in healthcare system	# Injured seeking non-formal care	Willingness to seek formal care	# Injured seeking care								
210	12	Care Quality Loop	Total available workforce	GDP	System level funding	Available Funding for injury care	Availability of quality injury care	# Patients receiving Lower Quality Injury Care	Patients Perception of Health System	Patients trust in healthcare system	# Injured seeking non-formal care	Willingness to seek formal care	# Injured seeking care								
211	12	Care Quality Loop	Total available workforce	GDP	System level funding	Available Funding for injury care	Availability of quality injury care	# Patients Receiving Higher Quality Injury care	Patients Perception of Health System	Patients trust in healthcare system	Willingness to seek formal care	# Injured seeking care	# Patients taking an ambulance								
212	12	Care Quality Loop	Total available workforce	GDP	Poverty	Community Conflict Violence etc	Availability of quality injury care	Discrimination	# Patients receiving Lower Quality Injury Care	Patients Perception of Health System	Patients trust in healthcare system	Willingness to seek formal care	# Injured seeking care								
213	12	Care Quality Loop	Total available workforce	GDP	Poverty	Community Conflict Violence etc	Availability of quality injury care	Discrimination	# Patients Receiving Higher Quality Injury care	Patients Perception of Health System	Patients trust in healthcare system	Willingness to seek formal care	# Injured seeking care								
214	12	Care Quality Loop	Total available workforce	GDP	Poverty	Community Conflict Violence etc	Geographical accessibility	Availability of other transport	# Patients taking other transport	# Patients in Definitive Care on other Transport	# Patients in Definitive Care	# Patients remaining in care/rehab	# Patients receiving Lower Quality Rehab								
215	12	Care Quality Loop	Patients trust in healthcare system	# Injured seeking non-formal care	Willingness to seek formal care	# Injured seeking care	# Patients taking an ambulance	# Patients in Definitive Care on Ambulance	# Patients in Definitive Care	# Patients Receiving Higher Quality Injury care	Willingness to remain in care	# Patients remaining in care/rehab	# Patients receiving Lower Quality Rehab								
216	12	Care Quality Loop	Total available workforce	GDP	System level funding	Available Funding for injury care	Availability of quality injury care	# Patients receiving Lower Quality Injury Care	Patients Perception of Health System	Patients trust in healthcare system	Willingness to remain in care	# Patients remaining in care/rehab	# Patients receiving Lower Quality Rehab								
217	12	Care Quality Loop	Total available workforce	GDP	Poverty	Community Conflict Violence etc	Geographical accessibility	Availability of Ambulance Service	# Patients taking an ambulance	# Patients in Definitive Care on Ambulance	# Patients in Definitive Care	# Patients remaining in care/rehab	# Patients receiving Lower Quality Rehab								
218	12	Care Quality Loop	Total available workforce	GDP	System level funding	Available Funding for injury care	Availability of quality injury care	# Patients receiving Lower Quality Injury Care	Patients Perception of Health System	Patients trust in healthcare system	Willingness to remain in care	# Patients remaining in care/rehab	# Patients Receiving Higher Quality Rehab								
219	12	Care Quality Loop	Total available workforce	GDP	Poverty	Community Conflict Violence etc	Availability of quality injury care	# Patients receiving Lower Quality Injury Care	Patients Perception of Health System	Patients trust in healthcare system	# Injured seeking non-formal care	Willingness to seek formal care	# Injured seeking care								
220	12	Care Quality Loop	Total available workforce	GDP	System level funding	Available Funding for injury care	Universal health insurance	Ability to pay	Willingness to seek formal care	# Injured seeking care	# Patients taking other transport	# Patients in Definitive Care on other Transport	# Patients in Definitive care on time								
221	12	Care Quality Loop	Total available workforce	GDP	Poverty	Individual wealth	Ability to pay	Willingness to seek formal care	# Injured seeking care	# Patients taking an ambulance	# Patients in Definitive Care on Ambulance	# Patients in Definitive Care	# Patients receiving Lower Quality Injury Care								
222	12	Care Quality Loop	Total available workforce	GDP	Poverty	Individual wealth	Ability to pay	Willingness to receive care	# Patients in Definitive Care	# Patients Receiving Higher Quality Injury care	Willingness to remain in care	# Patients remaining in care/rehab	# Patients receiving Lower Quality Rehab								

223	12	Care Quality Loop	Total available workforce	GDP	Poverty	Community Conflict Violence etc	Availability of quality injury rehab or follow-up	Discrimination	# Patients Receiving Higher Quality Injury care	Patients Perception of Health System	Patients trust in healthcare system	Willingness to seek formal care	# Injured seeking care								
224	12	Care Quality Loop	Total available workforce	GDP	System level funding	Available Funding for injury care	Availability of quality injury care	# Patients receiving Lower Quality Injury Care	Patients Perception of Health System	Patients trust in healthcare system	Willingness to receive care	# Patients in Definitive Care	# Patients Receiving Higher Quality Injury care								
225	12	Care Quality Loop	Total available workforce	GDP	Poverty	Community Conflict Violence etc	Availability of quality injury care	# Patients Receiving Higher Quality Injury care	Patients Perception of Health System	Patients trust in healthcare system	Willingness to remain in care	# Patients remaining in care/rehab	# Patients receiving Lower Quality Rehab								
226	12	Care Quality Loop	Total available workforce	GDP	System level funding	Available Funding for injury care	Availability of quality injury care	Discrimination	# Patients receiving Lower Quality Injury Care	Patients Perception of Health System	Patients trust in healthcare system	Willingness to seek formal care	# Injured seeking care								
227	12	Care Quality Loop	Total available workforce	GDP	Poverty	Individual wealth	Ability to pay	Willingness to seek formal care	# Injured seeking care	# Patients taking an ambulance	# Patients in Definitive Care on Ambulance	# Patients in Definitive Care	# Patients Receiving Higher Quality Injury care								
228	12	Care Quality Loop	Total available workforce	GDP	Poverty	Community Conflict Violence etc	Availability of quality injury care	# Patients Receiving Higher Quality Injury care	Patients Perception of Health System	Patients trust in healthcare system	Willingness to seek formal care	# Injured seeking care	# Patients taking an ambulance								
229	12	Care Quality Loop	Total available workforce	GDP	Poverty	Community Conflict Violence etc	Geographical accessibility	Availability of other transport	# Patients taking other transport	# Patients in Definitive Care on other Transport	# Patients in Definitive Care	# Patients remaining in care/rehab	# Patients Receiving Higher Quality Rehab								
230	12	Care Quality Loop	Total available workforce	GDP	Poverty	Community Conflict Violence etc	Availability of quality injury care	# Patients receiving Lower Quality Injury Care	Patients Perception of Health System	Patients trust in healthcare system	Willingness to receive care	# Patients in Definitive Care	# Patients Receiving Higher Quality Injury care								
231	13	Funding & System Capacity Loop	Total available workforce	GDP	System level funding	Available Funding for injury care	Availability of quality injury care	Discrimination	# Patients Receiving Higher Quality Injury care	Patients Perception of Health System	Patients trust in healthcare system	Willingness to remain in care	# Patients remaining in care/rehab	# Patients receiving Lower Quality Rehab							
232	13	Funding & System Capacity Loop	Total available workforce	GDP	System level funding	Available Funding for injury care	Availability of Ambulance Service	# Patients taking an ambulance	# Patients in Definitive Care on Ambulance	# Patients in Definitive Care	# Patients Receiving Higher Quality Injury care	Willingness to remain in care	# Patients remaining in care/rehab	# Patients Receiving Higher Quality Rehab							
233	13	Funding & System Capacity Loop	Total available workforce	GDP	Poverty	Community Conflict Violence etc	Availability of quality injury care	# Patients Receiving Higher Quality Injury care	Patients Perception of Health System	Patients trust in healthcare system	Willingness to receive care	# Patients in Definitive Care	# Patients remaining in care/rehab	# Patients receiving Lower Quality Rehab							
234	13	Funding & System Capacity Loop	Total available workforce	GDP	System level funding	Available Funding for injury care	Availability of quality injury care	# Patients receiving Lower Quality Injury Care	Patients Perception of Health System	Patients trust in healthcare system	Willingness to receive care	# Patients in Definitive Care	# Patients remaining in care/rehab	# Patients receiving Lower Quality Rehab							
235	13	Funding & System Capacity Loop	Total available workforce	GDP	System level funding	Available Funding for injury care	Availability of Ambulance Service	# Patients taking an ambulance	# Patients in Definitive Care on Ambulance	# Patients in Definitive Care	# Patients receiving Lower Quality Injury Care	Patients Perception of Health System	Patients trust in healthcare system	# Injured seeking non-formal care							
236	13	Funding & System Capacity Loop	Total available workforce	GDP	System level funding	Available Funding for injury care	Universal health insurance	Ability to pay	Willingness to seek formal care	# Injured seeking care	# Patients taking an ambulance	# Patients in Definitive Care on Ambulance	# Patients in Definitive Care	# Patients Receiving Higher Quality Injury care							

237	13	Funding & System Capacity Loop	Total available workforce	GDP	Poverty	Individual wealth	Ability to pay	Willingness to receive care	# Patients in Definitive Care	# Patients Receiving Higher Quality Injury care	Patients Perceptio n of Health System	Patients trust in healthcare system	Willingnes s to seek formal care	# Injured seeking care							
238	13	Funding & System Capacity Loop	Total available workforce	GDP	System level funding	Available Funding for injury care	Availability of quality injury care	Discriminatio n	# Patients Receiving Higher Quality Injury care	Patients Perceptio n of Health System	Patients trust in healthcare system	# Injured seeking non-formal care	Willingnes s to seek formal care	# Injured seeking care							
239	13	Funding & System Capacity Loop	Total available workforce	GDP	Poverty	Individual wealth	Ability to pay	Willingness to seek formal care	# Injured seeking care	# Patients taking other transport	# Patients in Definitive Care on other Transport	# Patients in Definitive Care	# Patients remaining in care/reha b	# Patients receiving Lower Quality Rehab							
240	13	Funding & System Capacity Loop	Total available workforce	GDP	System level funding	Available Funding for injury care	Availability of quality injury rehab or follow-up	Discriminatio n	# Patients Receiving Higher Quality Injury care	Patients Perceptio n of Health System	Patients trust in healthcare system	Willingnes s to remain in care	# Patients remaining in care/reha b	# Patients receiving Lower Quality Rehab							
241	13	Funding & System Capacity Loop	Total available workforce	GDP	System level funding	Available Funding for injury care	Universal health insurance	Ability to pay	Willingnes s to seek formal care	# Injured seeking care	# Patients taking other transport	# Patients in Definitive Care on other Transport	# Patients in Definitive Care	# Patients receiving Lower Quality Injury Care							
242	13	Funding & System Capacity Loop	Total available workforce	GDP	Poverty	Communit y Conflict Violence etc	Availability of quality injury care	# Patients Receiving Higher Quality Injury care	Patients Perceptio n of Health System	Patients trust in healthcare system	Willingnes s to receive care	# Patients in Definitive Care	# Patients remaining in care/reha b	# Patients Receiving Higher Quality Rehab							
243	13	Funding & System Capacity Loop	Total available workforce	GDP	Poverty	Communit y Conflict Violence etc	Availability of quality injury rehab or follow-up	Discriminatio n	# Patients receiving Lower Quality Injury Care	Patients Perceptio n of Health System	Patients trust in healthcare system	Willingnes s to seek formal care	# Injured seeking care	# Patients taking an ambulanc e							
244	13	Funding & System Capacity Loop	Total available workforce	GDP	Poverty	Individual wealth	Ability to pay	Willingness to seek formal care	# Injured seeking care	# Patients taking an ambulanc e	# Patients in Definitive Care on Ambulanc e	# Patients in Definitive Care	# Patients remaining in care/reha b	# Patients Receiving Higher Quality Rehab							
245	13	Funding & System Capacity Loop	Total available workforce	GDP	System level funding	Available Funding for injury care	Availability of quality injury care	# Patients Receiving Higher Quality Injury care	Patients Perceptio n of Health System	Patients trust in healthcare system	# Injured seeking non-formal care	Willingnes s to seek formal care	# Injured seeking care	# Patients taking an ambulanc e							
246	13	Funding & System Capacity Loop	Total available workforce	GDP	System level funding	Available Funding for injury care	Universal health insurance	Ability to pay	Willingnes s to receive care	# Patients in Definitive Care	# Patients Receiving Higher Quality Injury care	Patients Perceptio n of Health System	Patients trust in healthcare system	# Injured seeking non-formal care							
247	13	Funding & System Capacity Loop	Total available workforce	GDP	System level funding	Available Funding for injury care	Availability of quality injury rehab or follow-up	Discriminatio n	# Patients receiving Lower Quality Injury Care	Patients Perceptio n of Health System	Patients trust in healthcare system	# Injured seeking non-formal care	Willingnes s to seek formal care	# Injured seeking care							
248	13	Funding & System Capacity Loop	Total available workforce	GDP	System level funding	Available Funding for injury care	Universal health insurance	Ability to pay	Willingnes s to seek formal care	# Injured seeking care	# Patients taking other transport	# Patients in Definitive Care on other Transport	# Patients in Definitive Care	# Patients Receiving Higher Quality Injury care							
249	13	Funding & System Capacity Loop	Total available workforce	GDP	Poverty	Individual wealth	Availability of other transport	# Patients taking other transport	# Patients in Definitive Care on other Transport	# Patients in Definitive Care	# Patients Receiving Higher Quality Injury care	Willingnes s to remain in care	# Patients remaining in care/reha b	# Patients receiving Lower Quality Rehab							
250	13	Funding & System Capacity Loop	Total available workforce	GDP	System level funding	Available Funding for injury care	Availability of Ambulance Service	# Patients taking an ambulance	# Patients in Definitive Care on Ambulanc e	# Patients in Definitive Care	# Patients receiving Lower Quality	Willingnes s to remain in care	# Patients remaining in care/reha b	# Patients Receiving Higher Quality Rehab							

											Injury Care										
251	13	Funding & System Capacity Loop	Total available workforce	GDP	Poverty	Individual wealth	Availability of other transport	# Patients taking other transport	# Patients in Definitive Care on other Transport	# Patients in Definitive Care	# Patients receiving Lower Quality Injury Care	Willingness to remain in care	# Patients remaining in care/rehab	# Patients Receiving Higher Quality Rehab							
252	13	Funding & System Capacity Loop	Total available workforce	GDP	System level funding	Available Funding for injury care	Availability of quality injury care	Discrimination	# Patients Receiving Higher Quality Injury care	Patients Perception of Health System	Patients trust in healthcare system	Willingness to receive care	# Patients in Definitive Care	# Patients receiving Lower Quality Injury Care							
253	13	Funding & System Capacity Loop	Total available workforce	GDP	Poverty	Community Conflict Violence etc	Availability of quality injury care	Discrimination	# Patients receiving Lower Quality Injury Care	Patients Perception of Health System	Patients trust in healthcare system	Willingness to remain in care	# Patients remaining in care/rehab	# Patients receiving Lower Quality Rehab							
254	13	Funding & System Capacity Loop	Total available workforce	GDP	Poverty	Community Conflict Violence etc	Availability of quality injury care	Discrimination	# Patients Receiving Higher Quality Injury care	Patients Perception of Health System	Patients trust in healthcare system	Willingness to remain in care	# Patients remaining in care/rehab	# Patients receiving Lower Quality Rehab							
255	13	Funding & System Capacity Loop	Total available workforce	GDP	System level funding	Available Funding for injury care	Universal health insurance	Ability to pay	Willingness to receive care	# Patients in Definitive Care	# Patients receiving Lower Quality Injury Care	Willingness to remain in care	# Patients remaining in care/rehab	# Patients receiving Lower Quality Rehab							
256	13	Funding & System Capacity Loop	Total available workforce	GDP	Poverty	Individual wealth	Ability to pay	Willingness to seek formal care	# Injured seeking care	# Patients taking an ambulance	# Patients in Definitive Care on Ambulance	# Patients in Definitive Care	# Patients remaining in care/rehab	# Patients receiving Lower Quality Rehab							
257	13	Funding & System Capacity Loop	Total available workforce	GDP	System level funding	Available Funding for injury care	Universal health insurance	Ability to pay	Willingness to receive care	# Patients in Definitive Care	# Patients receiving Lower Quality Injury Care	Willingness to remain in care	# Patients remaining in care/rehab	# Patients Receiving Higher Quality Rehab							
258	13	Funding & System Capacity Loop	Total available workforce	GDP	Poverty	Community Conflict Violence etc	Availability of quality injury rehab or follow-up	Discrimination	# Patients Receiving Higher Quality Injury care	Patients Perception of Health System	Patients trust in healthcare system	# Injured seeking non-formal care	Willingness to seek formal care	# Injured seeking care							
259	13	Funding & System Capacity Loop	Total available workforce	GDP	System level funding	Available Funding for injury care	Universal health insurance	Ability to pay	Willingness to receive care	# Patients in Definitive Care	# Patients receiving Lower Quality Injury Care	Patients Perception of Health System	Patients trust in healthcare system	# Injured seeking non-formal care							
260	13	Funding & System Capacity Loop	Total available workforce	GDP	System level funding	Available Funding for injury care	Availability of quality injury care	Discrimination	# Patients Receiving Higher Quality Injury care	Patients Perception of Health System	Patients trust in healthcare system	Willingness to remain in care	# Patients remaining in care/rehab	# Patients Receiving Higher Quality Rehab							
261	13	Funding & System Capacity Loop	Total available workforce	GDP	Poverty	Community Conflict Violence etc	Availability of quality injury care	Discrimination	# Patients Receiving Higher Quality Injury care	Patients Perception of Health System	Patients trust in healthcare system	Willingness to receive care	# Patients in Definitive Care	# Patients receiving Lower Quality Injury Care							
262	13	Funding & System Capacity Loop	Total available workforce	GDP	Poverty	Community Conflict Violence etc	Availability of quality injury care	Discrimination	# Patients Receiving Higher Quality Injury care	Patients Perception of Health System	Patients trust in healthcare system	# Injured seeking non-formal care	Willingness to seek formal care	# Injured seeking care							
263	13	Funding & System Capacity Loop	Total available workforce	GDP	Poverty	Community Conflict Violence etc	Availability of quality injury care	Discrimination	# Patients receiving Lower Quality	Patients Perception of	Patients trust in healthcare system	Willingness to seek formal care	# Injured seeking care	# Patients taking an ambulance							

									Injury Care	Health System											
264	13	Funding & System Capacity Loop	Total available workforce	GDP	System level funding	Available Funding for injury care	Availability of quality injury rehab or follow-up	Discrimination	# Patients Receiving Higher Quality Injury care	Patients Perception of Health System	Patients trust in healthcare system	Willingness to seek formal care	# Injured seeking care	# Patients taking an ambulance							
265	13	Funding & System Capacity Loop	Total available workforce	GDP	Poverty	Individual wealth	Availability of other transport	# Patients taking other transport	# Patients in Definitive Care on other Transport	# Patients in Definitive Care	# Patients receiving Lower Quality Injury Care	Willingness to remain in care	# Patients remaining in care/rehab	# Patients receiving Lower Quality Rehab							
266	13	Funding & System Capacity Loop	Total available workforce	GDP	Poverty	Individual wealth	Availability of other transport	# Patients taking other transport	# Patients in Definitive Care on other Transport	# Patients in Definitive Care	# Patients Receiving Higher Quality Injury care	Willingness to remain in care	# Patients remaining in care/rehab	# Patients Receiving Higher Quality Rehab							
267	13	Funding & System Capacity Loop	Total available workforce	GDP	System level funding	Available Funding for injury care	Availability of quality injury care	Discrimination	# Patients receiving Lower Quality Injury Care	Patients Perception of Health System	Patients trust in healthcare system	Willingness to receive care	# Patients in Definitive Care	# Patients Receiving Higher Quality Injury care							
268	13	Funding & System Capacity Loop	Total available workforce	GDP	System level funding	Available Funding for injury care	Availability of quality injury care	Discrimination	# Patients receiving Lower Quality Injury Care	Patients Perception of Health System	Patients trust in healthcare system	# Injured seeking non-formal care	Willingness to seek formal care	# Injured seeking care							
269	13	Funding & System Capacity Loop	Total available workforce	GDP	Poverty	Community Conflict Violence etc	Availability of quality injury care	Discrimination	# Patients receiving Lower Quality Injury Care	Patients Perception of Health System	Patients trust in healthcare system	Willingness to remain in care	# Patients remaining in care/rehab	# Patients Receiving Higher Quality Rehab							
270	13	Funding & System Capacity Loop	Total available workforce	GDP	Poverty	Community Conflict Violence etc	Availability of quality injury rehab or follow-up	Discrimination	# Patients Receiving Higher Quality Injury care	Patients Perception of Health System	Patients trust in healthcare system	Willingness to receive care	# Patients in Definitive Care	# Patients receiving Lower Quality Injury Care							
271	13	Funding & System Capacity Loop	Total available workforce	GDP	System level funding	Available Funding for injury care	Availability of Ambulance Service	# Patients taking an ambulance	# Patients in Definitive Care on Ambulance	# Patients in Definitive Care	# Patients Receiving Higher Quality Injury care	Patients Perception of Health System	Patients trust in healthcare system	# Injured seeking non-formal care							
272	13	Funding & System Capacity Loop	Total available workforce	GDP	Poverty	Community Conflict Violence etc	Availability of quality injury rehab or follow-up	Discrimination	# Patients receiving Lower Quality Injury Care	Patients Perception of Health System	Patients trust in healthcare system	# Injured seeking non-formal care	Willingness to seek formal care	# Injured seeking care							
273	13	Funding & System Capacity Loop	Total available workforce	GDP	Poverty	Community Conflict Violence etc	Availability of quality injury care	# Patients receiving Lower Quality Injury Care	Patients Perception of Health System	Patients trust in healthcare system	Willingness to receive care	# Patients in Definitive Care	# Patients remaining in care/rehab	# Patients receiving Lower Quality Rehab							
274	13	Funding & System Capacity Loop	Total available workforce	GDP	Poverty	Community Conflict Violence etc	Availability of quality injury rehab or follow-up	Discrimination	# Patients Receiving Higher Quality Injury care	Patients Perception of Health System	Patients trust in healthcare system	Willingness to seek formal care	# Injured seeking care	# Patients taking an ambulance							
275	13	Funding & System Capacity Loop	Total available workforce	GDP	System level funding	Available Funding for injury care	Universal health insurance	Ability to pay	Willingness to receive care	# Patients in Definitive Care	# Patients Receiving Higher Quality Injury care	Willingness to remain in care	# Patients remaining in care/rehab	# Patients receiving Lower Quality Rehab							
276	13	Funding & System Capacity Loop	Total available workforce	GDP	Poverty	Community Conflict Violence etc	Availability of quality injury care	Discrimination	# Patients receiving Lower Quality	Patients Perception of Health System	Patients trust in healthcare system	# Injured seeking non-formal care	Willingness to seek formal care	# Injured seeking care							

									Injury Care												
277	13	Funding & System Capacity Loop	Total available workforce	GDP	Poverty	Individual wealth	Availability of other transport	# Patients taking other transport	# Patients in Definitive Care on other Transport	# Patients in Definitive Care	# Patients receiving Lower Quality Injury Care	Patients Perception of Health System	Patients trust in healthcare system	# Injured seeking non-formal care							
278	13	Funding & System Capacity Loop	Total available workforce	GDP	System level funding	Available Funding for injury care	Availability of quality injury care	# Patients receiving Lower Quality Injury Care	Patients Perception of Health System	Patients trust in healthcare system	Willingness to receive care	# Patients in Definitive Care	# Patients remaining in care/rehab	# Patients Receiving Higher Quality Rehab							
279	13	Funding & System Capacity Loop	Total available workforce	GDP	Poverty	Individual wealth	Ability to pay	Willingness to receive care	# Patients in Definitive Care	# Patients receiving Lower Quality Injury Care	Patients Perception of Health System	Patients trust in healthcare system	Willingness to seek formal care	# Injured seeking care							
280	13	Funding & System Capacity Loop	Total available workforce	GDP	Poverty	Individual wealth	Availability of other transport	Geographical accessibility	Availability of Ambulance Service	# Patients taking an ambulance	# Patients in Definitive Care on Ambulance	# Patients in Definitive Care	# Patients remaining in care/rehab	# Patients Receiving Higher Quality Rehab							
281	13	Funding & System Capacity Loop	Total available workforce	GDP	Poverty	Community Conflict Violence etc	Availability of quality injury care	Discrimination	# Patients Receiving Higher Quality Injury care	Patients Perception of Health System	Patients trust in healthcare system	Willingness to seek formal care	# Injured seeking care	# Patients taking an ambulance							
282	13	Funding & System Capacity Loop	Total available workforce	GDP	System level funding	Available Funding for injury care	Availability of quality injury rehab or follow-up	Discrimination	# Patients receiving Lower Quality Injury Care	Patients Perception of Health System	Patients trust in healthcare system	Willingness to seek formal care	# Injured seeking care	# Patients taking an ambulance							
283	13	Funding & System Capacity Loop	Total available workforce	GDP	System level funding	Available Funding for injury care	Availability of quality injury rehab or follow-up	Discrimination	# Patients receiving Lower Quality Injury Care	Patients Perception of Health System	Patients trust in healthcare system	Willingness to remain in care	# Patients remaining in care/rehab	# Patients receiving Lower Quality Rehab							
284	13	Funding & System Capacity Loop	Total available workforce	GDP	System level funding	Available Funding for injury care	Availability of quality injury rehab or follow-up	Discrimination	# Patients Receiving Higher Quality Injury care	Patients Perception of Health System	Patients trust in healthcare system	Willingness to remain in care	# Patients remaining in care/rehab	# Patients Receiving Higher Quality Rehab							
285	13	Funding & System Capacity Loop	Total available workforce	GDP	Poverty	Individual wealth	Ability to pay	Willingness to seek formal care	# Injured seeking care	# Patients taking other transport	# Patients in Definitive Care on other Transport	# Patients in Definitive Care	# Patients remaining in care/rehab	# Patients Receiving Higher Quality Rehab							
286	13	Funding & System Capacity Loop	Total available workforce	GDP	Poverty	Community Conflict Violence etc	Availability of quality injury care	# Patients receiving Lower Quality Injury Care	Patients Perception of Health System	Patients trust in healthcare system	# Injured seeking non-formal care	Willingness to seek formal care	# Injured seeking care	# Patients taking an ambulance							
287	13	Funding & System Capacity Loop	Total available workforce	GDP	Poverty	Community Conflict Violence etc	Availability of quality injury rehab or follow-up	Discrimination	# Patients receiving Lower Quality Injury Care	Patients Perception of Health System	Patients trust in healthcare system	Willingness to receive care	# Patients in Definitive Care	# Patients Receiving Higher Quality Injury care							
288	13	Funding & System Capacity Loop	Total available workforce	GDP	System level funding	Available Funding for injury care	Availability of Ambulance Service	Geographical accessibility	Availability of other transport	# Patients taking other transport	# Patients in Definitive Care on other Transport	# Patients in Definitive Care	# Patients remaining in care/rehab	# Patients receiving Lower Quality Rehab							
289	13	Funding & System Capacity Loop	Total available workforce	GDP	System level funding	Available Funding for injury care	Availability of quality injury care	Discrimination	# Patients receiving Lower Quality Injury Care	Patients Perception of Health System	Patients trust in healthcare system	Willingness to remain in care	# Patients remaining in care/rehab	# Patients receiving Lower Quality Rehab							

290	13	Funding & System Capacity Loop	Total available workforce	GDP	System level funding	Available Funding for injury care	Availability of Ambulance Service	# Patients taking an ambulance	# Patients in Definitive Care on Ambulance	# Patients in Definitive Care	# Patients Receiving Higher Quality Injury care	Willingness to remain in care	# Patients remaining in care/rehab	# Patients receiving Lower Quality Rehab							
291	13	Funding & System Capacity Loop	Total available workforce	GDP	Poverty	Individual wealth	Availability of other transport	# Patients taking other transport	# Patients in Definitive Care on other Transport	# Patients in Definitive Care	# Patients Receiving Higher Quality Injury care	Patients Perception of Health System	Patients trust in healthcare system	# Injured seeking non-formal care							
292	13	Funding & System Capacity Loop	Total available workforce	GDP	System level funding	Available Funding for injury care	Availability of quality injury rehab or follow-up	Discrimination	# Patients Receiving Higher Quality Injury care	Patients Perception of Health System	Patients trust in healthcare system	# Injured seeking non-formal care	Willingness to seek formal care	# Injured seeking care							
293	13	Funding & System Capacity Loop	Total available workforce	GDP	Poverty	Community Conflict Violence etc	Availability of quality injury rehab or follow-up	Discrimination	# Patients receiving Lower Quality Injury Care	Patients Perception of Health System	Patients trust in healthcare system	Willingness to remain in care	# Patients remaining in care/rehab	# Patients Receiving Higher Quality Rehab							
294	13	Funding & System Capacity Loop	Total available workforce	GDP	Poverty	Community Conflict Violence etc	Availability of quality injury rehab or follow-up	Discrimination	# Patients Receiving Higher Quality Injury care	Patients Perception of Health System	Patients trust in healthcare system	Willingness to remain in care	# Patients remaining in care/rehab	# Patients Receiving Higher Quality Rehab							
295	13	Funding & System Capacity Loop	Total available workforce	GDP	System level funding	Available Funding for injury care	Availability of quality injury care	Discrimination	# Patients Receiving Higher Quality Injury care	Patients Perception of Health System	Patients trust in healthcare system	Willingness to seek formal care	# Injured seeking care	# Patients taking an ambulance							
296	13	Funding & System Capacity Loop	Total available workforce	GDP	Poverty	Community Conflict Violence etc	Availability of quality injury care	# Patients receiving Lower Quality Injury Care	Patients Perception of Health System	Patients trust in healthcare system	Willingness to receive care	# Patients in Definitive Care	# Patients remaining in care/rehab	# Patients Receiving Higher Quality Rehab							
297	13	Funding & System Capacity Loop	Total available workforce	GDP	Poverty	Individual wealth	Availability of other transport	Geographical accessibility	Availability of Ambulance Service	# Patients taking an ambulance	# Patients in Definitive Care on Ambulance	# Patients in Definitive Care	# Patients remaining in care/rehab	# Patients receiving Lower Quality Rehab							
298	13	Funding & System Capacity Loop	Total available workforce	GDP	System level funding	Available Funding for injury care	Availability of quality injury care	Discrimination	# Patients receiving Lower Quality Injury Care	Patients Perception of Health System	Patients trust in healthcare system	Willingness to seek formal care	# Injured seeking care	# Patients taking an ambulance							
299	13	Funding & System Capacity Loop	Total available workforce	GDP	Poverty	Community Conflict Violence etc	Availability of quality injury care	Discrimination	# Patients Receiving Higher Quality Injury care	Patients Perception of Health System	Patients trust in healthcare system	Willingness to remain in care	# Patients remaining in care/rehab	# Patients Receiving Higher Quality Rehab							
300	13	Funding & System Capacity Loop	Total available workforce	GDP	Poverty	Community Conflict Violence etc	Availability of quality injury care	# Patients Receiving Higher Quality Injury care	Patients Perception of Health System	Patients trust in healthcare system	# Injured seeking non-formal care	Willingness to seek formal care	# Injured seeking care	# Patients taking an ambulance							
301	13	Funding & System Capacity Loop	Total available workforce	GDP	System level funding	Available Funding for injury care	Availability of quality injury rehab or follow-up	Discrimination	# Patients receiving Lower Quality Injury Care	Patients Perception of Health System	Patients trust in healthcare system	Willingness to remain in care	# Patients remaining in care/rehab	# Patients Receiving Higher Quality Rehab							
302	13	Funding & System Capacity Loop	Total available workforce	GDP	System level funding	Available Funding for injury care	Availability of quality injury rehab or follow-up	Discrimination	# Patients receiving Lower Quality Injury Care	Patients Perception of Health System	Patients trust in healthcare system	Willingness to receive care	# Patients in Definitive Care	# Patients Receiving Higher Quality Injury care							
303	13	Funding & System Capacity Loop	Total available workforce	GDP	System level funding	Available Funding for injury care	Availability of quality injury care	# Patients Receiving Higher	Patients Perception of	Patients trust in healthcare system	Willingness to receive care	# Patients in Definitive Care	# Patients remaining in	# Patients receiving Lower							

								Quality Injury care	Health System				care/reha b	Quality Rehab							
304	13	Funding & System Capacity Loop	Total available workforce	GDP	System level funding	Available Funding for injury care	Universal health insurance	Ability to pay	Willingnes s to seek formal care	# Injured seeking care	# Patients taking an ambulanc e	# Patients in Definitive Care on Ambulanc e	# Patients in Definitive Care	# Patients receiving Lower Quality Injury Care							
305	13	Funding & System Capacity Loop	Total available workforce	GDP	System level funding	Available Funding for injury care	Availability of Ambulance Service	# Patients taking an ambulance	# Patients in Definitive Care on Ambulanc e	# Patients in Definitive Care	# Patients receiving Lower Quality Injury Care	Willingnes s to remain in care	# Patients remaining in care/reha b	# Patients receiving Lower Quality Rehab							
306	13	Funding & System Capacity Loop	Total available workforce	GDP	Poverty	Communit y Conflict Violence etc	Availability of quality injury care	Discriminatio n	# Patients receiving Lower Quality Injury Care	Patients Perceptio n of Health System	Patients trust in healthcare system	Willingnes s to receive care	# Patients in Definitive Care	# Patients Receiving Higher Quality Injury care							
307	13	Funding & System Capacity Loop	Total available workforce	GDP	Poverty	Communit y Conflict Violence etc	Availability of quality injury rehab or follow-up	Discriminatio n	# Patients Receiving Higher Quality Injury care	Patients Perceptio n of Health System	Patients trust in healthcare system	Willingnes s to remain in care	# Patients remaining in care/reha b	# Patients receiving Lower Quality Rehab							
308	13	Funding & System Capacity Loop	Total available workforce	GDP	System level funding	Available Funding for injury care	Availability of Ambulance Service	Geographica l accessibility	Availability of other transport	# Patients taking other transport	# Patients in Definitive Care on other Transport	# Patients in Definitive Care	# Patients remaining in care/reha b	# Patients Receiving Higher Quality Rehab							
309	13	Funding & System Capacity Loop	Total available workforce	GDP	Poverty	Communit y Conflict Violence etc	Availability of quality injury rehab or follow-up	Discriminatio n	# Patients receiving Lower Quality Injury Care	Patients Perceptio n of Health System	Patients trust in healthcare system	Willingnes s to remain in care	# Patients remaining in care/reha b	# Patients receiving Lower Quality Rehab							
310	13	Funding & System Capacity Loop	Total available workforce	GDP	System level funding	Available Funding for injury care	Universal health insurance	Ability to pay	Willingnes s to receive care	# Patients in Definitive Care	# Patients Receiving Higher Quality Injury care	Willingnes s to remain in care	# Patients remaining in care/reha b	# Patients Receiving Higher Quality Rehab							
311	13	Funding & System Capacity Loop	Total available workforce	GDP	System level funding	Available Funding for injury care	Availability of quality injury care	# Patients Receiving Higher Quality Injury care	Patients Perceptio n of Health System	Patients trust in healthcare system	Willingnes s to receive care	# Patients in Definitive Care	# Patients remaining in care/reha b	# Patients Receiving Higher Quality Rehab							
312	13	Funding & System Capacity Loop	Total available workforce	GDP	System level funding	Available Funding for injury care	Availability of quality injury rehab or follow-up	Discriminatio n	# Patients Receiving Higher Quality Injury care	Patients Perceptio n of Health System	Patients trust in healthcare system	Willingnes s to receive care	# Patients in Definitive Care	# Patients receiving Lower Quality Injury Care							
313	13	Funding & System Capacity Loop	Total available workforce	GDP	System level funding	Available Funding for injury care	Availability of quality injury care	Discriminatio n	# Patients receiving Lower Quality Injury Care	Patients Perceptio n of Health System	Patients trust in healthcare system	Willingnes s to remain in care	# Patients remaining in care/reha b	# Patients Receiving Higher Quality Rehab							
314	13	Funding & System Capacity Loop	Total available workforce	GDP	System level funding	Available Funding for injury care	Availability of quality injury care	# Patients receiving Lower Quality Injury Care	Patients Perceptio n of Health System	Patients trust in healthcare system	# Injured seeking non-formal care	Willingnes s to seek formal care	# Injured seeking care	# Patients taking an ambulanc e							
315	14	Funding & System Capacity Loop	Total available workforce	GDP	System level funding	Available Funding for injury care	Availability of quality injury care	# Patients receiving Lower Quality Injury Care	Patients Perceptio n of Health System	Patients trust in healthcare system	Willingnes s to seek formal care	# Injured seeking care	# Patients taking other transport	# Patients in Definitive Care on other Transport	# Patients in Definitive care on time						
316	14	Funding & System Capacity Loop	Total available workforce	GDP	System level funding	Available Funding for injury care	Availability of Ambulance Service	# Patients taking an ambulance	# Patients in Definitive Care on Ambulanc e	# Patients in Definitive Care	# Patients receiving Lower Quality	Patients Perceptio n of Health System	Patients trust in healthcare system	Willingnes s to seek formal care	# Injured seeking care						

											Injury Care										
317	14	Funding & System Capacity Loop	Total available workforce	GDP	System level funding	Available Funding for injury care	Availability of quality injury care	# Patients receiving Lower Quality Injury Care	Patients Perception of Health System	Patients trust in healthcare system	Willingness to seek formal care	# Injured seeking care	# Patients taking an ambulance	# Patients in Definitive Care on Ambulance	# Patients in Definitive care on time						
318	14	Funding & System Capacity Loop	Total available workforce	GDP	System level funding	Available Funding for injury care	Universal health insurance	Ability to pay	Willingness to seek formal care	# Injured seeking care	# Patients taking other transport	# Patients in Definitive Care on other Transport	# Patients in Definitive Care	# Patients remaining in care/rehab	# Patients Receiving Higher Quality Rehab						
319	14	Funding & System Capacity Loop	Total available workforce	GDP	Poverty	Individual wealth	Ability to pay	Willingness to receive care	# Patients in Definitive Care	# Patients Receiving Higher Quality Injury care	Patients Perception of Health System	Patients trust in healthcare system	Willingness to remain in care	# Patients remaining in care/rehab	# Patients Receiving Higher Quality Rehab						
320	14	Funding & System Capacity Loop	Total available workforce	GDP	Poverty	Community Conflict Violence etc	Geographical accessibility	Availability of Ambulance Service	# Patients taking an ambulance	# Patients in Definitive Care on Ambulance	# Patients in Definitive Care	# Patients receiving Lower Quality Injury Care	Patients Perception of Health System	Patients trust in healthcare system	# Injured seeking non-formal care						
321	14	Funding & System Capacity Loop	Total available workforce	GDP	System level funding	Available Funding for injury care	Availability of quality injury care	# Patients receiving Lower Quality Injury Care	Patients Perception of Health System	Patients trust in healthcare system	Willingness to seek formal care	# Injured seeking care	# Patients taking other transport	# Patients in Definitive Care on other Transport	# Patients in Definitive care Delayed						
322	14	Funding & System Capacity Loop	Total available workforce	GDP	Poverty	Community Conflict Violence etc	Availability of quality injury care	Discrimination	# Patients Receiving Higher Quality Injury care	Patients Perception of Health System	Patients trust in healthcare system	Willingness to receive care	# Patients in Definitive Care	# Patients remaining in care/rehab	# Patients Receiving Higher Quality Rehab						
323	14	Funding & System Capacity Loop	Total available workforce	GDP	System level funding	Available Funding for injury care	Availability of Ambulance Service	# Patients taking an ambulance	# Patients in Definitive Care on Ambulance	# Patients in Definitive Care	# Patients Receiving Higher Quality Injury care	Patients Perception of Health System	Patients trust in healthcare system	Willingness to seek formal care	# Injured seeking care						
324	14	Funding & System Capacity Loop	Total available workforce	GDP	Poverty	Community Conflict Violence etc	Availability of quality injury rehab or follow-up	Discrimination	# Patients receiving Lower Quality Injury Care	Patients Perception of Health System	Patients trust in healthcare system	Willingness to receive care	# Patients in Definitive Care	# Patients remaining in care/rehab	# Patients receiving Lower Quality Rehab						
325	14	Funding & System Capacity Loop	Total available workforce	GDP	Poverty	Individual wealth	Ability to pay	Willingness to receive care	# Patients in Definitive Care	# Patients Receiving Higher Quality Injury care	Patients Perception of Health System	Patients trust in healthcare system	# Injured seeking non-formal care	Willingness to seek formal care	# Injured seeking care						
326	14	Funding & System Capacity Loop	Total available workforce	GDP	Poverty	Community Conflict Violence etc	Geographical accessibility	Availability of other transport	# Patients taking other transport	# Patients in Definitive Care on other Transport	# Patients in Definitive Care	# Patients Receiving Higher Quality Injury care	Patients Perception of Health System	Patients trust in healthcare system	# Injured seeking non-formal care						
327	14	Funding & System Capacity Loop	Total available workforce	GDP	Poverty	Community Conflict Violence etc	Availability of quality injury care	# Patients Receiving Higher Quality Injury care	Patients Perception of Health System	Patients trust in healthcare system	Willingness to seek formal care	# Injured seeking care	# Patients taking an ambulance	# Patients in Definitive Care on Ambulance	# Patients in Definitive care on time						
328	14	Funding & System Capacity Loop	Total available workforce	GDP	Poverty	Community Conflict Violence etc	Availability of quality injury rehab or follow-up	Discrimination	# Patients receiving Lower Quality Injury Care	Patients Perception of Health System	Patients trust in healthcare system	Willingness to receive care	# Patients in Definitive Care	# Patients remaining in care/rehab	# Patients Receiving Higher Quality Rehab						
329	14	Funding & System Capacity Loop	Total available workforce	GDP	System level funding	Available Funding for injury care	Availability of quality injury rehab or follow-up	Discrimination	# Patients receiving Lower Quality Injury Care	Patients Perception of Health System	Patients trust in healthcare system	Willingness to receive care	# Patients in Definitive Care	# Patients remaining in care/rehab	# Patients Receiving Higher Quality Rehab						

330	14	Funding & System Capacity Loop	Total available workforce	GDP	System level funding	Available Funding for injury care	Availability of quality injury rehab or follow-up	Discriminatio n	# Patients receiving Lower Quality Injury Care	Patients Perceptio n of Health System	Patients trust in healthcare system	Willingnes s to receive care	# Patients in Definitive Care	# Patients remaining in care/reha b	# Patients receiving Lower Quality Rehab						
331	14	Funding & System Capacity Loop	Total available workforce	GDP	Poverty	Communit y Conflict Violence etc	Geographic al accessibility	Availability of other transport	# Patients taking other transport	# Patients in Definitive Care on other Transport	# Patients in Definitive Care	# Patients Receiving Higher Quality Injury care	Willingnes s to remain in care	# Patients remaining in care/reha b	# Patients Receiving Higher Quality Rehab						
332	14	Funding & System Capacity Loop	Total available workforce	GDP	System level funding	Available Funding for injury care	Universal health insurance	Ability to pay	Willingnes s to receive care	# Patients in Definitive Care	# Patients receiving Lower Quality Injury Care	Patients Perceptio n of Health System	Patients trust in healthcare system	Willingnes s to seek formal care	# Injured seeking care						
333	14	Funding & System Capacity Loop	Total available workforce	GDP	Poverty	Communit y Conflict Violence etc	Availability of quality injury rehab or follow-up	Discriminatio n	# Patients Receiving Higher Quality Injury care	Patients Perceptio n of Health System	Patients trust in healthcare system	Willingnes s to receive care	# Patients in Definitive Care	# Patients remaining in care/reha b	# Patients Receiving Higher Quality Rehab						
334	14	Funding & System Capacity Loop	Total available workforce	GDP	System level funding	Available Funding for injury care	Availability of quality injury care	Discriminatio n	# Patients receiving Lower Quality Injury Care	Patients Perceptio n of Health System	Patients trust in healthcare system	Willingnes s to receive care	# Patients in Definitive Care	# Patients remaining in care/reha b	# Patients receiving Lower Quality Rehab						
335	14	Funding & System Capacity Loop	Total available workforce	GDP	Poverty	Communit y Conflict Violence etc	Geographic al accessibility	Availability of Ambulance Service	# Patients taking an ambulanc e	# Patients in Definitive Care on Ambulanc e	# Patients in Definitive Care	# Patients Receiving Higher Quality Injury care	Willingnes s to remain in care	# Patients remaining in care/reha b	# Patients Receiving Higher Quality Rehab						
336	14	Funding & System Capacity Loop	Total available workforce	GDP	Poverty	Individual wealth	Ability to pay	Willingness to receive care	# Patients in Definitive Care	# Patients Receiving Higher Quality Injury care	Patients Perceptio n of Health System	Patients trust in healthcare system	Willingnes s to remain in care	# Patients remaining in care/reha b	# Patients receiving Lower Quality Rehab						
337	14	Funding & System Capacity Loop	Total available workforce	GDP	System level funding	Available Funding for injury care	Universal health insurance	Ability to pay	Willingnes s to seek formal care	# Injured seeking care	# Patients taking other transport	# Patients in Definitive Care on other Transport	# Patients in Definitive Care	# Patients remaining in care/reha b	# Patients receiving Lower Quality Rehab						
338	14	Funding & System Capacity Loop	Total available workforce	GDP	Poverty	Communit y Conflict Violence etc	Availability of quality injury rehab or follow-up	Discriminatio n	# Patients Receiving Higher Quality Injury care	Patients Perceptio n of Health System	Patients trust in healthcare system	# Injured seeking non-formal care	Willingnes s to seek formal care	# Injured seeking care	# Patients taking an ambulanc e						
339	14	Funding & System Capacity Loop	Total available workforce	GDP	Poverty	Communit y Conflict Violence etc	Availability of quality injury rehab or follow-up	Discriminatio n	# Patients Receiving Higher Quality Injury care	Patients Perceptio n of Health System	Patients trust in healthcare system	Willingnes s to receive care	# Patients in Definitive Care	# Patients remaining in care/reha b	# Patients receiving Lower Quality Rehab						
340	14	Funding & System Capacity Loop	Total available workforce	GDP	Poverty	Individual wealth	Ability to pay	Willingness to receive care	# Patients in Definitive Care	# Patients receiving Lower Quality Injury Care	Patients Perceptio n of Health System	Patients trust in healthcare system	Willingnes s to seek formal care	# Injured seeking care	# Patients taking an ambulanc e						
341	14	Funding & System Capacity Loop	Total available workforce	GDP	Poverty	Individual wealth	Availability of other transport	# Patients taking other transport	# Patients in Definitive Care on other Transport	# Patients in Definitive Care	# Patients Receiving Higher Quality Injury care	Patients Perceptio n of Health System	Patients trust in healthcare system	Willingnes s to seek formal care	# Injured seeking care						
342	14	Funding & System Capacity Loop	Total available workforce	GDP	Poverty	Communit y Conflict Violence etc	Geographic al accessibility	Availability of Ambulance Service	# Patients taking an ambulanc e	# Patients in Definitive Care on Ambulanc e	# Patients in Definitive Care	# Patients Receiving Higher Quality Injury care	Willingnes s to remain in care	# Patients remaining in care/reha b	# Patients receiving Lower Quality Rehab						
343	14	Funding & System Capacity Loop	Total available workforce	GDP	Poverty	Communit y Conflict Violence etc	Availability of quality injury care	Discriminatio n	# Patients Receiving Higher	Patients Perceptio n of	Patients trust in healthcare system	Willingnes s to receive care	# Patients in Definitive Care	# Patients remaining in	# Patients receiving Lower						

									Quality Injury care	Health System				care/reha b	Quality Rehab						
344	14	Funding & System Capacity Loop	Total available workforce	GDP	Poverty	Communit y Conflict Violence etc	Geographic al accessibility	Availability of other transport	# Patients taking other transport	# Patients in Definitive Care on other Transport	# Patients in Definitive Care	# Patients receiving Lower Quality Injury Care	Willingnes s to remain in care	# Patients remaining in care/reha b	# Patients receiving Lower Quality Rehab						
345	14	Funding & System Capacity Loop	Total available workforce	GDP	Poverty	Communit y Conflict Violence etc	Availability of quality injury care	Discriminatio n	# Patients receiving Lower Quality Injury Care	Patients Perceptio n of Health System	Patients trust in healthcare system	Willingnes s to receive care	# Patients in Definitive Care	# Patients remaining in care/reha b	# Patients receiving Lower Quality Rehab						
346	14	Funding & System Capacity Loop	Total available workforce	GDP	Poverty	Communit y Conflict Violence etc	Geographic al accessibility	Availability of other transport	# Patients taking other transport	# Patients in Definitive Care on other Transport	# Patients in Definitive Care	# Patients receiving Lower Quality Injury Care	Willingnes s to remain in care	# Patients remaining in care/reha b	# Patients Receiving Higher Quality Rehab						
347	14	Funding & System Capacity Loop	Total available workforce	GDP	System level funding	Available Funding for injury care	Availability of quality injury rehab or follow-up	Discriminatio n	# Patients Receiving Higher Quality Injury care	Patients Perceptio n of Health System	Patients trust in healthcare system	Willingnes s to receive care	# Patients in Definitive Care	# Patients remaining in care/reha b	# Patients Receiving Higher Quality Rehab						
348	14	Funding & System Capacity Loop	Total available workforce	GDP	System level funding	Available Funding for injury care	Availability of quality injury care	Discriminatio n	# Patients Receiving Higher Quality Injury care	Patients Perceptio n of Health System	Patients trust in healthcare system	# Injured seeking non-formal care	Willingnes s to seek formal care	# Injured seeking care	# Patients taking an ambulanc e						
349	14	Funding & System Capacity Loop	Total available workforce	GDP	Poverty	Individual wealth	Ability to pay	Willingness to receive care	# Patients in Definitive Care	# Patients Receiving Higher Quality Injury care	Patients Perceptio n of Health System	Patients trust in healthcare system	Willingnes s to seek formal care	# Injured seeking care	# Patients taking an ambulanc e						
350	14	Funding & System Capacity Loop	Total available workforce	GDP	Poverty	Individual wealth	Ability to pay	Willingness to receive care	# Patients in Definitive Care	# Patients receiving Lower Quality Injury Care	Patients Perceptio n of Health System	Patients trust in healthcare system	Willingnes s to remain in care	# Patients remaining in care/reha b	# Patients Receiving Higher Quality Rehab						
351	14	Funding & System Capacity Loop	Total available workforce	GDP	System level funding	Available Funding for injury care	Availability of quality injury care	Discriminatio n	# Patients receiving Lower Quality Injury Care	Patients Perceptio n of Health System	Patients trust in healthcare system	Willingnes s to receive care	# Patients in Definitive Care	# Patients remaining in care/reha b	# Patients Receiving Higher Quality Rehab						
352	14	Funding & System Capacity Loop	Total available workforce	GDP	Poverty	Communit y Conflict Violence etc	Availability of quality injury rehab or follow-up	Discriminatio n	# Patients receiving Lower Quality Injury Care	Patients Perceptio n of Health System	Patients trust in healthcare system	# Injured seeking non-formal care	Willingnes s to seek formal care	# Injured seeking care	# Patients taking an ambulanc e						
353	14	Funding & System Capacity Loop	Total available workforce	GDP	System level funding	Available Funding for injury care	Availability of quality injury care	# Patients Receiving Higher Quality Injury care	Patients Perceptio n of Health System	Patients trust in healthcare system	Willingnes s to seek formal care	# Injured seeking care	# Patients taking other transport	# Patients in Definitive Care on other Transport	# Patients in Definitive care on time						
354	14	Funding & System Capacity Loop	Total available workforce	GDP	System level funding	Available Funding for injury care	Availability of quality injury care	Discriminatio n	# Patients Receiving Higher Quality Injury care	Patients Perceptio n of Health System	Patients trust in healthcare system	Willingnes s to receive care	# Patients in Definitive Care	# Patients remaining in care/reha b	# Patients Receiving Higher Quality Rehab						
355	14	Funding & System Capacity Loop	Total available workforce	GDP	System level funding	Available Funding for injury care	Universal health insurance	Ability to pay	Willingnes s to seek formal care	# Injured seeking care	# Patients taking an ambulanc e	# Patients in Definitive Care on Ambulanc e	# Patients in Definitive Care	# Patients remaining in care/reha b	# Patients receiving Lower Quality Rehab						
356	14	Funding & System Capacity Loop	Total available workforce	GDP	Poverty	Communit y Conflict Violence etc	Geographic al accessibility	Availability of other transport	# Patients taking other transport	# Patients in Definitive Care on other Transport	# Patients in Definitive Care	# Patients receiving Lower Quality	Patients Perceptio n of Health System	Patients trust in healthcare system	# Injured seeking non-formal care						

												Injury Care									
357	14	Funding & System Capacity Loop	Total available workforce	GDP	System level funding	Available Funding for injury care	Availability of quality injury rehab or follow-up	Discriminatio n	# Patients Receiving Higher Quality Injury care	Patients Perceptio n of Health System	Patients trust in healthcare system	# Injured seeking non-formal care	Willingnes s to seek formal care	# Injured seeking care	# Patients taking an ambulanc e						
358	14	Funding & System Capacity Loop	Total available workforce	GDP	Poverty	Communit y Conflict Violence etc	Geographic al accessibility	Availability of other transport	# Patients taking other transport	# Patients in Definitive Care on other Transport	# Patients in Definitive Care	# Patients Receiving Higher Quality Injury care	Willingnes s to remain in care	# Patients remaining in care/reha b	# Patients receiving Lower Quality Rehab						
359	14	Funding & System Capacity Loop	Total available workforce	GDP	Poverty	Individual wealth	Ability to pay	Willingness to receive care	# Patients in Definitive Care	# Patients receiving Lower Quality Injury Care	Patients Perceptio n of Health System	Patients trust in healthcare system	# Injured seeking non-formal care	Willingnes s to seek formal care	# Injured seeking care						
360	14	Funding & System Capacity Loop	Total available workforce	GDP	System level funding	Available Funding for injury care	Availability of quality injury rehab or follow-up	Discriminatio n	# Patients receiving Lower Quality Injury Care	Patients Perceptio n of Health System	Patients trust in healthcare system	# Injured seeking non-formal care	Willingnes s to seek formal care	# Injured seeking care	# Patients taking an ambulanc e						
361	14	Funding & System Capacity Loop	Total available workforce	GDP	Poverty	Communit y Conflict Violence etc	Availability of quality injury care	Discriminatio n	# Patients receiving Lower Quality Injury Care	Patients Perceptio n of Health System	Patients trust in healthcare system	Willingnes s to receive care	# Patients in Definitive Care	# Patients remaining in care/reha b	# Patients Receiving Higher Quality Rehab						
362	14	Funding & System Capacity Loop	Total available workforce	GDP	System level funding	Available Funding for injury care	Availability of quality injury care	# Patients Receiving Higher Quality Injury care	Patients Perceptio n of Health System	Patients trust in healthcare system	Willingnes s to seek formal care	# Injured seeking care	# Patients taking an ambulanc e	# Patients in Definitive Care on Ambulanc e	# Patients in Definitive care on time						
363	14	Funding & System Capacity Loop	Total available workforce	GDP	Poverty	Individual wealth	Ability to pay	Willingness to receive care	# Patients in Definitive Care	# Patients receiving Lower Quality Injury Care	Patients Perceptio n of Health System	Patients trust in healthcare system	Willingnes s to remain in care	# Patients remaining in care/reha b	# Patients receiving Lower Quality Rehab						
364	14	Funding & System Capacity Loop	Total available workforce	GDP	Poverty	Communit y Conflict Violence etc	Availability of quality injury care	# Patients receiving Lower Quality Injury Care	Patients Perceptio n of Health System	Patients trust in healthcare system	Willingnes s to seek formal care	# Injured seeking care	# Patients taking other transport	# Patients in Definitive Care on other Transport	# Patients in Definitive care Delayed						
365	14	Funding & System Capacity Loop	Total available workforce	GDP	System level funding	Available Funding for injury care	Availability of quality injury care	Discriminatio n	# Patients Receiving Higher Quality Injury care	Patients Perceptio n of Health System	Patients trust in healthcare system	Willingnes s to receive care	# Patients in Definitive Care	# Patients remaining in care/reha b	# Patients receiving Lower Quality Rehab						
366	14	Funding & System Capacity Loop	Total available workforce	GDP	Poverty	Communit y Conflict Violence etc	Geographic al accessibility	Availability of Ambulance Service	# Patients taking an ambulanc e	# Patients in Definitive Care on Ambulanc e	# Patients in Definitive Care	# Patients receiving Lower Quality Injury Care	Willingnes s to remain in care	# Patients remaining in care/reha b	# Patients receiving Lower Quality Rehab						
367	14	Funding & System Capacity Loop	Total available workforce	GDP	Poverty	Communit y Conflict Violence etc	Availability of quality injury care	# Patients Receiving Higher Quality Injury care	Patients Perceptio n of Health System	Patients trust in healthcare system	Willingnes s to seek formal care	# Injured seeking care	# Patients taking other transport	# Patients in Definitive Care on other Transport	# Patients in Definitive care on time						
368	14	Funding & System Capacity Loop	Total available workforce	GDP	Poverty	Communit y Conflict Violence etc	Availability of quality injury care	# Patients receiving Lower Quality Injury Care	Patients Perceptio n of Health System	Patients trust in healthcare system	Willingnes s to seek formal care	# Injured seeking care	# Patients taking an ambulanc e	# Patients in Definitive Care on Ambulanc e	# Patients in Definitive care Delayed						
369	14	Funding & System Capacity Loop	Total available workforce	GDP	System level funding	Available Funding for injury care	Universal health insurance	Ability to pay	Willingnes s to receive care	# Patients in Definitive Care	# Patients Receiving Higher Quality Injury care	Patients Perceptio n of Health System	Patients trust in healthcare system	Willingnes s to seek formal care	# Injured seeking care						

370	14	Funding & System Capacity Loop	Total available workforce	GDP	System level funding	Available Funding for injury care	Universal health insurance	Ability to pay	Willingness to seek formal care	# Injured seeking care	# Patients taking an ambulance	# Patients in Definitive Care on Ambulance	# Patients in Definitive Care	# Patients remaining in care/rehab	# Patients Receiving Higher Quality Rehab						
371	14	Funding & System Capacity Loop	Total available workforce	GDP	Poverty	Community Conflict Violence etc	Availability of quality injury care	# Patients Receiving Higher Quality Injury care	Patients Perception of Health System	Patients trust in healthcare system	Willingness to seek formal care	# Injured seeking care	# Patients taking an ambulance	# Patients in Definitive Care on Ambulance	# Patients in Definitive care Delayed						
372	14	Funding & System Capacity Loop	Total available workforce	GDP	Poverty	Community Conflict Violence etc	Availability of quality injury care	Discrimination	# Patients Receiving Higher Quality Injury care	Patients Perception of Health System	Patients trust in healthcare system	# Injured seeking non-formal care	Willingness to seek formal care	# Injured seeking care	# Patients taking an ambulance						
373	14	Funding & System Capacity Loop	Total available workforce	GDP	Poverty	Community Conflict Violence etc	Availability of quality injury care	# Patients receiving Lower Quality Injury Care	Patients Perception of Health System	Patients trust in healthcare system	Willingness to seek formal care	# Injured seeking care	# Patients taking an ambulance	# Patients in Definitive Care on Ambulance	# Patients in Definitive care on time						
374	14	Funding & System Capacity Loop	Total available workforce	GDP	System level funding	Available Funding for injury care	Availability of quality injury care	# Patients receiving Lower Quality Injury Care	Patients Perception of Health System	Patients trust in healthcare system	Willingness to seek formal care	# Injured seeking care	# Patients taking an ambulance	# Patients in Definitive Care on Ambulance	# Patients in Definitive care Delayed						
375	14	Funding & System Capacity Loop	Total available workforce	GDP	Poverty	Community Conflict Violence etc	Geographical accessibility	Availability of Ambulance Service	# Patients taking an ambulance	# Patients in Definitive Care on Ambulance	# Patients in Definitive Care	# Patients receiving Lower Quality Injury Care	Willingness to remain in care	# Patients remaining in care/rehab	# Patients Receiving Higher Quality Rehab						
376	14	Funding & System Capacity Loop	Total available workforce	GDP	System level funding	Available Funding for injury care	Availability of quality injury rehab or follow-up	Discrimination	# Patients Receiving Higher Quality Injury care	Patients Perception of Health System	Patients trust in healthcare system	Willingness to receive care	# Patients in Definitive Care	# Patients remaining in care/rehab	# Patients receiving Lower Quality Rehab						
377	14	Funding & System Capacity Loop	Total available workforce	GDP	Poverty	Community Conflict Violence etc	Availability of quality injury care	Discrimination	# Patients receiving Lower Quality Injury Care	Patients Perception of Health System	Patients trust in healthcare system	# Injured seeking non-formal care	Willingness to seek formal care	# Injured seeking care	# Patients taking an ambulance						
378	14	Funding & System Capacity Loop	Total available workforce	GDP	System level funding	Available Funding for injury care	Availability of quality injury care	# Patients Receiving Higher Quality Injury care	Patients Perception of Health System	Patients trust in healthcare system	Willingness to seek formal care	# Injured seeking care	# Patients taking other transport	# Patients in Definitive Care on other Transport	# Patients in Definitive care Delayed						
379	14	Funding & System Capacity Loop	Total available workforce	GDP	Poverty	Community Conflict Violence etc	Availability of quality injury care	# Patients Receiving Higher Quality Injury care	Patients Perception of Health System	Patients trust in healthcare system	Willingness to seek formal care	# Injured seeking care	# Patients taking other transport	# Patients in Definitive Care on other Transport	# Patients in Definitive care Delayed						
380	14	Funding & System Capacity Loop	Total available workforce	GDP	Poverty	Community Conflict Violence etc	Availability of quality injury care	# Patients receiving Lower Quality Injury Care	Patients Perception of Health System	Patients trust in healthcare system	Willingness to seek formal care	# Injured seeking care	# Patients taking other transport	# Patients in Definitive Care on other Transport	# Patients in Definitive care on time						
381	14	Funding & System Capacity Loop	Total available workforce	GDP	System level funding	Available Funding for injury care	Availability of quality injury care	Discrimination	# Patients receiving Lower Quality Injury Care	Patients Perception of Health System	Patients trust in healthcare system	# Injured seeking non-formal care	Willingness to seek formal care	# Injured seeking care	# Patients taking an ambulance						
382	14	Funding & System Capacity Loop	Total available workforce	GDP	Poverty	Individual wealth	Availability of other transport	# Patients taking other transport	# Patients in Definitive Care on other Transport	# Patients in Definitive Care	# Patients receiving Lower Quality Injury Care	Patients Perception of Health System	Patients trust in healthcare system	Willingness to seek formal care	# Injured seeking care						
383	14	Funding & System Capacity Loop	Total available workforce	GDP	Poverty	Community Conflict Violence etc	Geographical accessibility	Availability of Ambulance Service	# Patients taking an ambulance	# Patients in Definitive Care on	# Patients in Definitive Care	# Patients Receiving Higher	Patients Perception of	Patients trust in healthcare system	# Injured seeking non-						

										Ambulance		Quality Injury care	Health System		formal care						
384	14	Funding & System Capacity Loop	Total available workforce	GDP	System level funding	Available Funding for injury care	Availability of quality injury care	# Patients Receiving Higher Quality Injury care	Patients Perception of Health System	Patients trust in healthcare system	Willingness to seek formal care	# Injured seeking care	# Patients taking an ambulance	# Patients in Definitive Care on Ambulance	# Patients in Definitive care Delayed						
385	15	Socioeconomic Determinant Loop	Total available workforce	GDP	System level funding	Available Funding for injury care	Availability of quality injury care	# Patients Receiving Higher Quality Injury care	Patients Perception of Health System	Patients trust in healthcare system	# Injured seeking non-formal care	Willingness to seek formal care	# Injured seeking care	# Patients taking other transport	# Patients in Definitive Care on other Transport	# Patients in Definitive care Delayed					
386	15	Socioeconomic Determinant Loop	Total available workforce	GDP	System level funding	Available Funding for injury care	Availability of Ambulance Service	# Patients taking an ambulance	# Patients in Definitive Care on Ambulance	# Patients in Definitive Care	# Patients receiving Lower Quality Injury Care	Patients Perception of Health System	Patients trust in healthcare system	Willingness to remain in care	# Patients remaining in care/rehab	# Patients receiving Lower Quality Rehab					
387	15	Socioeconomic Determinant Loop	Total available workforce	GDP	System level funding	Available Funding for injury care	Availability of quality injury care	# Patients receiving Lower Quality Injury Care	Patients Perception of Health System	Patients trust in healthcare system	# Injured seeking non-formal care	Willingness to seek formal care	# Injured seeking care	# Patients taking an ambulance	# Patients in Definitive Care on Ambulance	# Patients in Definitive care on time					
388	15	Socioeconomic Determinant Loop	Total available workforce	GDP	System level funding	Available Funding for injury care	Availability of quality injury care	# Patients Receiving Higher Quality Injury care	Patients Perception of Health System	Patients trust in healthcare system	# Injured seeking non-formal care	Willingness to seek formal care	# Injured seeking care	# Patients taking an ambulance	# Patients in Definitive Care on Ambulance	# Patients in Definitive care on time					
389	15	Socioeconomic Determinant Loop	Total available workforce	GDP	System level funding	Available Funding for injury care	Availability of quality injury care	# Patients receiving Lower Quality Injury Care	Patients Perception of Health System	Patients trust in healthcare system	# Injured seeking non-formal care	Willingness to seek formal care	# Injured seeking care	# Patients taking an ambulance	# Patients in Definitive Care on Ambulance	# Patients in Definitive care Delayed					
390	15	Socioeconomic Determinant Loop	Total available workforce	GDP	Poverty	Individual wealth	Availability of other transport	Geographical accessibility	Availability of Ambulance Service	# Patients taking an ambulance	# Patients in Definitive Care on Ambulance	# Patients in Definitive Care	# Patients receiving Lower Quality Injury Care	Willingness to remain in care	# Patients remaining in care/rehab	# Patients receiving Lower Quality Rehab					
391	15	Socioeconomic Determinant Loop	Total available workforce	GDP	Poverty	Community Conflict Violence etc	Availability of quality injury care	# Patients Receiving Higher Quality Injury care	Patients Perception of Health System	Patients trust in healthcare system	Willingness to receive care	# Patients in Definitive Care	# Patients receiving Lower Quality Injury Care	Willingness to remain in care	# Patients remaining in care/rehab	# Patients receiving Lower Quality Rehab					
392	15	Socioeconomic Determinant Loop	Total available workforce	GDP	Poverty	Individual wealth	Ability to pay	Willingness to seek formal care	# Injured seeking care	# Patients taking other transport	# Patients in Definitive Care on other Transport	# Patients in Definitive Care	# Patients receiving Lower Quality Injury Care	Patients Perception of Health System	Patients trust in healthcare system	# Injured seeking non-formal care					
393	15	Socioeconomic Determinant Loop	Total available workforce	GDP	System level funding	Available Funding for injury care	Availability of Ambulance Service	Geographical accessibility	Availability of other transport	# Patients taking other transport	# Patients in Definitive Care on other Transport	# Patients in Definitive Care	# Patients Receiving Higher Quality Injury care	Willingness to remain in care	# Patients remaining in care/rehab	# Patients receiving Lower Quality Rehab					
394	15	Socioeconomic Determinant Loop	Total available workforce	GDP	System level funding	Available Funding for injury care	Availability of quality injury care	Discrimination	# Patients receiving Lower Quality Injury Care	Patients Perception of Health System	Patients trust in healthcare system	Willingness to seek formal care	# Injured seeking care	# Patients taking an ambulance	# Patients in Definitive Care on Ambulance	# Patients in Definitive care on time					
395	15	Socioeconomic Determinant Loop	Total available workforce	GDP	System level funding	Available Funding for injury care	Availability of quality injury care	# Patients Receiving Higher Quality Injury care	Patients Perception of Health System	Patients trust in healthcare system	Willingness to seek formal care	# Injured seeking care	# Patients taking other transport	# Patients in Definitive Care on other Transport	# Patients in Definitive Care	# Patients receiving Lower Quality Injury Care					
396	15	Socioeconomic Determinant Loop	Total available workforce	GDP	System level funding	Available Funding for injury care	Availability of Ambulance Service	Geographical accessibility	Availability of other transport	# Patients taking other transport	# Patients in Definitive Care on other Transport	# Patients in Definitive Care	# Patients Receiving Higher Quality Injury care	Willingness to remain in care	# Patients remaining in care/rehab	# Patients Receiving Higher Quality Rehab					

397	15	Socioeconomi c Determinant Loop	Total available workforce	GDP	Poverty	Individual wealth	Availability of other transport	Geographica l accessibility	Availability of Ambulanc e Service	# Patients taking an ambulanc e	# Patients in Definitive Care on Ambulanc e	# Patients in Definitive Care	# Patients Receiving Higher Quality Injury care	Willingnes s to remain in care	# Patients remaining in care/reha b	# Patients Receiving Higher Quality Rehab					
398	15	Socioeconomi c Determinant Loop	Total available workforce	GDP	Poverty	Communit y Conflict Violence etc	Availability of quality injury care	# Patients Receiving Higher Quality Injury care	Patients Perceptio n of Health System	Patients trust in healthcare system	Willingnes s to seek formal care	# Injured seeking care	# Patients taking an ambulanc e	# Patients in Definitive Care on Ambulanc e	# Patients in Definitive Care	# Patients receiving Lower Quality Injury Care					
399	15	Socioeconomi c Determinant Loop	Total available workforce	GDP	System level funding	Available Funding for injury care	Availability of quality injury care	# Patients receiving Lower Quality Injury Care	Patients Perceptio n of Health System	Patients trust in healthcare system	Willingnes s to seek formal care	# Injured seeking care	# Patients taking other transport	# Patients in Definitive Care on other Transport	# Patients in Definitive Care	# Patients Receiving Higher Quality Injury care					
400	15	Socioeconomi c Determinant Loop	Total available workforce	GDP	System level funding	Available Funding for injury care	Availability of Ambulance Service	# Patients taking an ambulance	# Patients in Definitive Care on Ambulanc e	# Patients in Definitive Care	# Patients receiving Lower Quality Injury Care	Patients Perceptio n of Health System	Patients trust in healthcare system	Willingnes s to remain in care	# Patients remaining in care/reha b	# Patients Receiving Higher Quality Rehab					
401	15	Socioeconomi c Determinant Loop	Total available workforce	GDP	System level funding	Available Funding for injury care	Universal health insurance	Ability to pay	Willingnes s to receive care	# Patients in Definitive Care	# Patients receiving Lower Quality Injury Care	Patients Perceptio n of Health System	Patients trust in healthcare system	Willingnes s to remain in care	# Patients remaining in care/reha b	# Patients receiving Lower Quality Rehab					
402	15	Socioeconomi c Determinant Loop	Total available workforce	GDP	Poverty	Individual wealth	Ability to pay	Willingness to seek formal care	# Injured seeking care	# Patients taking other transport	# Patients in Definitive Care on other Transport	# Patients in Definitive Care	# Patients receiving Lower Quality Injury Care	Willingnes s to remain in care	# Patients remaining in care/reha b	# Patients Receiving Higher Quality Rehab					
403	15	Socioeconomi c Determinant Loop	Total available workforce	GDP	System level funding	Available Funding for injury care	Availability of quality injury care	# Patients receiving Lower Quality Injury Care	Patients Perceptio n of Health System	Patients trust in healthcare system	Willingnes s to seek formal care	# Injured seeking care	# Patients taking an ambulanc e	# Patients in Definitive Care on Ambulanc e	# Patients in Definitive Care	# Patients Receiving Higher Quality Injury care					
404	15	Socioeconomi c Determinant Loop	Total available workforce	GDP	System level funding	Available Funding for injury care	Universal health insurance	Ability to pay	Willingnes s to receive care	# Patients in Definitive Care	# Patients receiving Lower Quality Injury Care	Patients Perceptio n of Health System	Patients trust in healthcare system	Willingnes s to remain in care	# Patients remaining in care/reha b	# Patients Receiving Higher Quality Rehab					
405	15	Socioeconomi c Determinant Loop	Total available workforce	GDP	System level funding	Available Funding for injury care	Availability of quality injury care	# Patients receiving Lower Quality Injury Care	Patients Perceptio n of Health System	Patients trust in healthcare system	# Injured seeking non-formal care	Willingnes s to seek formal care	# Injured seeking care	# Patients taking other transport	# Patients in Definitive Care on other Transport	# Patients in Definitive care Delayed					
406	15	Socioeconomi c Determinant Loop	Total available workforce	GDP	Poverty	Communit y Conflict Violence etc	Availability of quality injury care	# Patients Receiving Higher Quality Injury care	Patients Perceptio n of Health System	Patients trust in healthcare system	Willingnes s to receive care	# Patients in Definitive Care	# Patients receiving Lower Quality Injury Care	Willingnes s to remain in care	# Patients remaining in care/reha b	# Patients Receiving Higher Quality Rehab					
407	15	Socioeconomi c Determinant Loop	Total available workforce	GDP	System level funding	Available Funding for injury care	Availability of Ambulance Service	# Patients taking an ambulance	# Patients in Definitive Care on Ambulanc e	# Patients in Definitive Care	# Patients Receiving Higher Quality Injury care	Patients Perceptio n of Health System	Patients trust in healthcare system	Willingnes s to remain in care	# Patients remaining in care/reha b	# Patients Receiving Higher Quality Rehab					
408	15	Socioeconomi c Determinant Loop	Total available workforce	GDP	System level funding	Available Funding for injury care	Availability of Ambulance Service	# Patients taking an ambulance	# Patients in Definitive Care on Ambulanc e	# Patients in Definitive Care	# Patients Receiving Higher Quality Injury care	Patients Perceptio n of Health System	Patients trust in healthcare system	Willingnes s to remain in care	# Patients remaining in care/reha b	# Patients receiving Lower Quality Rehab					
409	15	Socioeconomi c Determinant Loop	Total available workforce	GDP	Poverty	Individual wealth	Ability to pay	Willingness to seek formal care	# Injured seeking care	# Patients taking an ambulanc e	# Patients in Definitive Care on Ambulanc e	# Patients in Definitive Care	# Patients Receiving Higher Quality Injury care	Willingnes s to remain in care	# Patients remaining in care/reha b	# Patients receiving Lower Quality Rehab					
410	15	Socioeconomi c Determinant Loop	Total available workforce	GDP	System	Available Funding	Availability	# Patients receiving	Patients Perceptio	Patients trust in	Willingnes s to	# Patients in	# Patients Receiving	Willingnes s to	# Patients remaining	# Patients Receiving					

					level funding	for injury care	of quality injury care	Lower Quality Injury Care	n of Health System	healthcare system	receive care	Definitive Care	Higher Quality Injury care	remain in care	in care/rehab	Higher Quality Rehab					
411	15	Socioeconomic Determinant Loop	Total available workforce	GDP	Poverty	Individual wealth	Availability of other transport	# Patients taking other transport	# Patients in Definitive Care on other Transport	# Patients in Definitive Care	# Patients Receiving Higher Quality Injury care	Patients Perception of Health System	Patients trust in healthcare system	Willingness to remain in care	# Patients remaining in care/rehab	# Patients receiving Lower Quality Rehab					
412	15	Socioeconomic Determinant Loop	Total available workforce	GDP	System level funding	Available Funding for injury care	Availability of quality injury care	Discrimination	# Patients receiving Lower Quality Injury Care	Patients Perception of Health System	Patients trust in healthcare system	Willingness to seek formal care	# Injured seeking care	# Patients taking other transport	# Patients in Definitive Care on other Transport	# Patients in Definitive care on time					
413	15	Socioeconomic Determinant Loop	Total available workforce	GDP	System level funding	Available Funding for injury care	Availability of quality injury care	Discrimination	# Patients receiving Lower Quality Injury Care	Patients Perception of Health System	Patients trust in healthcare system	Willingness to seek formal care	# Injured seeking care	# Patients taking other transport	# Patients in Definitive Care on other Transport	# Patients in Definitive care Delayed					
414	15	Socioeconomic Determinant Loop	Total available workforce	GDP	Poverty	Community Conflict Violence etc	Availability of quality injury care	# Patients receiving Lower Quality Injury Care	Patients Perception of Health System	Patients trust in healthcare system	# Injured seeking non-formal care	Willingness to seek formal care	# Injured seeking care	# Patients taking other transport	# Patients in Definitive Care on other Transport	# Patients in Definitive care Delayed					
415	15	Socioeconomic Determinant Loop	Total available workforce	GDP	Poverty	Individual wealth	Ability to pay	Willingness to seek formal care	# Injured seeking care	# Patients taking other transport	# Patients in Definitive Care on other Transport	# Patients in Definitive Care	# Patients receiving Lower Quality Injury Care	Willingness to remain in care	# Patients remaining in care/rehab	# Patients receiving Lower Quality Rehab					
416	15	Socioeconomic Determinant Loop	Total available workforce	GDP	Poverty	Community Conflict Violence etc	Availability of quality injury rehab or follow-up	Discrimination	# Patients Receiving Higher Quality Injury care	Patients Perception of Health System	Patients trust in healthcare system	Willingness to seek formal care	# Injured seeking care	# Patients taking other transport	# Patients in Definitive Care on other Transport	# Patients in Definitive care on time					
417	15	Socioeconomic Determinant Loop	Total available workforce	GDP	System level funding	Available Funding for injury care	Availability of quality injury care	# Patients Receiving Higher Quality Injury care	Patients Perception of Health System	Patients trust in healthcare system	# Injured seeking non-formal care	Willingness to seek formal care	# Injured seeking care	# Patients taking other transport	# Patients in Definitive Care on other Transport	# Patients in Definitive care on time					
418	15	Socioeconomic Determinant Loop	Total available workforce	GDP	System level funding	Available Funding for injury care	Availability of quality injury care	# Patients Receiving Higher Quality Injury care	Patients Perception of Health System	Patients trust in healthcare system	Willingness to seek formal care	# Injured seeking care	# Patients taking an ambulance	# Patients in Definitive Care on Ambulance	# Patients in Definitive Care	# Patients receiving Lower Quality Injury Care					
419	15	Socioeconomic Determinant Loop	Total available workforce	GDP	Poverty	Individual wealth	Availability of other transport	Geographical accessibility	Availability of Ambulance Service	# Patients taking an ambulance	# Patients in Definitive Care on Ambulance	# Patients in Definitive Care	# Patients Receiving Higher Quality Injury care	Patients Perception of Health System	Patients trust in healthcare system	# Injured seeking non-formal care					
420	15	Socioeconomic Determinant Loop	Total available workforce	GDP	Poverty	Community Conflict Violence etc	Geographical accessibility	Availability of other transport	# Patients taking other transport	# Patients in Definitive Care on other Transport	# Patients in Definitive Care	# Patients Receiving Higher Quality Injury care	Patients Perception of Health System	Patients trust in healthcare system	Willingness to seek formal care	# Injured seeking care					
421	15	Socioeconomic Determinant Loop	Total available workforce	GDP	Poverty	Individual wealth	Ability to pay	Willingness to seek formal care	# Injured seeking care	# Patients taking other transport	# Patients in Definitive Care on other Transport	# Patients in Definitive Care	# Patients Receiving Higher Quality Injury care	Willingness to remain in care	# Patients remaining in care/rehab	# Patients receiving Lower Quality Rehab					
422	15	Socioeconomic Determinant Loop	Total available workforce	GDP	Poverty	Community Conflict Violence etc	Availability of quality injury care	Discrimination	# Patients Receiving Higher Quality Injury care	Patients Perception of Health System	Patients trust in healthcare system	Willingness to seek formal care	# Injured seeking care	# Patients taking other transport	# Patients in Definitive Care on other Transport	# Patients in Definitive care Delayed					
423	15	Socioeconomic Determinant Loop	Total available workforce	GDP	System level funding	Available Funding for injury care	Availability of quality injury care	# Patients receiving Lower Quality Injury Care	Patients Perception of Health System	Patients trust in healthcare system	Willingness to receive care	# Patients in Definitive Care	# Patients Receiving Higher Quality Injury care	Willingness to remain in care	# Patients remaining in care/rehab	# Patients receiving Lower Quality Rehab					

424	15	Socioeconomic Determinant Loop	Total available workforce	GDP	System level funding	Available Funding for injury care	Availability of quality injury care	# Patients Receiving Higher Quality Injury care	Patients Perception of Health System	Patients trust in healthcare system	Willingness to receive care	# Patients in Definitive Care	# Patients receiving Lower Quality Injury Care	Willingness to remain in care	# Patients remaining in care/rehab	# Patients Receiving Higher Quality Rehab				
425	15	Socioeconomic Determinant Loop	Total available workforce	GDP	Poverty	Individual wealth	Ability to pay	Willingness to seek formal care	# Injured seeking care	# Patients taking an ambulance	# Patients in Definitive Care on Ambulance	# Patients in Definitive Care	# Patients Receiving Higher Quality Injury care	Willingness to remain in care	# Patients remaining in care/rehab	# Patients Receiving Higher Quality Rehab				
426	15	Socioeconomic Determinant Loop	Total available workforce	GDP	Poverty	Individual wealth	Ability to pay	Willingness to seek formal care	# Injured seeking care	# Patients taking other transport	# Patients in Definitive Care on other Transport	# Patients in Definitive Care	# Patients Receiving Higher Quality Injury care	Patients Perception of Health System	Patients trust in healthcare system	# Injured seeking non-formal care				
427	15	Socioeconomic Determinant Loop	Total available workforce	GDP	System level funding	Available Funding for injury care	Availability of quality injury care	# Patients Receiving Higher Quality Injury care	Patients Perception of Health System	Patients trust in healthcare system	# Injured seeking non-formal care	Willingness to seek formal care	# Injured seeking care	# Patients taking an ambulance	# Patients in Definitive Care on Ambulance	# Patients in Definitive care Delayed				
428	15	Socioeconomic Determinant Loop	Total available workforce	GDP	System level funding	Available Funding for injury care	Availability of Ambulance Service	# Patients taking an ambulance	# Patients in Definitive Care on Ambulance	# Patients in Definitive Care	# Patients Receiving Higher Quality Injury care	Patients Perception of Health System	Patients trust in healthcare system	# Injured seeking non-formal care	Willingness to seek formal care	# Injured seeking care				
429	15	Socioeconomic Determinant Loop	Total available workforce	GDP	System level funding	Available Funding for injury care	Universal health insurance	Ability to pay	Willingness to receive care	# Patients in Definitive Care	# Patients Receiving Higher Quality Injury care	Patients Perception of Health System	Patients trust in healthcare system	# Injured seeking non-formal care	Willingness to seek formal care	# Injured seeking care				
430	15	Socioeconomic Determinant Loop	Total available workforce	GDP	Poverty	Community Conflict Violence etc	Availability of quality injury care	Discrimination	# Patients Receiving Higher Quality Injury care	Patients Perception of Health System	Patients trust in healthcare system	Willingness to seek formal care	# Injured seeking care	# Patients taking an ambulance	# Patients in Definitive Care on Ambulance	# Patients in Definitive care Delayed				
431	15	Socioeconomic Determinant Loop	Total available workforce	GDP	System level funding	Available Funding for injury care	Availability of Ambulance Service	Geographical accessibility	Availability of other transport	# Patients taking other transport	# Patients in Definitive Care on other Transport	# Patients in Definitive Care	# Patients receiving Lower Quality Injury Care	Patients Perception of Health System	Patients trust in healthcare system	# Injured seeking non-formal care				
432	15	Socioeconomic Determinant Loop	Total available workforce	GDP	Poverty	Individual wealth	Availability of other transport	# Patients taking other transport	# Patients in Definitive Care on other Transport	# Patients in Definitive Care	# Patients receiving Lower Quality Injury Care	Patients Perception of Health System	Patients trust in healthcare system	# Injured seeking non-formal care	Willingness to seek formal care	# Injured seeking care				
433	15	Socioeconomic Determinant Loop	Total available workforce	GDP	Poverty	Individual wealth	Availability of other transport	# Patients taking other transport	# Patients in Definitive Care on other Transport	# Patients in Definitive Care	# Patients Receiving Higher Quality Injury care	Patients Perception of Health System	Patients trust in healthcare system	# Injured seeking non-formal care	Willingness to seek formal care	# Injured seeking care				
434	15	Socioeconomic Determinant Loop	Total available workforce	GDP	Poverty	Individual wealth	Ability to pay	Willingness to seek formal care	# Injured seeking care	# Patients taking an ambulance	# Patients in Definitive Care on Ambulance	# Patients in Definitive Care	# Patients receiving Lower Quality Injury Care	Willingness to remain in care	# Patients remaining in care/rehab	# Patients Receiving Higher Quality Rehab				
435	15	Socioeconomic Determinant Loop	Total available workforce	GDP	Poverty	Community Conflict Violence etc	Availability of quality injury care	# Patients receiving Lower Quality Injury Care	Patients Perception of Health System	Patients trust in healthcare system	# Injured seeking non-formal care	Willingness to seek formal care	# Injured seeking care	# Patients taking an ambulance	# Patients in Definitive Care on Ambulance	# Patients in Definitive care on time				
436	15	Socioeconomic Determinant Loop	Total available workforce	GDP	System level funding	Available Funding for injury care	Availability of quality injury rehab or follow-up	Discrimination	# Patients Receiving Higher Quality Injury care	Patients Perception of Health System	Patients trust in healthcare system	Willingness to seek formal care	# Injured seeking care	# Patients taking other transport	# Patients in Definitive Care on other Transport	# Patients in Definitive care on time				
437	15	Socioeconomic Determinant Loop	Total available workforce	GDP	System level funding	Available Funding for injury care	Availability of quality injury rehab or follow-up	Discrimination	# Patients Receiving Higher	Patients Perception of	Patients trust in healthcare system	Willingness to seek formal care	# Injured seeking care	# Patients taking other transport	# Patients in Definitive Care on	# Patients in Definitive care Delayed				

									Quality Injury care	Health System					other Transport						
438	15	Socioeconomic Determinant Loop	Total available workforce	GDP	Poverty	Community Conflict Violence etc	Availability of quality injury rehab or follow-up	Discrimination	# Patients receiving Lower Quality Injury Care	Patients Perception of Health System	Patients trust in healthcare system	Willingness to seek formal care	# Injured seeking care	# Patients taking an ambulance	# Patients in Definitive Care on Ambulance	# Patients in Definitive care on time					
439	15	Socioeconomic Determinant Loop	Total available workforce	GDP	Poverty	Community Conflict Violence etc	Availability of quality injury rehab or follow-up	Discrimination	# Patients receiving Lower Quality Injury Care	Patients Perception of Health System	Patients trust in healthcare system	Willingness to seek formal care	# Injured seeking care	# Patients taking an ambulance	# Patients in Definitive Care on Ambulance	# Patients in Definitive care Delayed					
440	15	Socioeconomic Determinant Loop	Total available workforce	GDP	Poverty	Community Conflict Violence etc	Availability of quality injury rehab or follow-up	Discrimination	# Patients Receiving Higher Quality Injury care	Patients Perception of Health System	Patients trust in healthcare system	Willingness to seek formal care	# Injured seeking care	# Patients taking an ambulance	# Patients in Definitive Care on Ambulance	# Patients in Definitive care Delayed					
441	15	Socioeconomic Determinant Loop	Total available workforce	GDP	Poverty	Individual wealth	Availability of other transport	# Patients taking other transport	# Patients in Definitive Care on other Transport	# Patients in Definitive Care	# Patients receiving Lower Quality Injury Care	Patients Perception of Health System	Patients trust in healthcare system	Willingness to remain in care	# Patients remaining in care/rehab	# Patients receiving Lower Quality Rehab					
442	15	Socioeconomic Determinant Loop	Total available workforce	GDP	Poverty	Individual wealth	Availability of other transport	# Patients taking other transport	# Patients in Definitive Care on other Transport	# Patients in Definitive Care	# Patients receiving Lower Quality Injury Care	Patients Perception of Health System	Patients trust in healthcare system	Willingness to remain in care	# Patients remaining in care/rehab	# Patients Receiving Higher Quality Rehab					
443	15	Socioeconomic Determinant Loop	Total available workforce	GDP	Poverty	Individual wealth	Ability to pay	Willingness to seek formal care	# Injured seeking care	# Patients taking an ambulance	# Patients in Definitive Care on Ambulance	# Patients in Definitive Care	# Patients Receiving Higher Quality Injury care	Patients Perception of Health System	Patients trust in healthcare system	# Injured seeking non-formal care					
444	15	Socioeconomic Determinant Loop	Total available workforce	GDP	Poverty	Community Conflict Violence etc	Availability of quality injury care	Discrimination	# Patients Receiving Higher Quality Injury care	Patients Perception of Health System	Patients trust in healthcare system	Willingness to seek formal care	# Injured seeking care	# Patients taking an ambulance	# Patients in Definitive Care on Ambulance	# Patients in Definitive care on time					
445	15	Socioeconomic Determinant Loop	Total available workforce	GDP	System level funding	Available Funding for injury care	Availability of Ambulance Service	Geographical accessibility	Availability of other transport	# Patients taking other transport	# Patients in Definitive Care on other Transport	# Patients in Definitive Care	# Patients Receiving Higher Quality Injury care	Patients Perception of Health System	Patients trust in healthcare system	# Injured seeking non-formal care					
446	15	Socioeconomic Determinant Loop	Total available workforce	GDP	Poverty	Community Conflict Violence etc	Availability of quality injury rehab or follow-up	Discrimination	# Patients receiving Lower Quality Injury Care	Patients Perception of Health System	Patients trust in healthcare system	Willingness to seek formal care	# Injured seeking care	# Patients taking other transport	# Patients in Definitive Care on other Transport	# Patients in Definitive care Delayed					
447	15	Socioeconomic Determinant Loop	Total available workforce	GDP	System level funding	Available Funding for injury care	Availability of Ambulance Service	# Patients taking an ambulance	# Patients in Definitive Care on Ambulance	# Patients in Definitive Care	# Patients receiving Lower Quality Injury Care	Patients Perception of Health System	Patients trust in healthcare system	# Injured seeking non-formal care	Willingness to seek formal care	# Injured seeking care					
448	15	Socioeconomic Determinant Loop	Total available workforce	GDP	System level funding	Available Funding for injury care	Availability of quality injury care	# Patients receiving Lower Quality Injury Care	Patients Perception of Health System	Patients trust in healthcare system	# Injured seeking non-formal care	Willingness to seek formal care	# Injured seeking care	# Patients taking other transport	# Patients in Definitive Care on other Transport	# Patients in Definitive care on time					
449	15	Socioeconomic Determinant Loop	Total available workforce	GDP	Poverty	Community Conflict Violence etc	Availability of quality injury care	# Patients receiving Lower Quality Injury Care	Patients Perception of Health System	Patients trust in healthcare system	Willingness to seek formal care	# Injured seeking care	# Patients taking other transport	# Patients in Definitive Care on other Transport	# Patients in Definitive Care	# Patients Receiving Higher Quality Injury care					
450	15	Socioeconomic Determinant Loop	Total available workforce	GDP	Poverty	Community Conflict Violence etc	Availability of quality injury care	Discrimination	# Patients Receiving Higher Quality Injury care	Patients Perception of Health System	Patients trust in healthcare system	Willingness to seek formal care	# Injured seeking care	# Patients taking other transport	# Patients in Definitive Care on other Transport	# Patients in Definitive care on time					

451	15	Socioeconomic Determinant Loop	Total available workforce	GDP	System level funding	Available Funding for injury care	Availability of quality injury rehab or follow-up	Discrimination	# Patients receiving Lower Quality Injury Care	Patients Perception of Health System	Patients trust in healthcare system	Willingness to seek formal care	# Injured seeking care	# Patients taking other transport	# Patients in Definitive Care on other Transport	# Patients in Definitive care Delayed					
452	15	Socioeconomic Determinant Loop	Total available workforce	GDP	System level funding	Available Funding for injury care	Universal health insurance	Ability to pay	Willingness to receive care	# Patients in Definitive Care	# Patients receiving Lower Quality Injury Care	Patients Perception of Health System	Patients trust in healthcare system	Willingness to seek formal care	# Injured seeking care	# Patients taking an ambulance					
453	15	Socioeconomic Determinant Loop	Total available workforce	GDP	Poverty	Community Conflict Violence etc	Availability of quality injury rehab or follow-up	Discrimination	# Patients Receiving Higher Quality Injury care	Patients Perception of Health System	Patients trust in healthcare system	Willingness to seek formal care	# Injured seeking care	# Patients taking an ambulance	# Patients in Definitive Care on Ambulance	# Patients in Definitive care on time					
454	15	Socioeconomic Determinant Loop	Total available workforce	GDP	Poverty	Community Conflict Violence etc	Geographical accessibility	Availability of Ambulance Service	# Patients taking an ambulance	# Patients in Definitive Care on Ambulance	# Patients in Definitive Care	# Patients receiving Lower Quality Injury Care	Patients Perception of Health System	Patients trust in healthcare system	Willingness to seek formal care	# Injured seeking care					
455	15	Socioeconomic Determinant Loop	Total available workforce	GDP	System level funding	Available Funding for injury care	Universal health insurance	Ability to pay	Willingness to receive care	# Patients in Definitive Care	# Patients receiving Lower Quality Injury Care	Patients Perception of Health System	Patients trust in healthcare system	# Injured seeking non-formal care	Willingness to seek formal care	# Injured seeking care					
456	15	Socioeconomic Determinant Loop	Total available workforce	GDP	System level funding	Available Funding for injury care	Availability of quality injury care	Discrimination	# Patients Receiving Higher Quality Injury care	Patients Perception of Health System	Patients trust in healthcare system	Willingness to seek formal care	# Injured seeking care	# Patients taking other transport	# Patients in Definitive Care on other Transport	# Patients in Definitive care on time					
457	15	Socioeconomic Determinant Loop	Total available workforce	GDP	Poverty	Community Conflict Violence etc	Availability of quality injury care	# Patients Receiving Higher Quality Injury care	Patients Perception of Health System	Patients trust in healthcare system	# Injured seeking non-formal care	Willingness to seek formal care	# Injured seeking care	# Patients taking other transport	# Patients in Definitive Care on other Transport	# Patients in Definitive care Delayed					
458	15	Socioeconomic Determinant Loop	Total available workforce	GDP	Poverty	Community Conflict Violence etc	Availability of quality injury care	Discrimination	# Patients receiving Lower Quality Injury Care	Patients Perception of Health System	Patients trust in healthcare system	Willingness to seek formal care	# Injured seeking care	# Patients taking an ambulance	# Patients in Definitive Care on Ambulance	# Patients in Definitive care on time					
459	15	Socioeconomic Determinant Loop	Total available workforce	GDP	Poverty	Community Conflict Violence etc	Availability of quality injury rehab or follow-up	Discrimination	# Patients receiving Lower Quality Injury Care	Patients Perception of Health System	Patients trust in healthcare system	Willingness to seek formal care	# Injured seeking care	# Patients taking other transport	# Patients in Definitive Care on other Transport	# Patients in Definitive care on time					
460	15	Socioeconomic Determinant Loop	Total available workforce	GDP	System level funding	Available Funding for injury care	Availability of quality injury rehab or follow-up	Discrimination	# Patients Receiving Higher Quality Injury care	Patients Perception of Health System	Patients trust in healthcare system	Willingness to seek formal care	# Injured seeking care	# Patients taking an ambulance	# Patients in Definitive Care on Ambulance	# Patients in Definitive care Delayed					
461	15	Socioeconomic Determinant Loop	Total available workforce	GDP	Poverty	Community Conflict Violence etc	Availability of quality injury care	Discrimination	# Patients receiving Lower Quality Injury Care	Patients Perception of Health System	Patients trust in healthcare system	Willingness to seek formal care	# Injured seeking care	# Patients taking an ambulance	# Patients in Definitive Care on Ambulance	# Patients in Definitive care Delayed					
462	15	Socioeconomic Determinant Loop	Total available workforce	GDP	Poverty	Community Conflict Violence etc	Availability of quality injury rehab or follow-up	Discrimination	# Patients Receiving Higher Quality Injury care	Patients Perception of Health System	Patients trust in healthcare system	Willingness to seek formal care	# Injured seeking care	# Patients taking other transport	# Patients in Definitive Care on other Transport	# Patients in Definitive care Delayed					
463	15	Socioeconomic Determinant Loop	Total available workforce	GDP	System level funding	Available Funding for injury care	Availability of Ambulance Service	Geographical accessibility	Availability of other transport	# Patients taking other transport	# Patients in Definitive Care on other Transport	# Patients in Definitive Care	# Patients receiving Lower Quality Injury Care	Willingness to remain in care	# Patients remaining in care/rehab	# Patients receiving Lower Quality Rehab					

464	15	Socioeconomic Determinant Loop	Total available workforce	GDP	Poverty	Community Conflict Violence etc	Availability of quality injury care	Discrimination	# Patients receiving Lower Quality Injury Care	Patients Perception of Health System	Patients trust in healthcare system	Willingness to seek formal care	# Injured seeking care	# Patients taking other transport	# Patients in Definitive Care on other Transport	# Patients in Definitive care on time					
465	15	Socioeconomic Determinant Loop	Total available workforce	GDP	Poverty	Community Conflict Violence etc	Availability of quality injury care	Discrimination	# Patients receiving Lower Quality Injury Care	Patients Perception of Health System	Patients trust in healthcare system	Willingness to seek formal care	# Injured seeking care	# Patients taking other transport	# Patients in Definitive Care on other Transport	# Patients in Definitive care Delayed					
466	15	Socioeconomic Determinant Loop	Total available workforce	GDP	System level funding	Available Funding for injury care	Universal health insurance	Ability to pay	Willingness to receive care	# Patients in Definitive Care	# Patients Receiving Higher Quality Injury care	Patients Perception of Health System	Patients trust in healthcare system	Willingness to remain in care	# Patients remaining in care/rehab	# Patients Receiving Higher Quality Rehab					
467	15	Socioeconomic Determinant Loop	Total available workforce	GDP	Poverty	Individual wealth	Ability to pay	Willingness to seek formal care	# Injured seeking care	# Patients taking other transport	# Patients in Definitive Care on other Transport	# Patients in Definitive Care	# Patients Receiving Higher Quality Injury care	Willingness to remain in care	# Patients remaining in care/rehab	# Patients Receiving Higher Quality Rehab					
468	15	Socioeconomic Determinant Loop	Total available workforce	GDP	System level funding	Available Funding for injury care	Availability of quality injury care	# Patients Receiving Higher Quality Injury care	Patients Perception of Health System	Patients trust in healthcare system	Willingness to receive care	# Patients in Definitive Care	# Patients receiving Lower Quality Injury Care	Willingness to remain in care	# Patients remaining in care/rehab	# Patients receiving Lower Quality Rehab					
469	15	Socioeconomic Determinant Loop	Total available workforce	GDP	System level funding	Available Funding for injury care	Availability of quality injury care	Discrimination	# Patients Receiving Higher Quality Injury care	Patients Perception of Health System	Patients trust in healthcare system	Willingness to seek formal care	# Injured seeking care	# Patients taking an ambulance	# Patients in Definitive Care on Ambulance	# Patients in Definitive care on time					
470	15	Socioeconomic Determinant Loop	Total available workforce	GDP	System level funding	Available Funding for injury care	Universal health insurance	Ability to pay	Willingness to receive care	# Patients in Definitive Care	# Patients Receiving Higher Quality Injury care	Patients Perception of Health System	Patients trust in healthcare system	Willingness to seek formal care	# Injured seeking care	# Patients taking an ambulance					
471	15	Socioeconomic Determinant Loop	Total available workforce	GDP	Poverty	Community Conflict Violence etc	Availability of quality injury care	# Patients receiving Lower Quality Injury Care	Patients Perception of Health System	Patients trust in healthcare system	# Injured seeking non-formal care	Willingness to seek formal care	# Injured seeking care	# Patients taking other transport	# Patients in Definitive Care on other Transport	# Patients in Definitive care on time					
472	15	Socioeconomic Determinant Loop	Total available workforce	GDP	Poverty	Community Conflict Violence etc	Availability of quality injury care	# Patients receiving Lower Quality Injury Care	Patients Perception of Health System	Patients trust in healthcare system	Willingness to seek formal care	# Injured seeking care	# Patients taking an ambulance	# Patients in Definitive Care on Ambulance	# Patients in Definitive Care	# Patients Receiving Higher Quality Injury care					
473	15	Socioeconomic Determinant Loop	Total available workforce	GDP	System level funding	Available Funding for injury care	Availability of quality injury rehab or follow-up	Discrimination	# Patients receiving Lower Quality Injury Care	Patients Perception of Health System	Patients trust in healthcare system	Willingness to seek formal care	# Injured seeking care	# Patients taking an ambulance	# Patients in Definitive Care on Ambulance	# Patients in Definitive care on time					
474	15	Socioeconomic Determinant Loop	Total available workforce	GDP	System level funding	Available Funding for injury care	Availability of quality injury rehab or follow-up	Discrimination	# Patients receiving Lower Quality Injury Care	Patients Perception of Health System	Patients trust in healthcare system	Willingness to seek formal care	# Injured seeking care	# Patients taking an ambulance	# Patients in Definitive Care on Ambulance	# Patients in Definitive care Delayed					
475	15	Socioeconomic Determinant Loop	Total available workforce	GDP	Poverty	Individual wealth	Availability of other transport	Geographical accessibility	Availability of Ambulance Service	# Patients taking an ambulance	# Patients in Definitive Care on Ambulance	# Patients in Definitive Care	# Patients Receiving Higher Quality Injury care	Willingness to remain in care	# Patients remaining in care/rehab	# Patients receiving Lower Quality Rehab					
476	15	Socioeconomic Determinant Loop	Total available workforce	GDP	Poverty	Community Conflict Violence etc	Availability of quality injury care	# Patients Receiving Higher Quality Injury care	Patients Perception of Health System	Patients trust in healthcare system	# Injured seeking non-formal care	Willingness to seek formal care	# Injured seeking care	# Patients taking an ambulance	# Patients in Definitive Care on Ambulance	# Patients in Definitive care Delayed					
477	15	Socioeconomic Determinant Loop	Total available workforce	GDP	Poverty	Individual wealth	Ability to pay	Willingness to seek formal care	# Injured seeking care	# Patients taking an	# Patients in Definitive Care on	# Patients in Definitive Care	# Patients receiving Lower	Willingness to	# Patients remaining in	# Patients receiving Lower					

										ambulance	Ambulance		Quality Injury Care	remain in care	care/rehab	Quality Rehab					
478	15	Socioeconomic Determinant Loop	Total available workforce	GDP	Poverty	Community Conflict Violence etc	Availability of quality injury care	# Patients Receiving Higher Quality Injury care	Patients Perception of Health System	Patients trust in healthcare system	Willingness to seek formal care	# Injured seeking care	# Patients taking other transport	# Patients in Definitive Care on other Transport	# Patients in Definitive Care	# Patients receiving Lower Quality Injury Care					
479	15	Socioeconomic Determinant Loop	Total available workforce	GDP	Poverty	Individual wealth	Ability to pay	Willingness to receive care	# Patients in Definitive Care	# Patients Receiving Higher Quality Injury care	Patients Perception of Health System	Patients trust in healthcare system	# Injured seeking non-formal care	Willingness to seek formal care	# Injured seeking care	# Patients taking an ambulance					
480	15	Socioeconomic Determinant Loop	Total available workforce	GDP	Poverty	Community Conflict Violence etc	Availability of quality injury care	# Patients receiving Lower Quality Injury Care	Patients Perception of Health System	Patients trust in healthcare system	# Injured seeking non-formal care	Willingness to seek formal care	# Injured seeking care	# Patients taking an ambulance	# Patients in Definitive Care on Ambulance	# Patients in Definitive care Delayed					
481	15	Socioeconomic Determinant Loop	Total available workforce	GDP	Poverty	Individual wealth	Ability to pay	Willingness to seek formal care	# Injured seeking care	# Patients taking an ambulance	# Patients in Definitive Care on Ambulance	# Patients in Definitive Care	# Patients receiving Lower Quality Injury Care	Patients Perception of Health System	Patients trust in healthcare system	# Injured seeking non-formal care					
482	15	Socioeconomic Determinant Loop	Total available workforce	GDP	Poverty	Individual wealth	Availability of other transport	# Patients taking other transport	# Patients in Definitive Care on other Transport	# Patients in Definitive Care	# Patients Receiving Higher Quality Injury care	Patients Perception of Health System	Patients trust in healthcare system	Willingness to remain in care	# Patients remaining in care/rehab	# Patients Receiving Higher Quality Rehab					
483	15	Socioeconomic Determinant Loop	Total available workforce	GDP	Poverty	Community Conflict Violence etc	Availability of quality injury care	# Patients Receiving Higher Quality Injury care	Patients Perception of Health System	Patients trust in healthcare system	# Injured seeking non-formal care	Willingness to seek formal care	# Injured seeking care	# Patients taking other transport	# Patients in Definitive Care on other Transport	# Patients in Definitive care on time					
484	15	Socioeconomic Determinant Loop	Total available workforce	GDP	Poverty	Individual wealth	Availability of other transport	# Patients taking other transport	# Patients in Definitive Care on other Transport	# Patients in Definitive Care	# Patients Receiving Higher Quality Injury care	Patients Perception of Health System	Patients trust in healthcare system	Willingness to seek formal care	# Injured seeking care	# Patients taking an ambulance					
485	15	Socioeconomic Determinant Loop	Total available workforce	GDP	System level funding	Available Funding for injury care	Availability of quality injury rehab or follow-up	Discrimination	# Patients Receiving Higher Quality Injury care	Patients Perception of Health System	Patients trust in healthcare system	Willingness to seek formal care	# Injured seeking care	# Patients taking an ambulance	# Patients in Definitive Care on Ambulance	# Patients in Definitive care on time					
486	15	Socioeconomic Determinant Loop	Total available workforce	GDP	System level funding	Available Funding for injury care	Availability of quality injury care	Discrimination	# Patients Receiving Higher Quality Injury care	Patients Perception of Health System	Patients trust in healthcare system	Willingness to seek formal care	# Injured seeking care	# Patients taking an ambulance	# Patients in Definitive Care on Ambulance	# Patients in Definitive care Delayed					
487	15	Socioeconomic Determinant Loop	Total available workforce	GDP	System level funding	Available Funding for injury care	Availability of quality injury care	Discrimination	# Patients receiving Lower Quality Injury Care	Patients Perception of Health System	Patients trust in healthcare system	Willingness to seek formal care	# Injured seeking care	# Patients taking an ambulance	# Patients in Definitive Care on Ambulance	# Patients in Definitive care Delayed					
488	15	Socioeconomic Determinant Loop	Total available workforce	GDP	System level funding	Available Funding for injury care	Availability of quality injury rehab or follow-up	Discrimination	# Patients receiving Lower Quality Injury Care	Patients Perception of Health System	Patients trust in healthcare system	Willingness to seek formal care	# Injured seeking care	# Patients taking other transport	# Patients in Definitive Care on other Transport	# Patients in Definitive care on time					
489	15	Socioeconomic Determinant Loop	Total available workforce	GDP	Poverty	Individual wealth	Ability to pay	Willingness to receive care	# Patients in Definitive Care	# Patients receiving Lower Quality Injury Care	Patients Perception of Health System	Patients trust in healthcare system	# Injured seeking non-formal care	Willingness to seek formal care	# Injured seeking care	# Patients taking an ambulance					
490	15	Socioeconomic Determinant Loop	Total available workforce	GDP	System level funding	Available Funding for injury care	Availability of Ambulance Service	Geographical accessibility	Availability of other transport	# Patients taking other transport	# Patients in Definitive Care on other Transport	# Patients in Definitive Care	# Patients receiving Lower Quality Injury Care	Willingness to remain in care	# Patients remaining in care/rehab	# Patients Receiving Higher Quality Rehab					

491	15	Socioeconomic Determinant Loop	Total available workforce	GDP	Poverty	Community Conflict Violence etc	Availability of quality injury care	# Patients receiving Lower Quality Injury Care	Patients Perception of Health System	Patients trust in healthcare system	Willingness to receive care	# Patients in Definitive Care	# Patients Receiving Higher Quality Injury care	Willingness to remain in care	# Patients remaining in care/rehab	# Patients Receiving Higher Quality Rehab					
492	15	Socioeconomic Determinant Loop	Total available workforce	GDP	Poverty	Community Conflict Violence etc	Geographical accessibility	Availability of Ambulance Service	# Patients taking an ambulance	# Patients in Definitive Care on Ambulance	# Patients in Definitive Care	# Patients Receiving Higher Quality Injury care	Patients Perception of Health System	Patients trust in healthcare system	Willingness to seek formal care	# Injured seeking care					
493	15	Socioeconomic Determinant Loop	Total available workforce	GDP	Poverty	Individual wealth	Availability of other transport	Geographical accessibility	Availability of Ambulance Service	# Patients taking an ambulance	# Patients in Definitive Care on Ambulance	# Patients in Definitive Care	# Patients receiving Lower Quality Injury Care	Willingness to remain in care	# Patients remaining in care/rehab	# Patients Receiving Higher Quality Rehab					
494	15	Socioeconomic Determinant Loop	Total available workforce	GDP	System level funding	Available Funding for injury care	Universal health insurance	Ability to pay	Willingness to receive care	# Patients in Definitive Care	# Patients Receiving Higher Quality Injury care	Patients Perception of Health System	Patients trust in healthcare system	Willingness to remain in care	# Patients remaining in care/rehab	# Patients receiving Lower Quality Rehab					
495	15	Socioeconomic Determinant Loop	Total available workforce	GDP	System level funding	Available Funding for injury care	Availability of quality injury care	Discrimination	# Patients Receiving Higher Quality Injury care	Patients Perception of Health System	Patients trust in healthcare system	Willingness to seek formal care	# Injured seeking care	# Patients taking other transport	# Patients in Definitive Care on other Transport	# Patients in Definitive care Delayed					
496	15	Socioeconomic Determinant Loop	Total available workforce	GDP	Poverty	Individual wealth	Availability of other transport	# Patients taking other transport	# Patients in Definitive Care on other Transport	# Patients in Definitive Care	# Patients receiving Lower Quality Injury Care	Patients Perception of Health System	Patients trust in healthcare system	Willingness to seek formal care	# Injured seeking care	# Patients taking an ambulance					
497	15	Socioeconomic Determinant Loop	Total available workforce	GDP	Poverty	Community Conflict Violence etc	Availability of quality injury care	# Patients Receiving Higher Quality Injury care	Patients Perception of Health System	Patients trust in healthcare system	# Injured seeking non-formal care	Willingness to seek formal care	# Injured seeking care	# Patients taking an ambulance	# Patients in Definitive Care on Ambulance	# Patients in Definitive care on time					
498	15	Socioeconomic Determinant Loop	Total available workforce	GDP	Poverty	Community Conflict Violence etc	Availability of quality injury care	# Patients receiving Lower Quality Injury Care	Patients Perception of Health System	Patients trust in healthcare system	Willingness to receive care	# Patients in Definitive Care	# Patients Receiving Higher Quality Injury care	Willingness to remain in care	# Patients remaining in care/rehab	# Patients receiving Lower Quality Rehab					
499	15	Socioeconomic Determinant Loop	Total available workforce	GDP	Poverty	Individual wealth	Availability of other transport	Geographical accessibility	Availability of Ambulance Service	# Patients taking an ambulance	# Patients in Definitive Care on Ambulance	# Patients in Definitive Care	# Patients receiving Lower Quality Injury Care	Patients Perception of Health System	Patients trust in healthcare system	# Injured seeking non-formal care					
500	15	Socioeconomic Determinant Loop	Total available workforce	GDP	Poverty	Community Conflict Violence etc	Geographical accessibility	Availability of other transport	# Patients taking other transport	# Patients in Definitive Care on other Transport	# Patients in Definitive Care	# Patients receiving Lower Quality Injury Care	Patients Perception of Health System	Patients trust in healthcare system	Willingness to seek formal care	# Injured seeking care					
501	16	Socioeconomic Determinant Loop	Total available workforce	GDP	System level funding	Available Funding for injury care	Availability of quality injury care	Discrimination	# Patients Receiving Higher Quality Injury care	Patients Perception of Health System	Patients trust in healthcare system	# Injured seeking non-formal care	Willingness to seek formal care	# Injured seeking care	# Patients taking other transport	# Patients in Definitive Care on other Transport	# Patients in Definitive care on time				
502	16	Socioeconomic Determinant Loop	Total available workforce	GDP	System level funding	Available Funding for injury care	Availability of quality injury care	Discrimination	# Patients Receiving Higher Quality Injury care	Patients Perception of Health System	Patients trust in healthcare system	Willingness to seek formal care	# Injured seeking care	# Patients taking other transport	# Patients in Definitive Care on other Transport	# Patients in Definitive Care	# Patients receiving Lower Quality Injury Care				
503	16	Socioeconomic Determinant Loop	Total available workforce	GDP	Poverty	Community Conflict Violence etc	Availability of quality injury care	Discrimination	# Patients Receiving Higher Quality Injury care	Patients Perception of Health System	Patients trust in healthcare system	Willingness to receive care	# Patients in Definitive Care	# Patients receiving Lower Quality Injury Care	Willingness to remain in care	# Patients remaining in care/rehab	# Patients receiving Lower Quality Rehab				
504	16	Socioeconomic Determinant Loop	Total available workforce	GDP	System	Available Funding	Availability	# Patients receiving	Patients Perceptio	Patients trust in	# Injured seeking	Willingness to seek	# Injured	# Patients taking an	# Patients in Definitive	# Patients in	# Patients Receiving				

					level funding	for injury care	of quality injury care	Lower Quality Injury Care	n of Health System	healthcare system	non- formal care	formal care	seeking care	ambulanc e	Care on Ambulanc e	Definitive Care	Higher Quality Injury care				
505	16	Socioeconomi c Determinant Loop	Total available workforce	GDP	System level funding	Available Funding for injury care	Universal health insurance	Ability to pay	Willingnes s to receive care	# Patients in Definitive Care	# Patients Receiving Higher Quality Injury care	Patients Perceptio n of Health System	Patients trust in healthcare system	# Injured seeking non- formal care	Willingnes s to seek formal care	# Injured seeking care	# Patients taking an ambulanc e				
506	16	Socioeconomi c Determinant Loop	Total available workforce	GDP	System level funding	Available Funding for injury care	Availability of quality injury care	Discriminatio n	# Patients receiving Lower Quality Injury Care	Patients Perceptio n of Health System	Patients trust in healthcare system	Willingnes s to receive care	# Patients in Definitive Care	# Patients Receiving Higher Quality Injury care	Willingnes s to remain in care	# Patients remaining in care/reha b	# Patients Receiving Higher Quality Rehab				
507	16	Socioeconomi c Determinant Loop	Total available workforce	GDP	System level funding	Available Funding for injury care	Availability of quality injury care	Discriminatio n	# Patients Receiving Higher Quality Injury care	Patients Perceptio n of Health System	Patients trust in healthcare system	Willingnes s to receive care	# Patients in Definitive Care	# Patients receiving Lower Quality Injury Care	Willingnes s to remain in care	# Patients remaining in care/reha b	# Patients Receiving Higher Quality Rehab				
508	16	Socioeconomi c Determinant Loop	Total available workforce	GDP	Poverty	Individual wealth	Ability to pay	Willingness to receive care	# Patients in Definitive Care	# Patients Receiving Higher Quality Injury care	Patients Perceptio n of Health System	Patients trust in healthcare system	Willingnes s to seek formal care	# Injured seeking care	# Patients taking an ambulanc e	# Patients in Definitive Care on Ambulanc e	# Patients in Definitive care Delayed				
509	16	Socioeconomi c Determinant Loop	Total available workforce	GDP	System level funding	Available Funding for injury care	Universal health insurance	Ability to pay	Willingnes s to seek formal care	# Injured seeking care	# Patients taking other transport	# Patients in Definitive Care on other Transport	# Patients in Definitive Care	# Patients Receiving Higher Quality Injury care	Willingnes s to remain in care	# Patients remaining in care/reha b	# Patients Receiving Higher Quality Rehab				
510	16	Socioeconomi c Determinant Loop	Total available workforce	GDP	Poverty	Individual wealth	Ability to pay	Willingness to receive care	# Patients in Definitive Care	# Patients Receiving Higher Quality Injury care	Patients Perceptio n of Health System	Patients trust in healthcare system	Willingnes s to seek formal care	# Injured seeking care	# Patients taking an ambulanc e	# Patients in Definitive Care on Ambulanc e	# Patients in Definitive care on time				
511	16	Socioeconomi c Determinant Loop	Total available workforce	GDP	System level funding	Available Funding for injury care	Universal health insurance	Ability to pay	Willingnes s to seek formal care	# Injured seeking care	# Patients taking an ambulanc e	# Patients in Definitive Care on Ambulanc e	# Patients in Definitive Care	# Patients Receiving Higher Quality Injury care	Patients Perceptio n of Health System	Patients trust in healthcare system	# Injured seeking non- formal care				
512	16	Socioeconomi c Determinant Loop	Total available workforce	GDP	System level funding	Available Funding for injury care	Availability of quality injury care	Discriminatio n	# Patients Receiving Higher Quality Injury care	Patients Perceptio n of Health System	Patients trust in healthcare system	# Injured seeking non- formal care	Willingnes s to seek formal care	# Injured seeking care	# Patients taking other transport	# Patients in Definitive Care on other Transport	# Patients in Definitive care Delayed				
513	16	Socioeconomi c Determinant Loop	Total available workforce	GDP	System level funding	Available Funding for injury care	Universal health insurance	Ability to pay	Willingnes s to seek formal care	# Injured seeking care	# Patients taking other transport	# Patients in Definitive Care on other Transport	# Patients in Definitive Care	# Patients Receiving Higher Quality Injury care	Willingnes s to remain in care	# Patients remaining in care/reha b	# Patients receiving Lower Quality Rehab				
514	16	Socioeconomi c Determinant Loop	Total available workforce	GDP	Poverty	Individual wealth	Ability to pay	Willingness to receive care	# Patients in Definitive Care	# Patients Receiving Higher Quality Injury care	Patients Perceptio n of Health System	Patients trust in healthcare system	Willingnes s to seek formal care	# Injured seeking care	# Patients taking other transport	# Patients in Definitive Care on other Transport	# Patients in Definitive care on time				
515	16	Socioeconomi c Determinant Loop	Total available workforce	GDP	System level funding	Available Funding for injury care	Availability of quality injury care	# Patients Receiving Higher Quality Injury care	Patients Perceptio n of Health System	Patients trust in healthcare system	# Injured seeking non- formal care	Willingnes s to seek formal care	# Injured seeking care	# Patients taking an ambulanc e	# Patients in Definitive Care on Ambulanc e	# Patients in Definitive Care	# Patients receiving Lower Quality Injury Care				
516	16	Socioeconomi c Determinant Loop	Total available workforce	GDP	Poverty	Communit y Conflict Violence etc	Availability of quality injury care	# Patients Receiving Higher Quality Injury care	Patients Perceptio n of Health System	Patients trust in healthcare system	# Injured seeking non- formal care	Willingnes s to seek formal care	# Injured seeking care	# Patients taking other transport	# Patients in Definitive Care on other Transport	# Patients in Definitive Care	# Patients receiving Lower Quality Injury Care				
517	16	Socioeconomi c Determinant Loop	Total available workforce	GDP	Poverty	Communit y Conflict Violence etc	Availability of quality injury rehab or follow-up	Discriminatio n	# Patients receiving Lower Quality Injury Care	Patients Perceptio n of Health System	Patients trust in healthcare system	# Injured seeking non- formal care	Willingnes s to seek formal care	# Injured seeking care	# Patients taking an ambulanc e	# Patients in Definitive Care on Ambulanc e	# Patients in Definitive care Delayed				

518	16	Socioeconomic Determinant Loop	Total available workforce	GDP	Poverty	Community Conflict Violence etc	Availability of quality injury care	# Patients Receiving Higher Quality Injury care	Patients Perception of Health System	Patients trust in healthcare system	Willingness to seek formal care	# Injured seeking care	# Patients taking other transport	# Patients in Definitive Care on other Transport	# Patients in Definitive Care	# Patients remaining in care/rehab	# Patients Receiving Higher Quality Rehab			
519	16	Socioeconomic Determinant Loop	Total available workforce	GDP	System level funding	Available Funding for injury care	Availability of quality injury care	# Patients receiving Lower Quality Injury Care	Patients Perception of Health System	Patients trust in healthcare system	# Injured seeking non-formal care	Willingness to seek formal care	# Injured seeking care	# Patients taking other transport	# Patients in Definitive Care on other Transport	# Patients in Definitive Care	# Patients Receiving Higher Quality Injury care			
520	16	Socioeconomic Determinant Loop	Total available workforce	GDP	System level funding	Available Funding for injury care	Availability of quality injury rehab or follow-up	Discrimination	# Patients receiving Lower Quality Injury Care	Patients Perception of Health System	Patients trust in healthcare system	Willingness to receive care	# Patients in Definitive Care	# Patients Receiving Higher Quality Injury care	Willingness to remain in care	# Patients remaining in care/rehab	# Patients receiving Lower Quality Rehab			
521	16	Socioeconomic Determinant Loop	Total available workforce	GDP	Poverty	Community Conflict Violence etc	Availability of quality injury care	Discrimination	# Patients receiving Lower Quality Injury Care	Patients Perception of Health System	Patients trust in healthcare system	# Injured seeking non-formal care	Willingness to seek formal care	# Injured seeking care	# Patients taking other transport	# Patients in Definitive Care on other Transport	# Patients in Definitive care on time			
522	16	Socioeconomic Determinant Loop	Total available workforce	GDP	Poverty	Community Conflict Violence etc	Availability of quality injury care	Discrimination	# Patients Receiving Higher Quality Injury care	Patients Perception of Health System	Patients trust in healthcare system	Willingness to seek formal care	# Injured seeking care	# Patients taking an ambulance	# Patients in Definitive Care on Ambulance	# Patients in Definitive Care	# Patients receiving Lower Quality Injury Care			
523	16	Socioeconomic Determinant Loop	Total available workforce	GDP	System level funding	Available Funding for injury care	Availability of quality injury care	# Patients Receiving Higher Quality Injury care	Patients Perception of Health System	Patients trust in healthcare system	Willingness to seek formal care	# Injured seeking care	# Patients taking an ambulance	# Patients in Definitive Care on Ambulance	# Patients in Definitive Care	# Patients remaining in care/rehab	# Patients receiving Lower Quality Rehab			
524	16	Socioeconomic Determinant Loop	Total available workforce	GDP	Poverty	Community Conflict Violence etc	Availability of quality injury rehab or follow-up	Discrimination	# Patients Receiving Higher Quality Injury care	Patients Perception of Health System	Patients trust in healthcare system	# Injured seeking non-formal care	Willingness to seek formal care	# Injured seeking care	# Patients taking an ambulance	# Patients in Definitive Care on Ambulance	# Patients in Definitive care on time			
525	16	Socioeconomic Determinant Loop	Total available workforce	GDP	Poverty	Community Conflict Violence etc	Availability of quality injury care	# Patients receiving Lower Quality Injury Care	Patients Perception of Health System	Patients trust in healthcare system	Willingness to seek formal care	# Injured seeking care	# Patients taking an ambulance	# Patients in Definitive Care on Ambulance	# Patients in Definitive Care	# Patients remaining in care/rehab	# Patients receiving Lower Quality Rehab			
526	16	Socioeconomic Determinant Loop	Total available workforce	GDP	System level funding	Available Funding for injury care	Availability of quality injury care	Discrimination	# Patients Receiving Higher Quality Injury care	Patients Perception of Health System	Patients trust in healthcare system	Willingness to receive care	# Patients in Definitive Care	# Patients receiving Lower Quality Injury Care	Willingness to remain in care	# Patients remaining in care/rehab	# Patients receiving Lower Quality Rehab			
527	16	Socioeconomic Determinant Loop	Total available workforce	GDP	System level funding	Available Funding for injury care	Availability of quality injury care	Discrimination	# Patients receiving Lower Quality Injury Care	Patients Perception of Health System	Patients trust in healthcare system	Willingness to seek formal care	# Injured seeking care	# Patients taking other transport	# Patients in Definitive Care on other Transport	# Patients in Definitive Care	# Patients Receiving Higher Quality Injury care			
528	16	Socioeconomic Determinant Loop	Total available workforce	GDP	System level funding	Available Funding for injury care	Availability of quality injury care	Discrimination	# Patients receiving Lower Quality Injury Care	Patients Perception of Health System	Patients trust in healthcare system	# Injured seeking non-formal care	Willingness to seek formal care	# Injured seeking care	# Patients taking an ambulance	# Patients in Definitive Care on Ambulance	# Patients in Definitive care on time			
529	16	Socioeconomic Determinant Loop	Total available workforce	GDP	Poverty	Community Conflict Violence etc	Geographical accessibility	Availability of Ambulance Service	# Patients taking an ambulance	# Patients in Definitive Care on Ambulance	# Patients in Definitive Care	# Patients receiving Lower Quality Injury Care	Patients Perception of Health System	Patients trust in healthcare system	# Injured seeking non-formal care	Willingness to seek formal care	# Injured seeking care			
530	16	Socioeconomic Determinant Loop	Total available workforce	GDP	System level funding	Available Funding for injury care	Availability of quality injury rehab or follow-up	Discrimination	# Patients Receiving Higher Quality Injury care	Patients Perception of Health System	Patients trust in healthcare system	# Injured seeking non-formal care	Willingness to seek formal care	# Injured seeking care	# Patients taking other transport	# Patients in Definitive Care on other Transport	# Patients in Definitive care on time			

531	16	Socioeconomic Determinant Loop	Total available workforce	GDP	Poverty	Individual wealth	Availability of other transport	Geographical accessibility	Availability of Ambulance Service	# Patients taking an ambulance	# Patients in Definitive Care on Ambulance	# Patients in Definitive Care	# Patients receiving Lower Quality Injury Care	Patients Perception of Health System	Patients trust in healthcare system	Willingness to seek formal care	# Injured seeking care				
532	16	Socioeconomic Determinant Loop	Total available workforce	GDP	System level funding	Available Funding for injury care	Universal health insurance	Ability to pay	Willingness to seek formal care	# Injured seeking care	# Patients taking other transport	# Patients in Definitive Care on other Transport	# Patients in Definitive Care	# Patients receiving Lower Quality Injury Care	Patients Perception of Health System	Patients trust in healthcare system	# Injured seeking non-formal care				
533	16	Socioeconomic Determinant Loop	Total available workforce	GDP	System level funding	Available Funding for injury care	Availability of quality injury care	# Patients Receiving Higher Quality Injury care	Patients Perception of Health System	Patients trust in healthcare system	# Injured seeking non-formal care	Willingness to seek formal care	# Injured seeking care	# Patients taking other transport	# Patients in Definitive Care on other Transport	# Patients in Definitive Care	# Patients receiving Lower Quality Injury Care				
534	16	Socioeconomic Determinant Loop	Total available workforce	GDP	Poverty	Community Conflict Violence etc	Geographical accessibility	Availability of other transport	# Patients taking other transport	# Patients in Definitive Care on other Transport	# Patients in Definitive Care	# Patients Receiving Higher Quality Injury care	Patients Perception of Health System	Patients trust in healthcare system	Willingness to remain in care	# Patients remaining in care/rehab	# Patients receiving Lower Quality Rehab				
535	16	Socioeconomic Determinant Loop	Total available workforce	GDP	Poverty	Community Conflict Violence etc	Geographical accessibility	Availability of Ambulance Service	# Patients taking an ambulance	# Patients in Definitive Care on Ambulance	# Patients in Definitive Care	# Patients Receiving Higher Quality Injury care	Patients Perception of Health System	Patients trust in healthcare system	Willingness to remain in care	# Patients remaining in care/rehab	# Patients receiving Lower Quality Rehab				
536	16	Socioeconomic Determinant Loop	Total available workforce	GDP	Poverty	Community Conflict Violence etc	Availability of quality injury care	Discrimination	# Patients receiving Lower Quality Injury Care	Patients Perception of Health System	Patients trust in healthcare system	Willingness to seek formal care	# Injured seeking care	# Patients taking other transport	# Patients in Definitive Care on other Transport	# Patients in Definitive Care	# Patients Receiving Higher Quality Injury care				
537	16	Socioeconomic Determinant Loop	Total available workforce	GDP	System level funding	Available Funding for injury care	Availability of quality injury care	Discrimination	# Patients receiving Lower Quality Injury Care	Patients Perception of Health System	Patients trust in healthcare system	Willingness to receive care	# Patients in Definitive Care	# Patients Receiving Higher Quality Injury care	Willingness to remain in care	# Patients remaining in care/rehab	# Patients receiving Lower Quality Rehab				
538	16	Socioeconomic Determinant Loop	Total available workforce	GDP	Poverty	Community Conflict Violence etc	Geographical accessibility	Availability of Ambulance Service	# Patients taking an ambulance	# Patients in Definitive Care on Ambulance	# Patients in Definitive Care	# Patients Receiving Higher Quality Injury care	Patients Perception of Health System	Patients trust in healthcare system	# Injured seeking non-formal care	Willingness to seek formal care	# Injured seeking care				
539	16	Socioeconomic Determinant Loop	Total available workforce	GDP	Poverty	Community Conflict Violence etc	Availability of quality injury care	# Patients Receiving Higher Quality Injury care	Patients Perception of Health System	Patients trust in healthcare system	Willingness to seek formal care	# Injured seeking care	# Patients taking an ambulance	# Patients in Definitive Care on Ambulance	# Patients in Definitive Care	# Patients remaining in care/rehab	# Patients receiving Lower Quality Rehab				
540	16	Socioeconomic Determinant Loop	Total available workforce	GDP	Poverty	Community Conflict Violence etc	Availability of quality injury rehab or follow-up	Discrimination	# Patients Receiving Higher Quality Injury care	Patients Perception of Health System	Patients trust in healthcare system	# Injured seeking non-formal care	Willingness to seek formal care	# Injured seeking care	# Patients taking other transport	# Patients in Definitive Care on other Transport	# Patients in Definitive care Delayed				
541	16	Socioeconomic Determinant Loop	Total available workforce	GDP	Poverty	Community Conflict Violence etc	Availability of quality injury care	# Patients receiving Lower Quality Injury Care	Patients Perception of Health System	Patients trust in healthcare system	Willingness to seek formal care	# Injured seeking care	# Patients taking an ambulance	# Patients in Definitive Care on Ambulance	# Patients in Definitive Care	# Patients remaining in care/rehab	# Patients Receiving Higher Quality Rehab				
542	16	Socioeconomic Determinant Loop	Total available workforce	GDP	Poverty	Individual wealth	Ability to pay	Willingness to receive care	# Patients in Definitive Care	# Patients receiving Lower Quality Injury Care	Patients Perception of Health System	Patients trust in healthcare system	Willingness to seek formal care	# Injured seeking care	# Patients taking other transport	# Patients in Definitive Care on other Transport	# Patients in Definitive care Delayed				
543	16	Socioeconomic Determinant Loop	Total available workforce	GDP	System level funding	Available Funding for injury care	Availability of quality injury care	Discrimination	# Patients Receiving Higher Quality Injury care	Patients Perception of Health System	Patients trust in healthcare system	# Injured seeking non-formal care	Willingness to seek formal care	# Injured seeking care	# Patients taking an ambulance	# Patients in Definitive Care on Ambulance	# Patients in Definitive care on time				
544	16	Socioeconomic Determinant Loop	Total available workforce	GDP	System	Available Funding	Availability	Discrimination	# Patients Receiving	Patients Perceptio	Patients trust in	# Injured seeking	Willingness to seek	# Injured	# Patients taking an	# Patients in Definitive	# Patients in Definitive				

					level funding	for injury care	of quality injury care		Higher Quality Injury care	n of Health System	healthcare system	non-formal care	formal care	seeking care	ambulance	Care on Ambulance	care Delayed				
545	16	Socioeconomic Determinant Loop	Total available workforce	GDP	Poverty	Community Conflict Violence etc	Availability of quality injury care	Discrimination	# Patients Receiving Higher Quality Injury care	Patients Perception of Health System	Patients trust in healthcare system	# Injured seeking non-formal care	Willingness to seek formal care	# Injured seeking care	# Patients taking other transport	# Patients in Definitive Care on other Transport	# Patients in Definitive care Delayed				
546	16	Socioeconomic Determinant Loop	Total available workforce	GDP	Poverty	Community Conflict Violence etc	Availability of quality injury rehab or follow-up	Discrimination	# Patients Receiving Higher Quality Injury care	Patients Perception of Health System	Patients trust in healthcare system	Willingness to seek formal care	# Injured seeking care	# Patients taking other transport	# Patients in Definitive Care on other Transport	# Patients in Definitive Care	# Patients receiving Lower Quality Injury Care				
547	16	Socioeconomic Determinant Loop	Total available workforce	GDP	Poverty	Community Conflict Violence etc	Availability of quality injury rehab or follow-up	Discrimination	# Patients receiving Lower Quality Injury Care	Patients Perception of Health System	Patients trust in healthcare system	# Injured seeking non-formal care	Willingness to seek formal care	# Injured seeking care	# Patients taking an ambulance	# Patients in Definitive Care on Ambulance	# Patients in Definitive care on time				
548	16	Socioeconomic Determinant Loop	Total available workforce	GDP	Poverty	Community Conflict Violence etc	Availability of quality injury rehab or follow-up	Discrimination	# Patients Receiving Higher Quality Injury care	Patients Perception of Health System	Patients trust in healthcare system	# Injured seeking non-formal care	Willingness to seek formal care	# Injured seeking care	# Patients taking an ambulance	# Patients in Definitive Care on Ambulance	# Patients in Definitive care Delayed				
549	16	Socioeconomic Determinant Loop	Total available workforce	GDP	System level funding	Available Funding for injury care	Availability of quality injury care	Discrimination	# Patients receiving Lower Quality Injury Care	Patients Perception of Health System	Patients trust in healthcare system	Willingness to seek formal care	# Injured seeking care	# Patients taking an ambulance	# Patients in Definitive Care on Ambulance	# Patients in Definitive Care	# Patients Receiving Higher Quality Injury care				
550	16	Socioeconomic Determinant Loop	Total available workforce	GDP	Poverty	Community Conflict Violence etc	Geographical accessibility	Availability of other transport	# Patients taking other transport	# Patients in Definitive Care on other Transport	# Patients in Definitive Care	# Patients receiving Lower Quality Injury Care	Patients Perception of Health System	Patients trust in healthcare system	Willingness to remain in care	# Patients remaining in care/rehab	# Patients Receiving Higher Quality Rehab				
551	16	Socioeconomic Determinant Loop	Total available workforce	GDP	Poverty	Community Conflict Violence etc	Availability of quality injury care	Discrimination	# Patients receiving Lower Quality Injury Care	Patients Perception of Health System	Patients trust in healthcare system	# Injured seeking non-formal care	Willingness to seek formal care	# Injured seeking care	# Patients taking an ambulance	# Patients in Definitive Care on Ambulance	# Patients in Definitive care on time				
552	16	Socioeconomic Determinant Loop	Total available workforce	GDP	Poverty	Community Conflict Violence etc	Geographical accessibility	Availability of other transport	# Patients taking other transport	# Patients in Definitive Care on other Transport	# Patients in Definitive Care	# Patients Receiving Higher Quality Injury care	Patients Perception of Health System	Patients trust in healthcare system	# Injured seeking non-formal care	Willingness to seek formal care	# Injured seeking care				
553	16	Socioeconomic Determinant Loop	Total available workforce	GDP	Poverty	Community Conflict Violence etc	Availability of quality injury rehab or follow-up	Discrimination	# Patients Receiving Higher Quality Injury care	Patients Perception of Health System	Patients trust in healthcare system	# Injured seeking non-formal care	Willingness to seek formal care	# Injured seeking care	# Patients taking other transport	# Patients in Definitive Care on other Transport	# Patients in Definitive care on time				
554	16	Socioeconomic Determinant Loop	Total available workforce	GDP	Poverty	Community Conflict Violence etc	Availability of quality injury rehab or follow-up	Discrimination	# Patients Receiving Higher Quality Injury care	Patients Perception of Health System	Patients trust in healthcare system	Willingness to seek formal care	# Injured seeking care	# Patients taking an ambulance	# Patients in Definitive Care on Ambulance	# Patients in Definitive Care	# Patients receiving Lower Quality Injury Care				
555	16	Socioeconomic Determinant Loop	Total available workforce	GDP	Poverty	Community Conflict Violence etc	Availability of quality injury care	# Patients Receiving Higher Quality Injury care	Patients Perception of Health System	Patients trust in healthcare system	Willingness to seek formal care	# Injured seeking care	# Patients taking an ambulance	# Patients in Definitive Care on Ambulance	# Patients in Definitive Care	# Patients remaining in care/rehab	# Patients Receiving Higher Quality Rehab				
556	16	Socioeconomic Determinant Loop	Total available workforce	GDP	Poverty	Individual wealth	Ability to pay	Willingness to receive care	# Patients in Definitive Care	# Patients receiving Lower Quality Injury Care	Patients Perception of Health System	Patients trust in healthcare system	Willingness to seek formal care	# Injured seeking care	# Patients taking other transport	# Patients in Definitive Care on other Transport	# Patients in Definitive care on time				
557	16	Socioeconomic Determinant Loop	Total available workforce	GDP	Poverty	Community Conflict Violence etc	Availability of quality injury care	# Patients receiving Lower	Patients Perception of	Patients trust in healthcare system	# Injured seeking non-	Willingness to seek formal care	# Injured seeking care	# Patients taking other transport	# Patients in Definitive Care on	# Patients in Definitive Care	# Patients Receiving Higher				

								Quality Injury Care	Health System		formal care				other Transport		Quality Injury care				
558	16	Socioeconomi c Determinant Loop	Total available workforce	GDP	System level funding	Available Funding for injury care	Availability of quality injury care	Discriminatio n	# Patients receiving Lower Quality Injury Care	Patients Perceptio n of Health System	Patients trust in healthcare system	# Injured seeking non-formal care	Willingnes s to seek formal care	# Injured seeking care	# Patients taking an ambulanc e	# Patients in Definitive Care on Ambulanc e	# Patients in Definitive care Delayed				
559	16	Socioeconomi c Determinant Loop	Total available workforce	GDP	Poverty	Communit y Conflict Violence etc	Availability of quality injury care	Discriminatio n	# Patients receiving Lower Quality Injury Care	Patients Perceptio n of Health System	Patients trust in healthcare system	# Injured seeking non-formal care	Willingnes s to seek formal care	# Injured seeking care	# Patients taking other transport	# Patients in Definitive Care on other Transport	# Patients in Definitive care Delayed				
560	16	Socioeconomi c Determinant Loop	Total available workforce	GDP	Poverty	Individual wealth	Ability to pay	Willingness to receive care	# Patients in Definitive Care	# Patients Receiving Higher Quality Injury care	Patients Perceptio n of Health System	Patients trust in healthcare system	Willingnes s to seek formal care	# Injured seeking care	# Patients taking other transport	# Patients in Definitive Care on other Transport	# Patients in Definitive care Delayed				
561	16	Socioeconomi c Determinant Loop	Total available workforce	GDP	Poverty	Communit y Conflict Violence etc	Availability of quality injury care	# Patients Receiving Higher Quality Injury care	Patients Perceptio n of Health System	Patients trust in healthcare system	Willingnes s to seek formal care	# Injured seeking care	# Patients taking other transport	# Patients in Definitive Care on other Transport	# Patients in Definitive Care	# Patients remaining in care/reha b	# Patients receiving Lower Quality Rehab				
562	16	Socioeconomi c Determinant Loop	Total available workforce	GDP	Poverty	Communit y Conflict Violence etc	Availability of quality injury care	# Patients receiving Lower Quality Injury Care	Patients Perceptio n of Health System	Patients trust in healthcare system	Willingnes s to seek formal care	# Injured seeking care	# Patients taking other transport	# Patients in Definitive Care on other Transport	# Patients in Definitive Care	# Patients remaining in care/reha b	# Patients Receiving Higher Quality Rehab				
563	16	Socioeconomi c Determinant Loop	Total available workforce	GDP	System level funding	Available Funding for injury care	Availability of quality injury care	# Patients receiving Lower Quality Injury Care	Patients Perceptio n of Health System	Patients trust in healthcare system	Willingnes s to seek formal care	# Injured seeking care	# Patients taking an ambulanc e	# Patients in Definitive Care on Ambulanc e	# Patients in Definitive Care	# Patients remaining in care/reha b	# Patients Receiving Higher Quality Rehab				
564	16	Socioeconomi c Determinant Loop	Total available workforce	GDP	Poverty	Communit y Conflict Violence etc	Geographic al accessibility	Availability of other transport	# Patients taking other transport	# Patients in Definitive Care on other Transport	# Patients in Definitive Care	# Patients Receiving Higher Quality Injury care	Patients Perceptio n of Health System	Patients trust in healthcare system	Willingnes s to remain in care	# Patients remaining in care/reha b	# Patients Receiving Higher Quality Rehab				
565	16	Socioeconomi c Determinant Loop	Total available workforce	GDP	Poverty	Communit y Conflict Violence etc	Availability of quality injury care	Discriminatio n	# Patients Receiving Higher Quality Injury care	Patients Perceptio n of Health System	Patients trust in healthcare system	Willingnes s to receive care	# Patients in Definitive Care	# Patients receiving Lower Quality Injury Care	Willingnes s to remain in care	# Patients remaining in care/reha b	# Patients Receiving Higher Quality Rehab				
566	16	Socioeconomi c Determinant Loop	Total available workforce	GDP	System level funding	Available Funding for injury care	Availability of quality injury rehab or follow-up	Discriminatio n	# Patients receiving Lower Quality Injury Care	Patients Perceptio n of Health System	Patients trust in healthcare system	# Injured seeking non-formal care	Willingnes s to seek formal care	# Injured seeking care	# Patients taking an ambulanc e	# Patients in Definitive Care on Ambulanc e	# Patients in Definitive care Delayed				
567	16	Socioeconomi c Determinant Loop	Total available workforce	GDP	Poverty	Communit y Conflict Violence etc	Availability of quality injury care	Discriminatio n	# Patients Receiving Higher Quality Injury care	Patients Perceptio n of Health System	Patients trust in healthcare system	# Injured seeking non-formal care	Willingnes s to seek formal care	# Injured seeking care	# Patients taking an ambulanc e	# Patients in Definitive Care on Ambulanc e	# Patients in Definitive care Delayed				
568	16	Socioeconomi c Determinant Loop	Total available workforce	GDP	System level funding	Available Funding for injury care	Availability of quality injury rehab or follow-up	Discriminatio n	# Patients Receiving Higher Quality Injury care	Patients Perceptio n of Health System	Patients trust in healthcare system	# Injured seeking non-formal care	Willingnes s to seek formal care	# Injured seeking care	# Patients taking an ambulanc e	# Patients in Definitive Care on Ambulanc e	# Patients in Definitive care Delayed				
569	16	Socioeconomi c Determinant Loop	Total available workforce	GDP	System level funding	Available Funding for injury care	Availability of Ambulance Service	Geographica l accessibility	Availability of other transport	# Patients taking other transport	# Patients in Definitive Care on other Transport	# Patients in Definitive Care	# Patients receiving Lower Quality Injury Care	Patients Perceptio n of Health System	Patients trust in healthcare system	Willingnes s to seek formal care	# Injured seeking care				
570	16	Socioeconomi c Determinant Loop	Total available workforce	GDP	Poverty	Communit y Conflict Violence etc	Geographic al accessibility	Availability of other transport	# Patients taking other transport	# Patients in Definitive Care on other Transport	# Patients in Definitive Care	# Patients receiving Lower Quality Injury Care	Patients Perceptio n of Health System	Patients trust in healthcare system	# Injured seeking non-formal care	Willingnes s to seek formal care	# Injured seeking care				

571	16	Socioeconomi c Determinant Loop	Total available workforce	GDP	Poverty	Communit y Conflict Violence etc	Geographic al accessibility	Availability of Ambulance Service	# Patients taking an ambulanc e	# Patients in Definitive Care on Ambulanc e	# Patients in Definitive Care	# Patients receiving Lower Quality Injury Care	Patients Perceptio n of Health System	Patients trust in healthcare system	Willingnes s to remain in care	# Patients remaining in care/reha b	# Patients Receiving Higher Quality Rehab				
572	16	Socioeconomi c Determinant Loop	Total available workforce	GDP	Poverty	Individual wealth	Availability of other transport	Geographica l accessibility	Availability of Ambulanc e Service	# Patients taking an ambulanc e	# Patients in Definitive Care on Ambulanc e	# Patients in Definitive Care	# Patients Receiving Higher Quality Injury care	Patients Perceptio n of Health System	Patients trust in healthcare system	Willingnes s to seek formal care	# Injured seeking care				
573	16	Socioeconomi c Determinant Loop	Total available workforce	GDP	Poverty	Communit y Conflict Violence etc	Availability of quality injury care	Discriminatio n	# Patients Receiving Higher Quality Injury care	Patients Perceptio n of Health System	Patients trust in healthcare system	# Injured seeking non-formal care	Willingnes s to seek formal care	# Injured seeking care	# Patients taking other transport	# Patients in Definitive Care on other Transport	# Patients in Definitive care on time				
574	16	Socioeconomi c Determinant Loop	Total available workforce	GDP	System level funding	Available Funding for injury care	Universal health insurance	Ability to pay	Willingnes s to receive care	# Patients in Definitive Care	# Patients receiving Lower Quality Injury Care	Patients Perceptio n of Health System	Patients trust in healthcare system	# Injured seeking non-formal care	Willingnes s to seek formal care	# Injured seeking care	# Patients taking an ambulanc e				
575	16	Socioeconomi c Determinant Loop	Total available workforce	GDP	System level funding	Available Funding for injury care	Availability of quality injury rehab or follow-up	Discriminatio n	# Patients Receiving Higher Quality Injury care	Patients Perceptio n of Health System	Patients trust in healthcare system	# Injured seeking non-formal care	Willingnes s to seek formal care	# Injured seeking care	# Patients taking other transport	# Patients in Definitive Care on other Transport	# Patients in Definitive care Delayed				
576	16	Socioeconomi c Determinant Loop	Total available workforce	GDP	Poverty	Communit y Conflict Violence etc	Availability of quality injury care	# Patients receiving Lower Quality Injury Care	Patients Perceptio n of Health System	Patients trust in healthcare system	Willingnes s to seek formal care	# Injured seeking care	# Patients taking other transport	# Patients in Definitive Care on other Transport	# Patients in Definitive Care	# Patients remaining in care/reha b	# Patients receiving Lower Quality Rehab				
577	16	Socioeconomi c Determinant Loop	Total available workforce	GDP	System level funding	Available Funding for injury care	Universal health insurance	Ability to pay	Willingnes s to seek formal care	# Injured seeking care	# Patients taking an ambulanc e	# Patients in Definitive Care on Ambulanc e	# Patients in Definitive Care	# Patients receiving Lower Quality Injury Care	Willingnes s to remain in care	# Patients remaining in care/reha b	# Patients Receiving Higher Quality Rehab				
578	16	Socioeconomi c Determinant Loop	Total available workforce	GDP	Poverty	Communit y Conflict Violence etc	Geographic al accessibility	Availability of other transport	# Patients taking other transport	# Patients in Definitive Care on other Transport	# Patients in Definitive Care	# Patients receiving Lower Quality Injury Care	Patients Perceptio n of Health System	Patients trust in healthcare system	Willingnes s to remain in care	# Patients remaining in care/reha b	# Patients receiving Lower Quality Rehab				
579	16	Socioeconomi c Determinant Loop	Total available workforce	GDP	Poverty	Communit y Conflict Violence etc	Availability of quality injury care	# Patients Receiving Higher Quality Injury care	Patients Perceptio n of Health System	Patients trust in healthcare system	# Injured seeking non-formal care	Willingnes s to seek formal care	# Injured seeking care	# Patients taking an ambulanc e	# Patients in Definitive Care on Ambulanc e	# Patients in Definitive Care	# Patients receiving Lower Quality Injury Care				
580	16	Socioeconomi c Determinant Loop	Total available workforce	GDP	Poverty	Communit y Conflict Violence etc	Geographic al accessibility	Availability of other transport	# Patients taking other transport	# Patients in Definitive Care on other Transport	# Patients in Definitive Care	# Patients Receiving Higher Quality Injury care	Patients Perceptio n of Health System	Patients trust in healthcare system	Willingnes s to seek formal care	# Injured seeking care	# Patients taking an ambulanc e				
581	16	Socioeconomi c Determinant Loop	Total available workforce	GDP	System level funding	Available Funding for injury care	Availability of quality injury rehab or follow-up	Discriminatio n	# Patients Receiving Higher Quality Injury care	Patients Perceptio n of Health System	Patients trust in healthcare system	# Injured seeking non-formal care	Willingnes s to seek formal care	# Injured seeking care	# Patients taking an ambulanc e	# Patients in Definitive Care on Ambulanc e	# Patients in Definitive care on time				
582	16	Socioeconomi c Determinant Loop	Total available workforce	GDP	Poverty	Communit y Conflict Violence etc	Geographic al accessibility	Availability of Ambulance Service	# Patients taking an ambulanc e	# Patients in Definitive Care on Ambulanc e	# Patients in Definitive Care	# Patients receiving Lower Quality Injury Care	Patients Perceptio n of Health System	Patients trust in healthcare system	Willingnes s to remain in care	# Patients remaining in care/reha b	# Patients receiving Lower Quality Rehab				
583	16	Socioeconomi c Determinant Loop	Total available workforce	GDP	System level funding	Available Funding for injury care	Availability of Ambulance Service	Geographica l accessibility	Availability of other transport	# Patients taking other transport	# Patients in Definitive Care on other Transport	# Patients in Definitive Care	# Patients Receiving Higher Quality Injury care	Patients Perceptio n of Health System	Patients trust in healthcare system	Willingnes s to seek formal care	# Injured seeking care				
584	16	Socioeconomi c Determinant Loop	Total available workforce	GDP	Poverty	Communit y Conflict	Availability of quality	Discriminatio n	# Patients receiving	Patients Perceptio	Patients trust in	# Injured seeking	Willingnes s to seek	# Injured	# Patients taking	# Patients in Definitive	# Patients in Definitive				

						Violence etc	injury rehab or follow-up		Lower Quality Injury Care	n of Health System	healthcare system	non-formal care	formal care	seeking care	other transport	Care on other Transport	care Delayed				
585	16	Socioeconomic Determinant Loop	Total available workforce	GDP	System level funding	Available Funding for injury care	Availability of quality injury care	# Patients receiving Lower Quality Injury Care	Patients Perception of Health System	Patients trust in healthcare system	Willingness to seek formal care	# Injured seeking care	# Patients taking other transport	# Patients in Definitive Care on other Transport	# Patients in Definitive Care	# Patients remaining in care/rehab	# Patients receiving Lower Quality Rehab				
586	16	Socioeconomic Determinant Loop	Total available workforce	GDP	System level funding	Available Funding for injury care	Universal health insurance	Ability to pay	Willingness to seek formal care	# Injured seeking care	# Patients taking other transport	# Patients in Definitive Care on other Transport	# Patients in Definitive Care	# Patients receiving Lower Quality Injury Care	Willingness to remain in care	# Patients remaining in care/rehab	# Patients receiving Lower Quality Rehab				
587	16	Socioeconomic Determinant Loop	Total available workforce	GDP	System level funding	Available Funding for injury care	Universal health insurance	Ability to pay	Willingness to seek formal care	# Injured seeking care	# Patients taking other transport	# Patients in Definitive Care on other Transport	# Patients in Definitive Care	# Patients receiving Lower Quality Injury Care	Willingness to remain in care	# Patients remaining in care/rehab	# Patients Receiving Higher Quality Rehab				
588	16	Socioeconomic Determinant Loop	Total available workforce	GDP	System level funding	Available Funding for injury care	Availability of quality injury rehab or follow-up	Discrimination	# Patients receiving Lower Quality Injury Care	Patients Perception of Health System	Patients trust in healthcare system	Willingness to seek formal care	# Injured seeking care	# Patients taking an ambulance	# Patients in Definitive Care on Ambulance	# Patients in Definitive Care	# Patients Receiving Higher Quality Injury care				
589	16	Socioeconomic Determinant Loop	Total available workforce	GDP	System level funding	Available Funding for injury care	Availability of quality injury rehab or follow-up	Discrimination	# Patients Receiving Higher Quality Injury care	Patients Perception of Health System	Patients trust in healthcare system	Willingness to seek formal care	# Injured seeking care	# Patients taking an ambulance	# Patients in Definitive Care on Ambulance	# Patients in Definitive Care	# Patients receiving Lower Quality Injury Care				
590	16	Socioeconomic Determinant Loop	Total available workforce	GDP	System level funding	Available Funding for injury care	Availability of quality injury care	# Patients Receiving Higher Quality Injury care	Patients Perception of Health System	Patients trust in healthcare system	Willingness to seek formal care	# Injured seeking care	# Patients taking an ambulance	# Patients in Definitive Care on Ambulance	# Patients in Definitive Care	# Patients remaining in care/rehab	# Patients Receiving Higher Quality Rehab				
591	16	Socioeconomic Determinant Loop	Total available workforce	GDP	System level funding	Available Funding for injury care	Availability of quality injury care	# Patients receiving Lower Quality Injury Care	Patients Perception of Health System	Patients trust in healthcare system	Willingness to seek formal care	# Injured seeking care	# Patients taking an ambulance	# Patients in Definitive Care on Ambulance	# Patients in Definitive Care	# Patients remaining in care/rehab	# Patients receiving Lower Quality Rehab				
592	16	Socioeconomic Determinant Loop	Total available workforce	GDP	Poverty	Community Conflict Violence etc	Availability of quality injury rehab or follow-up	Discrimination	# Patients Receiving Higher Quality Injury care	Patients Perception of Health System	Patients trust in healthcare system	Willingness to receive care	# Patients in Definitive Care	# Patients receiving Lower Quality Injury Care	Willingness to remain in care	# Patients remaining in care/rehab	# Patients Receiving Higher Quality Rehab				
593	16	Socioeconomic Determinant Loop	Total available workforce	GDP	Poverty	Individual wealth	Availability of other transport	# Patients taking other transport	# Patients in Definitive Care on other Transport	# Patients in Definitive Care	# Patients receiving Lower Quality Injury Care	Patients Perception of Health System	Patients trust in healthcare system	# Injured seeking non-formal care	Willingness to seek formal care	# Injured seeking care	# Patients taking an ambulance				
594	16	Socioeconomic Determinant Loop	Total available workforce	GDP	Poverty	Community Conflict Violence etc	Availability of quality injury care	# Patients receiving Lower Quality Injury Care	Patients Perception of Health System	Patients trust in healthcare system	# Injured seeking non-formal care	Willingness to seek formal care	# Injured seeking care	# Patients taking an ambulance	# Patients in Definitive Care on Ambulance	# Patients in Definitive Care	# Patients Receiving Higher Quality Injury care				
595	16	Socioeconomic Determinant Loop	Total available workforce	GDP	Poverty	Community Conflict Violence etc	Geographical accessibility	Availability of other transport	# Patients taking other transport	# Patients in Definitive Care on other Transport	# Patients in Definitive Care	# Patients receiving Lower Quality Injury Care	Patients Perception of Health System	Patients trust in healthcare system	Willingness to seek formal care	# Injured seeking care	# Patients taking an ambulance				
596	16	Socioeconomic Determinant Loop	Total available workforce	GDP	Poverty	Community Conflict Violence etc	Availability of quality injury care	Discrimination	# Patients receiving Lower Quality Injury Care	Patients Perception of Health System	Patients trust in healthcare system	# Injured seeking non-formal care	Willingness to seek formal care	# Injured seeking care	# Patients taking an ambulance	# Patients in Definitive Care on Ambulance	# Patients in Definitive care Delayed				
597	16	Socioeconomic Determinant Loop	Total available workforce	GDP	System	Available Funding	Availability	# Patients receiving	Patients Perceptio	Patients trust in	Willingnes s to seek	# Injured	# Patients taking	# Patients in Definitive	# Patients in	# Patients remaining	# Patients Receiving				

					level funding	for injury care	of quality injury care	Lower Quality Injury Care	n of Health System	healthcare system	formal care	seeking care	other transport	Care on other Transport	Definitive Care	in care/rehab	Higher Quality Rehab				
598	16	Socioeconomic Determinant Loop	Total available workforce	GDP	Poverty	Community Conflict Violence etc	Availability of quality injury rehab or follow-up	Discrimination	# Patients receiving Lower Quality Injury Care	Patients Perception of Health System	Patients trust in healthcare system	Willingness to seek formal care	# Injured seeking care	# Patients taking an ambulance	# Patients in Definitive Care on Ambulance	# Patients in Definitive Care	# Patients Receiving Higher Quality Injury care				
599	16	Socioeconomic Determinant Loop	Total available workforce	GDP	System level funding	Available Funding for injury care	Availability of quality injury rehab or follow-up	Discrimination	# Patients Receiving Higher Quality Injury care	Patients Perception of Health System	Patients trust in healthcare system	Willingness to receive care	# Patients in Definitive Care	# Patients receiving Lower Quality Injury Care	Willingness to remain in care	# Patients remaining in care/rehab	# Patients Receiving Higher Quality Rehab				
600	16	Socioeconomic Determinant Loop	Total available workforce	GDP	System level funding	Available Funding for injury care	Universal health insurance	Ability to pay	Willingness to seek formal care	# Injured seeking care	# Patients taking an ambulance	# Patients in Definitive Care on Ambulance	# Patients in Definitive Care	# Patients receiving Lower Quality Injury Care	Patients Perception of Health System	Patients trust in healthcare system	# Injured seeking non-formal care				
601	16	Socioeconomic Determinant Loop	Total available workforce	GDP	System level funding	Available Funding for injury care	Availability of quality injury care	Discrimination	# Patients receiving Lower Quality Injury Care	Patients Perception of Health System	Patients trust in healthcare system	# Injured seeking non-formal care	Willingness to seek formal care	# Injured seeking care	# Patients taking other transport	# Patients in Definitive Care on other Transport	# Patients in Definitive care Delayed				
602	16	Socioeconomic Determinant Loop	Total available workforce	GDP	Poverty	Community Conflict Violence etc	Availability of quality injury care	Discrimination	# Patients receiving Lower Quality Injury Care	Patients Perception of Health System	Patients trust in healthcare system	Willingness to seek formal care	# Injured seeking care	# Patients taking an ambulance	# Patients in Definitive Care on Ambulance	# Patients in Definitive Care	# Patients Receiving Higher Quality Injury care				
603	16	Socioeconomic Determinant Loop	Total available workforce	GDP	Poverty	Community Conflict Violence etc	Availability of quality injury rehab or follow-up	Discrimination	# Patients Receiving Higher Quality Injury care	Patients Perception of Health System	Patients trust in healthcare system	Willingness to receive care	# Patients in Definitive Care	# Patients receiving Lower Quality Injury Care	Willingness to remain in care	# Patients remaining in care/rehab	# Patients receiving Lower Quality Rehab				
604	16	Socioeconomic Determinant Loop	Total available workforce	GDP	Poverty	Community Conflict Violence etc	Availability of quality injury rehab or follow-up	Discrimination	# Patients receiving Lower Quality Injury Care	Patients Perception of Health System	Patients trust in healthcare system	Willingness to seek formal care	# Injured seeking care	# Patients taking other transport	# Patients in Definitive Care on other Transport	# Patients in Definitive Care	# Patients Receiving Higher Quality Injury care				
605	16	Socioeconomic Determinant Loop	Total available workforce	GDP	System level funding	Available Funding for injury care	Universal health insurance	Ability to pay	Willingness to seek formal care	# Injured seeking care	# Patients taking an ambulance	# Patients in Definitive Care on Ambulance	# Patients in Definitive Care	# Patients receiving Lower Quality Injury Care	Willingness to remain in care	# Patients remaining in care/rehab	# Patients receiving Lower Quality Rehab				
606	16	Socioeconomic Determinant Loop	Total available workforce	GDP	Poverty	Community Conflict Violence etc	Availability of quality injury rehab or follow-up	Discrimination	# Patients receiving Lower Quality Injury Care	Patients Perception of Health System	Patients trust in healthcare system	Willingness to receive care	# Patients in Definitive Care	# Patients Receiving Higher Quality Injury care	Willingness to remain in care	# Patients remaining in care/rehab	# Patients Receiving Higher Quality Rehab				
607	16	Socioeconomic Determinant Loop	Total available workforce	GDP	System level funding	Available Funding for injury care	Availability of quality injury rehab or follow-up	Discrimination	# Patients receiving Lower Quality Injury Care	Patients Perception of Health System	Patients trust in healthcare system	# Injured seeking non-formal care	Willingness to seek formal care	# Injured seeking care	# Patients taking other transport	# Patients in Definitive Care on other Transport	# Patients in Definitive care Delayed				
608	16	Socioeconomic Determinant Loop	Total available workforce	GDP	Poverty	Individual wealth	Availability of other transport	# Patients taking other transport	# Patients in Definitive Care on other Transport	# Patients in Definitive Care	# Patients Receiving Higher Quality Injury care	Patients Perception of Health System	Patients trust in healthcare system	# Injured seeking non-formal care	Willingness to seek formal care	# Injured seeking care	# Patients taking an ambulance				
609	16	Socioeconomic Determinant Loop	Total available workforce	GDP	System level funding	Available Funding for injury care	Availability of quality injury rehab or follow-up	Discrimination	# Patients Receiving Higher Quality Injury care	Patients Perception of Health System	Patients trust in healthcare system	Willingness to seek formal care	# Injured seeking care	# Patients taking other transport	# Patients in Definitive Care on other Transport	# Patients in Definitive Care	# Patients receiving Lower Quality Injury Care				

610	16	Socioeconomic Determinant Loop	Total available workforce	GDP	System level funding	Available Funding for injury care	Universal health insurance	Ability to pay	Willingness to seek formal care	# Injured seeking care	# Patients taking an ambulance	# Patients in Definitive Care on Ambulance	# Patients in Definitive Care	# Patients Receiving Higher Quality Injury care	Willingness to remain in care	# Patients remaining in care/rehab	# Patients Receiving Higher Quality Rehab				
611	16	Socioeconomic Determinant Loop	Total available workforce	GDP	System level funding	Available Funding for injury care	Universal health insurance	Ability to pay	Willingness to seek formal care	# Injured seeking care	# Patients taking other transport	# Patients in Definitive Care on other Transport	# Patients in Definitive Care	# Patients Receiving Higher Quality Injury care	Patients Perception of Health System	Patients trust in healthcare system	# Injured seeking non-formal care				
612	16	Socioeconomic Determinant Loop	Total available workforce	GDP	Poverty	Community Conflict Violence etc	Availability of quality injury rehab or follow-up	Discrimination	# Patients receiving Lower Quality Injury Care	Patients Perception of Health System	Patients trust in healthcare system	# Injured seeking non-formal care	Willingness to seek formal care	# Injured seeking care	# Patients taking other transport	# Patients in Definitive Care on other Transport	# Patients in Definitive care on time				
613	16	Socioeconomic Determinant Loop	Total available workforce	GDP	System level funding	Available Funding for injury care	Availability of quality injury rehab or follow-up	Discrimination	# Patients receiving Lower Quality Injury Care	Patients Perception of Health System	Patients trust in healthcare system	Willingness to receive care	# Patients in Definitive Care	# Patients Receiving Higher Quality Injury care	Willingness to remain in care	# Patients remaining in care/rehab	# Patients Receiving Higher Quality Rehab				
614	16	Socioeconomic Determinant Loop	Total available workforce	GDP	Poverty	Individual wealth	Ability to pay	Willingness to receive care	# Patients in Definitive Care	# Patients receiving Lower Quality Injury Care	Patients Perception of Health System	Patients trust in healthcare system	Willingness to seek formal care	# Injured seeking care	# Patients taking an ambulance	# Patients in Definitive Care on Ambulance	# Patients in Definitive care on time				
615	16	Socioeconomic Determinant Loop	Total available workforce	GDP	Poverty	Individual wealth	Ability to pay	Willingness to receive care	# Patients in Definitive Care	# Patients receiving Lower Quality Injury Care	Patients Perception of Health System	Patients trust in healthcare system	Willingness to seek formal care	# Injured seeking care	# Patients taking an ambulance	# Patients in Definitive Care on Ambulance	# Patients in Definitive care Delayed				
616	16	Socioeconomic Determinant Loop	Total available workforce	GDP	System level funding	Available Funding for injury care	Availability of quality injury rehab or follow-up	Discrimination	# Patients receiving Lower Quality Injury Care	Patients Perception of Health System	Patients trust in healthcare system	# Injured seeking non-formal care	Willingness to seek formal care	# Injured seeking care	# Patients taking other transport	# Patients in Definitive Care on other Transport	# Patients in Definitive care on time				
617	16	Socioeconomic Determinant Loop	Total available workforce	GDP	System level funding	Available Funding for injury care	Availability of quality injury care	Discrimination	# Patients Receiving Higher Quality Injury care	Patients Perception of Health System	Patients trust in healthcare system	Willingness to seek formal care	# Injured seeking care	# Patients taking an ambulance	# Patients in Definitive Care on Ambulance	# Patients in Definitive Care	# Patients receiving Lower Quality Injury Care				
618	16	Socioeconomic Determinant Loop	Total available workforce	GDP	Poverty	Community Conflict Violence etc	Geographical accessibility	Availability of Ambulance Service	# Patients taking an ambulance	# Patients in Definitive Care on Ambulance	# Patients in Definitive Care	# Patients Receiving Higher Quality Injury care	Patients Perception of Health System	Patients trust in healthcare system	Willingness to remain in care	# Patients remaining in care/rehab	# Patients Receiving Higher Quality Rehab				
619	16	Socioeconomic Determinant Loop	Total available workforce	GDP	System level funding	Available Funding for injury care	Availability of quality injury rehab or follow-up	Discrimination	# Patients receiving Lower Quality Injury Care	Patients Perception of Health System	Patients trust in healthcare system	# Injured seeking non-formal care	Willingness to seek formal care	# Injured seeking care	# Patients taking an ambulance	# Patients in Definitive Care on Ambulance	# Patients in Definitive care on time				
620	16	Socioeconomic Determinant Loop	Total available workforce	GDP	System level funding	Available Funding for injury care	Availability of quality injury care	# Patients Receiving Higher Quality Injury care	Patients Perception of Health System	Patients trust in healthcare system	Willingness to seek formal care	# Injured seeking care	# Patients taking other transport	# Patients in Definitive Care on other Transport	# Patients in Definitive Care	# Patients remaining in care/rehab	# Patients Receiving Higher Quality Rehab				
621	16	Socioeconomic Determinant Loop	Total available workforce	GDP	System level funding	Available Funding for injury care	Availability of quality injury care	Discrimination	# Patients receiving Lower Quality Injury Care	Patients Perception of Health System	Patients trust in healthcare system	# Injured seeking non-formal care	Willingness to seek formal care	# Injured seeking care	# Patients taking other transport	# Patients in Definitive Care on other Transport	# Patients in Definitive care on time				
622	16	Socioeconomic Determinant Loop	Total available workforce	GDP	System level funding	Available Funding for injury care	Universal health insurance	Ability to pay	Willingness to seek formal care	# Injured seeking care	# Patients taking an ambulance	# Patients in Definitive Care on Ambulance	# Patients in Definitive Care	# Patients Receiving Higher Quality Injury care	Willingness to remain in care	# Patients remaining in care/rehab	# Patients receiving Lower Quality Rehab				

623	16	Socioeconomic Determinant Loop	Total available workforce	GDP	Poverty	Community Conflict Violence etc	Availability of quality injury rehab or follow-up	Discrimination	# Patients receiving Lower Quality Injury Care	Patients Perception of Health System	Patients trust in healthcare system	Willingness to receive care	# Patients in Definitive Care	# Patients Receiving Higher Quality Injury care	Willingness to remain in care	# Patients remaining in care/rehab	# Patients receiving Lower Quality Rehab				
624	16	Socioeconomic Determinant Loop	Total available workforce	GDP	Poverty	Community Conflict Violence etc	Availability of quality injury care	Discrimination	# Patients Receiving Higher Quality Injury care	Patients Perception of Health System	Patients trust in healthcare system	# Injured seeking non-formal care	Willingness to seek formal care	# Injured seeking care	# Patients taking an ambulance	# Patients in Definitive Care on Ambulance	# Patients in Definitive care on time				
625	16	Socioeconomic Determinant Loop	Total available workforce	GDP	Poverty	Community Conflict Violence etc	Availability of quality injury care	Discrimination	# Patients Receiving Higher Quality Injury care	Patients Perception of Health System	Patients trust in healthcare system	Willingness to seek formal care	# Injured seeking care	# Patients taking other transport	# Patients in Definitive Care on other Transport	# Patients in Definitive Care	# Patients receiving Lower Quality Injury Care				
626	16	Socioeconomic Determinant Loop	Total available workforce	GDP	Poverty	Community Conflict Violence etc	Availability of quality injury care	Discrimination	# Patients receiving Lower Quality Injury Care	Patients Perception of Health System	Patients trust in healthcare system	Willingness to receive care	# Patients in Definitive Care	# Patients Receiving Higher Quality Injury care	Willingness to remain in care	# Patients remaining in care/rehab	# Patients Receiving Higher Quality Rehab				
627	16	Socioeconomic Determinant Loop	Total available workforce	GDP	System level funding	Available Funding for injury care	Availability of quality injury care	# Patients Receiving Higher Quality Injury care	Patients Perception of Health System	Patients trust in healthcare system	Willingness to seek formal care	# Injured seeking care	# Patients taking other transport	# Patients in Definitive Care on other Transport	# Patients in Definitive Care	# Patients remaining in care/rehab	# Patients receiving Lower Quality Rehab				
628	16	Socioeconomic Determinant Loop	Total available workforce	GDP	System level funding	Available Funding for injury care	Availability of quality injury rehab or follow-up	Discrimination	# Patients receiving Lower Quality Injury Care	Patients Perception of Health System	Patients trust in healthcare system	Willingness to seek formal care	# Injured seeking care	# Patients taking other transport	# Patients in Definitive Care on other Transport	# Patients in Definitive Care	# Patients Receiving Higher Quality Injury care				
629	16	Socioeconomic Determinant Loop	Total available workforce	GDP	System level funding	Available Funding for injury care	Availability of quality injury rehab or follow-up	Discrimination	# Patients Receiving Higher Quality Injury care	Patients Perception of Health System	Patients trust in healthcare system	Willingness to receive care	# Patients in Definitive Care	# Patients receiving Lower Quality Injury Care	Willingness to remain in care	# Patients remaining in care/rehab	# Patients receiving Lower Quality Rehab				
630	16	Socioeconomic Determinant Loop	Total available workforce	GDP	Poverty	Community Conflict Violence etc	Availability of quality injury care	Discrimination	# Patients receiving Lower Quality Injury Care	Patients Perception of Health System	Patients trust in healthcare system	Willingness to receive care	# Patients in Definitive Care	# Patients Receiving Higher Quality Injury care	Willingness to remain in care	# Patients remaining in care/rehab	# Patients receiving Lower Quality Rehab				
631	17	Socioeconomic Determinant Loop	Total available workforce	GDP	System level funding	Available Funding for injury care	Availability of quality injury rehab or follow-up	Discrimination	# Patients Receiving Higher Quality Injury care	Patients Perception of Health System	Patients trust in healthcare system	Willingness to seek formal care	# Injured seeking care	# Patients taking an ambulance	# Patients in Definitive Care on Ambulance	# Patients in Definitive Care	# Patients remaining in care/rehab	# Patients Receiving Higher Quality Rehab			
632	17	Socioeconomic Determinant Loop	Total available workforce	GDP	System level funding	Available Funding for injury care	Availability of quality injury care	Discrimination	# Patients Receiving Higher Quality Injury care	Patients Perception of Health System	Patients trust in healthcare system	# Injured seeking non-formal care	Willingness to seek formal care	# Injured seeking care	# Patients taking an ambulance	# Patients in Definitive Care on Ambulance	# Patients in Definitive Care	# Patients receiving Lower Quality Injury Care			
633	17	Socioeconomic Determinant Loop	Total available workforce	GDP	System level funding	Available Funding for injury care	Availability of Ambulance Service	Geographical accessibility	Availability of other transport	# Patients taking other transport	# Patients in Definitive Care on other Transport	# Patients in Definitive Care	# Patients Receiving Higher Quality Injury care	Patients Perception of Health System	Patients trust in healthcare system	# Injured seeking non-formal care	Willingness to seek formal care	# Injured seeking care			
634	17	Socioeconomic Determinant Loop	Total available workforce	GDP	Poverty	Community Conflict Violence etc	Availability of quality injury rehab or follow-up	Discrimination	# Patients receiving Lower Quality Injury Care	Patients Perception of Health System	Patients trust in healthcare system	Willingness to seek formal care	# Injured seeking care	# Patients taking other transport	# Patients in Definitive Care on other Transport	# Patients in Definitive Care	# Patients remaining in care/rehab	# Patients Receiving Higher Quality Rehab			
635	17	Socioeconomic Determinant Loop	Total available workforce	GDP	Poverty	Individual wealth	Ability to pay	Willingness to seek formal care	# Injured seeking care	# Patients taking other transport	# Patients in Definitive Care on other Transport	# Patients in Definitive Care	# Patients receiving Lower Quality Injury Care	Patients Perception of Health System	Patients trust in healthcare system	Willingness to remain in care	# Patients remaining in care/rehab	# Patients receiving Lower Quality Rehab			

636	17	Socioeconomic Determinant Loop	Total available workforce	GDP	System level funding	Available Funding for injury care	Availability of quality injury rehab or follow-up	Discrimination	# Patients receiving Lower Quality Injury Care	Patients Perception of Health System	Patients trust in healthcare system	Willingness to seek formal care	# Injured seeking care	# Patients taking an ambulance	# Patients in Definitive Care on Ambulance	# Patients in Definitive Care	# Patients remaining in care/rehab	# Patients Receiving Higher Quality Rehab			
637	17	Socioeconomic Determinant Loop	Total available workforce	GDP	Poverty	Individual wealth	Ability to pay	Willingness to seek formal care	# Injured seeking care	# Patients taking other transport	# Patients in Definitive Care on other Transport	# Patients in Definitive Care	# Patients Receiving Higher Quality Injury care	Patients Perception of Health System	Patients trust in healthcare system	Willingness to remain in care	# Patients remaining in care/rehab	# Patients Receiving Higher Quality Rehab			
638	17	Socioeconomic Determinant Loop	Total available workforce	GDP	Poverty	Community Conflict Violence etc	Availability of quality injury rehab or follow-up	Discrimination	# Patients Receiving Higher Quality Injury care	Patients Perception of Health System	Patients trust in healthcare system	Willingness to seek formal care	# Injured seeking care	# Patients taking other transport	# Patients in Definitive Care on other Transport	# Patients in Definitive Care	# Patients remaining in care/rehab	# Patients receiving Lower Quality Rehab			
639	17	Socioeconomic Determinant Loop	Total available workforce	GDP	System level funding	Available Funding for injury care	Universal health insurance	Ability to pay	Willingness to receive care	# Patients in Definitive Care	# Patients Receiving Higher Quality Injury care	Patients Perception of Health System	Patients trust in healthcare system	Willingness to seek formal care	# Injured seeking care	# Patients taking other transport	# Patients in Definitive Care on other Transport	# Patients in Definitive care Delayed			
640	17	Socioeconomic Determinant Loop	Total available workforce	GDP	System level funding	Available Funding for injury care	Availability of quality injury care	Discrimination	# Patients receiving Lower Quality Injury Care	Patients Perception of Health System	Patients trust in healthcare system	# Injured seeking non-formal care	Willingness to seek formal care	# Injured seeking care	# Patients taking other transport	# Patients in Definitive Care on other Transport	# Patients in Definitive Care	# Patients Receiving Higher Quality Injury care			
641	17	Socioeconomic Determinant Loop	Total available workforce	GDP	System level funding	Available Funding for injury care	Availability of quality injury rehab or follow-up	Discrimination	# Patients Receiving Higher Quality Injury care	Patients Perception of Health System	Patients trust in healthcare system	Willingness to seek formal care	# Injured seeking care	# Patients taking other transport	# Patients in Definitive Care on other Transport	# Patients in Definitive Care	# Patients remaining in care/rehab	# Patients Receiving Higher Quality Rehab			
642	17	Socioeconomic Determinant Loop	Total available workforce	GDP	Poverty	Individual wealth	Ability to pay	Willingness to seek formal care	# Injured seeking care	# Patients taking other transport	# Patients in Definitive Care on other Transport	# Patients in Definitive Care	# Patients receiving Lower Quality Injury Care	Patients Perception of Health System	Patients trust in healthcare system	Willingness to remain in care	# Patients remaining in care/rehab	# Patients Receiving Higher Quality Rehab			
643	17	Socioeconomic Determinant Loop	Total available workforce	GDP	System level funding	Available Funding for injury care	Availability of quality injury rehab or follow-up	Discrimination	# Patients Receiving Higher Quality Injury care	Patients Perception of Health System	Patients trust in healthcare system	# Injured seeking non-formal care	Willingness to seek formal care	# Injured seeking care	# Patients taking other transport	# Patients in Definitive Care on other Transport	# Patients in Definitive Care	# Patients receiving Lower Quality Injury Care			
644	17	Socioeconomic Determinant Loop	Total available workforce	GDP	Poverty	Community Conflict Violence etc	Availability of quality injury care	Discrimination	# Patients receiving Lower Quality Injury Care	Patients Perception of Health System	Patients trust in healthcare system	Willingness to seek formal care	# Injured seeking care	# Patients taking an ambulance	# Patients in Definitive Care on Ambulance	# Patients in Definitive Care	# Patients remaining in care/rehab	# Patients Receiving Higher Quality Rehab			
645	17	Socioeconomic Determinant Loop	Total available workforce	GDP	System level funding	Available Funding for injury care	Universal health insurance	Ability to pay	Willingness to receive care	# Patients in Definitive Care	# Patients Receiving Higher Quality Injury care	Patients Perception of Health System	Patients trust in healthcare system	Willingness to seek formal care	# Injured seeking care	# Patients taking other transport	# Patients in Definitive Care on other Transport	# Patients in Definitive care on time			
646	17	Socioeconomic Determinant Loop	Total available workforce	GDP	Poverty	Community Conflict Violence etc	Availability of quality injury care	Discrimination	# Patients Receiving Higher Quality Injury care	Patients Perception of Health System	Patients trust in healthcare system	Willingness to seek formal care	# Injured seeking care	# Patients taking an ambulance	# Patients in Definitive Care on Ambulance	# Patients in Definitive Care	# Patients remaining in care/rehab	# Patients Receiving Higher Quality Rehab			
647	17	Socioeconomic Determinant Loop	Total available workforce	GDP	System level funding	Available Funding for injury care	Availability of quality injury rehab or follow-up	Discrimination	# Patients receiving Lower Quality Injury Care	Patients Perception of Health System	Patients trust in healthcare system	# Injured seeking non-formal care	Willingness to seek formal care	# Injured seeking care	# Patients taking other transport	# Patients in Definitive Care on other Transport	# Patients in Definitive Care	# Patients Receiving Higher Quality Injury care			
648	17	Socioeconomic Determinant Loop	Total available workforce	GDP	System level funding	Available Funding for injury care	Availability of quality injury rehab or follow-up	Discrimination	# Patients Receiving Higher Quality Injury care	Patients Perception of Health System	Patients trust in healthcare system	Willingness to seek formal care	# Injured seeking care	# Patients taking other transport	# Patients in Definitive Care on other Transport	# Patients in Definitive Care	# Patients remaining in care/rehab	# Patients receiving Lower Quality Rehab			
649	17	Socioeconomic Determinant Loop	Total available workforce	GDP	Poverty	Individual wealth	Ability to pay	Willingness	# Patients in	# Patients receiving	Patients Perceptio	Patients trust in	# Injured seeking	Willingness to seek	# Injured	# Patients taking	# Patients in Definitive	# Patients in Definitive			

								to receive care	Definitive Care	Lower Quality Injury Care	n of Health System	healthcare system	non-formal care	formal care	seeking care	other transport	Care on other Transport	care Delayed			
650	17	Socioeconomic Determinant Loop	Total available workforce	GDP	System level funding	Available Funding for injury care	Availability of quality injury care	Discrimination	# Patients Receiving Higher Quality Injury care	Patients Perception of Health System	Patients trust in healthcare system	# Injured seeking non-formal care	Willingness to seek formal care	# Injured seeking care	# Patients taking other transport	# Patients in Definitive Care on other Transport	# Patients in Definitive Care	# Patients receiving Lower Quality Injury Care			
651	17	Socioeconomic Determinant Loop	Total available workforce	GDP	Poverty	Individual wealth	Ability to pay	Willingness to seek formal care	# Injured seeking care	# Patients taking an ambulance	# Patients in Definitive Care on Ambulance	# Patients in Definitive Care	# Patients Receiving Higher Quality Injury care	Patients Perception of Health System	Patients trust in healthcare system	Willingness to remain in care	# Patients remaining in care/rehab	# Patients receiving Lower Quality Rehab			
652	17	Socioeconomic Determinant Loop	Total available workforce	GDP	Poverty	Individual wealth	Ability to pay	Willingness to receive care	# Patients in Definitive Care	# Patients receiving Lower Quality Injury Care	Patients Perception of Health System	Patients trust in healthcare system	# Injured seeking non-formal care	Willingness to seek formal care	# Injured seeking care	# Patients taking other transport	# Patients in Definitive Care on other Transport	# Patients in Definitive care on time			
653	17	Socioeconomic Determinant Loop	Total available workforce	GDP	Poverty	Individual wealth	Availability of other transport	Geographical accessibility	Availability of Ambulance Service	# Patients taking an ambulance	# Patients in Definitive Care on Ambulance	# Patients in Definitive Care	# Patients receiving Lower Quality Injury Care	Patients Perception of Health System	Patients trust in healthcare system	# Injured seeking non-formal care	Willingness to seek formal care	# Injured seeking care			
654	17	Socioeconomic Determinant Loop	Total available workforce	GDP	System level funding	Available Funding for injury care	Availability of quality injury rehab or follow-up	Discrimination	# Patients receiving Lower Quality Injury Care	Patients Perception of Health System	Patients trust in healthcare system	Willingness to seek formal care	# Injured seeking care	# Patients taking other transport	# Patients in Definitive Care on other Transport	# Patients in Definitive Care	# Patients remaining in care/rehab	# Patients Receiving Higher Quality Rehab			
655	17	Socioeconomic Determinant Loop	Total available workforce	GDP	Poverty	Community Conflict Violence etc	Availability of quality injury care	Discrimination	# Patients Receiving Higher Quality Injury care	Patients Perception of Health System	Patients trust in healthcare system	Willingness to seek formal care	# Injured seeking care	# Patients taking an ambulance	# Patients in Definitive Care on Ambulance	# Patients in Definitive Care	# Patients remaining in care/rehab	# Patients receiving Lower Quality Rehab			
656	17	Socioeconomic Determinant Loop	Total available workforce	GDP	Poverty	Individual wealth	Ability to pay	Willingness to receive care	# Patients in Definitive Care	# Patients Receiving Higher Quality Injury care	Patients Perception of Health System	Patients trust in healthcare system	# Injured seeking non-formal care	Willingness to seek formal care	# Injured seeking care	# Patients taking an ambulance	# Patients in Definitive Care on Ambulance	# Patients in Definitive care Delayed			
657	17	Socioeconomic Determinant Loop	Total available workforce	GDP	System level funding	Available Funding for injury care	Availability of quality injury rehab or follow-up	Discrimination	# Patients Receiving Higher Quality Injury care	Patients Perception of Health System	Patients trust in healthcare system	Willingness to seek formal care	# Injured seeking care	# Patients taking an ambulance	# Patients in Definitive Care on Ambulance	# Patients in Definitive Care	# Patients remaining in care/rehab	# Patients receiving Lower Quality Rehab			
658	17	Socioeconomic Determinant Loop	Total available workforce	GDP	Poverty	Individual wealth	Availability of other transport	Geographical accessibility	Availability of Ambulance Service	# Patients taking an ambulance	# Patients in Definitive Care on Ambulance	# Patients in Definitive Care	# Patients Receiving Higher Quality Injury care	Patients Perception of Health System	Patients trust in healthcare system	Willingness to remain in care	# Patients remaining in care/rehab	# Patients Receiving Higher Quality Rehab			
659	17	Socioeconomic Determinant Loop	Total available workforce	GDP	System level funding	Available Funding for injury care	Universal health insurance	Ability to pay	Willingness to receive care	# Patients in Definitive Care	# Patients Receiving Higher Quality Injury care	Patients Perception of Health System	Patients trust in healthcare system	Willingness to seek formal care	# Injured seeking care	# Patients taking an ambulance	# Patients in Definitive Care on Ambulance	# Patients in Definitive care on time			
660	17	Socioeconomic Determinant Loop	Total available workforce	GDP	Poverty	Community Conflict Violence etc	Availability of quality injury care	Discrimination	# Patients Receiving Higher Quality Injury care	Patients Perception of Health System	Patients trust in healthcare system	Willingness to seek formal care	# Injured seeking care	# Patients taking other transport	# Patients in Definitive Care on other Transport	# Patients in Definitive Care	# Patients remaining in care/rehab	# Patients receiving Lower Quality Rehab			
661	17	Socioeconomic Determinant Loop	Total available workforce	GDP	Poverty	Community Conflict Violence etc	Availability of quality injury care	Discrimination	# Patients receiving Lower Quality Injury Care	Patients Perception of Health System	Patients trust in healthcare system	Willingness to seek formal care	# Injured seeking care	# Patients taking other transport	# Patients in Definitive Care on other Transport	# Patients in Definitive Care	# Patients remaining in care/rehab	# Patients Receiving Higher Quality Rehab			
662	17	Socioeconomic Determinant Loop	Total available workforce	GDP	Poverty	Community Conflict Violence etc	Availability of quality injury care	# Patients Receiving Higher Quality Injury care	Patients Perception of Health System	Patients trust in healthcare system	# Injured seeking non-formal care	Willingness to seek formal care	# Injured seeking care	# Patients taking an ambulance	# Patients in Definitive Care on Ambulance	# Patients in Definitive Care	# Patients remaining in care/rehab	# Patients Receiving Higher Quality Rehab			

663	17	Socioeconomic Determinant Loop	Total available workforce	GDP	Poverty	Community Conflict Violence etc	Availability of quality injury care	# Patients receiving Lower Quality Injury Care	Patients Perception of Health System	Patients trust in healthcare system	# Injured seeking non-formal care	Willingness to seek formal care	# Injured seeking care	# Patients taking an ambulance	# Patients in Definitive Care on Ambulance	# Patients in Definitive Care	# Patients remaining in care/rehab	# Patients Receiving Higher Quality Rehab			
664	17	Socioeconomic Determinant Loop	Total available workforce	GDP	Poverty	Community Conflict Violence etc	Availability of quality injury care	Discrimination	# Patients Receiving Higher Quality Injury care	Patients Perception of Health System	Patients trust in healthcare system	Willingness to seek formal care	# Injured seeking care	# Patients taking other transport	# Patients in Definitive Care on other Transport	# Patients in Definitive Care	# Patients remaining in care/rehab	# Patients Receiving Higher Quality Rehab			
665	17	Socioeconomic Determinant Loop	Total available workforce	GDP	Poverty	Community Conflict Violence etc	Availability of quality injury rehab or follow-up	Discrimination	# Patients receiving Lower Quality Injury Care	Patients Perception of Health System	Patients trust in healthcare system	Willingness to seek formal care	# Injured seeking care	# Patients taking an ambulance	# Patients in Definitive Care on Ambulance	# Patients in Definitive Care	# Patients remaining in care/rehab	# Patients receiving Lower Quality Rehab			
666	17	Socioeconomic Determinant Loop	Total available workforce	GDP	Poverty	Community Conflict Violence etc	Availability of quality injury care	Discrimination	# Patients Receiving Higher Quality Injury care	Patients Perception of Health System	Patients trust in healthcare system	# Injured seeking non-formal care	Willingness to seek formal care	# Injured seeking care	# Patients taking other transport	# Patients in Definitive Care on other Transport	# Patients in Definitive Care	# Patients receiving Lower Quality Injury Care			
667	17	Socioeconomic Determinant Loop	Total available workforce	GDP	Poverty	Individual wealth	Availability of other transport	Geographical accessibility	Availability of Ambulance Service	# Patients taking an ambulance	# Patients in Definitive Care on Ambulance	# Patients in Definitive Care	# Patients receiving Lower Quality Injury Care	Patients Perception of Health System	Patients trust in healthcare system	Willingness to remain in care	# Patients remaining in care/rehab	# Patients Receiving Higher Quality Rehab			
668	17	Socioeconomic Determinant Loop	Total available workforce	GDP	Poverty	Community Conflict Violence etc	Availability of quality injury rehab or follow-up	Discrimination	# Patients receiving Lower Quality Injury Care	Patients Perception of Health System	Patients trust in healthcare system	Willingness to seek formal care	# Injured seeking care	# Patients taking other transport	# Patients in Definitive Care on other Transport	# Patients in Definitive Care	# Patients remaining in care/rehab	# Patients receiving Lower Quality Rehab			
669	17	Socioeconomic Determinant Loop	Total available workforce	GDP	System level funding	Available Funding for injury care	Availability of Ambulance Service	Geographical accessibility	Availability of other transport	# Patients taking other transport	# Patients in Definitive Care on other Transport	# Patients in Definitive Care	# Patients Receiving Higher Quality Injury care	Patients Perception of Health System	Patients trust in healthcare system	Willingness to seek formal care	# Injured seeking care	# Patients taking an ambulance			
670	17	Socioeconomic Determinant Loop	Total available workforce	GDP	Poverty	Community Conflict Violence etc	Availability of quality injury care	# Patients receiving Lower Quality Injury Care	Patients Perception of Health System	Patients trust in healthcare system	# Injured seeking non-formal care	Willingness to seek formal care	# Injured seeking care	# Patients taking other transport	# Patients in Definitive Care on other Transport	# Patients in Definitive Care	# Patients remaining in care/rehab	# Patients Receiving Higher Quality Rehab			
671	17	Socioeconomic Determinant Loop	Total available workforce	GDP	System level funding	Available Funding for injury care	Availability of quality injury rehab or follow-up	Discrimination	# Patients receiving Lower Quality Injury Care	Patients Perception of Health System	Patients trust in healthcare system	Willingness to seek formal care	# Injured seeking care	# Patients taking an ambulance	# Patients in Definitive Care on Ambulance	# Patients in Definitive Care	# Patients remaining in care/rehab	# Patients receiving Lower Quality Rehab			
672	17	Socioeconomic Determinant Loop	Total available workforce	GDP	Poverty	Community Conflict Violence etc	Geographical accessibility	Availability of other transport	# Patients taking other transport	# Patients in Definitive Care on other Transport	# Patients in Definitive Care	# Patients receiving Lower Quality Injury Care	Patients Perception of Health System	Patients trust in healthcare system	# Injured seeking non-formal care	Willingness to seek formal care	# Injured seeking care	# Patients taking an ambulance			
673	17	Socioeconomic Determinant Loop	Total available workforce	GDP	Poverty	Community Conflict Violence etc	Availability of quality injury care	Discrimination	# Patients receiving Lower Quality Injury Care	Patients Perception of Health System	Patients trust in healthcare system	# Injured seeking non-formal care	Willingness to seek formal care	# Injured seeking care	# Patients taking other transport	# Patients in Definitive Care on other Transport	# Patients in Definitive Care	# Patients Receiving Higher Quality Injury care			
674	17	Socioeconomic Determinant Loop	Total available workforce	GDP	System level funding	Available Funding for injury care	Availability of quality injury care	# Patients Receiving Higher Quality Injury care	Patients Perception of Health System	Patients trust in healthcare system	# Injured seeking non-formal care	Willingness to seek formal care	# Injured seeking care	# Patients taking other transport	# Patients in Definitive Care on other Transport	# Patients in Definitive Care	# Patients remaining in care/rehab	# Patients receiving Lower Quality Rehab			
675	17	Socioeconomic Determinant Loop	Total available workforce	GDP	System level funding	Available Funding for injury care	Availability of quality injury rehab or follow-up	Discrimination	# Patients receiving Lower Quality Injury Care	Patients Perception of Health System	Patients trust in healthcare system	Willingness to seek formal care	# Injured seeking care	# Patients taking other transport	# Patients in Definitive Care on other Transport	# Patients in Definitive Care	# Patients remaining in care/rehab	# Patients receiving Lower Quality Rehab			

676	17	Socioeconomic Determinant Loop	Total available workforce	GDP	Poverty	Community Conflict Violence etc	Availability of quality injury care	Discrimination	# Patients receiving Lower Quality Injury Care	Patients Perception of Health System	Patients trust in healthcare system	Willingness to seek formal care	# Injured seeking care	# Patients taking other transport	# Patients in Definitive Care on other Transport	# Patients in Definitive Care	# Patients remaining in care/rehab	# Patients receiving Lower Quality Rehab			
677	17	Socioeconomic Determinant Loop	Total available workforce	GDP	System level funding	Available Funding for injury care	Availability of quality injury care	Discrimination	# Patients Receiving Higher Quality Injury care	Patients Perception of Health System	Patients trust in healthcare system	Willingness to seek formal care	# Injured seeking care	# Patients taking an ambulance	# Patients in Definitive Care on Ambulance	# Patients in Definitive Care	# Patients remaining in care/rehab	# Patients Receiving Higher Quality Rehab			
678	17	Socioeconomic Determinant Loop	Total available workforce	GDP	Poverty	Individual wealth	Availability of other transport	# Patients taking other transport	# Patients in Definitive Care on other Transport	# Patients in Definitive Care	# Patients receiving Lower Quality Injury Care	Patients Perception of Health System	Patients trust in healthcare system	Willingness to seek formal care	# Injured seeking care	# Patients taking an ambulance	# Patients in Definitive Care on Ambulance	# Patients in Definitive care Delayed			
679	17	Socioeconomic Determinant Loop	Total available workforce	GDP	Poverty	Individual wealth	Availability of other transport	# Patients taking other transport	# Patients in Definitive Care on other Transport	# Patients in Definitive Care	# Patients receiving Lower Quality Injury Care	Patients Perception of Health System	Patients trust in healthcare system	Willingness to seek formal care	# Injured seeking care	# Patients taking an ambulance	# Patients in Definitive Care on Ambulance	# Patients in Definitive care on time			
680	17	Socioeconomic Determinant Loop	Total available workforce	GDP	Poverty	Individual wealth	Ability to pay	Willingness to receive care	# Patients in Definitive Care	# Patients Receiving Higher Quality Injury care	Patients Perception of Health System	Patients trust in healthcare system	# Injured seeking non-formal care	Willingness to seek formal care	# Injured seeking care	# Patients taking other transport	# Patients in Definitive Care on other Transport	# Patients in Definitive care on time			
681	17	Socioeconomic Determinant Loop	Total available workforce	GDP	Poverty	Community Conflict Violence etc	Availability of quality injury care	Discrimination	# Patients receiving Lower Quality Injury Care	Patients Perception of Health System	Patients trust in healthcare system	Willingness to seek formal care	# Injured seeking care	# Patients taking an ambulance	# Patients in Definitive Care on Ambulance	# Patients in Definitive Care	# Patients remaining in care/rehab	# Patients receiving Lower Quality Rehab			
682	17	Socioeconomic Determinant Loop	Total available workforce	GDP	Poverty	Individual wealth	Ability to pay	Willingness to seek formal care	# Injured seeking care	# Patients taking other transport	# Patients in Definitive Care on other Transport	# Patients in Definitive Care	# Patients Receiving Higher Quality Injury care	Patients Perception of Health System	Patients trust in healthcare system	Willingness to remain in care	# Patients remaining in care/rehab	# Patients receiving Lower Quality Rehab			
683	17	Socioeconomic Determinant Loop	Total available workforce	GDP	Poverty	Individual wealth	Ability to pay	Willingness to receive care	# Patients in Definitive Care	# Patients Receiving Higher Quality Injury care	Patients Perception of Health System	Patients trust in healthcare system	# Injured seeking non-formal care	Willingness to seek formal care	# Injured seeking care	# Patients taking an ambulance	# Patients in Definitive Care on Ambulance	# Patients in Definitive care on time			
684	17	Socioeconomic Determinant Loop	Total available workforce	GDP	System level funding	Available Funding for injury care	Availability of quality injury care	Discrimination	# Patients Receiving Higher Quality Injury care	Patients Perception of Health System	Patients trust in healthcare system	Willingness to seek formal care	# Injured seeking care	# Patients taking other transport	# Patients in Definitive Care on other Transport	# Patients in Definitive Care	# Patients remaining in care/rehab	# Patients Receiving Higher Quality Rehab			
685	17	Socioeconomic Determinant Loop	Total available workforce	GDP	System level funding	Available Funding for injury care	Universal health insurance	Ability to pay	Willingness to receive care	# Patients in Definitive Care	# Patients receiving Lower Quality Injury Care	Patients Perception of Health System	Patients trust in healthcare system	Willingness to seek formal care	# Injured seeking care	# Patients taking an ambulance	# Patients in Definitive Care on Ambulance	# Patients in Definitive care on time			
686	17	Socioeconomic Determinant Loop	Total available workforce	GDP	Poverty	Community Conflict Violence etc	Geographical accessibility	Availability of other transport	# Patients taking other transport	# Patients in Definitive Care on other Transport	# Patients in Definitive Care	# Patients Receiving Higher Quality Injury care	Patients Perception of Health System	Patients trust in healthcare system	# Injured seeking non-formal care	Willingness to seek formal care	# Injured seeking care	# Patients taking an ambulance			
687	17	Socioeconomic Determinant Loop	Total available workforce	GDP	System level funding	Available Funding for injury care	Availability of Ambulance Service	# Patients taking an ambulance	# Patients in Definitive Care on Ambulance	# Patients in Definitive Care	# Patients Receiving Higher Quality Injury care	Patients Perception of Health System	Patients trust in healthcare system	Willingness to seek formal care	# Injured seeking care	# Patients taking other transport	# Patients in Definitive Care on other Transport	# Patients in Definitive care on time			
688	17	Socioeconomic Determinant Loop	Total available workforce	GDP	System level funding	Available Funding for injury care	Availability of Ambulance Service	# Patients taking an ambulance	# Patients in Definitive Care on Ambulance	# Patients in Definitive Care	# Patients Receiving Higher Quality Injury care	Patients Perception of Health System	Patients trust in healthcare system	Willingness to seek formal care	# Injured seeking care	# Patients taking other transport	# Patients in Definitive Care on other Transport	# Patients in Definitive care Delayed			
689	17	Socioeconomic Determinant Loop	Total available workforce	GDP	System level funding	Available Funding	Universal health insurance	Ability to pay	Willingness to	# Patients in Definitive Care	# Patients Receiving Higher	Patients Perception of	Patients trust in	Willingness to seek	# Injured seeking care	# Patients taking an	# Patients in Definitive Care on	# Patients in Definitive			

						for injury care			receive care		Quality Injury care	Health System	healthcare system	formal care		ambulance	Ambulance	care Delayed			
690	17	Socioeconomic Determinant Loop	Total available workforce	GDP	Poverty	Community Conflict Violence etc	Availability of quality injury care	# Patients Receiving Higher Quality Injury care	Patients Perception of Health System	Patients trust in healthcare system	# Injured seeking non-formal care	Willingness to seek formal care	# Injured seeking care	# Patients taking an ambulance	# Patients in Definitive Care on Ambulance	# Patients in Definitive Care	# Patients remaining in care/rehab	# Patients receiving Lower Quality Rehab			
691	17	Socioeconomic Determinant Loop	Total available workforce	GDP	Poverty	Community Conflict Violence etc	Availability of quality injury care	# Patients receiving Lower Quality Injury Care	Patients Perception of Health System	Patients trust in healthcare system	# Injured seeking non-formal care	Willingness to seek formal care	# Injured seeking care	# Patients taking an ambulance	# Patients in Definitive Care on Ambulance	# Patients in Definitive Care	# Patients remaining in care/rehab	# Patients receiving Lower Quality Rehab			
692	17	Socioeconomic Determinant Loop	Total available workforce	GDP	Poverty	Individual wealth	Availability of other transport	# Patients taking other transport	# Patients in Definitive Care on other Transport	# Patients in Definitive Care	# Patients Receiving Higher Quality Injury care	Patients Perception of Health System	Patients trust in healthcare system	Willingness to seek formal care	# Injured seeking care	# Patients taking an ambulance	# Patients in Definitive Care on Ambulance	# Patients in Definitive care Delayed			
693	17	Socioeconomic Determinant Loop	Total available workforce	GDP	Poverty	Individual wealth	Availability of other transport	# Patients taking other transport	# Patients in Definitive Care on other Transport	# Patients in Definitive Care	# Patients Receiving Higher Quality Injury care	Patients Perception of Health System	Patients trust in healthcare system	Willingness to seek formal care	# Injured seeking care	# Patients taking an ambulance	# Patients in Definitive Care on Ambulance	# Patients in Definitive care on time			
694	17	Socioeconomic Determinant Loop	Total available workforce	GDP	Poverty	Individual wealth	Availability of other transport	Geographical accessibility	Availability of Ambulance Service	# Patients taking an ambulance	# Patients in Definitive Care on Ambulance	# Patients in Definitive Care	# Patients Receiving Higher Quality Injury care	Patients Perception of Health System	Patients trust in healthcare system	# Injured seeking non-formal care	Willingness to seek formal care	# Injured seeking care			
695	17	Socioeconomic Determinant Loop	Total available workforce	GDP	Poverty	Community Conflict Violence etc	Availability of quality injury rehab or follow-up	Discrimination	# Patients receiving Lower Quality Injury Care	Patients Perception of Health System	Patients trust in healthcare system	Willingness to seek formal care	# Injured seeking care	# Patients taking an ambulance	# Patients in Definitive Care on Ambulance	# Patients in Definitive Care	# Patients remaining in care/rehab	# Patients Receiving Higher Quality Rehab			
696	17	Socioeconomic Determinant Loop	Total available workforce	GDP	System level funding	Available Funding for injury care	Availability of quality injury rehab or follow-up	Discrimination	# Patients Receiving Higher Quality Injury care	Patients Perception of Health System	Patients trust in healthcare system	# Injured seeking non-formal care	Willingness to seek formal care	# Injured seeking care	# Patients taking an ambulance	# Patients in Definitive Care on Ambulance	# Patients in Definitive Care	# Patients receiving Lower Quality Injury Care			
697	17	Socioeconomic Determinant Loop	Total available workforce	GDP	System level funding	Available Funding for injury care	Availability of Ambulance Service	Geographical accessibility	Availability of other transport	# Patients taking other transport	# Patients in Definitive Care on other Transport	# Patients in Definitive Care	# Patients receiving Lower Quality Injury Care	Patients Perception of Health System	Patients trust in healthcare system	Willingness to remain in care	# Patients remaining in care/rehab	# Patients Receiving Higher Quality Rehab			
698	17	Socioeconomic Determinant Loop	Total available workforce	GDP	System level funding	Available Funding for injury care	Availability of quality injury care	# Patients receiving Lower Quality Injury Care	Patients Perception of Health System	Patients trust in healthcare system	# Injured seeking non-formal care	Willingness to seek formal care	# Injured seeking care	# Patients taking an ambulance	# Patients in Definitive Care on Ambulance	# Patients in Definitive Care	# Patients remaining in care/rehab	# Patients Receiving Higher Quality Rehab			
699	17	Socioeconomic Determinant Loop	Total available workforce	GDP	Poverty	Community Conflict Violence etc	Availability of quality injury care	# Patients Receiving Higher Quality Injury care	Patients Perception of Health System	Patients trust in healthcare system	# Injured seeking non-formal care	Willingness to seek formal care	# Injured seeking care	# Patients taking other transport	# Patients in Definitive Care on other Transport	# Patients in Definitive Care	# Patients remaining in care/rehab	# Patients Receiving Higher Quality Rehab			
700	17	Socioeconomic Determinant Loop	Total available workforce	GDP	System level funding	Available Funding for injury care	Availability of Ambulance Service	Geographical accessibility	Availability of other transport	# Patients taking other transport	# Patients in Definitive Care on other Transport	# Patients in Definitive Care	# Patients receiving Lower Quality Injury Care	Patients Perception of Health System	Patients trust in healthcare system	Willingness to seek formal care	# Injured seeking care	# Patients taking an ambulance			
701	17	Socioeconomic Determinant Loop	Total available workforce	GDP	System level funding	Available Funding for injury care	Availability of quality injury care	Discrimination	# Patients receiving Lower Quality Injury Care	Patients Perception of Health System	Patients trust in healthcare system	# Injured seeking non-formal care	Willingness to seek formal care	# Injured seeking care	# Patients taking an ambulance	# Patients in Definitive Care on Ambulance	# Patients in Definitive Care	# Patients Receiving Higher Quality Injury care			
702	17	Socioeconomic Determinant Loop	Total available workforce	GDP	System level funding	Available Funding for injury care	Universal health insurance	Ability to pay	Willingness to receive care	# Patients in Definitive Care	# Patients receiving Lower Quality Injury Care	Patients Perception of Health System	Patients trust in healthcare system	Willingness to seek formal care	# Injured seeking care	# Patients taking an ambulance	# Patients in Definitive Care on Ambulance	# Patients in Definitive care Delayed			

703	17	Socioeconomic Determinant Loop	Total available workforce	GDP	Poverty	Individual wealth	Availability of other transport	Geographical accessibility	Availability of Ambulance Service	# Patients taking an ambulance	# Patients in Definitive Care on Ambulance	# Patients in Definitive Care	# Patients receiving Lower Quality Injury Care	Patients Perception of Health System	Patients trust in healthcare system	Willingness to remain in care	# Patients remaining in care/rehab	# Patients receiving Lower Quality Rehab				
704	17	Socioeconomic Determinant Loop	Total available workforce	GDP	System level funding	Available Funding for injury care	Availability of Ambulance Service	# Patients taking an ambulance	# Patients in Definitive Care on Ambulance	# Patients in Definitive Care	# Patients receiving Lower Quality Injury Care	Patients Perception of Health System	Patients trust in healthcare system	Willingness to seek formal care	# Injured seeking care	# Patients taking other transport	# Patients in Definitive Care on other Transport	# Patients in Definitive care on time				
705	17	Socioeconomic Determinant Loop	Total available workforce	GDP	System level funding	Available Funding for injury care	Availability of quality injury care	Discrimination	# Patients Receiving Higher Quality Injury care	Patients Perception of Health System	Patients trust in healthcare system	Willingness to seek formal care	# Injured seeking care	# Patients taking an ambulance	# Patients in Definitive Care on Ambulance	# Patients in Definitive Care	# Patients remaining in care/rehab	# Patients receiving Lower Quality Rehab				
706	17	Socioeconomic Determinant Loop	Total available workforce	GDP	System level funding	Available Funding for injury care	Availability of quality injury care	Discrimination	# Patients receiving Lower Quality Injury Care	Patients Perception of Health System	Patients trust in healthcare system	Willingness to seek formal care	# Injured seeking care	# Patients taking an ambulance	# Patients in Definitive Care on Ambulance	# Patients in Definitive Care	# Patients remaining in care/rehab	# Patients Receiving Higher Quality Rehab				
707	17	Socioeconomic Determinant Loop	Total available workforce	GDP	System level funding	Available Funding for injury care	Availability of Ambulance Service	# Patients taking an ambulance	# Patients in Definitive Care on Ambulance	# Patients in Definitive Care	# Patients receiving Lower Quality Injury Care	Patients Perception of Health System	Patients trust in healthcare system	Willingness to seek formal care	# Injured seeking care	# Patients taking other transport	# Patients in Definitive Care on other Transport	# Patients in Definitive care Delayed				
708	17	Socioeconomic Determinant Loop	Total available workforce	GDP	Poverty	Individual wealth	Availability of other transport	Geographical accessibility	Availability of Ambulance Service	# Patients taking an ambulance	# Patients in Definitive Care on Ambulance	# Patients in Definitive Care	# Patients Receiving Higher Quality Injury care	Patients Perception of Health System	Patients trust in healthcare system	Willingness to remain in care	# Patients remaining in care/rehab	# Patients receiving Lower Quality Rehab				
709	17	Socioeconomic Determinant Loop	Total available workforce	GDP	System level funding	Available Funding for injury care	Availability of Ambulance Service	Geographical accessibility	Availability of other transport	# Patients taking other transport	# Patients in Definitive Care on other Transport	# Patients in Definitive Care	# Patients Receiving Higher Quality Injury care	Patients Perception of Health System	Patients trust in healthcare system	Willingness to remain in care	# Patients remaining in care/rehab	# Patients Receiving Higher Quality Rehab				
710	17	Socioeconomic Determinant Loop	Total available workforce	GDP	Poverty	Community Conflict Violence etc	Availability of quality injury care	Discrimination	# Patients receiving Lower Quality Injury Care	Patients Perception of Health System	Patients trust in healthcare system	# Injured seeking non-formal care	Willingness to seek formal care	# Injured seeking care	# Patients taking an ambulance	# Patients in Definitive Care on Ambulance	# Patients in Definitive Care	# Patients Receiving Higher Quality Injury care				
711	17	Socioeconomic Determinant Loop	Total available workforce	GDP	System level funding	Available Funding for injury care	Availability of quality injury care	# Patients Receiving Higher Quality Injury care	Patients Perception of Health System	Patients trust in healthcare system	# Injured seeking non-formal care	Willingness to seek formal care	# Injured seeking care	# Patients taking an ambulance	# Patients in Definitive Care on Ambulance	# Patients in Definitive Care	# Patients remaining in care/rehab	# Patients Receiving Higher Quality Rehab				
712	17	Socioeconomic Determinant Loop	Total available workforce	GDP	Poverty	Community Conflict Violence etc	Availability of quality injury care	# Patients Receiving Higher Quality Injury care	Patients Perception of Health System	Patients trust in healthcare system	# Injured seeking non-formal care	Willingness to seek formal care	# Injured seeking care	# Patients taking other transport	# Patients in Definitive Care on other Transport	# Patients in Definitive Care	# Patients remaining in care/rehab	# Patients receiving Lower Quality Rehab				
713	17	Socioeconomic Determinant Loop	Total available workforce	GDP	Poverty	Community Conflict Violence etc	Availability of quality injury care	# Patients receiving Lower Quality Injury Care	Patients Perception of Health System	Patients trust in healthcare system	# Injured seeking non-formal care	Willingness to seek formal care	# Injured seeking care	# Patients taking other transport	# Patients in Definitive Care on other Transport	# Patients in Definitive Care	# Patients remaining in care/rehab	# Patients receiving Lower Quality Rehab				
714	17	Socioeconomic Determinant Loop	Total available workforce	GDP	System level funding	Available Funding for injury care	Availability of quality injury care	Discrimination	# Patients receiving Lower Quality Injury Care	Patients Perception of Health System	Patients trust in healthcare system	Willingness to seek formal care	# Injured seeking care	# Patients taking other transport	# Patients in Definitive Care on other Transport	# Patients in Definitive Care	# Patients remaining in care/rehab	# Patients Receiving Higher Quality Rehab				
715	17	Socioeconomic Determinant Loop	Total available workforce	GDP	Poverty	Community Conflict Violence etc	Availability of quality injury rehab or follow-up	Discrimination	# Patients Receiving Higher Quality Injury care	Patients Perception of Health System	Patients trust in healthcare system	# Injured seeking non-formal care	Willingness to seek formal care	# Injured seeking care	# Patients taking other transport	# Patients in Definitive Care on other Transport	# Patients in Definitive Care	# Patients receiving Lower Quality Injury Care				

716	17	Socioeconomic Determinant Loop	Total available workforce	GDP	Poverty	Community Conflict Violence etc	Availability of quality injury rehab or follow-up	Discrimination	# Patients receiving Lower Quality Injury Care	Patients Perception of Health System	Patients trust in healthcare system	# Injured seeking non-formal care	Willingness to seek formal care	# Injured seeking care	# Patients taking other transport	# Patients in Definitive Care on other Transport	# Patients in Definitive Care	# Patients Receiving Higher Quality Injury care			
717	17	Socioeconomic Determinant Loop	Total available workforce	GDP	Poverty	Community Conflict Violence etc	Availability of quality injury rehab or follow-up	Discrimination	# Patients Receiving Higher Quality Injury care	Patients Perception of Health System	Patients trust in healthcare system	Willingness to seek formal care	# Injured seeking care	# Patients taking other transport	# Patients in Definitive Care on other Transport	# Patients in Definitive Care	# Patients remaining in care/rehab	# Patients Receiving Higher Quality Rehab			
718	17	Socioeconomic Determinant Loop	Total available workforce	GDP	System level funding	Available Funding for injury care	Availability of quality injury care	# Patients receiving Lower Quality Injury Care	Patients Perception of Health System	Patients trust in healthcare system	# Injured seeking non-formal care	Willingness to seek formal care	# Injured seeking care	# Patients taking other transport	# Patients in Definitive Care on other Transport	# Patients in Definitive Care	# Patients remaining in care/rehab	# Patients receiving Lower Quality Rehab			
719	17	Socioeconomic Determinant Loop	Total available workforce	GDP	System level funding	Available Funding for injury care	Availability of quality injury care	Discrimination	# Patients receiving Lower Quality Injury Care	Patients Perception of Health System	Patients trust in healthcare system	Willingness to seek formal care	# Injured seeking care	# Patients taking an ambulance	# Patients in Definitive Care on Ambulance	# Patients in Definitive Care	# Patients remaining in care/rehab	# Patients receiving Lower Quality Rehab			
720	17	Socioeconomic Determinant Loop	Total available workforce	GDP	Poverty	Community Conflict Violence etc	Availability of quality injury rehab or follow-up	Discrimination	# Patients Receiving Higher Quality Injury care	Patients Perception of Health System	Patients trust in healthcare system	# Injured seeking non-formal care	Willingness to seek formal care	# Injured seeking care	# Patients taking an ambulance	# Patients in Definitive Care on Ambulance	# Patients in Definitive Care	# Patients receiving Lower Quality Injury Care			
721	17	Socioeconomic Determinant Loop	Total available workforce	GDP	System level funding	Available Funding for injury care	Universal health insurance	Ability to pay	Willingness to receive care	# Patients in Definitive Care	# Patients receiving Lower Quality Injury Care	Patients Perception of Health System	Patients trust in healthcare system	Willingness to seek formal care	Injured seeking care	# Patients taking other transport	# Patients in Definitive Care on other Transport	# Patients in Definitive care Delayed			
722	17	Socioeconomic Determinant Loop	Total available workforce	GDP	System level funding	Available Funding for injury care	Availability of Ambulance Service	Geographical accessibility	Availability of other transport	# Patients taking other transport	# Patients in Definitive Care on other Transport	# Patients in Definitive Care	# Patients receiving Lower Quality Injury Care	Patients Perception of Health System	Patients trust in healthcare system	Willingness to remain in care	# Patients remaining in care/rehab	# Patients receiving Lower Quality Rehab			
723	17	Socioeconomic Determinant Loop	Total available workforce	GDP	Poverty	Community Conflict Violence etc	Availability of quality injury rehab or follow-up	Discrimination	# Patients Receiving Higher Quality Injury care	Patients Perception of Health System	Patients trust in healthcare system	Willingness to seek formal care	# Injured seeking care	# Patients taking an ambulance	# Patients in Definitive Care on Ambulance	# Patients in Definitive Care	# Patients remaining in care/rehab	# Patients Receiving Higher Quality Rehab			
724	17	Socioeconomic Determinant Loop	Total available workforce	GDP	Poverty	Individual wealth	Ability to pay	Willingness to receive care	# Patients in Definitive Care	# Patients receiving Lower Quality Injury Care	Patients Perception of Health System	Patients trust in healthcare system	# Injured seeking non-formal care	Willingness to seek formal care	# Injured seeking care	# Patients taking an ambulance	# Patients in Definitive Care on Ambulance	# Patients in Definitive care on time			
725	17	Socioeconomic Determinant Loop	Total available workforce	GDP	System level funding	Available Funding for injury care	Availability of quality injury care	# Patients Receiving Higher Quality Injury care	Patients Perception of Health System	Patients trust in healthcare system	# Injured seeking non-formal care	Willingness to seek formal care	# Injured seeking care	# Patients taking an ambulance	# Patients in Definitive Care on Ambulance	# Patients in Definitive Care	# Patients remaining in care/rehab	# Patients receiving Lower Quality Rehab			
726	17	Socioeconomic Determinant Loop	Total available workforce	GDP	System level funding	Available Funding for injury care	Availability of quality injury care	Discrimination	# Patients receiving Lower Quality Injury Care	Patients Perception of Health System	Patients trust in healthcare system	Willingness to seek formal care	# Injured seeking care	# Patients taking other transport	# Patients in Definitive Care on other Transport	# Patients in Definitive Care	# Patients remaining in care/rehab	# Patients receiving Lower Quality Rehab			
727	17	Socioeconomic Determinant Loop	Total available workforce	GDP	System level funding	Available Funding for injury care	Availability of quality injury care	Discrimination	# Patients Receiving Higher Quality Injury care	Patients Perception of Health System	Patients trust in healthcare system	Willingness to seek formal care	# Injured seeking care	# Patients taking other transport	# Patients in Definitive Care on other Transport	# Patients in Definitive Care	# Patients remaining in care/rehab	# Patients receiving Lower Quality Rehab			
728	17	Socioeconomic Determinant Loop	Total available workforce	GDP	Poverty	Individual wealth	Ability to pay	Willingness to seek formal care	# Injured seeking care	# Patients taking an ambulance	# Patients in Definitive Care on Ambulance	# Patients in Definitive Care	# Patients receiving Lower Quality Injury Care	Patients Perception of Health System	Patients trust in healthcare system	Willingness to remain in care	# Patients remaining in care/rehab	# Patients Receiving Higher Quality Rehab			

729	17	Socioeconomic Determinant Loop	Total available workforce	GDP	Poverty	Individual wealth	Ability to pay	Willingness to seek formal care	# Injured seeking care	# Patients taking an ambulance	# Patients in Definitive Care on Ambulance	# Patients in Definitive Care	# Patients receiving Lower Quality Injury Care	Patients Perception of Health System	Patients trust in healthcare system	Willingness to remain in care	# Patients remaining in care/rehab	# Patients receiving Lower Quality Rehab			
730	17	Socioeconomic Determinant Loop	Total available workforce	GDP	System level funding	Available Funding for injury care	Availability of quality injury care	# Patients Receiving Higher Quality Injury care	Patients Perception of Health System	Patients trust in healthcare system	# Injured seeking non-formal care	Willingness to seek formal care	# Injured seeking care	# Patients taking other transport	# Patients in Definitive Care on other Transport	# Patients in Definitive Care	# Patients remaining in care/rehab	# Patients Receiving Higher Quality Rehab			
731	17	Socioeconomic Determinant Loop	Total available workforce	GDP	Poverty	Community Conflict Violence etc	Availability of quality injury rehab or follow-up	Discrimination	# Patients receiving Lower Quality Injury Care	Patients Perception of Health System	Patients trust in healthcare system	# Injured seeking non-formal care	Willingness to seek formal care	# Injured seeking care	# Patients taking an ambulance	# Patients in Definitive Care on Ambulance	# Patients in Definitive Care	# Patients Receiving Higher Quality Injury care			
732	17	Socioeconomic Determinant Loop	Total available workforce	GDP	Poverty	Individual wealth	Ability to pay	Willingness to seek formal care	# Injured seeking care	# Patients taking an ambulance	# Patients in Definitive Care on Ambulance	# Patients in Definitive Care	# Patients Receiving Higher Quality Injury care	Patients Perception of Health System	Patients trust in healthcare system	Willingness to remain in care	# Patients remaining in care/rehab	# Patients Receiving Higher Quality Rehab			
733	17	Socioeconomic Determinant Loop	Total available workforce	GDP	Poverty	Community Conflict Violence etc	Availability of quality injury rehab or follow-up	Discrimination	# Patients Receiving Higher Quality Injury care	Patients Perception of Health System	Patients trust in healthcare system	Willingness to seek formal care	# Injured seeking care	# Patients taking an ambulance	# Patients in Definitive Care on Ambulance	# Patients in Definitive Care	# Patients remaining in care/rehab	# Patients receiving Lower Quality Rehab			
734	17	Socioeconomic Determinant Loop	Total available workforce	GDP	System level funding	Available Funding for injury care	Universal health insurance	Ability to pay	Willingness to receive care	# Patients in Definitive Care	# Patients receiving Lower Quality Injury Care	Patients Perception of Health System	Patients trust in healthcare system	Willingness to seek formal care	# Injured seeking care	# Patients taking other transport	# Patients in Definitive Care on other Transport	# Patients in Definitive care on time			
735	17	Socioeconomic Determinant Loop	Total available workforce	GDP	Poverty	Individual wealth	Ability to pay	Willingness to receive care	# Patients in Definitive Care	# Patients Receiving Higher Quality Injury care	Patients Perception of Health System	Patients trust in healthcare system	# Injured seeking non-formal care	Willingness to seek formal care	# Injured seeking care	# Patients taking other transport	# Patients in Definitive Care on other Transport	# Patients in Definitive care Delayed			
736	17	Socioeconomic Determinant Loop	Total available workforce	GDP	System level funding	Available Funding for injury care	Availability of quality injury rehab or follow-up	Discrimination	# Patients receiving Lower Quality Injury Care	Patients Perception of Health System	Patients trust in healthcare system	# Injured seeking non-formal care	Willingness to seek formal care	# Injured seeking care	# Patients taking an ambulance	# Patients in Definitive Care on Ambulance	# Patients in Definitive Care	# Patients Receiving Higher Quality Injury care			
737	17	Socioeconomic Determinant Loop	Total available workforce	GDP	System level funding	Available Funding for injury care	Availability of Ambulance Service	Geographical accessibility	Availability of other transport	# Patients taking other transport	# Patients in Definitive Care on other Transport	# Patients in Definitive Care	# Patients Receiving Higher Quality Injury care	Patients Perception of Health System	Patients trust in healthcare system	Willingness to remain in care	# Patients remaining in care/rehab	# Patients receiving Lower Quality Rehab			
738	17	Socioeconomic Determinant Loop	Total available workforce	GDP	System level funding	Available Funding for injury care	Availability of quality injury care	# Patients receiving Lower Quality Injury Care	Patients Perception of Health System	Patients trust in healthcare system	# Injured seeking non-formal care	Willingness to seek formal care	# Injured seeking care	# Patients taking an ambulance	# Patients in Definitive Care on Ambulance	# Patients in Definitive Care	# Patients remaining in care/rehab	# Patients receiving Lower Quality Rehab			
739	17	Socioeconomic Determinant Loop	Total available workforce	GDP	System level funding	Available Funding for injury care	Availability of quality injury care	# Patients receiving Lower Quality Injury Care	Patients Perception of Health System	Patients trust in healthcare system	# Injured seeking non-formal care	Willingness to seek formal care	# Injured seeking care	# Patients taking other transport	# Patients in Definitive Care on other Transport	# Patients in Definitive Care	# Patients remaining in care/rehab	# Patients Receiving Higher Quality Rehab			
740	17	Socioeconomic Determinant Loop	Total available workforce	GDP	System level funding	Available Funding for injury care	Availability of Ambulance Service	Geographical accessibility	Availability of other transport	# Patients taking other transport	# Patients in Definitive Care on other Transport	# Patients in Definitive Care	# Patients receiving Lower Quality Injury Care	Patients Perception of Health System	Patients trust in healthcare system	# Injured seeking non-formal care	Willingness to seek formal care	# Injured seeking care			
741	17	Socioeconomic Determinant Loop	Total available workforce	GDP	Poverty	Individual wealth	Ability to pay	Willingness to receive care	# Patients in Definitive Care	# Patients receiving Lower Quality Injury Care	Patients Perception of Health System	Patients trust in healthcare system	# Injured seeking non-formal care	Willingness to seek formal care	# Injured seeking care	# Patients taking an ambulance	# Patients in Definitive Care on Ambulance	# Patients in Definitive care Delayed			
742	17	Socioeconomic Determinant Loop	Total available workforce	GDP	Poverty	Community Conflict	Availability	Discrimination	# Patients Receiving	Patients Perceptio	Patients trust in	# Injured seeking	Willingness to seek	# Injured	# Patients taking an	# Patients in Definitive	# Patients in	# Patients receiving			

						Violence etc	of quality injury care		Higher Quality Injury care	n of Health System	healthcare system	non-formal care	formal care	seeking care	ambulance	Care on Ambulance	Definitive Care	Lower Quality Injury Care			
743	18	Full-System Multicausal Loop	Total available workforce	GDP	Poverty	Community Conflict Violence etc	Availability of quality injury care	# Patients Receiving Higher Quality Injury care	Patients Perception of Health System	Patients trust in healthcare system	Willingness to seek formal care	# Injured seeking care	# Patients taking an ambulance	# Patients in Definitive Care on Ambulance	# Patients in Definitive Care	# Patients receiving Lower Quality Injury Care	Willingness to remain in care	# Patients remaining in care/rehab	# Patients Receiving Higher Quality Rehab		
744	18	Full-System Multicausal Loop	Total available workforce	GDP	Poverty	Community Conflict Violence etc	Availability of quality injury rehab or follow-up	Discrimination	# Patients receiving Lower Quality Injury Care	Patients Perception of Health System	Patients trust in healthcare system	# Injured seeking non-formal care	Willingness to seek formal care	# Injured seeking care	# Patients taking other transport	# Patients in Definitive Care on other Transport	# Patients in Definitive Care	# Patients remaining in care/rehab	# Patients Receiving Higher Quality Rehab		
745	18	Full-System Multicausal Loop	Total available workforce	GDP	Poverty	Community Conflict Violence etc	Availability of quality injury care	# Patients receiving Lower Quality Injury Care	Patients Perception of Health System	Patients trust in healthcare system	Willingness to seek formal care	# Injured seeking care	# Patients taking other transport	# Patients in Definitive Care on other Transport	# Patients in Definitive Care	# Patients Receiving Higher Quality Injury care	Willingness to remain in care	# Patients remaining in care/rehab	# Patients Receiving Higher Quality Rehab		
746	18	Full-System Multicausal Loop	Total available workforce	GDP	System level funding	Available Funding for injury care	Availability of quality injury rehab or follow-up	Discrimination	# Patients Receiving Higher Quality Injury care	Patients Perception of Health System	Patients trust in healthcare system	# Injured seeking non-formal care	Willingness to seek formal care	# Injured seeking care	# Patients taking an ambulance	# Patients in Definitive Care on Ambulance	# Patients in Definitive Care	# Patients remaining in care/rehab	# Patients Receiving Higher Quality Rehab		
747	18	Full-System Multicausal Loop	Total available workforce	GDP	Poverty	Community Conflict Violence etc	Availability of quality injury rehab or follow-up	Discrimination	# Patients Receiving Higher Quality Injury care	Patients Perception of Health System	Patients trust in healthcare system	# Injured seeking non-formal care	Willingness to seek formal care	# Injured seeking care	# Patients taking other transport	# Patients in Definitive Care on other Transport	# Patients in Definitive Care	# Patients remaining in care/rehab	# Patients Receiving Higher Quality Rehab		
748	18	Full-System Multicausal Loop	Total available workforce	GDP	System level funding	Available Funding for injury care	Universal health insurance	Ability to pay	Willingness to receive care	# Patients in Definitive Care	# Patients Receiving Higher Quality Injury care	Patients Perception of Health System	Patients trust in healthcare system	# Injured seeking non-formal care	Willingness to seek formal care	# Injured seeking care	# Patients taking an ambulance	# Patients in Definitive Care on Ambulance	# Patients in Definitive care on time		
749	18	Full-System Multicausal Loop	Total available workforce	GDP	Poverty	Community Conflict Violence etc	Availability of quality injury care	# Patients receiving Lower Quality Injury Care	Patients Perception of Health System	Patients trust in healthcare system	Willingness to seek formal care	# Injured seeking care	# Patients taking an ambulance	# Patients in Definitive Care on Ambulance	# Patients in Definitive Care	# Patients Receiving Higher Quality Injury care	Willingness to remain in care	# Patients remaining in care/rehab	# Patients Receiving Higher Quality Rehab		
750	18	Full-System Multicausal Loop	Total available workforce	GDP	System level funding	Available Funding for injury care	Availability of Ambulance Service	# Patients taking an ambulance	# Patients in Definitive Care on Ambulance	# Patients in Definitive Care	# Patients receiving Lower Quality Injury Care	Patients Perception of Health System	Patients trust in healthcare system	# Injured seeking non-formal care	Willingness to seek formal care	# Injured seeking care	# Patients taking other transport	# Patients in Definitive Care on other Transport	# Patients in Definitive care Delayed		
751	18	Full-System Multicausal Loop	Total available workforce	GDP	Poverty	Community Conflict Violence etc	Availability of quality injury rehab or follow-up	Discrimination	# Patients Receiving Higher Quality Injury care	Patients Perception of Health System	Patients trust in healthcare system	# Injured seeking non-formal care	Willingness to seek formal care	# Injured seeking care	# Patients taking an ambulance	# Patients in Definitive Care on Ambulance	# Patients in Definitive Care	# Patients remaining in care/rehab	# Patients receiving Lower Quality Rehab		
752	18	Full-System Multicausal Loop	Total available workforce	GDP	System level funding	Available Funding for injury care	Availability of quality injury care	# Patients receiving Lower Quality Injury Care	Patients Perception of Health System	Patients trust in healthcare system	Willingness to seek formal care	# Injured seeking care	# Patients taking an ambulance	# Patients in Definitive Care on Ambulance	# Patients in Definitive Care	# Patients Receiving Higher Quality Injury care	Willingness to remain in care	# Patients remaining in care/rehab	# Patients Receiving Higher Quality Rehab		
753	18	Full-System Multicausal Loop	Total available workforce	GDP	System level funding	Available Funding for injury care	Universal health insurance	Ability to pay	Willingness to seek formal care	# Injured seeking care	# Patients taking an ambulance	# Patients in Definitive Care on Ambulance	# Patients in Definitive Care	# Patients receiving Lower Quality Injury Care	Patients Perception of Health System	Patients trust in healthcare system	Willingness to remain in care	# Patients remaining in care/rehab	# Patients Receiving Higher Quality Rehab		
754	18	Full-System Multicausal Loop	Total available workforce	GDP	Poverty	Community Conflict Violence etc	Availability of quality injury rehab or follow-up	Discrimination	# Patients receiving Lower Quality Injury Care	Patients Perception of Health System	Patients trust in healthcare system	# Injured seeking non-formal care	Willingness to seek formal care	# Injured seeking care	# Patients taking other transport	# Patients in Definitive Care on other Transport	# Patients in Definitive Care	# Patients remaining in care/rehab	# Patients receiving Lower Quality Rehab		
755	18	Full-System Multicausal Loop	Total available workforce	GDP	Poverty	Community Conflict Violence etc	Availability of quality injury care	# Patients Receiving Higher Quality Injury care	Patients Perception of Health System	Patients trust in healthcare system	Willingness to seek formal care	# Injured seeking care	# Patients taking other transport	# Patients in Definitive Care on other Transport	# Patients in Definitive Care	# Patients receiving Lower Quality	Willingness to remain in care	# Patients remaining in care/rehab	# Patients Receiving Higher Quality Rehab		

																Injury Care					
756	18	Full-System Multicausal Loop	Total available workforce	GDP	System level funding	Available Funding for injury care	Universal health insurance	Ability to pay	Willingness to seek formal care	# Injured seeking care	# Patients taking other transport	# Patients in Definitive Care on other Transport	# Patients in Definitive Care	# Patients Receiving Higher Quality Injury care	Patients Perception of Health System	Patients trust in healthcare system	Willingness to remain in care	# Patients remaining in care/rehab	# Patients receiving Lower Quality Rehab		
757	18	Full-System Multicausal Loop	Total available workforce	GDP	System level funding	Available Funding for injury care	Availability of quality injury care	# Patients receiving Lower Quality Injury Care	Patients Perception of Health System	Patients trust in healthcare system	Willingness to seek formal care	# Injured seeking care	# Patients taking an ambulance	# Patients in Definitive Care on Ambulance	# Patients in Definitive Care	# Patients Receiving Higher Quality Injury care	Willingness to remain in care	# Patients remaining in care/rehab	# Patients receiving Lower Quality Rehab		
758	18	Full-System Multicausal Loop	Total available workforce	GDP	Poverty	Community Conflict Violence etc	Geographical accessibility	Availability of Ambulance Service	# Patients taking an ambulance	# Patients in Definitive Care on Ambulance	# Patients in Definitive Care	# Patients receiving Lower Quality Injury Care	Patients Perception of Health System	Patients trust in healthcare system	Willingness to seek formal care	# Injured seeking care	# Patients taking other transport	# Patients in Definitive Care on other Transport	# Patients in Definitive care Delayed		
759	18	Full-System Multicausal Loop	Total available workforce	GDP	System level funding	Available Funding for injury care	Availability of quality injury care	# Patients Receiving Higher Quality Injury care	Patients Perception of Health System	Patients trust in healthcare system	Willingness to seek formal care	# Injured seeking care	# Patients taking an ambulance	# Patients in Definitive Care on Ambulance	# Patients in Definitive Care	# Patients receiving Lower Quality Injury Care	Willingness to remain in care	# Patients remaining in care/rehab	# Patients Receiving Higher Quality Rehab		
760	18	Full-System Multicausal Loop	Total available workforce	GDP	Poverty	Community Conflict Violence etc	Availability of quality injury rehab or follow-up	Discrimination	# Patients receiving Lower Quality Injury Care	Patients Perception of Health System	Patients trust in healthcare system	# Injured seeking non-formal care	Willingness to seek formal care	# Injured seeking care	# Patients taking an ambulance	# Patients in Definitive Care on Ambulance	# Patients in Definitive Care	# Patients remaining in care/rehab	# Patients Receiving Higher Quality Rehab		
761	18	Full-System Multicausal Loop	Total available workforce	GDP	System level funding	Available Funding for injury care	Availability of quality injury care	Discrimination	# Patients receiving Lower Quality Injury Care	Patients Perception of Health System	Patients trust in healthcare system	# Injured seeking non-formal care	Willingness to seek formal care	# Injured seeking care	# Patients taking other transport	# Patients in Definitive Care on other Transport	# Patients in Definitive Care	# Patients remaining in care/rehab	# Patients receiving Lower Quality Rehab		
762	18	Full-System Multicausal Loop	Total available workforce	GDP	Poverty	Community Conflict Violence etc	Availability of quality injury rehab or follow-up	Discrimination	# Patients receiving Lower Quality Injury Care	Patients Perception of Health System	Patients trust in healthcare system	# Injured seeking non-formal care	Willingness to seek formal care	# Injured seeking care	# Patients taking an ambulance	# Patients in Definitive Care on Ambulance	# Patients in Definitive Care	# Patients remaining in care/rehab	# Patients receiving Lower Quality Rehab		
763	18	Full-System Multicausal Loop	Total available workforce	GDP	System level funding	Available Funding for injury care	Universal health insurance	Ability to pay	Willingness to receive care	# Patients in Definitive Care	# Patients Receiving Higher Quality Injury care	Patients Perception of Health System	Patients trust in healthcare system	# Injured seeking non-formal care	Willingness to seek formal care	# Injured seeking care	# Patients taking other transport	# Patients in Definitive Care on other Transport	# Patients in Definitive care Delayed		
764	18	Full-System Multicausal Loop	Total available workforce	GDP	Poverty	Community Conflict Violence etc	Availability of quality injury care	Discrimination	# Patients Receiving Higher Quality Injury care	Patients Perception of Health System	Patients trust in healthcare system	# Injured seeking non-formal care	Willingness to seek formal care	# Injured seeking care	# Patients taking an ambulance	# Patients in Definitive Care on Ambulance	# Patients in Definitive Care	# Patients remaining in care/rehab	# Patients receiving Lower Quality Rehab		
765	18	Full-System Multicausal Loop	Total available workforce	GDP	System level funding	Available Funding for injury care	Availability of quality injury rehab or follow-up	Discrimination	# Patients Receiving Higher Quality Injury care	Patients Perception of Health System	Patients trust in healthcare system	# Injured seeking non-formal care	Willingness to seek formal care	# Injured seeking care	# Patients taking an ambulance	# Patients in Definitive Care on Ambulance	# Patients in Definitive Care	# Patients remaining in care/rehab	# Patients receiving Lower Quality Rehab		
766	18	Full-System Multicausal Loop	Total available workforce	GDP	Poverty	Community Conflict Violence etc	Availability of quality injury care	# Patients receiving Lower Quality Injury Care	Patients Perception of Health System	Patients trust in healthcare system	Willingness to seek formal care	# Injured seeking care	# Patients taking other transport	# Patients in Definitive Care on other Transport	# Patients in Definitive Care	# Patients Receiving Higher Quality Injury care	Willingness to remain in care	# Patients remaining in care/rehab	# Patients receiving Lower Quality Rehab		
767	18	Full-System Multicausal Loop	Total available workforce	GDP	Poverty	Community Conflict Violence etc	Availability of quality injury rehab or follow-up	Discrimination	# Patients Receiving Higher Quality Injury care	Patients Perception of Health System	Patients trust in healthcare system	# Injured seeking non-formal care	Willingness to seek formal care	# Injured seeking care	# Patients taking other transport	# Patients in Definitive Care on other Transport	# Patients in Definitive Care	# Patients remaining in care/rehab	# Patients receiving Lower Quality Rehab		
768	18	Full-System Multicausal Loop	Total available workforce	GDP	System level funding	Available Funding for injury care	Universal health insurance	Ability to pay	Willingness to receive care	# Patients in Definitive Care	# Patients Receiving Higher Quality Injury care	Patients Perception of Health System	Patients trust in healthcare system	# Injured seeking non-formal care	Willingness to seek formal care	# Injured seeking care	# Patients taking an ambulance	# Patients in Definitive Care on Ambulance	# Patients in Definitive care Delayed		

769	18	Full-System Multicausal Loop	Total available workforce	GDP	System level funding	Available Funding for injury care	Universal health insurance	Ability to pay	Willingness to seek formal care	# Injured seeking care	# Patients taking other transport	# Patients in Definitive Care on other Transport	# Patients in Definitive Care	# Patients receiving Lower Quality Injury Care	Patients Perception of Health System	Patients trust in healthcare system	Willingness to remain in care	# Patients remaining in care/rehab	# Patients receiving Lower Quality Rehab		
770	18	Full-System Multicausal Loop	Total available workforce	GDP	System level funding	Available Funding for injury care	Availability of quality injury care	# Patients Receiving Higher Quality Injury care	Patients Perception of Health System	Patients trust in healthcare system	Willingness to seek formal care	# Injured seeking care	# Patients taking an ambulance	# Patients in Definitive Care on Ambulance	# Patients in Definitive Care	# Patients receiving Lower Quality Injury Care	Willingness to remain in care	# Patients remaining in care/rehab	# Patients receiving Lower Quality Rehab		
771	18	Full-System Multicausal Loop	Total available workforce	GDP	System level funding	Available Funding for injury care	Availability of quality injury care	# Patients receiving Lower Quality Injury Care	Patients Perception of Health System	Patients trust in healthcare system	Willingness to seek formal care	# Injured seeking care	# Patients taking other transport	# Patients in Definitive Care on other Transport	# Patients in Definitive Care	# Patients Receiving Higher Quality Injury care	Willingness to remain in care	# Patients remaining in care/rehab	# Patients Receiving Higher Quality Rehab		
772	18	Full-System Multicausal Loop	Total available workforce	GDP	Poverty	Community Conflict Violence etc	Availability of quality injury care	# Patients receiving Lower Quality Injury Care	Patients Perception of Health System	Patients trust in healthcare system	Willingness to seek formal care	# Injured seeking care	# Patients taking an ambulance	# Patients in Definitive Care on Ambulance	# Patients in Definitive Care	# Patients Receiving Higher Quality Injury care	Willingness to remain in care	# Patients remaining in care/rehab	# Patients receiving Lower Quality Rehab		
773	18	Full-System Multicausal Loop	Total available workforce	GDP	Poverty	Community Conflict Violence etc	Availability of quality injury care	Discrimination	# Patients Receiving Higher Quality Injury care	Patients Perception of Health System	Patients trust in healthcare system	# Injured seeking non-formal care	Willingness to seek formal care	# Injured seeking care	# Patients taking other transport	# Patients in Definitive Care on other Transport	# Patients in Definitive Care	# Patients remaining in care/rehab	# Patients Receiving Higher Quality Rehab		
774	18	Full-System Multicausal Loop	Total available workforce	GDP	Poverty	Community Conflict Violence etc	Availability of quality injury care	# Patients Receiving Higher Quality Injury care	Patients Perception of Health System	Patients trust in healthcare system	Willingness to seek formal care	# Injured seeking care	# Patients taking an ambulance	# Patients in Definitive Care on Ambulance	# Patients in Definitive Care	# Patients receiving Lower Quality Injury Care	Willingness to remain in care	# Patients remaining in care/rehab	# Patients receiving Lower Quality Rehab		
775	18	Full-System Multicausal Loop	Total available workforce	GDP	Poverty	Community Conflict Violence etc	Availability of quality injury rehab or follow-up	Discrimination	# Patients Receiving Higher Quality Injury care	Patients Perception of Health System	Patients trust in healthcare system	# Injured seeking non-formal care	Willingness to seek formal care	# Injured seeking care	# Patients taking an ambulance	# Patients in Definitive Care on Ambulance	# Patients in Definitive Care	# Patients remaining in care/rehab	# Patients Receiving Higher Quality Rehab		
776	18	Full-System Multicausal Loop	Total available workforce	GDP	System level funding	Available Funding for injury care	Availability of Ambulance Service	Geographical accessibility	Availability of other transport	# Patients taking other transport	# Patients in Definitive Care on other Transport	# Patients in Definitive Care	# Patients Receiving Higher Quality Injury care	Patients Perception of Health System	Patients trust in healthcare system	# Injured seeking non-formal care	Willingness to seek formal care	# Injured seeking care	# Patients taking an ambulance		
777	18	Full-System Multicausal Loop	Total available workforce	GDP	System level funding	Available Funding for injury care	Availability of quality injury care	Discrimination	# Patients receiving Lower Quality Injury Care	Patients Perception of Health System	Patients trust in healthcare system	# Injured seeking non-formal care	Willingness to seek formal care	# Injured seeking care	# Patients taking other transport	# Patients in Definitive Care on other Transport	# Patients in Definitive Care	# Patients remaining in care/rehab	# Patients Receiving Higher Quality Rehab		
778	18	Full-System Multicausal Loop	Total available workforce	GDP	System level funding	Available Funding for injury care	Universal health insurance	Ability to pay	Willingness to receive care	# Patients in Definitive Care	# Patients Receiving Higher Quality Injury care	Patients Perception of Health System	Patients trust in healthcare system	# Injured seeking non-formal care	Willingness to seek formal care	# Injured seeking care	# Patients taking other transport	# Patients in Definitive Care on other Transport	# Patients in Definitive care on time		
779	18	Full-System Multicausal Loop	Total available workforce	GDP	System level funding	Available Funding for injury care	Universal health insurance	Ability to pay	Willingness to seek formal care	# Injured seeking care	# Patients taking an ambulance	# Patients in Definitive Care on Ambulance	# Patients in Definitive Care	# Patients receiving Lower Quality Injury Care	Patients Perception of Health System	Patients trust in healthcare system	Willingness to remain in care	# Patients remaining in care/rehab	# Patients receiving Lower Quality Rehab		
780	18	Full-System Multicausal Loop	Total available workforce	GDP	Poverty	Community Conflict Violence etc	Availability of quality injury care	Discrimination	# Patients Receiving Higher Quality Injury care	Patients Perception of Health System	Patients trust in healthcare system	# Injured seeking non-formal care	Willingness to seek formal care	# Injured seeking care	# Patients taking an ambulance	# Patients in Definitive Care on Ambulance	# Patients in Definitive Care	# Patients remaining in care/rehab	# Patients Receiving Higher Quality Rehab		
781	18	Full-System Multicausal Loop	Total available workforce	GDP	System level funding	Available Funding for injury care	Availability of quality injury rehab or follow-up	Discrimination	# Patients receiving Lower Quality Injury Care	Patients Perception of Health System	Patients trust in healthcare system	# Injured seeking non-formal care	Willingness to seek formal care	# Injured seeking care	# Patients taking an ambulance	# Patients in Definitive Care on Ambulance	# Patients in Definitive Care	# Patients remaining in care/rehab	# Patients Receiving Higher Quality Rehab		
782	18	Full-System Multicausal Loop	Total available workforce	GDP	Poverty	Individual wealth	Availability	# Patients	# Patients in Definitive	# Patients in	# Patients Receiving	Patients Perceptio	Patients trust in	# Injured seeking	Willingness to seek	# Injured	# Patients taking an	# Patients in Definitive	# Patients in Definitive		

							of other transport	taking other transport	Care on other Transport	Definitive Care	Higher Quality Injury care	n of Health System	healthcare system	non-formal care	formal care	seeking care	ambulance	Care on Ambulance	care on time		
783	18	Full-System Multicausal Loop	Total available workforce	GDP	System level funding	Available Funding for injury care	Availability of quality injury care	# Patients receiving Lower Quality Injury Care	Patients Perception of Health System	Patients trust in healthcare system	Willingness to seek formal care	# Injured seeking care	# Patients taking other transport	# Patients in Definitive Care on other Transport	# Patients in Definitive Care	# Patients Receiving Higher Quality Injury care	Willingness to remain in care	# Patients remaining in care/rehab	# Patients receiving Lower Quality Rehab		
784	18	Full-System Multicausal Loop	Total available workforce	GDP	Poverty	Community Conflict Violence etc	Geographical accessibility	Availability of Ambulance Service	# Patients taking an ambulance	# Patients in Definitive Care on Ambulance	# Patients in Definitive Care	# Patients Receiving Higher Quality Injury care	Patients Perception of Health System	Patients trust in healthcare system	Willingness to seek formal care	# Injured seeking care	# Patients taking other transport	# Patients in Definitive Care on other Transport	# Patients in Definitive care Delayed		
785	18	Full-System Multicausal Loop	Total available workforce	GDP	System level funding	Available Funding for injury care	Availability of quality injury rehab or follow-up	Discrimination	# Patients receiving Lower Quality Injury Care	Patients Perception of Health System	Patients trust in healthcare system	# Injured seeking non-formal care	Willingness to seek formal care	# Injured seeking care	# Patients taking other transport	# Patients in Definitive Care on other Transport	# Patients in Definitive Care	# Patients remaining in care/rehab	# Patients Receiving Higher Quality Rehab		
786	18	Full-System Multicausal Loop	Total available workforce	GDP	Poverty	Community Conflict Violence etc	Geographical accessibility	Availability of Ambulance Service	# Patients taking an ambulance	# Patients in Definitive Care on Ambulance	# Patients in Definitive Care	# Patients receiving Lower Quality Injury Care	Patients Perception of Health System	Patients trust in healthcare system	Willingness to seek formal care	# Injured seeking care	# Patients taking other transport	# Patients in Definitive Care on other Transport	# Patients in Definitive care on time		
787	18	Full-System Multicausal Loop	Total available workforce	GDP	System level funding	Available Funding for injury care	Availability of quality injury rehab or follow-up	Discrimination	# Patients Receiving Higher Quality Injury care	Patients Perception of Health System	Patients trust in healthcare system	# Injured seeking non-formal care	Willingness to seek formal care	# Injured seeking care	# Patients taking other transport	# Patients in Definitive Care on other Transport	# Patients in Definitive Care	# Patients remaining in care/rehab	# Patients receiving Lower Quality Rehab		
788	18	Full-System Multicausal Loop	Total available workforce	GDP	Poverty	Community Conflict Violence etc	Geographical accessibility	Availability of other transport	# Patients taking other transport	# Patients in Definitive Care on other Transport	# Patients in Definitive Care	# Patients Receiving Higher Quality Injury care	Patients Perception of Health System	Patients trust in healthcare system	Willingness to seek formal care	# Injured seeking care	# Patients taking an ambulance	# Patients in Definitive Care on Ambulance	# Patients in Definitive care Delayed		
789	18	Full-System Multicausal Loop	Total available workforce	GDP	Poverty	Community Conflict Violence etc	Availability of quality injury care	Discrimination	# Patients Receiving Higher Quality Injury care	Patients Perception of Health System	Patients trust in healthcare system	# Injured seeking non-formal care	Willingness to seek formal care	# Injured seeking care	# Patients taking other transport	# Patients in Definitive Care on other Transport	# Patients in Definitive Care	# Patients remaining in care/rehab	# Patients receiving Lower Quality Rehab		
790	18	Full-System Multicausal Loop	Total available workforce	GDP	System level funding	Available Funding for injury care	Universal health insurance	Ability to pay	Willingness to receive care	# Patients in Definitive Care	# Patients receiving Lower Quality Injury Care	Patients Perception of Health System	Patients trust in healthcare system	# Injured seeking non-formal care	Willingness to seek formal care	# Injured seeking care	# Patients taking other transport	# Patients in Definitive Care on other Transport	# Patients in Definitive care on time		
791	18	Full-System Multicausal Loop	Total available workforce	GDP	System level funding	Available Funding for injury care	Availability of Ambulance Service	Geographical accessibility	Availability of other transport	# Patients taking other transport	# Patients in Definitive Care on other Transport	# Patients in Definitive Care	# Patients receiving Lower Quality Injury Care	Patients Perception of Health System	Patients trust in healthcare system	# Injured seeking non-formal care	Willingness to seek formal care	# Injured seeking care	# Patients taking an ambulance		
792	18	Full-System Multicausal Loop	Total available workforce	GDP	System level funding	Available Funding for injury care	Universal health insurance	Ability to pay	Willingness to seek formal care	# Injured seeking care	# Patients taking other transport	# Patients in Definitive Care on other Transport	# Patients in Definitive Care	# Patients receiving Lower Quality Injury Care	Patients Perception of Health System	Patients trust in healthcare system	Willingness to remain in care	# Patients remaining in care/rehab	# Patients Receiving Higher Quality Rehab		
793	18	Full-System Multicausal Loop	Total available workforce	GDP	Poverty	Community Conflict Violence etc	Availability of quality injury care	Discrimination	# Patients receiving Lower Quality Injury Care	Patients Perception of Health System	Patients trust in healthcare system	# Injured seeking non-formal care	Willingness to seek formal care	# Injured seeking care	# Patients taking an ambulance	# Patients in Definitive Care on Ambulance	# Patients in Definitive Care	# Patients remaining in care/rehab	# Patients receiving Lower Quality Rehab		
794	18	Full-System Multicausal Loop	Total available workforce	GDP	Poverty	Community Conflict Violence etc	Availability of quality injury care	Discrimination	# Patients receiving Lower Quality Injury Care	Patients Perception of Health System	Patients trust in healthcare system	# Injured seeking non-formal care	Willingness to seek formal care	# Injured seeking care	# Patients taking an ambulance	# Patients in Definitive Care on Ambulance	# Patients in Definitive Care	# Patients remaining in care/rehab	# Patients Receiving Higher Quality Rehab		
795	18	Full-System Multicausal Loop	Total available workforce	GDP	System level funding	Available Funding for injury care	Availability of Ambulance Service	# Patients taking an ambulance	# Patients in Definitive Care on	# Patients in Definitive Care	# Patients receiving Lower Quality	Patients Perception of	Patients trust in healthcare system	# Injured seeking non-	Willingness to seek formal care	# Injured seeking care	# Patients taking other transport	# Patients in Definitive Care on	# Patients in Definitive care on time		

									Ambulanc e		Injury Care	Health System		formal care				other Transport			
796	18	Full-System Multicausal Loop	Total available workforce	GDP	Poverty	Communit y Conflict Violence etc	Availability of quality injury care	# Patients Receiving Higher Quality Injury care	Patients Perceptio n of Health System	Patients trust in healthcare system	Willingnes s to seek formal care	# Injured seeking care	# Patients taking other transport	# Patients in Definitive Care on other Transport	# Patients in Definitive Care	# Patients receiving Lower Quality Injury Care	Willingnes s to remain in care	# Patients remaining in care/reha b	# Patients receiving Lower Quality Rehab		
797	18	Full-System Multicausal Loop	Total available workforce	GDP	Poverty	Individual wealth	Availability of other transport	# Patients taking other transport	# Patients in Definitive Care on other Transport	# Patients in Definitive Care	# Patients receiving Lower Quality Injury Care	Patients Perceptio n of Health System	Patients trust in healthcare system	# Injured seeking non-formal care	Willingnes s to seek formal care	# Injured seeking care	# Patients taking an ambulanc e	# Patients in Definitive Care on Ambulanc e	# Patients in Definitive care on time		
798	18	Full-System Multicausal Loop	Total available workforce	GDP	System level funding	Available Funding for injury care	Universal health insurance	Ability to pay	Willingnes s to seek formal care	# Injured seeking care	# Patients taking an ambulanc e	# Patients in Definitive Care on Ambulanc e	# Patients in Definitive Care	# Patients Receiving Higher Quality Injury care	Patients Perceptio n of Health System	Patients trust in healthcare system	Willingnes s to remain in care	# Patients remaining in care/reha b	# Patients Receiving Higher Quality Rehab		
799	18	Full-System Multicausal Loop	Total available workforce	GDP	Poverty	Communit y Conflict Violence etc	Availability of quality injury care	Discriminatio n	# Patients receiving Lower Quality Injury Care	Patients Perceptio n of Health System	Patients trust in healthcare system	# Injured seeking non-formal care	Willingnes s to seek formal care	# Injured seeking care	# Patients taking other transport	# Patients in Definitive Care on other Transport	# Patients in Definitive Care	# Patients remaining in care/reha b	# Patients Receiving Higher Quality Rehab		
800	18	Full-System Multicausal Loop	Total available workforce	GDP	System level funding	Available Funding for injury care	Availability of quality injury rehab or follow-up	Discriminatio n	# Patients Receiving Higher Quality Injury care	Patients Perceptio n of Health System	Patients trust in healthcare system	# Injured seeking non-formal care	Willingnes s to seek formal care	# Injured seeking care	# Patients taking other transport	# Patients in Definitive Care on other Transport	# Patients in Definitive Care	# Patients remaining in care/reha b	# Patients Receiving Higher Quality Rehab		
801	18	Full-System Multicausal Loop	Total available workforce	GDP	Poverty	Communit y Conflict Violence etc	Geographic al accessibility	Availability of Ambulance Service	# Patients taking an ambulanc e	# Patients in Definitive Care on Ambulanc e	# Patients in Definitive Care	# Patients Receiving Higher Quality Injury care	Patients Perceptio n of Health System	Patients trust in healthcare system	Willingnes s to seek formal care	# Injured seeking care	# Patients taking other transport	# Patients in Definitive Care on other Transport	# Patients in Definitive care on time		
802	18	Full-System Multicausal Loop	Total available workforce	GDP	Poverty	Communit y Conflict Violence etc	Availability of quality injury care	Discriminatio n	# Patients receiving Lower Quality Injury Care	Patients Perceptio n of Health System	Patients trust in healthcare system	# Injured seeking non-formal care	Willingnes s to seek formal care	# Injured seeking care	# Patients taking other transport	# Patients in Definitive Care on other Transport	# Patients in Definitive Care	# Patients remaining in care/reha b	# Patients receiving Lower Quality Rehab		
803	18	Full-System Multicausal Loop	Total available workforce	GDP	System level funding	Available Funding for injury care	Availability of Ambulance Service	# Patients taking an ambulance	# Patients in Definitive Care on Ambulanc e	# Patients in Definitive Care	# Patients Receiving Higher Quality Injury care	Patients Perceptio n of Health System	Patients trust in healthcare system	# Injured seeking non-formal care	Willingnes s to seek formal care	# Injured seeking care	# Patients taking other transport	# Patients in Definitive Care on other Transport	# Patients in Definitive care on time		
804	18	Full-System Multicausal Loop	Total available workforce	GDP	System level funding	Available Funding for injury care	Universal health insurance	Ability to pay	Willingnes s to receive care	# Patients in Definitive Care	# Patients receiving Lower Quality Injury Care	Patients Perceptio n of Health System	Patients trust in healthcare system	# Injured seeking non-formal care	Willingnes s to seek formal care	# Injured seeking care	# Patients taking an ambulanc e	# Patients in Definitive Care on Ambulanc e	# Patients in Definitive care on time		
805	18	Full-System Multicausal Loop	Total available workforce	GDP	System level funding	Available Funding for injury care	Universal health insurance	Ability to pay	Willingnes s to receive care	# Patients in Definitive Care	# Patients receiving Lower Quality Injury Care	Patients Perceptio n of Health System	Patients trust in healthcare system	# Injured seeking non-formal care	Willingnes s to seek formal care	# Injured seeking care	# Patients taking other transport	# Patients in Definitive Care on other Transport	# Patients in Definitive care Delayed		
806	18	Full-System Multicausal Loop	Total available workforce	GDP	System level funding	Available Funding for injury care	Availability of quality injury rehab or follow-up	Discriminatio n	# Patients receiving Lower Quality Injury Care	Patients Perceptio n of Health System	Patients trust in healthcare system	# Injured seeking non-formal care	Willingnes s to seek formal care	# Injured seeking care	# Patients taking an ambulanc e	# Patients in Definitive Care on Ambulanc e	# Patients in Definitive Care	# Patients remaining in care/reha b	# Patients receiving Lower Quality Rehab		
807	18	Full-System Multicausal Loop	Total available workforce	GDP	System level funding	Available Funding for injury care	Availability of quality injury care	# Patients Receiving Higher Quality Injury care	Patients Perceptio n of Health System	Patients trust in healthcare system	Willingnes s to seek formal care	# Injured seeking care	# Patients taking other transport	# Patients in Definitive Care on other Transport	# Patients in Definitive Care	# Patients receiving Lower Quality Injury Care	Willingnes s to remain in care	# Patients remaining in care/reha b	# Patients receiving Lower Quality Rehab		
808	18	Full-System Multicausal Loop	Total available workforce	GDP	System level funding	Available Funding	Universal health insurance	Ability to pay	Willingnes s to	# Patients in Definitive Care	# Patients receiving Lower	Patients Perceptio n of	Patients trust in	# Injured seeking non-	Willingnes s to seek	# Injured seeking care	# Patients taking an	# Patients in Definitive Care on	# Patients in Definitive		

						for injury care			receive care		Quality Injury Care	Health System	healthcare system	formal care	formal care		ambulanc e	Ambulanc e	care Delayed		
809	18	Full-System Multicausal Loop	Total available workforce	GDP	System level funding	Available Funding for injury care	Availability of quality injury care	Discriminatio n	# Patients Receiving Higher Quality Injury care	Patients Perceptio n of Health System	Patients trust in healthcare system	# Injured seeking non-formal care	Willingnes s to seek formal care	# Injured seeking care	# Patients taking an ambulanc e	# Patients in Definitive Care on Ambulanc e	# Patients in Definitive Care	# Patients remaining in care/reha b	# Patients Receiving Higher Quality Rehab		
810	18	Full-System Multicausal Loop	Total available workforce	GDP	System level funding	Available Funding for injury care	Availability of quality injury care	Discriminatio n	# Patients Receiving Higher Quality Injury care	Patients Perceptio n of Health System	Patients trust in healthcare system	# Injured seeking non-formal care	Willingnes s to seek formal care	# Injured seeking care	# Patients taking an ambulanc e	# Patients in Definitive Care on Ambulanc e	# Patients in Definitive Care	# Patients remaining in care/reha b	# Patients receiving Lower Quality Rehab		
811	18	Full-System Multicausal Loop	Total available workforce	GDP	System level funding	Available Funding for injury care	Availability of quality injury care	# Patients Receiving Higher Quality Injury care	Patients Perceptio n of Health System	Patients trust in healthcare system	Willingnes s to seek formal care	# Injured seeking care	# Patients taking other transport	# Patients in Definitive Care on other Transport	# Patients in Definitive Care	# Patients receiving Lower Quality Injury Care	Willingnes s to remain in care	# Patients remaining in care/reha b	# Patients Receiving Higher Quality Rehab		
812	18	Full-System Multicausal Loop	Total available workforce	GDP	Poverty	Communit y Conflict Violence etc	Geographic al accessibility	Availability of other transport	# Patients taking other transport	# Patients in Definitive Care on other Transport	# Patients in Definitive Care	# Patients receiving Lower Quality Injury Care	Patients Perceptio n of Health System	Patients trust in healthcare system	Willingnes s to seek formal care	# Injured seeking care	# Patients taking an ambulanc e	# Patients in Definitive Care on Ambulanc e	# Patients in Definitive care on time		
813	18	Full-System Multicausal Loop	Total available workforce	GDP	System level funding	Available Funding for injury care	Availability of Ambulance Service	# Patients taking an ambulance	# Patients in Definitive Care on Ambulanc e	# Patients in Definitive Care	# Patients Receiving Higher Quality Injury care	Patients Perceptio n of Health System	Patients trust in healthcare system	# Injured seeking non-formal care	Willingnes s to seek formal care	# Injured seeking care	# Patients taking other transport	# Patients in Definitive Care on other Transport	# Patients in Definitive care Delayed		
814	18	Full-System Multicausal Loop	Total available workforce	GDP	System level funding	Available Funding for injury care	Availability of quality injury care	Discriminatio n	# Patients Receiving Higher Quality Injury care	Patients Perceptio n of Health System	Patients trust in healthcare system	# Injured seeking non-formal care	Willingnes s to seek formal care	# Injured seeking care	# Patients taking other transport	# Patients in Definitive Care on other Transport	# Patients in Definitive Care	# Patients remaining in care/reha b	# Patients receiving Lower Quality Rehab		
815	18	Full-System Multicausal Loop	Total available workforce	GDP	System level funding	Available Funding for injury care	Availability of quality injury care	Discriminatio n	# Patients Receiving Higher Quality Injury care	Patients Perceptio n of Health System	Patients trust in healthcare system	# Injured seeking non-formal care	Willingnes s to seek formal care	# Injured seeking care	# Patients taking other transport	# Patients in Definitive Care on other Transport	# Patients in Definitive Care	# Patients remaining in care/reha b	# Patients Receiving Higher Quality Rehab		
816	18	Full-System Multicausal Loop	Total available workforce	GDP	Poverty	Communit y Conflict Violence etc	Geographic al accessibility	Availability of other transport	# Patients taking other transport	# Patients in Definitive Care on other Transport	# Patients in Definitive Care	# Patients receiving Lower Quality Injury Care	Patients Perceptio n of Health System	Patients trust in healthcare system	Willingnes s to seek formal care	# Injured seeking care	# Patients taking an ambulanc e	# Patients in Definitive Care on Ambulanc e	# Patients in Definitive care Delayed		
817	18	Full-System Multicausal Loop	Total available workforce	GDP	Poverty	Communit y Conflict Violence etc	Geographic al accessibility	Availability of other transport	# Patients taking other transport	# Patients in Definitive Care on other Transport	# Patients in Definitive Care	# Patients Receiving Higher Quality Injury care	Patients Perceptio n of Health System	Patients trust in healthcare system	Willingnes s to seek formal care	# Injured seeking care	# Patients taking an ambulanc e	# Patients in Definitive Care on Ambulanc e	# Patients in Definitive care on time		
818	18	Full-System Multicausal Loop	Total available workforce	GDP	System level funding	Available Funding for injury care	Availability of quality injury care	Discriminatio n	# Patients receiving Lower Quality Injury Care	Patients Perceptio n of Health System	Patients trust in healthcare system	# Injured seeking non-formal care	Willingnes s to seek formal care	# Injured seeking care	# Patients taking an ambulanc e	# Patients in Definitive Care on Ambulanc e	# Patients in Definitive Care	# Patients remaining in care/reha b	# Patients Receiving Higher Quality Rehab		
819	18	Full-System Multicausal Loop	Total available workforce	GDP	Poverty	Individual wealth	Availability of other transport	# Patients taking other transport	# Patients in Definitive Care on other Transport	# Patients in Definitive Care	# Patients receiving Lower Quality Injury Care	Patients Perceptio n of Health System	Patients trust in healthcare system	# Injured seeking non-formal care	Willingnes s to seek formal care	# Injured seeking care	# Patients taking an ambulanc e	# Patients in Definitive Care on Ambulanc e	# Patients in Definitive care Delayed		
820	18	Full-System Multicausal Loop	Total available workforce	GDP	System level funding	Available Funding for injury care	Availability of quality injury rehab or follow-up	Discriminatio n	# Patients receiving Lower Quality Injury Care	Patients Perceptio n of Health System	Patients trust in healthcare system	# Injured seeking non-formal care	Willingnes s to seek formal care	# Injured seeking care	# Patients taking other transport	# Patients in Definitive Care on other Transport	# Patients in Definitive Care	# Patients remaining in care/reha b	# Patients receiving Lower Quality Rehab		
821	18	Full-System Multicausal Loop	Total available workforce	GDP	System level funding	Available Funding for injury care	Availability of quality injury care	Discriminatio n	# Patients receiving Lower Quality	Patients Perceptio n of Health System	Patients trust in healthcare system	# Injured seeking non-formal care	Willingnes s to seek formal care	# Injured seeking care	# Patients taking an ambulanc e	# Patients in Definitive Care on Ambulanc e	# Patients in Definitive Care	# Patients remaining in care/reha b	# Patients receiving Lower Quality Rehab		

									Injury Care												
822	18	Full-System Multicausal Loop	Total available workforce	GDP	Poverty	Individual wealth	Availability of other transport	# Patients taking other transport	# Patients in Definitive Care on other Transport	# Patients in Definitive Care	# Patients Receiving Higher Quality Injury care	Patients Perception of Health System	Patients trust in healthcare system	# Injured seeking non-formal care	Willingness to seek formal care	# Injured seeking care	# Patients taking an ambulance	# Patients in Definitive Care on Ambulance	# Patients in Definitive care Delayed		
823	18	Full-System Multicausal Loop	Total available workforce	GDP	System level funding	Available Funding for injury care	Universal health insurance	Ability to pay	Willingness to seek formal care	# Injured seeking care	# Patients taking an ambulance	# Patients in Definitive Care on Ambulance	# Patients in Definitive Care	# Patients Receiving Higher Quality Injury care	Patients Perception of Health System	Patients trust in healthcare system	Willingness to remain in care	# Patients remaining in care/rehab	# Patients receiving Lower Quality Rehab		
824	18	Full-System Multicausal Loop	Total available workforce	GDP	System level funding	Available Funding for injury care	Universal health insurance	Ability to pay	Willingness to seek formal care	# Injured seeking care	# Patients taking other transport	# Patients in Definitive Care on other Transport	# Patients in Definitive Care	# Patients Receiving Higher Quality Injury care	Patients Perception of Health System	Patients trust in healthcare system	Willingness to remain in care	# Patients remaining in care/rehab	# Patients Receiving Higher Quality Rehab		
825	19	Full-System Multicausal Loop	Total available workforce	GDP	Poverty	Community Conflict Violence etc	Availability of quality injury care	Discrimination	# Patients Receiving Higher Quality Injury care	Patients Perception of Health System	Patients trust in healthcare system	Willingness to seek formal care	# Injured seeking care	# Patients taking other transport	# Patients in Definitive Care on other Transport	# Patients in Definitive Care	# Patients receiving Lower Quality Injury Care	Willingness to remain in care	# Patients remaining in care/rehab	# Patients Receiving Higher Quality Rehab	
826	19	Full-System Multicausal Loop	Total available workforce	GDP	Poverty	Community Conflict Violence etc	Availability of quality injury care	Discrimination	# Patients receiving Lower Quality Injury Care	Patients Perception of Health System	Patients trust in healthcare system	Willingness to seek formal care	# Injured seeking care	# Patients taking an ambulance	# Patients in Definitive Care on Ambulance	# Patients in Definitive Care	# Patients Receiving Higher Quality Injury care	Willingness to remain in care	# Patients remaining in care/rehab	# Patients receiving Lower Quality Rehab	
827	19	Full-System Multicausal Loop	Total available workforce	GDP	System level funding	Available Funding for injury care	Availability of quality injury rehab or follow-up	Discrimination	# Patients receiving Lower Quality Injury Care	Patients Perception of Health System	Patients trust in healthcare system	Willingness to seek formal care	# Injured seeking care	# Patients taking an ambulance	# Patients in Definitive Care on Ambulance	# Patients in Definitive Care	# Patients Receiving Higher Quality Injury care	Willingness to remain in care	# Patients remaining in care/rehab	# Patients receiving Lower Quality Rehab	
828	19	Full-System Multicausal Loop	Total available workforce	GDP	System level funding	Available Funding for injury care	Availability of quality injury rehab or follow-up	Discrimination	# Patients receiving Lower Quality Injury Care	Patients Perception of Health System	Patients trust in healthcare system	Willingness to seek formal care	# Injured seeking care	# Patients taking an ambulance	# Patients in Definitive Care on Ambulance	# Patients in Definitive Care	# Patients Receiving Higher Quality Injury care	Willingness to remain in care	# Patients remaining in care/rehab	# Patients Receiving Higher Quality Rehab	
829	19	Full-System Multicausal Loop	Total available workforce	GDP	System level funding	Available Funding for injury care	Availability of quality injury rehab or follow-up	Discrimination	# Patients Receiving Higher Quality Injury care	Patients Perception of Health System	Patients trust in healthcare system	Willingness to seek formal care	# Injured seeking care	# Patients taking other transport	# Patients in Definitive Care on other Transport	# Patients in Definitive Care	# Patients receiving Lower Quality Injury Care	Willingness to remain in care	# Patients remaining in care/rehab	# Patients receiving Lower Quality Rehab	
830	19	Full-System Multicausal Loop	Total available workforce	GDP	System level funding	Available Funding for injury care	Availability of Ambulance Service	Geographical accessibility	Availability of other transport	# Patients taking other transport	# Patients in Definitive Care on other Transport	# Patients in Definitive Care	# Patients receiving Lower Quality Injury Care	Patients Perception of Health System	Patients trust in healthcare system	Willingness to seek formal care	# Injured seeking care	# Patients taking an ambulance	# Patients in Definitive Care on Ambulance	# Patients in Definitive care Delayed	
831	19	Full-System Multicausal Loop	Total available workforce	GDP	Poverty	Community Conflict Violence etc	Availability of quality injury care	Discrimination	# Patients receiving Lower Quality Injury Care	Patients Perception of Health System	Patients trust in healthcare system	Willingness to seek formal care	# Injured seeking care	# Patients taking other transport	# Patients in Definitive Care on other Transport	# Patients in Definitive Care	# Patients Receiving Higher Quality Injury care	Willingness to remain in care	# Patients remaining in care/rehab	# Patients Receiving Higher Quality Rehab	
832	19	Full-System Multicausal Loop	Total available workforce	GDP	System level funding	Available Funding for injury care	Availability of quality injury rehab or follow-up	Discrimination	# Patients Receiving Higher Quality Injury care	Patients Perception of Health System	Patients trust in healthcare system	Willingness to seek formal care	# Injured seeking care	# Patients taking other transport	# Patients in Definitive Care on other Transport	# Patients in Definitive Care	# Patients receiving Lower Quality Injury Care	Willingness to remain in care	# Patients remaining in care/rehab	# Patients Receiving Higher Quality Rehab	
833	19	Full-System Multicausal Loop	Total available workforce	GDP	System level funding	Available Funding for injury care	Availability of quality injury care	Discrimination	# Patients Receiving Higher Quality Injury care	Patients Perception of Health System	Patients trust in healthcare system	Willingness to seek formal care	# Injured seeking care	# Patients taking an ambulance	# Patients in Definitive Care on Ambulance	# Patients in Definitive Care	# Patients receiving Lower Quality Injury Care	Willingness to remain in care	# Patients remaining in care/rehab	# Patients Receiving Higher Quality Rehab	
834	19	Full-System Multicausal Loop	Total available workforce	GDP	Poverty	Community Conflict	Availability	Discrimination	# Patients Receiving	Patients Perceptio	Patients trust in	Willingness to seek	# Injured	# Patients taking an	# Patients in Definitive	# Patients in	# Patients receiving	Willingness to	# Patients remaining	# Patients Receiving	

						Violence etc	of quality injury care		Higher Quality Injury care	n of Health System	healthcare system	formal care	seeking care	ambulanc e	Care on Ambulanc e	Definitive Care	Lower Quality Injury Care	remain in care	in care/reha b	Higher Quality Rehab	
835	19	Full-System Multicausal Loop	Total available workforce	GDP	System level funding	Available Funding for injury care	Availability of Ambulance Service	Geographica l accessibility	Availability of other transport	# Patients taking other transport	# Patients in Definitive Care on other Transport	# Patients in Definitive Care	# Patients Receiving Higher Quality Injury care	Patients Perceptio n of Health System	Patients trust in healthcare system	Willingnes s to seek formal care	# Injured seeking care	# Patients taking an ambulanc e	# Patients in Definitive Care on Ambulanc e	# Patients in Definitive care Delayed	
836	19	Full-System Multicausal Loop	Total available workforce	GDP	Poverty	Communit y Conflict Violence etc	Geographic al accessibility	Availability of Ambulance Service	# Patients taking an ambulanc e	# Patients in Definitive Care on Ambulanc e	# Patients in Definitive Care	# Patients Receiving Higher Quality Injury care	Patients Perceptio n of Health System	Patients trust in healthcare system	# Injured seeking non-formal care	Willingnes s to seek formal care	# Injured seeking care	# Patients taking other transport	# Patients in Definitive Care on other Transport	# Patients in Definitive care on time	
837	19	Full-System Multicausal Loop	Total available workforce	GDP	Poverty	Communit y Conflict Violence etc	Availability of quality injury care	# Patients receiving Lower Quality Injury Care	Patients Perceptio n of Health System	Patients trust in healthcare system	# Injured seeking non-formal care	Willingnes s to seek formal care	# Injured seeking care	# Patients taking an ambulanc e	# Patients in Definitive Care on Ambulanc e	# Patients in Definitive Care	# Patients Receiving Higher Quality Injury care	Willingnes s to remain in care	# Patients remaining in care/reha b	# Patients Receiving Higher Quality Rehab	
838	19	Full-System Multicausal Loop	Total available workforce	GDP	Poverty	Communit y Conflict Violence etc	Availability of quality injury care	Discriminatio n	# Patients receiving Lower Quality Injury Care	Patients Perceptio n of Health System	Patients trust in healthcare system	Willingnes s to seek formal care	# Injured seeking care	# Patients taking other transport	# Patients in Definitive Care on other Transport	# Patients in Definitive Care	# Patients Receiving Higher Quality Injury care	Willingnes s to remain in care	# Patients remaining in care/reha b	# Patients receiving Lower Quality Rehab	
839	19	Full-System Multicausal Loop	Total available workforce	GDP	System level funding	Available Funding for injury care	Availability of quality injury rehab or follow-up	Discriminatio n	# Patients Receiving Higher Quality Injury care	Patients Perceptio n of Health System	Patients trust in healthcare system	Willingnes s to seek formal care	# Injured seeking care	# Patients taking an ambulanc e	# Patients in Definitive Care on Ambulanc e	# Patients in Definitive Care	# Patients receiving Lower Quality Injury Care	Willingnes s to remain in care	# Patients remaining in care/reha b	# Patients receiving Lower Quality Rehab	
840	19	Full-System Multicausal Loop	Total available workforce	GDP	System level funding	Available Funding for injury care	Availability of Ambulance Service	Geographica l accessibility	Availability of other transport	# Patients taking other transport	# Patients in Definitive Care on other Transport	# Patients in Definitive Care	# Patients receiving Lower Quality Injury Care	Patients Perceptio n of Health System	Patients trust in healthcare system	Willingnes s to seek formal care	# Injured seeking care	# Patients taking an ambulanc e	# Patients in Definitive Care on Ambulanc e	# Patients in Definitive care on time	
841	19	Full-System Multicausal Loop	Total available workforce	GDP	Poverty	Communit y Conflict Violence etc	Availability of quality injury rehab or follow-up	Discriminatio n	# Patients Receiving Higher Quality Injury care	Patients Perceptio n of Health System	Patients trust in healthcare system	Willingnes s to seek formal care	# Injured seeking care	# Patients taking other transport	# Patients in Definitive Care on other Transport	# Patients in Definitive Care	# Patients receiving Lower Quality Injury Care	Willingnes s to remain in care	# Patients remaining in care/reha b	# Patients receiving Lower Quality Rehab	
842	19	Full-System Multicausal Loop	Total available workforce	GDP	System level funding	Available Funding for injury care	Availability of quality injury rehab or follow-up	Discriminatio n	# Patients receiving Lower Quality Injury Care	Patients Perceptio n of Health System	Patients trust in healthcare system	Willingnes s to seek formal care	# Injured seeking care	# Patients taking other transport	# Patients in Definitive Care on other Transport	# Patients in Definitive Care	# Patients Receiving Higher Quality Injury care	Willingnes s to remain in care	# Patients remaining in care/reha b	# Patients receiving Lower Quality Rehab	
843	19	Full-System Multicausal Loop	Total available workforce	GDP	Poverty	Communit y Conflict Violence etc	Availability of quality injury care	Discriminatio n	# Patients Receiving Higher Quality Injury care	Patients Perceptio n of Health System	Patients trust in healthcare system	Willingnes s to seek formal care	# Injured seeking care	# Patients taking other transport	# Patients in Definitive Care on other Transport	# Patients in Definitive Care	# Patients receiving Lower Quality Injury Care	Willingnes s to remain in care	# Patients remaining in care/reha b	# Patients receiving Lower Quality Rehab	
844	19	Full-System Multicausal Loop	Total available workforce	GDP	System level funding	Available Funding for injury care	Availability of quality injury care	Discriminatio n	# Patients Receiving Higher Quality Injury care	Patients Perceptio n of Health System	Patients trust in healthcare system	Willingnes s to seek formal care	# Injured seeking care	# Patients taking other transport	# Patients in Definitive Care on other Transport	# Patients in Definitive Care	# Patients receiving Lower Quality Injury Care	Willingnes s to remain in care	# Patients remaining in care/reha b	# Patients Receiving Higher Quality Rehab	
845	19	Full-System Multicausal Loop	Total available workforce	GDP	Poverty	Communit y Conflict Violence etc	Availability of quality injury care	# Patients Receiving Higher Quality Injury care	Patients Perceptio n of Health System	Patients trust in healthcare system	# Injured seeking non-formal care	Willingnes s to seek formal care	# Injured seeking care	# Patients taking other transport	# Patients in Definitive Care on other Transport	# Patients in Definitive Care	# Patients receiving Lower Quality Injury Care	Willingnes s to remain in care	# Patients remaining in care/reha b	# Patients Receiving Higher Quality Rehab	
846	19	Full-System Multicausal Loop	Total available workforce	GDP	System level funding	Available Funding for injury care	Availability of quality injury rehab or follow-up	Discriminatio n	# Patients receiving Lower Quality Injury Care	Patients Perceptio n of Health System	Patients trust in healthcare system	Willingnes s to seek formal care	# Injured seeking care	# Patients taking other transport	# Patients in Definitive Care on other Transport	# Patients in Definitive Care	# Patients Receiving Higher Quality Injury care	Willingnes s to remain in care	# Patients remaining in care/reha b	# Patients Receiving Higher Quality Rehab	

847	19	Full-System Multicausal Loop	Total available workforce	GDP	Poverty	Community Conflict Violence etc	Geographical accessibility	Availability of other transport	# Patients taking other transport	# Patients in Definitive Care on other Transport	# Patients in Definitive Care	# Patients receiving Lower Quality Injury Care	Patients Perception of Health System	Patients trust in healthcare system	# Injured seeking non-formal care	Willingness to seek formal care	# Injured seeking care	# Patients taking an ambulance	# Patients in Definitive Care on Ambulance	# Patients in Definitive care on time	
848	19	Full-System Multicausal Loop	Total available workforce	GDP	Poverty	Community Conflict Violence etc	Availability of quality injury rehab or follow-up	Discrimination	# Patients receiving Lower Quality Injury Care	Patients Perception of Health System	Patients trust in healthcare system	Willingness to seek formal care	# Injured seeking care	# Patients taking other transport	# Patients in Definitive Care on other Transport	# Patients in Definitive Care	# Patients Receiving Higher Quality Injury care	Willingness to remain in care	# Patients remaining in care/rehab	# Patients receiving Lower Quality Rehab	
849	19	Full-System Multicausal Loop	Total available workforce	GDP	Poverty	Community Conflict Violence etc	Availability of quality injury care	# Patients receiving Lower Quality Injury Care	Patients Perception of Health System	Patients trust in healthcare system	# Injured seeking non-formal care	Willingness to seek formal care	# Injured seeking care	# Patients taking other transport	# Patients in Definitive Care on other Transport	# Patients in Definitive Care	# Patients Receiving Higher Quality Injury care	Willingness to remain in care	# Patients remaining in care/rehab	# Patients Receiving Higher Quality Rehab	
850	19	Full-System Multicausal Loop	Total available workforce	GDP	Poverty	Individual wealth	Availability of other transport	Geographical accessibility	Availability of Ambulance Service	# Patients taking an ambulance	# Patients in Definitive Care on Ambulance	# Patients in Definitive Care	# Patients receiving Lower Quality Injury Care	Patients Perception of Health System	Patients trust in healthcare system	Willingness to seek formal care	# Injured seeking care	# Patients taking other transport	# Patients in Definitive Care on other Transport	# Patients in Definitive care on time	
851	19	Full-System Multicausal Loop	Total available workforce	GDP	System level funding	Available Funding for injury care	Availability of quality injury care	# Patients Receiving Higher Quality Injury care	Patients Perception of Health System	Patients trust in healthcare system	# Injured seeking non-formal care	Willingness to seek formal care	# Injured seeking care	# Patients taking other transport	# Patients in Definitive Care on other Transport	# Patients in Definitive Care	# Patients receiving Lower Quality Injury Care	Willingness to remain in care	# Patients remaining in care/rehab	# Patients Receiving Higher Quality Rehab	
852	19	Full-System Multicausal Loop	Total available workforce	GDP	System level funding	Available Funding for injury care	Availability of quality injury care	Discrimination	# Patients receiving Lower Quality Injury Care	Patients Perception of Health System	Patients trust in healthcare system	Willingness to seek formal care	# Injured seeking care	# Patients taking an ambulance	# Patients in Definitive Care on Ambulance	# Patients in Definitive Care	# Patients Receiving Higher Quality Injury care	Willingness to remain in care	# Patients remaining in care/rehab	# Patients receiving Lower Quality Rehab	
853	19	Full-System Multicausal Loop	Total available workforce	GDP	Poverty	Community Conflict Violence etc	Availability of quality injury care	# Patients receiving Lower Quality Injury Care	Patients Perception of Health System	Patients trust in healthcare system	# Injured seeking non-formal care	Willingness to seek formal care	# Injured seeking care	# Patients taking an ambulance	# Patients in Definitive Care on Ambulance	# Patients in Definitive Care	# Patients Receiving Higher Quality Injury care	Willingness to remain in care	# Patients remaining in care/rehab	# Patients receiving Lower Quality Rehab	
854	19	Full-System Multicausal Loop	Total available workforce	GDP	Poverty	Community Conflict Violence etc	Availability of quality injury care	# Patients Receiving Higher Quality Injury care	Patients Perception of Health System	Patients trust in healthcare system	# Injured seeking non-formal care	Willingness to seek formal care	# Injured seeking care	# Patients taking an ambulance	# Patients in Definitive Care on Ambulance	# Patients in Definitive Care	# Patients receiving Lower Quality Injury Care	Willingness to remain in care	# Patients remaining in care/rehab	# Patients Receiving Higher Quality Rehab	
855	19	Full-System Multicausal Loop	Total available workforce	GDP	Poverty	Community Conflict Violence etc	Availability of quality injury care	Discrimination	# Patients receiving Lower Quality Injury Care	Patients Perception of Health System	Patients trust in healthcare system	Willingness to seek formal care	# Injured seeking care	# Patients taking an ambulance	# Patients in Definitive Care on Ambulance	# Patients in Definitive Care	# Patients Receiving Higher Quality Injury care	Willingness to remain in care	# Patients remaining in care/rehab	# Patients Receiving Higher Quality Rehab	
856	19	Full-System Multicausal Loop	Total available workforce	GDP	Poverty	Community Conflict Violence etc	Availability of quality injury rehab or follow-up	Discrimination	# Patients Receiving Higher Quality Injury care	Patients Perception of Health System	Patients trust in healthcare system	Willingness to seek formal care	# Injured seeking care	# Patients taking other transport	# Patients in Definitive Care on other Transport	# Patients in Definitive Care	# Patients receiving Lower Quality Injury Care	Willingness to remain in care	# Patients remaining in care/rehab	# Patients Receiving Higher Quality Rehab	
857	19	Full-System Multicausal Loop	Total available workforce	GDP	Poverty	Community Conflict Violence etc	Geographical accessibility	Availability of Ambulance Service	# Patients taking an ambulance	# Patients in Definitive Care on Ambulance	# Patients in Definitive Care	# Patients receiving Lower Quality Injury Care	Patients Perception of Health System	Patients trust in healthcare system	# Injured seeking non-formal care	Willingness to seek formal care	# Injured seeking care	# Patients taking other transport	# Patients in Definitive Care on other Transport	# Patients in Definitive care Delayed	
858	19	Full-System Multicausal Loop	Total available workforce	GDP	Poverty	Community Conflict Violence etc	Availability of quality injury care	# Patients receiving Lower Quality Injury Care	Patients Perception of Health System	Patients trust in healthcare system	# Injured seeking non-formal care	Willingness to seek formal care	# Injured seeking care	# Patients taking other transport	# Patients in Definitive Care on other Transport	# Patients in Definitive Care	# Patients Receiving Higher Quality Injury care	Willingness to remain in care	# Patients remaining in care/rehab	# Patients receiving Lower Quality Rehab	
859	19	Full-System Multicausal Loop	Total available workforce	GDP	System level funding	Available Funding for injury care	Availability of quality injury care	Discrimination	# Patients receiving Lower Quality	Patients Perception of Health System	Patients trust in healthcare system	Willingness to seek formal care	# Injured seeking care	# Patients taking other transport	# Patients in Definitive Care on other Transport	# Patients in Definitive Care	# Patients Receiving Higher Quality Injury care	Willingness to remain in care	# Patients remaining in care/rehab	# Patients Receiving Higher Quality Rehab	

									Injury Care												
860	19	Full-System Multicausal Loop	Total available workforce	GDP	System level funding	Available Funding for injury care	Availability of quality injury care	Discrimination	# Patients Receiving Higher Quality Injury care	Patients Perception of Health System	Patients trust in healthcare system	Willingness to seek formal care	# Injured seeking care	# Patients taking other transport	# Patients in Definitive Care on other Transport	# Patients in Definitive Care	# Patients receiving Lower Quality Injury Care	Willingness to remain in care	# Patients remaining in care/rehab	# Patients receiving Lower Quality Rehab	
861	19	Full-System Multicausal Loop	Total available workforce	GDP	Poverty	Community Conflict Violence etc	Geographical accessibility	Availability of other transport	# Patients taking other transport	# Patients in Definitive Care on other Transport	# Patients in Definitive Care	# Patients Receiving Higher Quality Injury care	Patients Perception of Health System	Patients trust in healthcare system	# Injured seeking non-formal care	Willingness to seek formal care	# Injured seeking care	# Patients taking an ambulance	# Patients in Definitive Care on Ambulance	# Patients in Definitive care on time	
862	19	Full-System Multicausal Loop	Total available workforce	GDP	Poverty	Community Conflict Violence etc	Availability of quality injury care	Discrimination	# Patients Receiving Higher Quality Injury care	Patients Perception of Health System	Patients trust in healthcare system	Willingness to seek formal care	# Injured seeking care	# Patients taking an ambulance	# Patients in Definitive Care on Ambulance	# Patients in Definitive Care	# Patients receiving Lower Quality Injury Care	Willingness to remain in care	# Patients remaining in care/rehab	# Patients receiving Lower Quality Rehab	
863	19	Full-System Multicausal Loop	Total available workforce	GDP	Poverty	Community Conflict Violence etc	Geographical accessibility	Availability of Ambulance Service	# Patients taking an ambulance	# Patients in Definitive Care on Ambulance	# Patients in Definitive Care	# Patients receiving Lower Quality Injury Care	Patients Perception of Health System	Patients trust in healthcare system	# Injured seeking non-formal care	Willingness to seek formal care	# Injured seeking care	# Patients taking other transport	# Patients in Definitive Care on other Transport	# Patients in Definitive care on time	
864	19	Full-System Multicausal Loop	Total available workforce	GDP	Poverty	Individual wealth	Availability of other transport	Geographical accessibility	Availability of Ambulance Service	# Patients taking an ambulance	# Patients in Definitive Care on Ambulance	# Patients in Definitive Care	# Patients Receiving Higher Quality Injury care	Patients Perception of Health System	Patients trust in healthcare system	Willingness to seek formal care	# Injured seeking care	# Patients taking other transport	# Patients in Definitive Care on other Transport	# Patients in Definitive care Delayed	
865	19	Full-System Multicausal Loop	Total available workforce	GDP	System level funding	Available Funding for injury care	Availability of quality injury care	# Patients receiving Lower Quality Injury Care	Patients Perception of Health System	Patients trust in healthcare system	# Injured seeking non-formal care	Willingness to seek formal care	# Injured seeking care	# Patients taking other transport	# Patients in Definitive Care on other Transport	# Patients in Definitive Care	# Patients Receiving Higher Quality Injury care	Willingness to remain in care	# Patients remaining in care/rehab	# Patients receiving Lower Quality Rehab	
866	19	Full-System Multicausal Loop	Total available workforce	GDP	System level funding	Available Funding for injury care	Availability of quality injury care	# Patients receiving Lower Quality Injury Care	Patients Perception of Health System	Patients trust in healthcare system	# Injured seeking non-formal care	Willingness to seek formal care	# Injured seeking care	# Patients taking other transport	# Patients in Definitive Care on other Transport	# Patients in Definitive Care	# Patients Receiving Higher Quality Injury care	Willingness to remain in care	# Patients remaining in care/rehab	# Patients Receiving Higher Quality Rehab	
867	19	Full-System Multicausal Loop	Total available workforce	GDP	Poverty	Community Conflict Violence etc	Availability of quality injury care	# Patients Receiving Higher Quality Injury care	Patients Perception of Health System	Patients trust in healthcare system	# Injured seeking non-formal care	Willingness to seek formal care	# Injured seeking care	# Patients taking an ambulance	# Patients in Definitive Care on Ambulance	# Patients in Definitive Care	# Patients receiving Lower Quality Injury Care	Willingness to remain in care	# Patients remaining in care/rehab	# Patients receiving Lower Quality Rehab	
868	19	Full-System Multicausal Loop	Total available workforce	GDP	System level funding	Available Funding for injury care	Availability of quality injury care	Discrimination	# Patients receiving Lower Quality Injury Care	Patients Perception of Health System	Patients trust in healthcare system	Willingness to seek formal care	# Injured seeking care	# Patients taking an ambulance	# Patients in Definitive Care on Ambulance	# Patients in Definitive Care	# Patients Receiving Higher Quality Injury care	Willingness to remain in care	# Patients remaining in care/rehab	# Patients Receiving Higher Quality Rehab	
869	19	Full-System Multicausal Loop	Total available workforce	GDP	System level funding	Available Funding for injury care	Availability of quality injury care	# Patients Receiving Higher Quality Injury care	Patients Perception of Health System	Patients trust in healthcare system	# Injured seeking non-formal care	Willingness to seek formal care	# Injured seeking care	# Patients taking an ambulance	# Patients in Definitive Care on Ambulance	# Patients in Definitive Care	# Patients receiving Lower Quality Injury Care	Willingness to remain in care	# Patients remaining in care/rehab	# Patients Receiving Higher Quality Rehab	
870	19	Full-System Multicausal Loop	Total available workforce	GDP	System level funding	Available Funding for injury care	Availability of quality injury rehab or follow-up	Discrimination	# Patients Receiving Higher Quality Injury care	Patients Perception of Health System	Patients trust in healthcare system	Willingness to seek formal care	# Injured seeking care	# Patients taking an ambulance	# Patients in Definitive Care on Ambulance	# Patients in Definitive Care	# Patients receiving Lower Quality Injury Care	Willingness to remain in care	# Patients remaining in care/rehab	# Patients Receiving Higher Quality Rehab	
871	19	Full-System Multicausal Loop	Total available workforce	GDP	System level funding	Available Funding for injury care	Availability of Ambulance Service	Geographical accessibility	Availability of other transport	# Patients taking other transport	# Patients in Definitive Care on other Transport	# Patients in Definitive Care	# Patients Receiving Higher Quality Injury care	Patients Perception of Health System	Patients trust in healthcare system	Willingness to seek formal care	# Injured seeking care	# Patients taking an ambulance	# Patients in Definitive Care on Ambulance	# Patients in Definitive care on time	
872	19	Full-System Multicausal Loop	Total available workforce	GDP	System level funding	Available Funding for injury care	Availability of quality injury care	Discrimination	# Patients Receiving Higher	Patients Perception of	Patients trust in healthcare system	Willingness to seek formal care	# Injured seeking care	# Patients taking an ambulance	# Patients in Definitive Care on	# Patients in Definitive Care	# Patients receiving Lower Quality	Willingness to remain in care	# Patients remaining in	# Patients receiving Lower	

									Quality Injury care	Health System					Ambulance		Injury Care		care/rehab	Quality Rehab	
873	19	Full-System Multicausal Loop	Total available workforce	GDP	Poverty	Community Conflict Violence etc	Availability of quality injury care	# Patients Receiving Higher Quality Injury care	Patients Perception of Health System	Patients trust in healthcare system	# Injured seeking non-formal care	Willingness to seek formal care	# Injured seeking care	# Patients taking other transport	# Patients in Definitive Care on other Transport	# Patients in Definitive Care	# Patients receiving Lower Quality Injury Care	Willingness to remain in care	# Patients remaining in care/rehab	# Patients receiving Lower Quality Rehab	
874	19	Full-System Multicausal Loop	Total available workforce	GDP	System level funding	Available Funding for injury care	Availability of quality injury care	# Patients Receiving Higher Quality Injury care	Patients Perception of Health System	Patients trust in healthcare system	# Injured seeking non-formal care	Willingness to seek formal care	# Injured seeking care	# Patients taking an ambulance	# Patients in Definitive Care on Ambulance	# Patients in Definitive Care	# Patients receiving Lower Quality Injury Care	Willingness to remain in care	# Patients remaining in care/rehab	# Patients receiving Lower Quality Rehab	
875	19	Full-System Multicausal Loop	Total available workforce	GDP	Poverty	Community Conflict Violence etc	Availability of quality injury rehab or follow-up	Discrimination	# Patients receiving Lower Quality Injury Care	Patients Perception of Health System	Patients trust in healthcare system	Willingness to seek formal care	# Injured seeking care	# Patients taking other transport	# Patients in Definitive Care on other Transport	# Patients in Definitive Care	# Patients Receiving Higher Quality Injury care	Willingness to remain in care	# Patients remaining in care/rehab	# Patients Receiving Higher Quality Rehab	
876	19	Full-System Multicausal Loop	Total available workforce	GDP	System level funding	Available Funding for injury care	Availability of quality injury care	# Patients Receiving Higher Quality Injury care	Patients Perception of Health System	Patients trust in healthcare system	# Injured seeking non-formal care	Willingness to seek formal care	# Injured seeking care	# Patients taking other transport	# Patients in Definitive Care on other Transport	# Patients in Definitive Care	# Patients receiving Lower Quality Injury Care	Willingness to remain in care	# Patients remaining in care/rehab	# Patients receiving Lower Quality Rehab	
877	19	Full-System Multicausal Loop	Total available workforce	GDP	Poverty	Community Conflict Violence etc	Geographical accessibility	Availability of other transport	# Patients taking other transport	# Patients in Definitive Care on other Transport	# Patients in Definitive Care	# Patients receiving Lower Quality Injury Care	Patients Perception of Health System	Patients trust in healthcare system	# Injured seeking non-formal care	Willingness to seek formal care	# Injured seeking care	# Patients taking an ambulance	# Patients in Definitive Care on Ambulance	# Patients in Definitive care Delayed	
878	19	Full-System Multicausal Loop	Total available workforce	GDP	Poverty	Individual wealth	Availability of other transport	Geographical accessibility	Availability of Ambulance Service	# Patients taking an ambulance	# Patients in Definitive Care on Ambulance	# Patients in Definitive Care	# Patients receiving Lower Quality Injury Care	Patients Perception of Health System	Patients trust in healthcare system	Willingness to seek formal care	# Injured seeking care	# Patients taking other transport	# Patients in Definitive Care on other Transport	# Patients in Definitive care Delayed	
879	19	Full-System Multicausal Loop	Total available workforce	GDP	System level funding	Available Funding for injury care	Availability of quality injury care	# Patients receiving Lower Quality Injury Care	Patients Perception of Health System	Patients trust in healthcare system	# Injured seeking non-formal care	Willingness to seek formal care	# Injured seeking care	# Patients taking an ambulance	# Patients in Definitive Care on Ambulance	# Patients in Definitive Care	# Patients Receiving Higher Quality Injury care	Willingness to remain in care	# Patients remaining in care/rehab	# Patients Receiving Higher Quality Rehab	
880	19	Full-System Multicausal Loop	Total available workforce	GDP	System level funding	Available Funding for injury care	Availability of quality injury care	Discrimination	# Patients receiving Lower Quality Injury Care	Patients Perception of Health System	Patients trust in healthcare system	Willingness to seek formal care	# Injured seeking care	# Patients taking other transport	# Patients in Definitive Care on other Transport	# Patients in Definitive Care	# Patients Receiving Higher Quality Injury care	Willingness to remain in care	# Patients remaining in care/rehab	# Patients receiving Lower Quality Rehab	
881	19	Full-System Multicausal Loop	Total available workforce	GDP	Poverty	Community Conflict Violence etc	Geographical accessibility	Availability of Ambulance Service	# Patients taking an ambulance	# Patients in Definitive Care on Ambulance	# Patients in Definitive Care	# Patients Receiving Higher Quality Injury care	Patients Perception of Health System	Patients trust in healthcare system	# Injured seeking non-formal care	Willingness to seek formal care	# Injured seeking care	# Patients taking other transport	# Patients in Definitive Care on other Transport	# Patients in Definitive care Delayed	
882	19	Full-System Multicausal Loop	Total available workforce	GDP	Poverty	Community Conflict Violence etc	Availability of quality injury rehab or follow-up	Discrimination	# Patients receiving Lower Quality Injury Care	Patients Perception of Health System	Patients trust in healthcare system	Willingness to seek formal care	# Injured seeking care	# Patients taking an ambulance	# Patients in Definitive Care on Ambulance	# Patients in Definitive Care	# Patients Receiving Higher Quality Injury care	Willingness to remain in care	# Patients remaining in care/rehab	# Patients Receiving Higher Quality Rehab	
883	19	Full-System Multicausal Loop	Total available workforce	GDP	System level funding	Available Funding for injury care	Availability of quality injury care	# Patients receiving Lower Quality Injury Care	Patients Perception of Health System	Patients trust in healthcare system	# Injured seeking non-formal care	Willingness to seek formal care	# Injured seeking care	# Patients taking an ambulance	# Patients in Definitive Care on Ambulance	# Patients in Definitive Care	# Patients Receiving Higher Quality Injury care	Willingness to remain in care	# Patients remaining in care/rehab	# Patients receiving Lower Quality Rehab	
884	19	Full-System Multicausal Loop	Total available workforce	GDP	Poverty	Community Conflict Violence etc	Availability of quality injury rehab or follow-up	Discrimination	# Patients Receiving Higher Quality Injury care	Patients Perception of Health System	Patients trust in healthcare system	Willingness to seek formal care	# Injured seeking care	# Patients taking an ambulance	# Patients in Definitive Care on Ambulance	# Patients in Definitive Care	# Patients receiving Lower Quality Injury Care	Willingness to remain in care	# Patients remaining in care/rehab	# Patients Receiving Higher Quality Rehab	
885	19	Full-System Multicausal Loop	Total available workforce	GDP	Poverty	Community Conflict	Geographic	Availability of	# Patients taking	# Patients in Definitive	# Patients in	# Patients Receiving	Patients Perceptio	Patients trust in	# Injured seeking	Willingness to seek	# Injured	# Patients taking an	# Patients in Definitive	# Patients in Definitive	

						Violence etc	al accessibility	other transport	other transport	Care on other Transport	Definitive Care	Higher Quality Injury care	n of Health System	healthcare system	non-formal care	formal care	seeking care	ambulance	Care on Ambulance	care Delayed	
886	19	Full-System Multicausal Loop	Total available workforce	GDP	Poverty	Individual wealth	Availability of other transport	Geographical accessibility	Availability of Ambulance Service	# Patients taking an ambulance	# Patients in Definitive Care on Ambulance	# Patients in Definitive Care	# Patients Receiving Higher Quality Injury care	Patients Perception of Health System	Patients trust in healthcare system	Willingness to seek formal care	# Injured seeking care	# Patients taking other transport	# Patients in Definitive Care on other Transport	# Patients in Definitive care on time	
887	19	Full-System Multicausal Loop	Total available workforce	GDP	Poverty	Community Conflict Violence etc	Availability of quality injury rehab or follow-up	Discrimination	# Patients Receiving Higher Quality Injury care	Patients Perception of Health System	Patients trust in healthcare system	Willingness to seek formal care	# Injured seeking care	# Patients taking ambulance	# Patients in Definitive Care on Ambulance	# Patients in Definitive Care	# Patients receiving Lower Quality Injury Care	Willingness to remain in care	# Patients remaining in care/rehab	# Patients receiving Lower Quality Rehab	
888	19	Full-System Multicausal Loop	Total available workforce	GDP	Poverty	Community Conflict Violence etc	Availability of quality injury rehab or follow-up	Discrimination	# Patients receiving Lower Quality Injury Care	Patients Perception of Health System	Patients trust in healthcare system	Willingness to seek formal care	# Injured seeking care	# Patients taking an ambulance	# Patients in Definitive Care on Ambulance	# Patients in Definitive Care	# Patients Receiving Higher Quality Injury care	Willingness to remain in care	# Patients remaining in care/rehab	# Patients receiving Lower Quality Rehab	
889	20	Full-System Multicausal Loop	Total available workforce	GDP	Poverty	Community Conflict Violence etc	Availability of quality injury rehab or follow-up	Discrimination	# Patients receiving Lower Quality Injury Care	Patients Perception of Health System	Patients trust in healthcare system	# Injured seeking non-formal care	Willingness to seek formal care	# Injured seeking care	# Patients taking other transport	# Patients in Definitive Care on other Transport	# Patients in Definitive Care	# Patients Receiving Higher Quality Injury care	Willingness to remain in care	# Patients remaining in care/rehab	# Patients receiving Lower Quality Rehab
890	20	Full-System Multicausal Loop	Total available workforce	GDP	Poverty	Individual wealth	Availability of other transport	Geographical accessibility	Availability of Ambulance Service	# Patients taking an ambulance	# Patients in Definitive Care on Ambulance	# Patients in Definitive Care	# Patients receiving Lower Quality Injury Care	Patients Perception of Health System	Patients trust in healthcare system	# Injured seeking non-formal care	Willingness to seek formal care	# Injured seeking care	# Patients taking other transport	# Patients in Definitive Care on other Transport	# Patients in Definitive care on time
891	20	Full-System Multicausal Loop	Total available workforce	GDP	Poverty	Community Conflict Violence etc	Availability of quality injury care	Discrimination	# Patients receiving Lower Quality Injury Care	Patients Perception of Health System	Patients trust in healthcare system	# Injured seeking non-formal care	Willingness to seek formal care	# Injured seeking care	# Patients taking other transport	# Patients in Definitive Care on other Transport	# Patients in Definitive Care	# Patients Receiving Higher Quality Injury care	Willingness to remain in care	# Patients remaining in care/rehab	# Patients Receiving Higher Quality Rehab
892	20	Full-System Multicausal Loop	Total available workforce	GDP	Poverty	Community Conflict Violence etc	Availability of quality injury care	Discrimination	# Patients Receiving Higher Quality Injury care	Patients Perception of Health System	Patients trust in healthcare system	# Injured seeking non-formal care	Willingness to seek formal care	# Injured seeking care	# Patients taking other transport	# Patients in Definitive Care on other Transport	# Patients in Definitive Care	# Patients receiving Lower Quality Injury Care	Willingness to remain in care	# Patients remaining in care/rehab	# Patients receiving Lower Quality Rehab
893	20	Full-System Multicausal Loop	Total available workforce	GDP	System level funding	Available Funding for injury care	Availability of quality injury rehab or follow-up	Discrimination	# Patients Receiving Higher Quality Injury care	Patients Perception of Health System	Patients trust in healthcare system	# Injured seeking non-formal care	Willingness to seek formal care	# Injured seeking care	# Patients taking an ambulance	# Patients in Definitive Care on Ambulance	# Patients in Definitive Care	# Patients receiving Lower Quality Injury Care	Willingness to remain in care	# Patients remaining in care/rehab	# Patients Receiving Higher Quality Rehab
894	20	Full-System Multicausal Loop	Total available workforce	GDP	System level funding	Available Funding for injury care	Availability of quality injury care	Discrimination	# Patients Receiving Higher Quality Injury care	Patients Perception of Health System	Patients trust in healthcare system	# Injured seeking non-formal care	Willingness to seek formal care	# Injured seeking care	# Patients taking an ambulance	# Patients in Definitive Care on Ambulance	# Patients in Definitive Care	# Patients receiving Lower Quality Injury Care	Willingness to remain in care	# Patients remaining in care/rehab	# Patients Receiving Higher Quality Rehab
895	20	Full-System Multicausal Loop	Total available workforce	GDP	System level funding	Available Funding for injury care	Availability of quality injury care	Discrimination	# Patients receiving Lower Quality Injury Care	Patients Perception of Health System	Patients trust in healthcare system	# Injured seeking non-formal care	Willingness to seek formal care	# Injured seeking care	# Patients taking other transport	# Patients in Definitive Care on other Transport	# Patients in Definitive Care	# Patients Receiving Higher Quality Injury care	Willingness to remain in care	# Patients remaining in care/rehab	# Patients Receiving Higher Quality Rehab
896	20	Full-System Multicausal Loop	Total available workforce	GDP	Poverty	Community Conflict Violence etc	Availability of quality injury care	Discrimination	# Patients Receiving Higher Quality Injury care	Patients Perception of Health System	Patients trust in healthcare system	# Injured seeking non-formal care	Willingness to seek formal care	# Injured seeking care	# Patients taking other transport	# Patients in Definitive Care on other Transport	# Patients in Definitive Care	# Patients receiving Lower Quality Injury Care	Willingness to remain in care	# Patients remaining in care/rehab	# Patients Receiving Higher Quality Rehab
897	20	Full-System Multicausal Loop	Total available workforce	GDP	Poverty	Individual wealth	Availability of other transport	Geographical accessibility	Availability of Ambulance Service	# Patients taking an ambulance	# Patients in Definitive Care on Ambulance	# Patients in Definitive Care	# Patients receiving Lower Quality Injury Care	Patients Perception of Health System	Patients trust in healthcare system	# Injured seeking non-formal care	Willingness to seek formal care	# Injured seeking care	# Patients taking other transport	# Patients in Definitive Care on other Transport	# Patients in Definitive care Delayed

898	20	Full-System Multicausal Loop	Total available workforce	GDP	Poverty	Community Conflict Violence etc	Availability of quality injury care	Discrimination	# Patients Receiving Higher Quality Injury care	Patients Perception of Health System	Patients trust in healthcare system	# Injured seeking non-formal care	Willingness to seek formal care	# Injured seeking care	# Patients taking an ambulance	# Patients in Definitive Care on Ambulance	# Patients in Definitive Care	# Patients receiving Lower Quality Injury Care	Willingness to remain in care	# Patients remaining in care/rehab	# Patients Receiving Higher Quality Rehab
899	20	Full-System Multicausal Loop	Total available workforce	GDP	Poverty	Community Conflict Violence etc	Availability of quality injury care	Discrimination	# Patients receiving Lower Quality Injury Care	Patients Perception of Health System	Patients trust in healthcare system	# Injured seeking non-formal care	Willingness to seek formal care	# Injured seeking care	# Patients taking an ambulance	# Patients in Definitive Care on Ambulance	# Patients in Definitive Care	# Patients Receiving Higher Quality Injury care	Willingness to remain in care	# Patients remaining in care/rehab	# Patients Receiving Higher Quality Rehab
900	20	Full-System Multicausal Loop	Total available workforce	GDP	Poverty	Community Conflict Violence etc	Availability of quality injury care	Discrimination	# Patients Receiving Higher Quality Injury care	Patients Perception of Health System	Patients trust in healthcare system	# Injured seeking non-formal care	Willingness to seek formal care	# Injured seeking care	# Patients taking an ambulance	# Patients in Definitive Care on Ambulance	# Patients in Definitive Care	# Patients receiving Lower Quality Injury Care	Willingness to remain in care	# Patients remaining in care/rehab	# Patients receiving Lower Quality Rehab
901	20	Full-System Multicausal Loop	Total available workforce	GDP	System level funding	Available Funding for injury care	Availability of quality injury care	Discrimination	# Patients Receiving Higher Quality Injury care	Patients Perception of Health System	Patients trust in healthcare system	# Injured seeking non-formal care	Willingness to seek formal care	# Injured seeking care	# Patients taking other transport	# Patients in Definitive Care on other Transport	# Patients in Definitive Care	# Patients receiving Lower Quality Injury Care	Willingness to remain in care	# Patients remaining in care/rehab	# Patients receiving Lower Quality Rehab
902	20	Full-System Multicausal Loop	Total available workforce	GDP	Poverty	Community Conflict Violence etc	Availability of quality injury care	Discrimination	# Patients receiving Lower Quality Injury Care	Patients Perception of Health System	Patients trust in healthcare system	# Injured seeking non-formal care	Willingness to seek formal care	# Injured seeking care	# Patients taking other transport	# Patients in Definitive Care on other Transport	# Patients in Definitive Care	# Patients Receiving Higher Quality Injury care	Willingness to remain in care	# Patients remaining in care/rehab	# Patients receiving Lower Quality Rehab
903	20	Full-System Multicausal Loop	Total available workforce	GDP	System level funding	Available Funding for injury care	Availability of Ambulance Service	Geographical accessibility	Availability of other transport	# Patients taking other transport	# Patients in Definitive Care on other Transport	# Patients in Definitive Care	# Patients Receiving Higher Quality Injury care	Patients Perception of Health System	Patients trust in healthcare system	# Injured seeking non-formal care	Willingness to seek formal care	# Injured seeking care	# Patients taking an ambulance	# Patients in Definitive Care on Ambulance	# Patients in Definitive care on time
904	20	Full-System Multicausal Loop	Total available workforce	GDP	System level funding	Available Funding for injury care	Availability of quality injury rehab or follow-up	Discrimination	# Patients Receiving Higher Quality Injury care	Patients Perception of Health System	Patients trust in healthcare system	# Injured seeking non-formal care	Willingness to seek formal care	# Injured seeking care	# Patients taking other transport	# Patients in Definitive Care on other Transport	# Patients in Definitive Care	# Patients receiving Lower Quality Injury Care	Willingness to remain in care	# Patients remaining in care/rehab	# Patients receiving Lower Quality Rehab
905	20	Full-System Multicausal Loop	Total available workforce	GDP	System level funding	Available Funding for injury care	Availability of quality injury rehab or follow-up	Discrimination	# Patients Receiving Higher Quality Injury care	Patients Perception of Health System	Patients trust in healthcare system	# Injured seeking non-formal care	Willingness to seek formal care	# Injured seeking care	# Patients taking other transport	# Patients in Definitive Care on other Transport	# Patients in Definitive Care	# Patients receiving Lower Quality Injury Care	Willingness to remain in care	# Patients remaining in care/rehab	# Patients Receiving Higher Quality Rehab
906	20	Full-System Multicausal Loop	Total available workforce	GDP	System level funding	Available Funding for injury care	Availability of quality injury care	Discrimination	# Patients receiving Lower Quality Injury Care	Patients Perception of Health System	Patients trust in healthcare system	# Injured seeking non-formal care	Willingness to seek formal care	# Injured seeking care	# Patients taking an ambulance	# Patients in Definitive Care on Ambulance	# Patients in Definitive Care	# Patients Receiving Higher Quality Injury care	Willingness to remain in care	# Patients remaining in care/rehab	# Patients Receiving Higher Quality Rehab
907	20	Full-System Multicausal Loop	Total available workforce	GDP	Poverty	Community Conflict Violence etc	Availability of quality injury rehab or follow-up	Discrimination	# Patients receiving Lower Quality Injury Care	Patients Perception of Health System	Patients trust in healthcare system	# Injured seeking non-formal care	Willingness to seek formal care	# Injured seeking care	# Patients taking an ambulance	# Patients in Definitive Care on Ambulance	# Patients in Definitive Care	# Patients Receiving Higher Quality Injury care	Willingness to remain in care	# Patients remaining in care/rehab	# Patients Receiving Higher Quality Rehab
908	20	Full-System Multicausal Loop	Total available workforce	GDP	System level funding	Available Funding for injury care	Availability of Ambulance Service	Geographical accessibility	Availability of other transport	# Patients taking other transport	# Patients in Definitive Care on other Transport	# Patients in Definitive Care	# Patients Receiving Higher Quality Injury care	Patients Perception of Health System	Patients trust in healthcare system	# Injured seeking non-formal care	Willingness to seek formal care	# Injured seeking care	# Patients taking an ambulance	# Patients in Definitive Care on Ambulance	# Patients in Definitive care Delayed
909	20	Full-System Multicausal Loop	Total available workforce	GDP	System level funding	Available Funding for injury care	Availability of Ambulance Service	Geographical accessibility	Availability of other transport	# Patients taking other transport	# Patients in Definitive Care on other Transport	# Patients in Definitive Care	# Patients receiving Lower Quality Injury Care	Patients Perception of Health System	Patients trust in healthcare system	# Injured seeking non-formal care	Willingness to seek formal care	# Injured seeking care	# Patients taking an ambulance	# Patients in Definitive Care on Ambulance	# Patients in Definitive care Delayed
910	20	Full-System Multicausal Loop	Total available workforce	GDP	System level funding	Available Funding for injury care	Availability of quality injury care	Discrimination	# Patients Receiving Higher	Patients Perception of	Patients trust in healthcare system	# Injured seeking non-	Willingness to seek formal care	# Injured seeking care	# Patients taking other transport	# Patients in Definitive Care on	# Patients in Definitive Care	# Patients receiving Lower Quality	Willingness to remain in care	# Patients remaining in	# Patients Receiving Higher

									Quality Injury care	Health System		formal care				other Transport		Injury Care		care/rehab	Quality Rehab
911	20	Full-System Multicausal Loop	Total available workforce	GDP	Poverty	Community Conflict Violence etc	Availability of quality injury care	Discrimination	# Patients receiving Lower Quality Injury Care	Patients Perception of Health System	Patients trust in healthcare system	# Injured seeking non-formal care	Willingness to seek formal care	# Injured seeking care	# Patients taking an ambulance	# Patients in Definitive Care on Ambulance	# Patients in Definitive Care	# Patients Receiving Higher Quality Injury care	Willingness to remain in care	# Patients remaining in care/rehab	# Patients receiving Lower Quality Rehab
912	20	Full-System Multicausal Loop	Total available workforce	GDP	System level funding	Available Funding for injury care	Availability of quality injury rehab or follow-up	Discrimination	# Patients Receiving Higher Quality Injury care	Patients Perception of Health System	Patients trust in healthcare system	# Injured seeking non-formal care	Willingness to seek formal care	# Injured seeking care	# Patients taking an ambulance	# Patients in Definitive Care on Ambulance	# Patients in Definitive Care	# Patients receiving Lower Quality Injury Care	Willingness to remain in care	# Patients remaining in care/rehab	# Patients receiving Lower Quality Rehab
913	20	Full-System Multicausal Loop	Total available workforce	GDP	Poverty	Community Conflict Violence etc	Availability of quality injury rehab or follow-up	Discrimination	# Patients Receiving Higher Quality Injury care	Patients Perception of Health System	Patients trust in healthcare system	# Injured seeking non-formal care	Willingness to seek formal care	# Injured seeking care	# Patients taking other transport	# Patients in Definitive Care on other Transport	# Patients in Definitive Care	# Patients receiving Lower Quality Injury Care	Willingness to remain in care	# Patients remaining in care/rehab	# Patients receiving Lower Quality Rehab
914	20	Full-System Multicausal Loop	Total available workforce	GDP	Poverty	Community Conflict Violence etc	Availability of quality injury rehab or follow-up	Discrimination	# Patients Receiving Higher Quality Injury care	Patients Perception of Health System	Patients trust in healthcare system	# Injured seeking non-formal care	Willingness to seek formal care	# Injured seeking care	# Patients taking other transport	# Patients in Definitive Care on other Transport	# Patients in Definitive Care	# Patients receiving Lower Quality Injury Care	Willingness to remain in care	# Patients remaining in care/rehab	# Patients Receiving Higher Quality Rehab
915	20	Full-System Multicausal Loop	Total available workforce	GDP	System level funding	Available Funding for injury care	Availability of Ambulance Service	Geographical accessibility	Availability of other transport	# Patients taking other transport	# Patients in Definitive Care on other Transport	# Patients in Definitive Care	# Patients receiving Lower Quality Injury Care	Patients Perception of Health System	Patients trust in healthcare system	# Injured seeking non-formal care	Willingness to seek formal care	# Injured seeking care	# Patients taking an ambulance	# Patients in Definitive Care on Ambulance	# Patients in Definitive care on time
916	20	Full-System Multicausal Loop	Total available workforce	GDP	System level funding	Available Funding for injury care	Availability of quality injury care	Discrimination	# Patients receiving Lower Quality Injury Care	Patients Perception of Health System	Patients trust in healthcare system	# Injured seeking non-formal care	Willingness to seek formal care	# Injured seeking care	# Patients taking an ambulance	# Patients in Definitive Care on Ambulance	# Patients in Definitive Care	# Patients Receiving Higher Quality Injury care	Willingness to remain in care	# Patients remaining in care/rehab	# Patients receiving Lower Quality Rehab
917	20	Full-System Multicausal Loop	Total available workforce	GDP	System level funding	Available Funding for injury care	Availability of quality injury care	Discrimination	# Patients Receiving Higher Quality Injury care	Patients Perception of Health System	Patients trust in healthcare system	# Injured seeking non-formal care	Willingness to seek formal care	# Injured seeking care	# Patients taking an ambulance	# Patients in Definitive Care on Ambulance	# Patients in Definitive Care	# Patients receiving Lower Quality Injury Care	Willingness to remain in care	# Patients remaining in care/rehab	# Patients receiving Lower Quality Rehab
918	20	Full-System Multicausal Loop	Total available workforce	GDP	System level funding	Available Funding for injury care	Availability of quality injury rehab or follow-up	Discrimination	# Patients receiving Lower Quality Injury Care	Patients Perception of Health System	Patients trust in healthcare system	# Injured seeking non-formal care	Willingness to seek formal care	# Injured seeking care	# Patients taking other transport	# Patients in Definitive Care on other Transport	# Patients in Definitive Care	# Patients Receiving Higher Quality Injury care	Willingness to remain in care	# Patients remaining in care/rehab	# Patients receiving Lower Quality Rehab
919	20	Full-System Multicausal Loop	Total available workforce	GDP	System level funding	Available Funding for injury care	Availability of quality injury rehab or follow-up	Discrimination	# Patients receiving Lower Quality Injury Care	Patients Perception of Health System	Patients trust in healthcare system	# Injured seeking non-formal care	Willingness to seek formal care	# Injured seeking care	# Patients taking other transport	# Patients in Definitive Care on other Transport	# Patients in Definitive Care	# Patients Receiving Higher Quality Injury care	Willingness to remain in care	# Patients remaining in care/rehab	# Patients Receiving Higher Quality Rehab
920	20	Full-System Multicausal Loop	Total available workforce	GDP	Poverty	Community Conflict Violence etc	Availability of quality injury rehab or follow-up	Discrimination	# Patients receiving Lower Quality Injury Care	Patients Perception of Health System	Patients trust in healthcare system	# Injured seeking non-formal care	Willingness to seek formal care	# Injured seeking care	# Patients taking an ambulance	# Patients in Definitive Care on Ambulance	# Patients in Definitive Care	# Patients Receiving Higher Quality Injury care	Willingness to remain in care	# Patients remaining in care/rehab	# Patients receiving Lower Quality Rehab
921	20	Full-System Multicausal Loop	Total available workforce	GDP	Poverty	Community Conflict Violence etc	Availability of quality injury rehab or follow-up	Discrimination	# Patients Receiving Higher Quality Injury care	Patients Perception of Health System	Patients trust in healthcare system	# Injured seeking non-formal care	Willingness to seek formal care	# Injured seeking care	# Patients taking an ambulance	# Patients in Definitive Care on Ambulance	# Patients in Definitive Care	# Patients receiving Lower Quality Injury Care	Willingness to remain in care	# Patients remaining in care/rehab	# Patients receiving Lower Quality Rehab
922	20	Full-System Multicausal Loop	Total available workforce	GDP	Poverty	Community Conflict Violence etc	Availability of quality injury rehab or follow-up	Discrimination	# Patients Receiving Higher Quality Injury care	Patients Perception of Health System	Patients trust in healthcare system	# Injured seeking non-formal care	Willingness to seek formal care	# Injured seeking care	# Patients taking an ambulance	# Patients in Definitive Care on Ambulance	# Patients in Definitive Care	# Patients receiving Lower Quality Injury Care	Willingness to remain in care	# Patients remaining in care/rehab	# Patients Receiving Higher Quality Rehab

923	20	Full-System Multicausal Loop	Total available workforce	GDP	System level funding	Available Funding for injury care	Availability of quality injury care	Discrimination	# Patients receiving Lower Quality Injury Care	Patients Perception of Health System	Patients trust in healthcare system	# Injured seeking non-formal care	Willingness to seek formal care	# Injured seeking care	# Patients taking other transport	# Patients in Definitive Care on other Transport	# Patients in Definitive Care	# Patients Receiving Higher Quality Injury care	Willingness to remain in care	# Patients remaining in care/rehab	# Patients receiving Lower Quality Rehab
924	20	Full-System Multicausal Loop	Total available workforce	GDP	Poverty	Individual wealth	Availability of other transport	Geographical accessibility	Availability of Ambulance Service	# Patients taking an ambulance	# Patients in Definitive Care on Ambulance	# Patients in Definitive Care	# Patients Receiving Higher Quality Injury care	Patients Perception of Health System	Patients trust in healthcare system	# Injured seeking non-formal care	Willingness to seek formal care	# Injured seeking care	# Patients taking other transport	# Patients in Definitive Care on other Transport	# Patients in Definitive care on time
925	20	Full-System Multicausal Loop	Total available workforce	GDP	Poverty	Individual wealth	Availability of other transport	Geographical accessibility	Availability of Ambulance Service	# Patients taking an ambulance	# Patients in Definitive Care on Ambulance	# Patients in Definitive Care	# Patients Receiving Higher Quality Injury care	Patients Perception of Health System	Patients trust in healthcare system	# Injured seeking non-formal care	Willingness to seek formal care	# Injured seeking care	# Patients taking other transport	# Patients in Definitive Care on other Transport	# Patients in Definitive care Delayed
926	20	Full-System Multicausal Loop	Total available workforce	GDP	System level funding	Available Funding for injury care	Availability of quality injury rehab or follow-up	Discrimination	# Patients receiving Lower Quality Injury Care	Patients Perception of Health System	Patients trust in healthcare system	# Injured seeking non-formal care	Willingness to seek formal care	# Injured seeking care	# Patients taking an ambulance	# Patients in Definitive Care on Ambulance	# Patients in Definitive Care	# Patients Receiving Higher Quality Injury care	Willingness to remain in care	# Patients remaining in care/rehab	# Patients receiving Lower Quality Rehab
927	20	Full-System Multicausal Loop	Total available workforce	GDP	System level funding	Available Funding for injury care	Availability of quality injury rehab or follow-up	Discrimination	# Patients receiving Lower Quality Injury Care	Patients Perception of Health System	Patients trust in healthcare system	# Injured seeking non-formal care	Willingness to seek formal care	# Injured seeking care	# Patients taking an ambulance	# Patients in Definitive Care on Ambulance	# Patients in Definitive Care	# Patients Receiving Higher Quality Injury care	Willingness to remain in care	# Patients remaining in care/rehab	# Patients Receiving Higher Quality Rehab
928	20	Full-System Multicausal Loop	Total available workforce	GDP	Poverty	Community Conflict Violence etc	Availability of quality injury rehab or follow-up	Discrimination	# Patients receiving Lower Quality Injury Care	Patients Perception of Health System	Patients trust in healthcare system	# Injured seeking non-formal care	Willingness to seek formal care	# Injured seeking care	# Patients taking other transport	# Patients in Definitive Care on other Transport	# Patients in Definitive Care	# Patients Receiving Higher Quality Injury care	Willingness to remain in care	# Patients remaining in care/rehab	# Patients Receiving Higher Quality Rehab

Supplementary material 8: Example causal loop diagrams (CLDs)

Central feedback loops

Of the central feedback loops, the reinforcing loops primarily include a sequence of positive feedback loops which should improve health outcomes either due to patients choosing to seek care and reaching care on time, or due to receiving higher quality injury care or rehabilitation. Balancing loops are primarily seen in relation to the negative association with health outcomes of patients incurring delays in reaching definitive care (B1, B2) or receiving lower quality care (B3) or rehab (B4).

For illustration, reinforcing loop R3 starts with the factor ‘patients trust in the health care system’, which influences ‘willingness to seek formal care’, which impacts ‘the number of injured persons seeking care’, and subsequently the ‘number of patients taking an ambulance’, leading to improved health outcomes which in turn closes with improved ‘patient trust in the healthcare system’ . Reinforcing loops R4 and R5, extend the impact of R3 from ‘number of patients taking an ambulance’, influencing ‘the number of patients in definitive care on [who arrived by] ambulance’, leading to an increase in the ‘number of patients in definitive care [who arrived] on time’ and ‘number of patients taking other transport’ influencing in turn ‘the number of patients in definitive care on [who arrived by] other transport’, respectively, leading to improved ‘health outcomes’ and improved ‘patient trust in the healthcare System’. The R4 and R5 loops further extend into the balancing loops B1 and B2 respectively, through ‘patients in definitive care on [who arrived by] ambulance’/ ‘patients in definitive care on [who arrived by] other transport’ which effects ‘number of patients in definitive care [who are] delayed’ and subsequently leads to deteriorated ‘health outcomes’ and ‘patient trust in the healthcare system’. Whereas patients arriving by ambulance or other transport might be expected to have less delay per se, delays suffered across multiple patients will be determined by the number of ambulances or other transport options available and geographical isolation. The effects of loops R6, R7 and B3 are extended from ‘number patients in definitive care’ with its positive impact on ‘number patients [who] remain in care/rehab’, which in turn, depending on the availability of quality rehabilitation, impacts positively ‘number patients receiving higher quality rehab’ or ‘number patients receiving lower quality rehab’ each impacting either positively (R8) or negatively (B4) on health outcomes, closing the loop again with impact on ‘patient trust in the healthcare system’.

Supplementary Material Figure 4: The 6 main reinforcing (R3, R4, R5, R6, R7 and R8) feedback loops that affect the injury care pathway

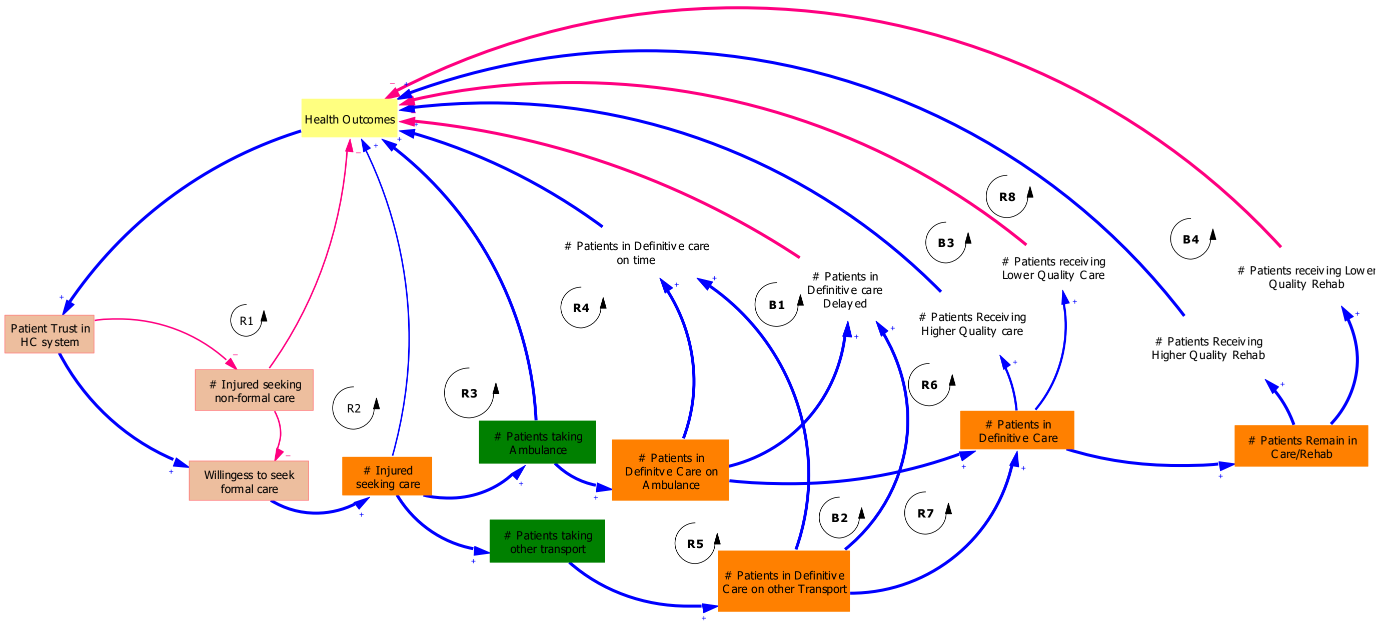

Blue arrows denote a positive causal relationship between pairs of variables and red arrows denote a negative causal relationship between pairs of variables. Reinforcing feedback loops are represented by the R symbol and balancing loops by a B symbol. # denotes number of. Positive relationships between pairs of variables are in blue and negative relationships in red font arrows.

Example central feedback loops are individually displayed below.

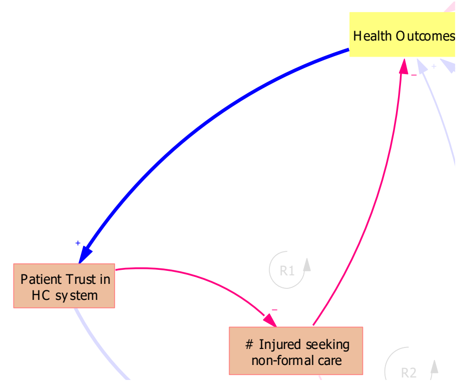

Reinforcing Feedback loop R1, involving injured patients seeking non-formal care

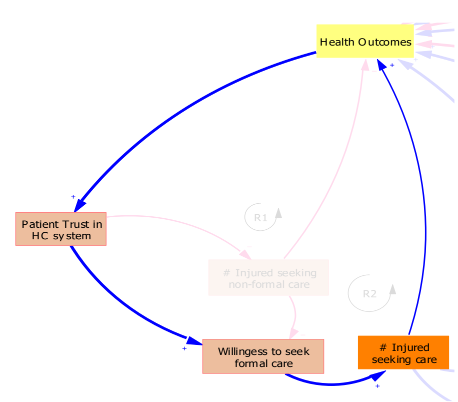

Reinforcing Feedback loop R2: involving injured patients seeking formal care

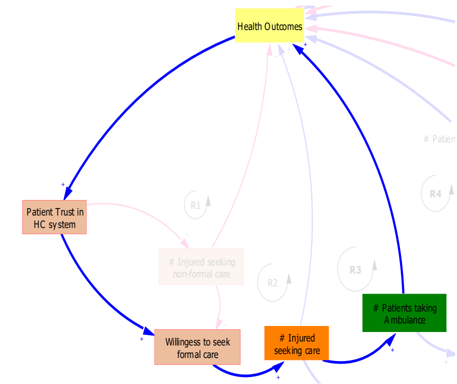

Reinforcing Feedback Loop R3: involving injured patients taking an ambulance

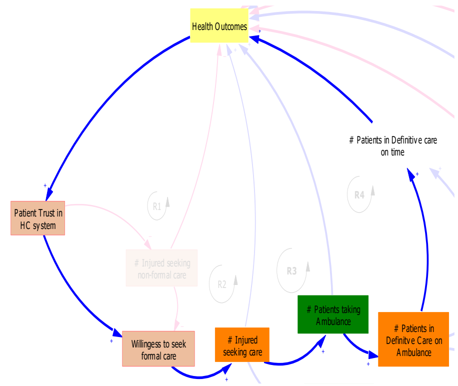

Reinforcing Feedback Loop R4, involving injured patients reaching definitive care on time on ambulance

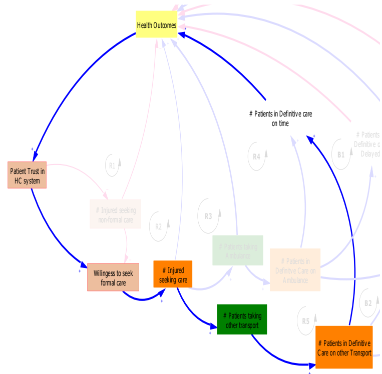

Reinforcing feedback loop R5, involving injured patients reaching definitive care on time using other transport

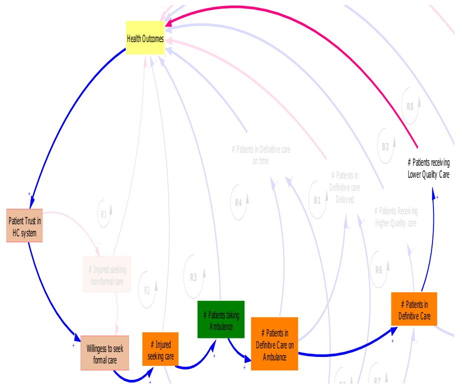

Reinforcing feedback loop R6, involving injured patients reaching definitive care on time using ambulances

<u>Supplementary Material 9: Causal loop diagram of the services offered at healthcare or rehabilitation facilities.</u>

Supplementary Material Figure 5: Isolated health services CLD

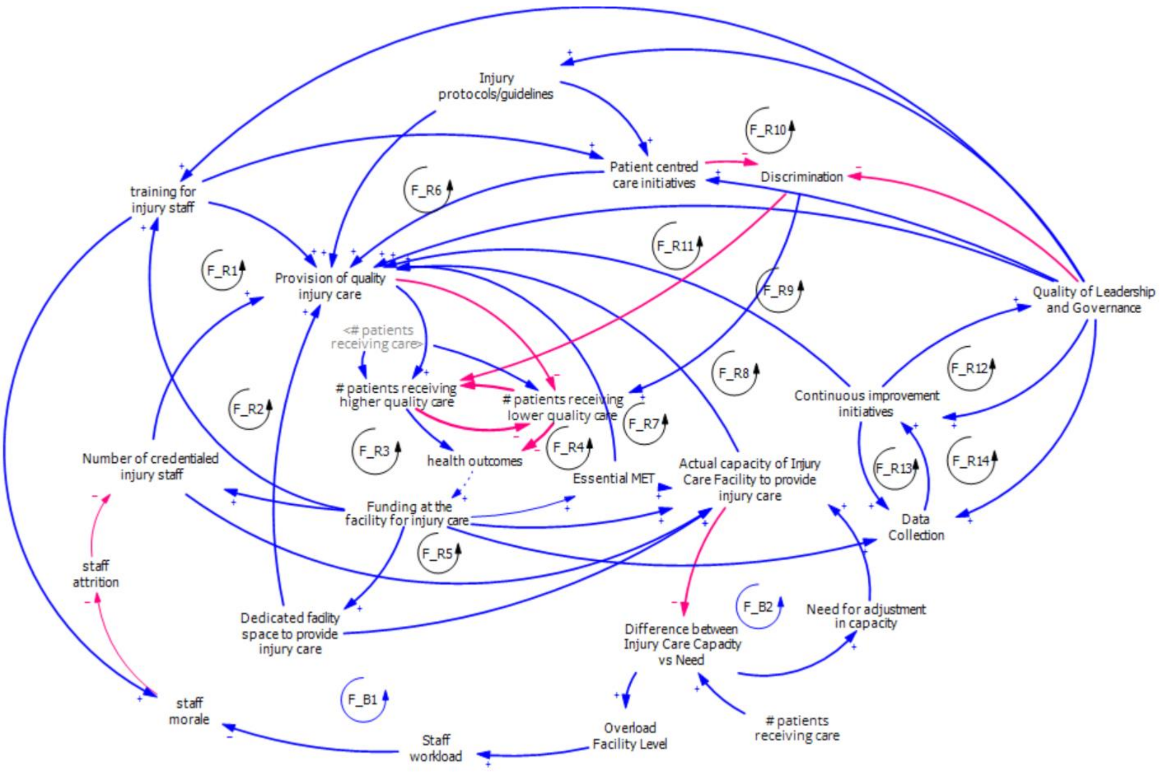

HRH is human resources for health, QI is quality improvement, EMT is essential medicines and technologies. Positive relationships between pairs of variables are in blue and negative relationships in red font arrows. 14 reinforcing and 2 balancing feedback loops are present, noted with the initial F for Facility followed by the type and number.

Supplementary material 10: Example feedback loops involving trust in the healthcare system

A reinforcing loop, R9 and a balancing loop, B5 offer contrasting examples of how the factor “trust in the healthcare system” acts within a complex system. R9 starts with ‘patients perception of health system’, an increase in which influences an increase in ‘patients trust in the healthcare system’, in turn leading to more ‘willingness to receive care’, consequently leading to higher ‘number of patients in definitive care’ and in turn to greater ‘number of patients receiving higher quality care’, closing the loop with via the improved ‘patients perception of health systems’, and thence ‘patient trust in the healthcare system’. On the other hand, the balancing loop B5 considers that the ‘number of patients in definitive care’, could lead to a higher ‘number of patients receiving lower quality care’, it can overload the capacity of the services (see Supplementary Material 8: Causal loop diagram of the services offered at healthcare or rehabilitation facilities), leading to quality of care provided being eroded, with a negative impact on ‘patient perception of health systems’ forming a balancing feedback loop.

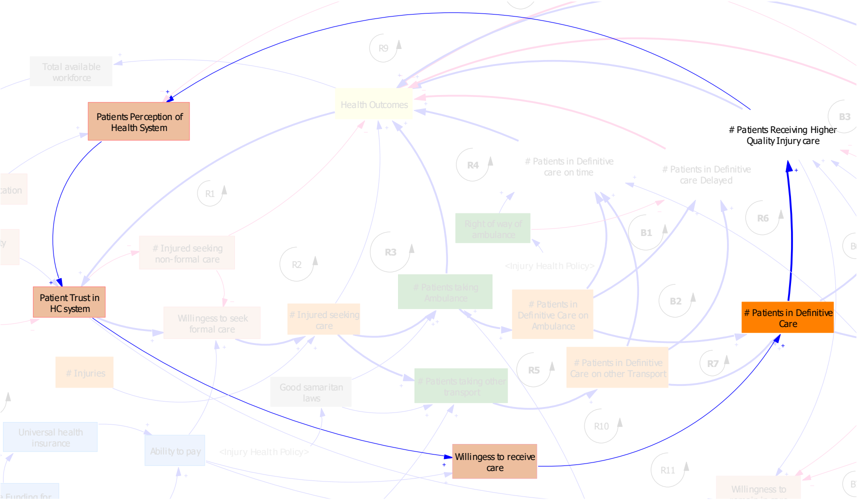

Reinforcing feedback loop R9

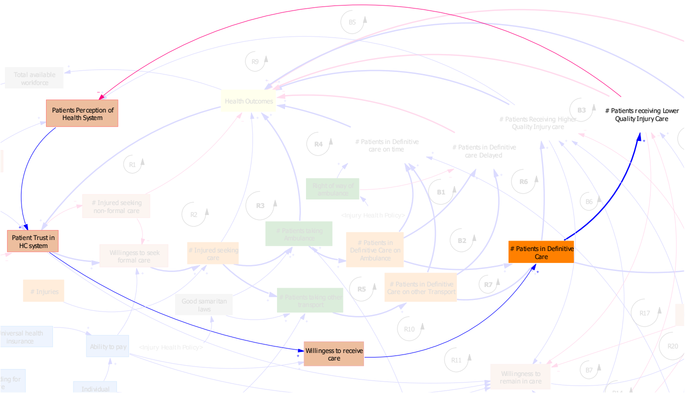

Feedback loop B5

Feedback loop B4 shows the effects of trust on the more downstream outcomes of receipt of rehabilitation. Like balancing loop B5, outcomes of increased patients in definitive care and rehab, can lead to more patients receiving lower quality care due to the system operating beyond capacity, leading subsequently to worse health outcomes, which in turn reduces patient trust in the healthcare system, meaning that B4 has a diminishing effect on health outcomes.

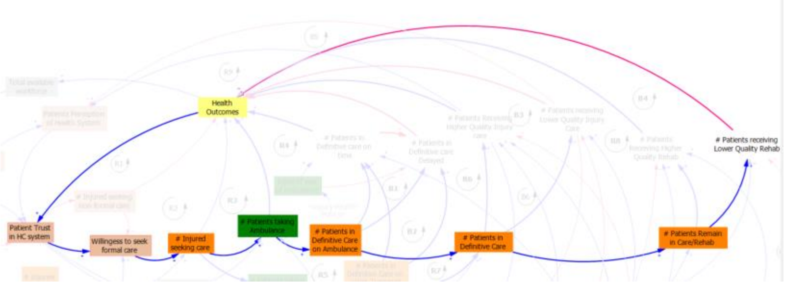

Feedback loop B4

Whereas feedback loop B6 is similar to B4, with the difference that here patients take other transport as opposed to an ambulance. A comparison of the two balancing loops, B4 and B6, suggests that they both have a diminishing effect on health outcomes, regardless of whether patients take an ambulance or other transport.

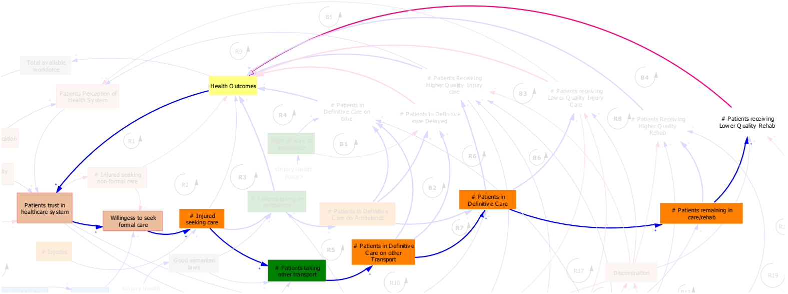

Feedback loop B6

Further complexities involving trust can be seen in loop B10. This shows the diminishing effect of trust on health outcomes leading to patients receiving lower quality injury care, and rehab. There are three negative causal relationships in this loop, hence forming a balancing loop. Here, improved patient trust in the health system leads to more willingness to seek formal care and in turn to more injured patients seeking care who ultimately receive higher quality injury care, which in turn leads to more willingness to remain in care, hence resulting in more patients remaining in care/rehab. However, an increase in the number of patients remaining in care could mean that more patients receive lower quality rehab, resulting from increased demand on the care facilities, which in turn leads to worse health outcomes. Worse health outcomes eventually reduce total available workforce, thence GDP, which leads to less funding at system level and funding for injury care and in return less availability of quality injury and rehab care. Eventually (see health services diagram in Supplementary Material 8) lower quality injury care or rehab leads to more discrimination meaning more patients receive lower quality injury care, which leads to worse patient perception of health system and in turn to reduced patient trust in the health care system.

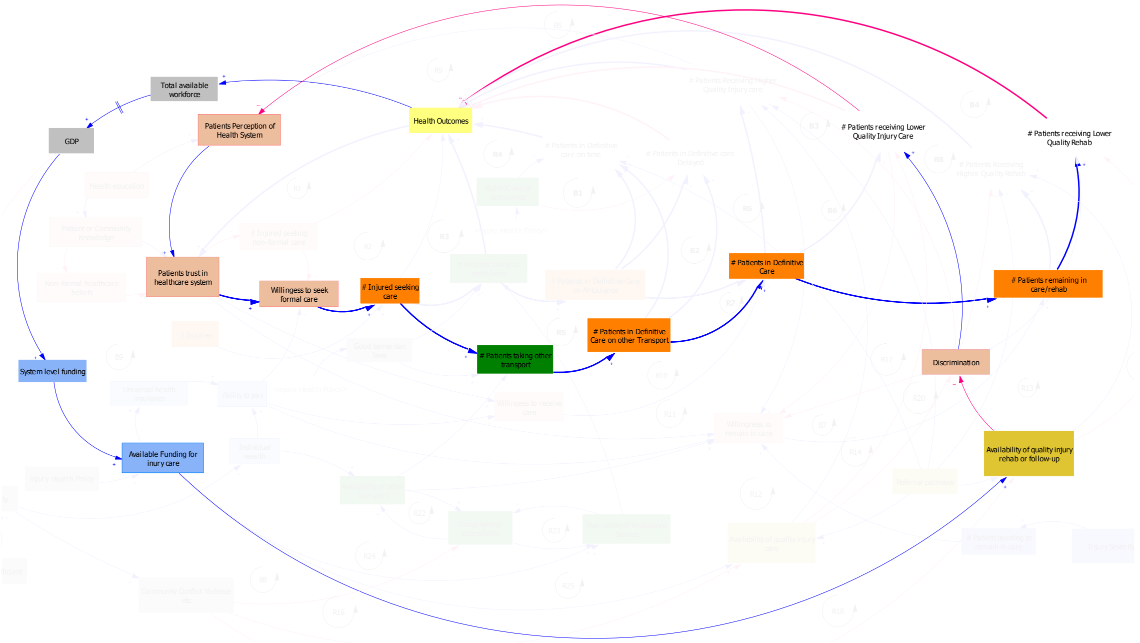

Balancing loop B10

<u>Supplementary Material 11: Example feedback loops involving discrimination during healthcare.</u>

‘Discrimination’ forms 360 loops in total of which 35 are reinforcing, which have the potential to influence an ever-increasing vicious negative effect on health outcomes. For example, in R20, an increase in discrimination leads to a reduction in ‘number patients receiving higher quality injury care’ (negative), this reduction in turn leads to worsened ‘health outcomes’, leading to decrease in ‘total available workforce’, in turn to an decrease in GDP, in ‘funding at system level’, in ‘available funding

for injury care', and in turn to an decrease in 'availability of quality injury care facility', and thus an increase in discrimination (negative), closing the loop with two (even) negative causal relationships in total, thus forming a reinforcing loop.

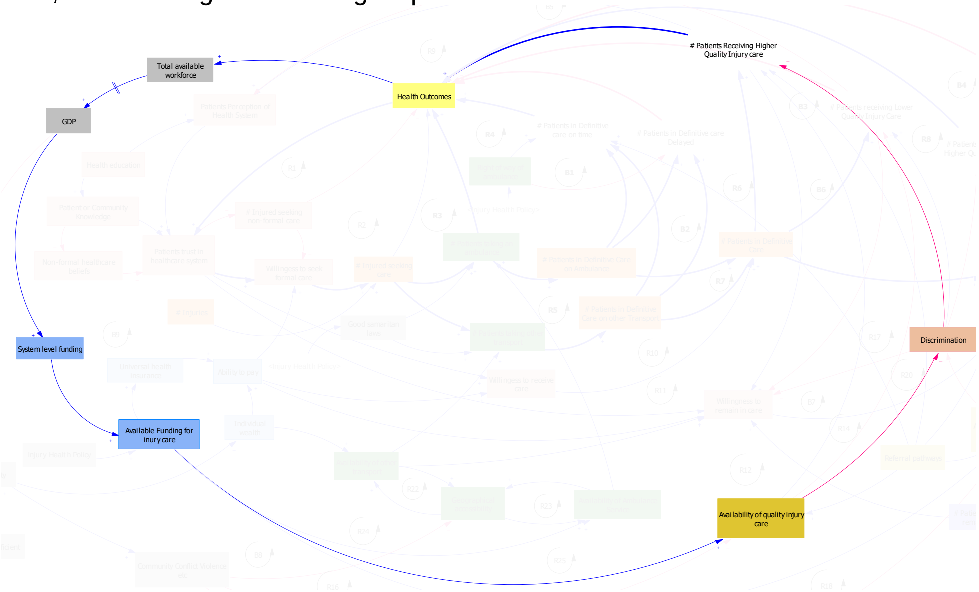

Feedback loop R20

Similarly, R21 has similar effects, with the difference that it includes the impact that more discrimination leads to greater 'number patients receiving lower quality rehabilitation' which in turn leads to reduced health outcomes. An increase in discrimination, or decrease in other variables part of these loops (i.e. available funding or availability of quality injury care facility), can have a vicious negative effect on health outcomes as a consequence.

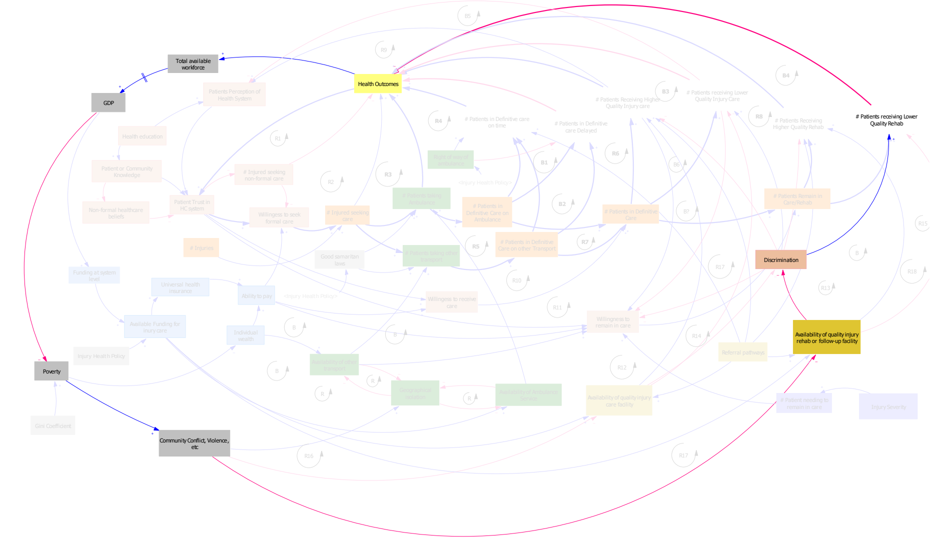

Feedback loop R21

Supplementary material 12: Example CLDs involving macro-factors

Example reinforcing feedback loops (R12 and R13) act at the macro-level and show the impact of injury care on the economy. Improved health outcomes lead to the availability of a larger and healthier workforce, which over time increases the country's total GDP, leading to more available funding, which in turn leads to more 'availability of quality injury care facility'(for R12) and 'availability of quality injury rehab or follow-up facility' (for R13) , each in turn leading to a higher 'number patients receiving

higher injury quality care' and 'number of patients receiving higher quality rehabilitation', respectively. These in turn lead to improved health outcomes.

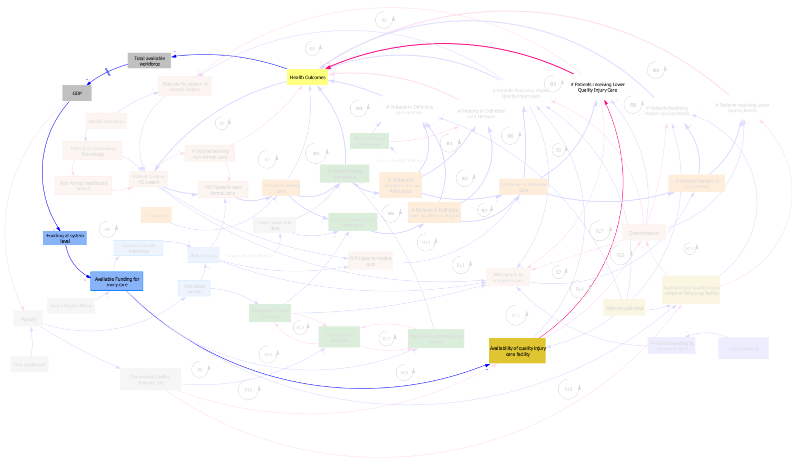

Feedback loop R12

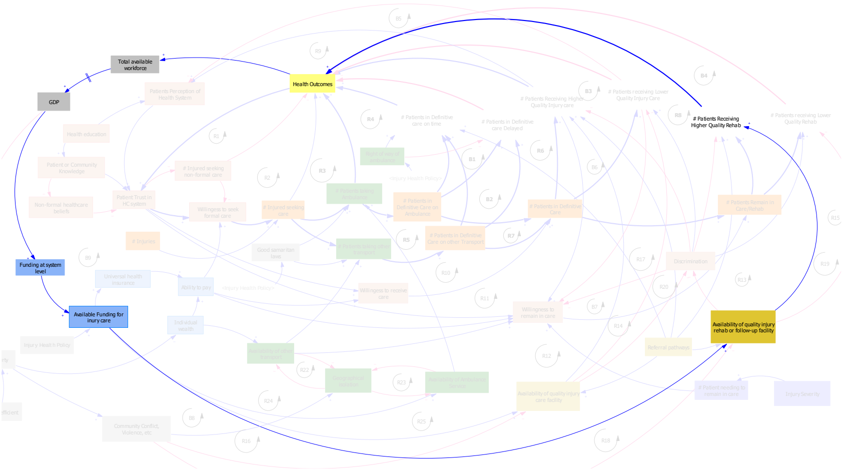

Feedback loop R13

The impact of poverty at a macro-level forms reinforcing feedback loops (R17), where a change i.e. reduction in GDP leads to a change increase in poverty (a negative association), whereas more poverty can lead to more 'community conflict, violence, etc.", which reduces the 'availability of quality injury care facility', in turn reducing the 'number patients receiving higher quality care', leading to a reduction in good health outcomes, which means reduced 'total available workforce' and, in time, GDP, thus closing the loop in a continuous vicious circle of reduced health outcomes. The opposite, a continuous vicious circle of increasing good health outcomes would apply in the case of increased GDP.

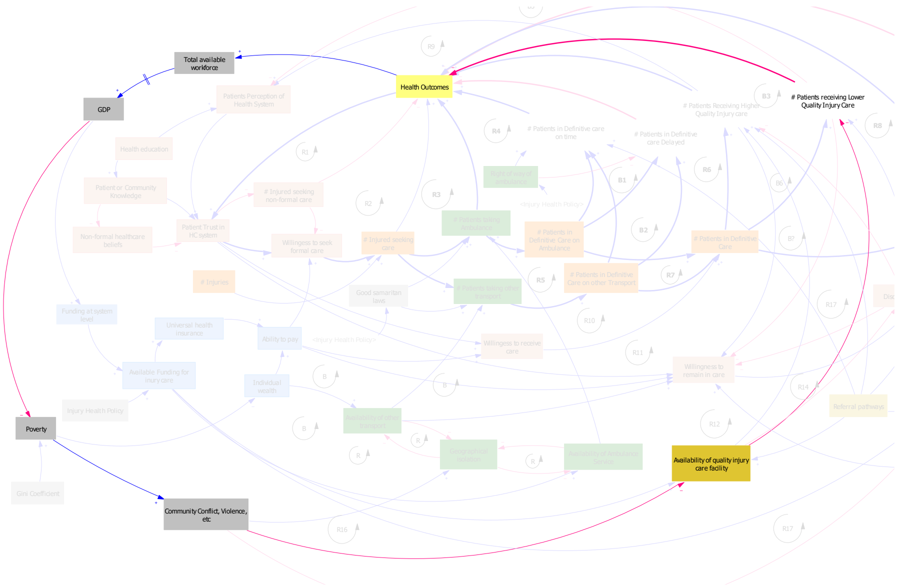

Reinforcing feedback loop R17

Geographical accessibility is involved in 128 balancing and reinforcing feedback loops in total, depending on its connection to provision of injury care/rehabilitation or timeliness of transport. For example, reinforcing loop R24, starts with geographical accessibility, which if increased leads to more ‘availability of ambulance service’ and in turn more ‘number patients taking an ambulance’, and more ‘patients in definitive care on ambulance’ and in turn to more ‘patients in definitive care on time’. This means improved health outcomes, having in turn a positive impact on ‘total available workforce’ and GDP, which lead to less poverty, and less ‘community conflict, violence, etc.’ which in turn improves geographical accessibility, thus closing the loop of an ever-increasing vicious circle. However, the opposite effect can be present that of an ever-decreasing vicious circle, if geographical accessibility was to be reduced.

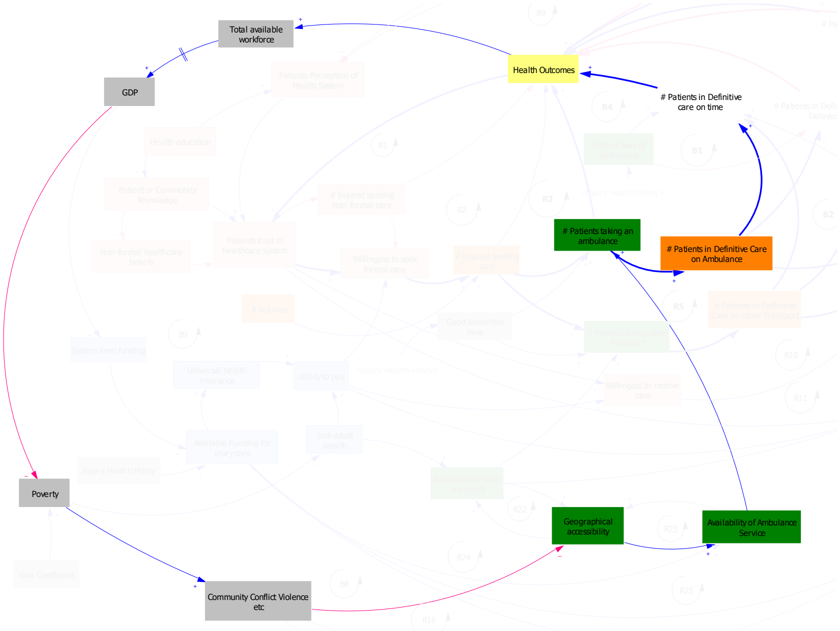

Reinforcing Feedback loop R24

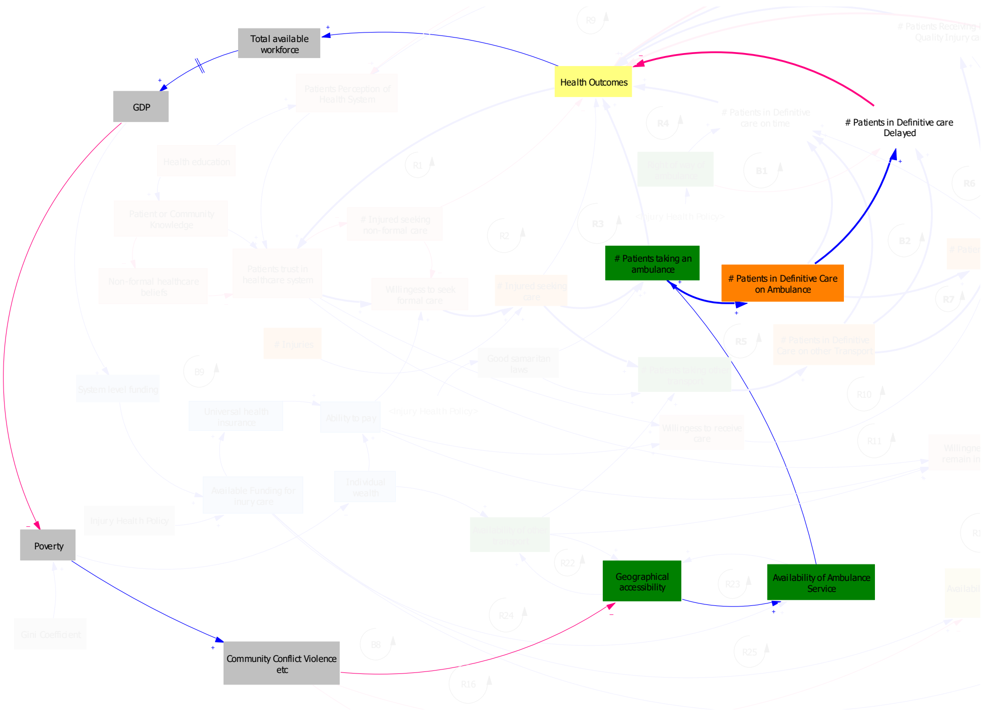

Balancing Feedback loop B8

On the other hand, as an example, balancing feedback loop B8, diminishes the impact of geographical accessibility. B8 includes the same links as R24, (which starts with increased geographical accessibility) but differs with the inclusion of the ‘number patients in definitive care delayed’, which, if increased, leads to reduced health outcomes (negative association), in time leading to a reduction in ‘total workforce’ and ‘GDP’ in turn to increased ‘poverty’ and more ‘community conflict, violence etc’ and in turn to reduced ‘geographical accessibility thus closing the balancing loop.

‘Funding at system level’ and ‘available funding for injury care’ are involved in 405 feedback loops in total of which the majority, 234, are reinforcing loops and 171 balancing loops. For example, R25 shows that an increase in funding leads to more ‘availability of quality injury care facility’, which in turn leads to a reduced ‘number of patients receiving lower quality injury care’ and, in turn, less negative impact on health outcomes, which leads to a greater ‘total available workforce’ and GDP, leading to more funding. However, the opposite effect can come into effect if a reduction in funding occurs, which can lead to deteriorated health outcomes. For example, in B9 an increase in universal health insurance, leads to greater ‘ability to pay’ for patients, leading to more ‘willingness to receive care’, hence a greater ‘number patients in definitive care’ which can lead to a greater ‘number patients receiving lower quality care’ and further deterioration in health outcomes, which leads to reduced ‘total available workforce’ and GDP hence less funding at system level and ‘availability of funding for injury care’, thus closing the loop with reduced ‘universal health insurance’, meaning that it diminishes the effect of funding on the whole.

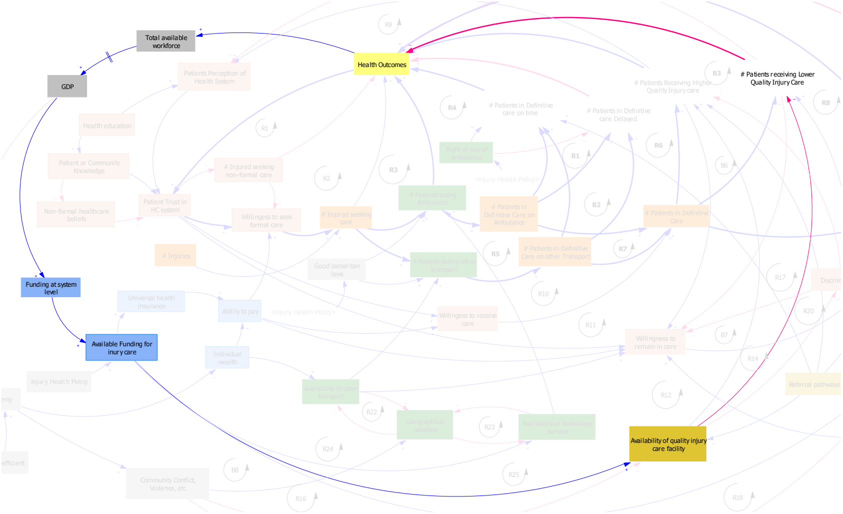

Feedback loop R25

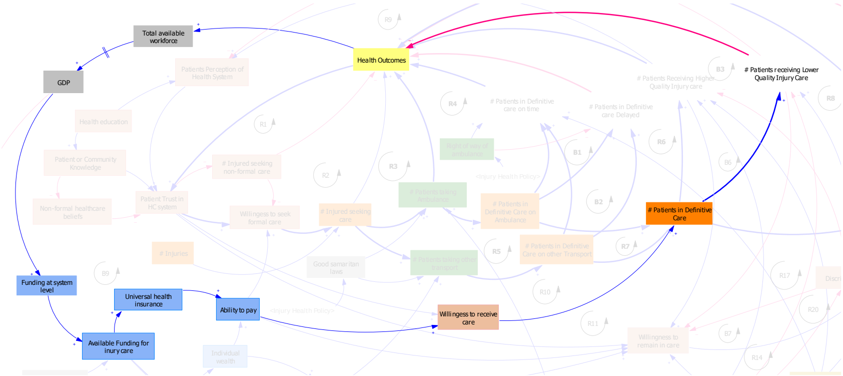

Feedback loop B9
